# Impact of Storage Conditions on the Breast Milk Peptidome

**DOI:** 10.3390/nu12092733

**Published:** 2020-09-08

**Authors:** Vanessa Howland, Maik Klaedtke, Johanna Ruhnau, Vishnu M. Dhople, Hans J. Grabe, Uwe Völker, Matthias Heckmann, Elke Hammer

**Affiliations:** 1 Department of Neonatology and Paediatric Intensive Care, University Medicine Greifswald, D-17475 Greifswald, Germany; vanessa.howland@uni-greifswald.de; 2 Department of Functional Genomics, Interfaculty Institute for Genetics and Functional Genomics, University Medicine Greifswald, D-17475 Greifswald, Germany; maik.k5@freenet.de (M.K.); dhoplevm@uni-greifswald.de (V.M.D.); voelker@uni-greifswald.de (U.V.); 3 Department of Neurology, University Medicine Greifswald, D-17475 Greifswald, Germany; johanna.ruhnau@uni-greifswald.de; 4 Department of Psychiatry & Psychotherapy, University Medicine Greifswald, D-17475 Greifswald, Germany; grabeh@uni-greifswald.de

**Keywords:** human breast milk, peptidome, LC-MS/MS, storage conditions, temperature

## Abstract

Human donor milk (HDM) provides appropriate nutrition and offers protective functions in preterm infants. The aim of the study is to examine the impact of different storage conditions on the stability of the human breast milk peptidome. HDM was directly frozen at −80 °C or stored at −20 °C (120 h), 4 °C (6 h), or room temperature (RT for 6 or 24 h). The milk peptidome was profiled by mass spectrometry after peptide collection by ultrafiltration. Profiling of the peptidome covered 3587 peptides corresponding to 212 proteins. The variance of the peptidome increased with storage temperature and time and varied for different peptides. The highest impact was observed when samples were stored at RT. Smaller but significant effects were still observed in samples stored at 4 °C, while samples showed highest similarity to those immediately frozen at −80 °C when stored at −20 °C. Peptide structures after storage at RT for 24 h point to the increased activity of thrombin and other proteases cleaving proteins at lysine/arginine. The results point to an ongoing protein degradation/peptide production by milk-derived proteases. They underline the need for immediate freezing of HDM at −20 °C or −80 °C to prevent degradation of peptides and enable reproducible investigation of prospectively collected samples.

## 1. Introduction

The composition of breast milk includes bioactive factors required to provide appropriate nutrition for developmental processes and offers simultaneously protective functions for the children as well as the mammary glands during lactation [1,2,3]. Many observational studies indicate that a mother’s own milk compared to term or preterm formula confers protection against invasive infection and necrotizing enterocolitis (NEC), resulting in reduced mortality in preterm infants [4,5,6]. The recent Cochrane meta-analysis found evidence that human donor milk (HDM), compared to formula also significantly reduced rates of NEC [7]. Furthermore, breastfeeding is also associated with numerous positive effects for term infants and their mothers as well. A recent meta-analysis indicated protection against infections in children, increases in intelligence, and probable reductions in overweight and diabetes. For nursing women, breastfeeding gave protection against breast cancer, and it might also protect against ovarian cancer and type 2 diabetes [8]. Therefore, feeding HDM is recommended when milk from the infant’s own mother is not available [9,10]. Positive effects are attributed to several components contained in breast milk, like proteins, peptides, and amino acids. However, the mechanism by which the components in milk protect preterm infants from NEC is still not yet clear. The human milk peptidome might play a role [11,12].

Therefore, recently, the focus of investigations has switched from proteins to peptides. Human milk has been reported to contain more than 1100 unique peptides derived from 42 milk proteins already produced in the mammary gland by milk proteases. By comparison to peptide sequences and their known functions, more than 300 of these peptides are considered as bioactive [13]. Until now, antimicrobial, immunomodulatory, and opioid-like functions, as well as antithrombotic and anti-hypertensive effects, have been reported for bioactive peptides [14,15,16,17]. Findings indicate that milk peptides can have additional physiological effects, such as antioxidant activity [18]. They might be able to support growth and bone health as well as immune responses and the cardiovascular system in neonates fed by breast milk [19]. Therefore, storage conditions might play an important role in processing HDM to preserve bioactive milk peptides. Storage conditions of a mother’s own milk for gavage feeding of preterm infants are also involved. So far, studies have focused on the impact of storage temperatures and durations on bacterial contamination, immunological components, nutritional quality, reviewed in [20], or metabolite contents and stability [21], and they have been considered for the development of guidelines for the care of breastfeeding mothers and infants [22]. Additionally, pasteurization of HDM was shown to affect bioactive peptide release [23] and further related studies are ongoing [24].

Furthermore, storage conditions play an important role in pre-analytical handling of the samples. Recently, Zhou et al. identified peptides of beta-casein 2 (CSN2), which are related to immunocompetence and development, among 3182 non-redundant peptides in colostrum and transitional and mature milk using liquid chromatography/mass spectrometry technology [25]. As in this study, the reported peptide number for human milk repeatedly exceeded 3000 and the question arises as to whether these identified peptides are biologically present from the beginning or produced during sample storage and pre-analytical sample preparation. Before studying bioactive peptides—with the aim to improve the well-being of infants and identify options for disease prevention and treatment—reliable and repeatable methods are required and the pre-analytical variation has to be assessed and controlled. One of those factors is temperature after sampling during short-term storage or transport until optimal long-term storage at −80 °C. This is especially important in larger cohort studies with participants that are not hospitalized or when later lactation periods are covered, and mothers are already in their home environment.

Therefore, it was the aim of this study to examine the impact of different storage temperatures and durations on the stability of the human breast milk peptidome. The conditions chosen resemble infrastructural pre-requisites at home and in specialist practices as well as during transport (room temperature (RT), 4 °C, −20 °C) and are compared to directly freezing at −80 °C. For this purpose, we used a mass spectrometry (MS)-centered approach after extraction of peptides by ultrafiltration and analyzed whole peptide patterns as well as the kinetics of bioactive peptides.

## 2. Materials and Methods

### 2.1. Sample Collection

Human breast milk samples were collected from four anonymous mothers living in the area of Greifswald, Germany. All participants provided informed consent. Milk samples were collected after at least six weeks of lactation from mothers who delivered full term infants. All donors were healthy and gave birth to healthy infants. Milk samples were collected from one expression at the mother’s home between 8 a.m. and 10 a.m. Before sampling, the first droplets of milk were hand expressed, and afterwards the breast was cleaned using water and a washcloth, and samples of 20 mL were collected using clean electric breast pumps (Medela Medizintechnik, Dietersheim, Germany). The breast milk was collected into plastic tubes and transferred to the analytical center within an hour, aliquoted in 2000 µL aliquots and stored, as indicated in Section 2.2., in four replicates each. After exposure to the specific temperatures for the specified time period, aliquots were stored at −80 °C until analysis.

### 2.2. Storage Experiment

**Figure 1:**
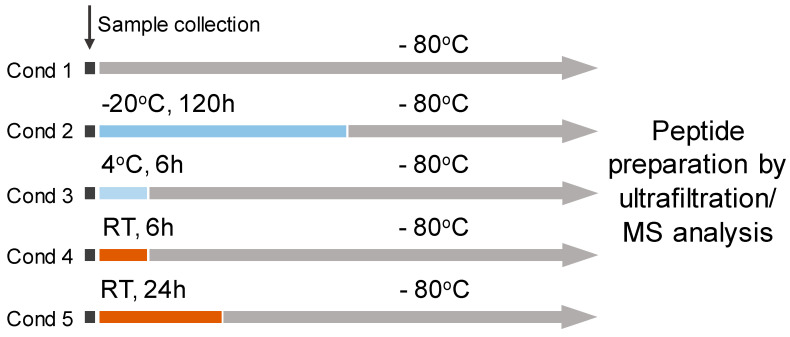
Study design for comparison of the stability of the human breast milk peptidome in different storage conditions.

The experiment should provide knowledge on the impact of storage temperature and duration on the human breast milk peptidome during the pre-analytical stage. The following conditions (Figure 1) were compared and analyzed in four technical replicates for each mother’s milk sample: immediate storage at −80 °C, (cond 1), at −20 °C after 120 h (cond 2), storage at 4 °C for 6 h (cond 3), and storage at room temperature (RT) for 6 h (cond 4) and 24 h (cond 5).

### 2.3. Sample Preparation

Samples were thawed at RT for 10 min and afterwards stored on ice for 20 min. Peptide extraction was carried out by a modified filter-aided methanol extraction, reported earlier [25]. Briefly, samples were centrifuged at 17,000× *g* for 30 min at 4 °C and the upper lipid layer removed. One hundred microliters of the collected skim milk were mixed with 25 µL methanol and incubated for 20 min at RT. Proteins were removed by centrifugation of samples through a 10 kDa molecular weight cut-off membrane (Vivacon^®^ 500, Sartorius, Goettingen, Germany) for 10 min at 14,000× *g*. Filtrates were dried by lyophilization at 0 °C and 1.030 mbar (Alpha1-4 LSC, Martin Christ Gefriertrocknungsanlagen GmbH, Osterode, Germany). Samples were reconstituted in 10 µL 1% acetic acid (AA) and desalted on C18 material (µZipTip^®^, Merck Millipore, Darmstadt, Germany).

### 2.4. Tandem Mass Spectrometry

The liquid chromatography–mass spectrometry (LC-MS) analysis was performed on a nanoAcquity UPLC (Waters Corporation, Washburn, MA, USA) coupled to an LTQ-Orbitrap Velos mass spectrometer (Thermo Electron, Bremen, Germany) equipped with a nano-electrospray ionization source (LC-ESI-MS/MS).

Peptides were trapped on a nanoAcquity UPLC 2G-V/M trap Symmetry C18 pre-column (2 cm, 180 µm i.d., 5 µm particle size, Waters) and afterwards separated on a nanoAcquity BEH130 C18 column (10 cm, 100 µm i.d., 1.7 µm particle size, Waters). The separation was achieved at a flow rate of 400 nL/min with a 99-min non-linear gradient of buffer A (0.5% DMSO in water with 0.1% AA) and buffer B (5% DMSO in ACN with 0.1% acetic acid, gradient: 1–5% buffer B in 2 min, 5–25% B in 63 min, 25–60% B in 25 min, 60–99% B in 2 min). Data were acquired in data-dependent mode. The MS automatically switches between Orbitrap-MS and LTQ-MS/MS acquisition to carry out the MS and MS/MS events. Survey full scan (MS1) data were recorded from m/z 325 to 1525 and were acquired in the Orbitrap at a resolution R = 30,000 with a target value of 1 × 10^6^. The twenty most intense double- and triple-charged ions depending on signal intensity were subjected to collision induced dissociation (CID) fragmentation with an isolation width of 2 Da and a target value of 1 × 10^4^ or with a maximum ion time of 100 ms (MS/MS). Target ions already selected for MS/MS were dynamically excluded for 60 s. General MS conditions were electrospray voltage, 1.5–1.7 kV; no sheath and auxiliary gas flow, capillary temperature of 300 °C. The ion selection threshold was 2000 counts for MS/MS, activation time 10 ms, and activation energy 35%.

### 2.5. Data Analysis

Proteome Discoverer 2.2 (Thermo Scientific, Bremen, Germany) was used for qualitative and quantitative MS data analysis. Peptide identification was accomplished using Sequest HT. Data were searched against an in-house library containing 509 milk human proteins and 10 contaminants (keratins), identified by mass spectrometric analyses of peptides in individual human milk samples immediately stored at −80 °C. MS raw data of samples stored at different conditions were analyzed separately for individual breast milk samples (five conditions in four replicates; *n* = 20) and searched against a reviewed human proteome FASTA database (Uniprot 2019_03). This smaller database was employed because of the required setting “non-specific cleavage” [15]. Peptide and fragment mass tolerance was 10 ppm and 0.6 Da, respectively. Methionine and protein N-terminal acetylation were the only variable modifications specified. Only peptides at high confidence (FDR < 0.01) were considered for further analysis.

Bioactive peptides were identified by a search of sequences and truncated sequences against the human milk protein peptide database [26]. Protease cleavage sites were inspected using the MEROPS database [27]. Peptide N- and C-termini were analyzed using WebLogo (https://weblogo.berkeley.edu/) [28].

Differential abundance of the 14 proteins representing the 10 highest abundant proteins per sample stored at −80 °C or stored for 120 h at RT before freezing was determined by application of a paired *t*-test to the mean values of the protein intensities of the four technical replicates per individual milk sample.

Variation between the milk samples from different mothers and the impact of the storage temperature was displayed by a principal component analysis (PCA) based on all quantified peptides per sample defined by the parameters “mother” and “storage condition”.

## 3. Results

### 3.1. Impact of Storage Conditions on the Composition of the Milk Peptidome

**Figure 2:**
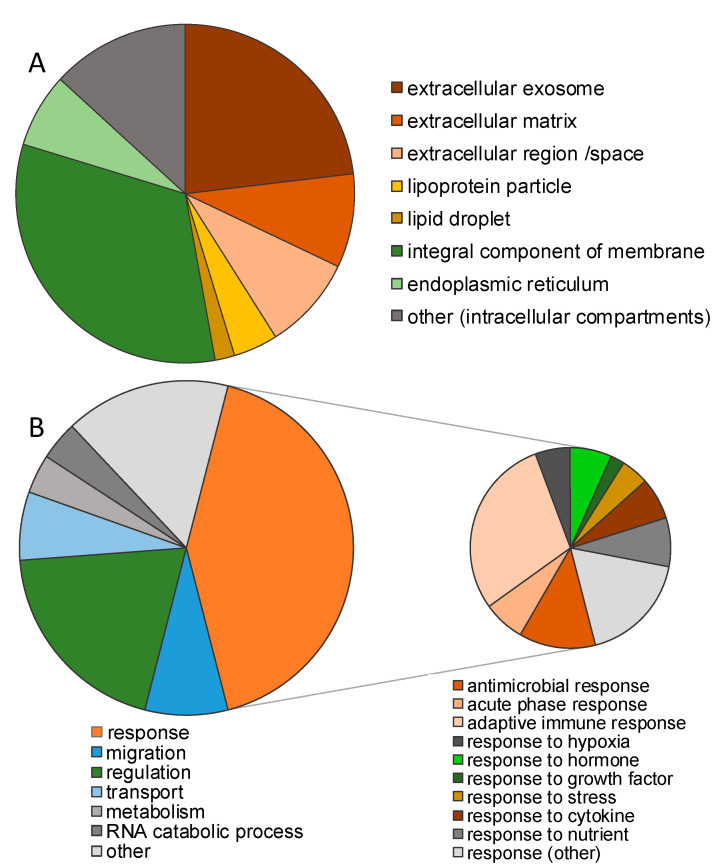
Assignment of peptides in the human milk peptidome to proteins (*n* = 212). (**A**) Protein localization and (**B**) protein function.

In this study, human breast milk of different participants was analyzed. The milk of one donor (T) was used to determine the technical variance and four samples (A–D) were used to study the impact of storage conditions. Analysis of the milk samples A–D immediately stored at −80 °C (control) revealed quantitative data on 3237 peptides corresponding to 204 proteins (Table S1). The majority of the proteins are assigned to the extracellular region, space, or exosomes or constitute integral membrane proteins (Figure 2A, Table S2). A higher proportion of these proteins functions in response to stimuli or cellular regulation. Some are important for cell migration or act as cytokines (Figure 2B, Table S2). The number of source proteins covered decreased slightly, depending on the storage condition, from 204 to 201 (−20 °C, 120 h), 188 (4 °C, 6 h), 189 (RT, 6 h), and 187 (RT, 24 h). Peptides of eight proteins were only detected after storage but not in the control, increasing the total number of proteins identified to 212. The number of detected peptides varied, but no distinct pattern dependent on the storage conditions was observed. Notably, in three of the four samples, the number of peptides increased after storage at RT for 24 h.

**Figure 3:**
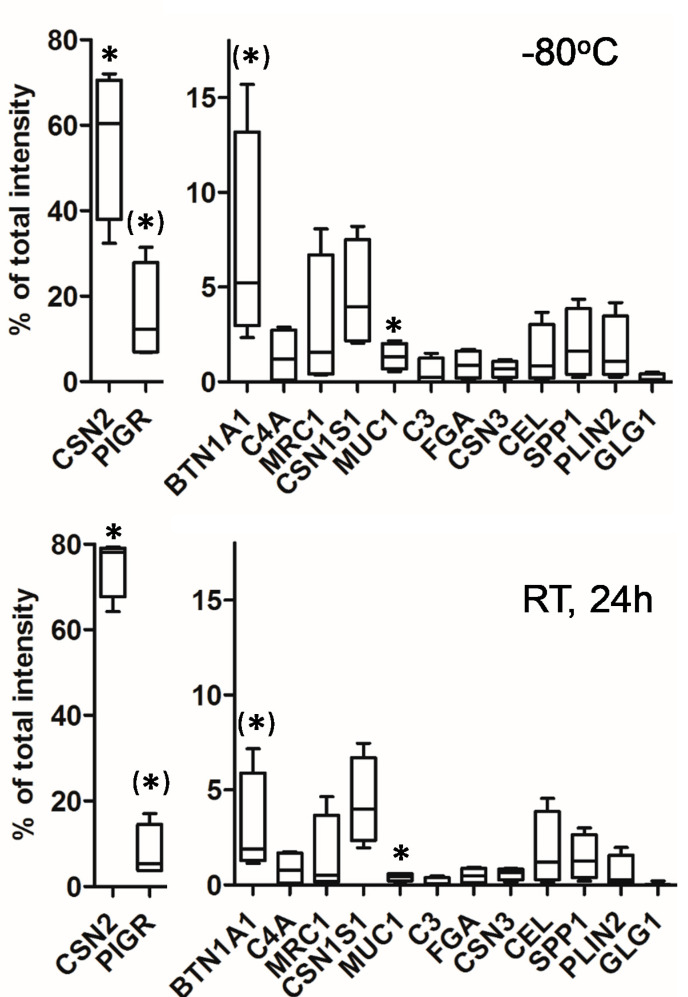
Percentage of the total intensity of 14 proteins representing the 10 most abundant proteins per sample. Boxes display the variation across the milk samples of four different mothers immediately frozen at −80 °C or stored for 24 h at RT. Difference in abundance after storage in the two different conditions was calculated by paired *t*-test. Lines indicate median values. Whiskers are set to minimum and maximum values. * *p* < 0.05; (*) PIGR *p* = 0.06; BTN1A1 *p* = 0.08. CSN2, beta-casein; PIGR, polymeric immunoglobulin receptor; BTN1A1, butyrophilin subfamily 1 member A1; CSN1S1, alpha S1-casein; MRC1, macrophage mannose receptor 1; SPP1, osteopontin; PLIN2, perilipin 2; MUC1, mucin 1; CEL, bile salt-activated lipase; C4A, complement C4-A; FGA, fibrinogen alpha chain; CSN3, kappa-casein; C3, complement C3; GLG1, Golgi apparatus protein 1.

The number of identified and quantified peptides in the samples of the particular participants varied substantially (A-1349, B-2042, C-2200, D-782). As expected, across all samples, peptides of CSN2 displayed the highest number and also the highest abundance. Beta-casein covered between 33–72% of the total peptide intensity (Figure 3). The peptidome was always dominated by only a few proteins. Hence, the 10 proteins with the highest intensity per sample (in sum, 14 different proteins, Figure 3) always represented >90% of the total intensity. Other highly abundant peptides belonged to alpha S1-casein (CSN1S1), polymeric immunoglobulin receptor (PIGR), butyrophilin subfamily 1 member A1 (BTN1A1), or osteopontin (SPP1). Larger differences between samples were obvious for macrophage mannose receptor 1 (MRC1, range 0.4–4.1%) or complement C4-A (C4A, range 0.1–2.2%). The proportion of the peptides of these proteins remained quite stable except for the samples stored at RT for a long time. In this condition, the proportion of ß-casein-derived peptides increased, whereas the relative fraction of PIGR, BTN1A1, and MUC1 peptides decreased substantially (Figure 3).

### 3.2. Impact of Storage Conditions on Human Breast Milk Peptidome Variability

**Figure 4:**
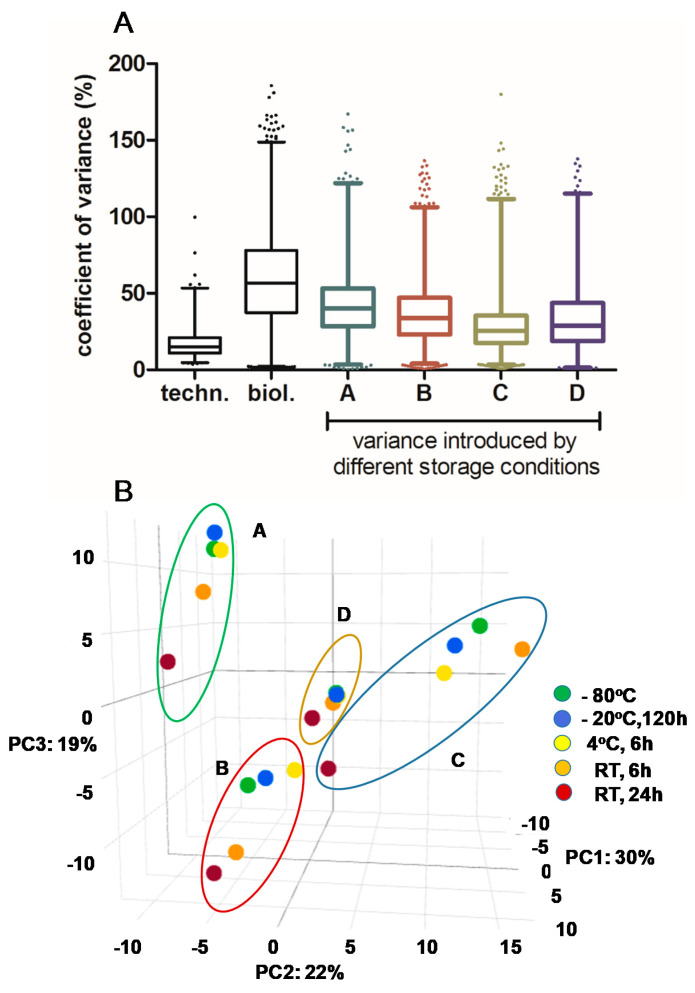
Impact of storage conditions on the whole peptidome. (**A**) Coefficient of variance (CV) in individual breast milk samples A–D due to storage at different conditions in comparison to technical (techn.) and biological (biol.) CVs. Lines indicate median values. Whiskers indicate 1–99 percentile to display outliers. (**B**) Principal component analysis of all peptides per individual sample reveals (i) high variance between the peptide pattern in breast milk from different participants, and (ii) highest effect on the peptide pattern after storage at RT for 24 h.

The applied conditions represent possible storage capabilities during the sampling of breast milk at neonatal units or at home before and during transfer to a unit with deep-freezing capacities. Thus, short-term storage in a refrigerator at 4 °C and at room temperature (RT) immediately after sampling was studied as well as storage in a freezer at −20 °C for five days and compared to the peptide pattern observed for continuous storage at −80 °C. The analysis of the impact of the storage conditions on the peptide intensities revealed inter-individual effects. Thus, the impact was lowest (25.4%) for milk sample C and highest for sample A (40%). In any case, the storage variance was lower than the biological variance (56%), whereas the technical variance for the sample preparation and measurement was determined to be 15.6% (Figure 4A).

An unsupervised multilevel principal component analysis across all quantified peptides confirmed the higher variance between the peptide pattern in breast milk from different participants in comparison to the variance introduced by the storage conditions (Figure 4B). Furthermore, the highest impact on peptide pattern was observed for samples stored at room temperature for 24 h.

**Figure 5:**
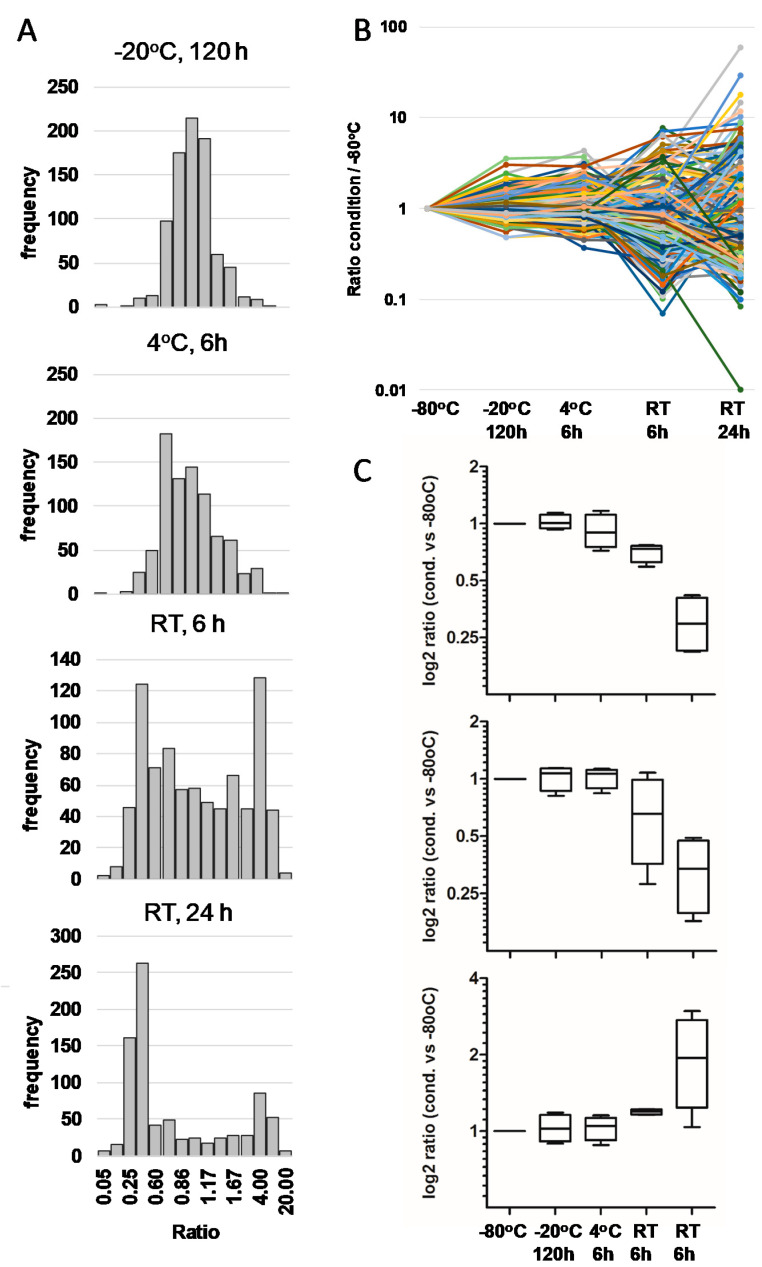
Behavior of breast milk peptides depending on storage conditions in comparison to immediate freezing of samples at −80 °C. (**A**) Histograms of peptide ratios (cond 2–5 vs. cond 1, −80 °C) exemplarily shown for sample A. Ratios close to 1 display minor alteration during storage. High and low ratios point to strong impact of storage temperature. The accumulation of peptides with ratios of approx. 0.25 after storage of samples for 24 h at RT point to degradation of peptides. (**B**) Stability of 255 peptides randomly chosen from 1575 peptides quantified in total in sample A. The majority of peptides kept quite stable when stored at −20 °C or at 4 °C, but showed strong alterations when stored at RT. (**C**) Stability of selected peptides with the same pattern in samples A–D. The pattern displays events pointing to degradation, but also peptides with increasing levels were detected. Strongly decreasing at RT: top: QPSTQIVANAKGAVT (perilipin-2); middle: IPASSLPRLTPWIVA (butyrophilin subfamily1 member A1); strongly increasing at RT: bottom: VLPIPQQVVPYPQRAVPVQA (ß-casein).

Analysis of the effect of the different storage conditions on individual peptides confirmed these findings. Thus, peptide intensities were not affected or less affected when samples were stored at −20 °C for 120 h. However, intensities were altered to a certain degree when stored at 4 °C for 6 h and even more after storage at RT. While both an increase and a decrease in peptide intensities were noted after short-term storage, peptide depletion was the major event in samples stored for 24 h at RT (Figure 5A,B, Table S1). The majority of such peptides disappeared completely, and this depletion was independent of peptide length. Furthermore, a certain number of peptides were only visible after storage at RT in comparison to immediate freezing at −80 °C. In general, the stability of peptides in the samples was highly variable and only a few peptides showed the same behavior across all samples, as shown in Figure 5C.

A closer inspection of peptides detected at all storage conditions with known function as assigned by the milk protein peptide database [26] displayed a rather good stability in comparison to the reference (−80 °C) when samples were frozen at −20 °C. All other conditions introduced a higher variance and increases in or depletions of peptides were observed.

### 3.3. Identification of Cleavage Sites Dependent on Storage Condition

**Figure 6:**
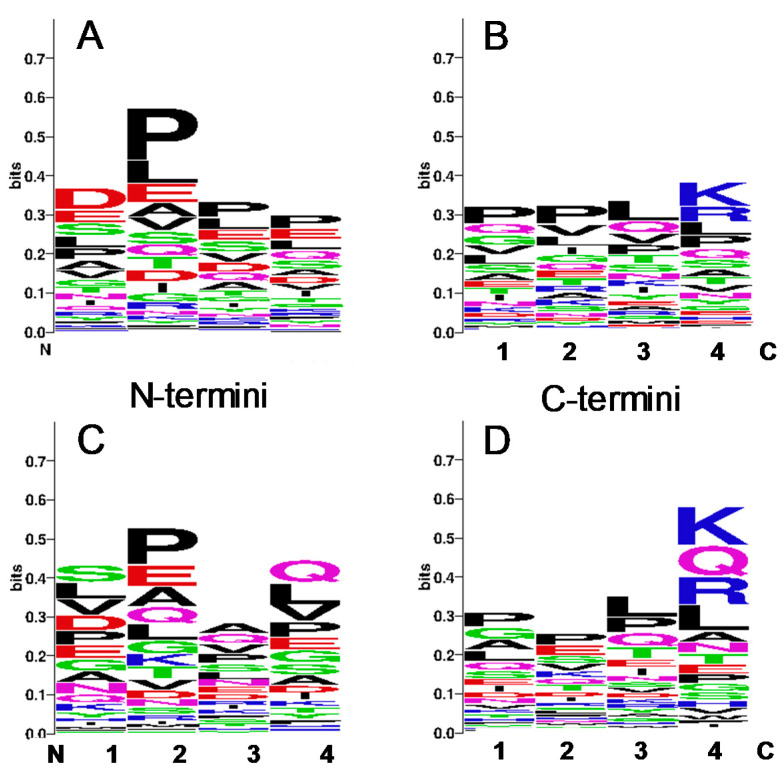
Structure of N- and C-terminal cleavage sites dependent on the storage condition. Amino acid sequences of peptides detected in samples immediately frozen at −80 °C (**A**,**B**) or stored for 24 h at RT (**C**,**D**). Analysis was performed via WebLogo v. 2.8.2. The overall height of the stack indicates the sequence conservation at that position, while the height of symbols within the stack indicates the relative frequency of each amino acid at that position.

Furthermore, we analyzed the structure of the N- and C-termini of peptides identified in immediately frozen samples (*n* = 3235) in comparison to peptides only observed after storage for 24 h at RT (*n* = 164). As shown in Figure 6, slight alterations were found in the amino acid frequency at the C-terminus as well as the N-terminus. At the N-terminus, the acidic glutamic acid and aspartic acid in peptides of cond 1 (Figure 6A) were found less frequently when samples were stored at RT. In contrast, peptides with serine and leucine were identified more frequently (Figure 6C). Lysine (K) and arginine (R) were the most prominent amino acids at the C-termini, followed by leucine and proline in the peptides found in cond 1 (Figure 6B). The frequency of K and R increased even more in peptides found only in cond 5, but glutamate was found in high frequency, too.

## 4. Discussion

More and more studies focus on the comprehensive analysis of the breast milk peptidome in order to better understand the positive effects on the immune system and the development of newborns and to support the configuration of nutrient replacement products. However, to identify peptides that are present naturally and therefore biologically relevant, it is important to understand the impact of pre-analytical conditions such as storage temperature and duration. Therefore, we analyzed the breast milk peptidome in conditions probably applied to breast milk at home, in practices, in hospitals, in milk banks, or during transport of samples collected from research cohorts. Our data clearly show that the stability of the peptidome decreases strongly when samples are stored unfrozen and that the variance increases with the temperature and the storage time. Our results therefore suggest a modification of the very recent European consensus recommendation that allows donors to freeze milk for donation as soon as possible but within a maximum of 24 h [29].

The peptidome is defined by proteases, being already active in the mammary gland [13]. However, these proteases are still active after milk is released and therefore affect the peptide pattern. Reported proteases in breast milk are carboxypeptidase B2, kallikrein, plasmin, elastase, thrombin, and cytosol aminopeptidase [30,31]. Although these enzymes have their temperature optimum at 37 °C, proteolysis of proteins also seems to occur at lower temperatures like RT since we found a certain proportion of peptides with increasing intensity during these conditions compared with those detected when samples were immediately frozen. The alteration of the cleavage site structure of peptides when samples are stored at RT points to an increased activity of proteases cleaving substrates at arginine and lysine, but also glutamate. In breast milk, the proteases plasmin and carboxypeptidase B2 belong to the enzymes with R/K cleavage specificity [27]. However, thrombin might also play a major role in peptide production. This enzyme cleaves at R and K in the presence of proline found especially in the N-termini of peptides identified after storage at RT. Furthermore, thrombin cleavage was also described at arginine/serine motifs [27], fitting to the findings, too. Glutamate/glycine motifs, as found in butyrophilin peptides, are assigned to cathepsin protease or lactopecin 3 [27]. On the other hand, a large number of peptides displayed decreasing amounts or disappeared completely, especially when samples were exposed to RT storage. This is in contrast to reports on protein stability in blood. There, a higher stability has been shown when samples are stored at RT because blood cells are stressed by lower temperatures and additional proteases are released when cells lyse [32]. Such events can also be important in breast milk due to a variety of cells (e.g., progenitor cells, leukocytes) delivered with the biofluid [33]. However, the contribution of additional protein secretion after lactation has not been studied in detail. Therefore, complete degradation by proteases, but also binding to lipids or adsorption to tube walls are assumed to diminish peptide intensities.

Peptide stability in biofluids has also been investigated with regard to the application of bioactive therapeutic peptides. In blood, the stability is highly dependent on the spectrum of active proteases and protease inhibitors highly correlated to the activation level of the coagulation and complement system [34]. The protease activity in breast milk seems to be less variable but varies depending on the lactation period and the gestational age [35]. A search of peptide and truncated peptide sequences against the milk protein peptide database revealed 239 bioactive molecules, all originating from CSN2 and CSN1S1. On average, the behavior of these peptides mirrors the observations for all peptides: intensities are quite stable when samples are frozen immediately but display increasing variation with increasing storage temperature and time.

Like other components of breast milk, the peptidome is influenced by several factors, such as the age of the mother [11], prematurity [36], health status [12,37], and lactation period [25]. Changes in the peptidome seem to have an impact on the well-being of the newborn. It was shown that the level of beta-endorphin in colostrum correlated significantly with pain and psychological involvement during and after delivery in term infants [38]. Furthermore, the peptidome may be part of the protective mechanisms of feeding human milk to prevent NEC in preterm infants because peptides that are highly enriched in preterm milk exosomes may possess protective activities against NEC [12]. However, in premature infants, the situation is more complex because they are typically not provided a single source of protein and they have a lower protein digestion capacity [26]. Human milk is usually supplemented with the nutrients in short supply, particularly with protein and electrolytes, but also lipids, carbohydrates, and vitamins, to meet the high requirements of very low birth weight infants [39]. There are a number of products available for fortifying human milk for preterm babies which differ by the origin of milk used (bovine, human, or donkey), and by nutrient composition. In the clinical setting, feeding preterm infants with a mother’s own milk or HDM reduces the incidence of NEC when compared to formula-fed infants but no differences emerged between infants receiving human or bovine milk-based fortifiers [40]. New knowledge about bioactive peptides could contribute to a targeted functional supplementation of human milk with specific peptides [12].

Bioactive peptides are generated from human milk proteins by proteolysis in the mammary gland and by enzymatic digestion in the gastrointestinal tract. Importantly, some bioactive peptides are generated only after gastric digestion of human milk [41]. Although our results suggest immediate freezing of HDM and mother´s own milk for preterm infants, freezing, duration of storage, and the effect of repeated freeze–thaw cycles may alter this complex digestive process and the release of bioactive peptides. Freezing human milk decreases the energy content and amount and bioactivity of secretory immunoglobulin A, lactoperoxidase, lysozyme, antibacterial factors, and antioxidants. Therefore, a large randomized controlled trial is under way to investigate whether feeding preterm infants with fresh versus frozen mother’s own milk results in a lower morbidity and mortality [42].

## 5. Study Limitations

Finally, potential limitations need to be considered. First, our donors were anonymous. Although all donors were healthy and gave birth to healthy term infants, the biological variance of the samples might be due to slightly different gestational age or duration of lactation, and we cannot relate detailed maternal or infant characteristics to inter-individual differences in certain storage conditions. The influences maternal and infant phenotype have on protein/peptide stability under different storage conditions should be studied in further research. Second, our sample is quite small, as we focused on the measurement of the different conditions rather than inter-individual differences. However, the research that examines the effect of storage conditions on breast milk composition is diverse, different components are always looked at separately and more general studies including all molecular levels are highly demanded for the development of safe clinical protocols [22].

The main strength of the study is the simulation of five highly relevant storage conditions, which occur frequently not only in a scientific context but also in home storage of human milk. Such storage conditions are not only relevant for bioactive peptide content, but also for bacterial contaminants, nutritional quality, and metabolite levels.

## 6. Conclusions

In view of the active proteases and protease inhibitors present in human breast milk, factors such as storage temperature and duration have to be controlled and set in standard operating procedures for the processing of HDM and clinical studies as well. To reduce sample environment-dependent protein degradation/peptide production, the preferred short- and long-term storage condition is freezing since peptides show individual differences in stability and production and increasing variance when samples are stored at 4 °C. Furthermore, immediate freezing of HDM at −20 °C or −80 °C is highly recommended to prevent degradation of bioactive peptides to maintain the positive effects of HDM for the nutrition of infants.

## Supplementary Materials

The following are available online at https://www.mdpi.com/2072-6643/12/9/2733/s1, Table S1: Protein intensity of quantified peptides per sample and storage condition, Table S2: Assignment of quantified peptides to proteins.
