# Supplementary material for: Impact of Storage Conditions on the Breast Milk Peptidome
Source: Nutrients. 2020 Sep 8;12(9):2733. doi: 10.3390/nu12092733 (PMC7551425; doi:10.3390/nu12092733)
Supplement: Supplementary file 1 [file nutrients-12-02733-s001.pdf]

Supplementary Table S1 Protein intensity of quantified peptides per sample and storage condition

Different breast milk samples are named A-D . Average values of technical replicates are shown.

Quantified modified peptide sequence	Protein ID	A_-80oC	A_-20oC_120h	A_4oC_6h	A_RT_6h	A_RT_24h	B_-80oC	B_-20oC_120h	B_4oC_6h	B_RT_6h	B_RT_24h	C_-80oC	C_-20oC_120h	C_4oC_6h	C_RT_6h	C_RT_24h	D_-80oC	D_-20oC_120h	D_4oC_6h	D_RT_6h	D_RT_24h
[V].MPVLKSPTIPFFDPQIP.[K]_	P05814	40234876	45478145	47154697	13886277	47609642	116803492	103079340	61611032	109014158	118300015	44385331	42724472	46561262	40653125	54699041	992752	1141731	957376	1053869	729896
[R].ETIESLSSSEESITEYK.[Q]_	P05814	35249499	26735057	26571614	70460067	43264598	65584927	71045990	55783277	129456510	114468783	30461800	31412007	32584801	44464885	37558686	11445930	9488081	12225357	13943069	12200324
[L].NQELLLNPTHQIYPVTQPLAPVHNPIS.[V]_	P05814	31649920	32979540	33656003	14243572	13439977	11711255	11371560	9499988	9989410	6333593	12982294	12444939	13135299	11065223	7914074	736428	935571	721109	779817	535414
[K].DTVYTKGRVMPVLK.[S]_	P05814	31413543	29450486	29269249	65020094	49516752	46361842	44532286	79978230	76289515	40605523	21938944	25638326	20923606	29482540	30065803	1067582	1294658	1273607	1039561	2409509
[A].VPVQALLLNQELLLNPTHQIYPVTQPLAPVHNPIS.[V]_	P05814	31151555	40457435	47374377	1510142	21170405	23122127	18939144	12466321	18815679	13845887	16795766	12597631	14808798	14487960	13990903					
[K].DDPDAPLQPVTPLQLFEGRRN.[R]_	P0C0L4	28806470	27657890	29384123	22729826	13902724	3135208	3564793	2903747	2719371	1973791	24298932	23704861	24559145	20505913	17515048	181374	190842	161631	203987	298019
[L].NQELLLNPTHQIYPVTQPLAPVHNPISV.[-]_	P05814	24936880	24788324	26027789	8896217	17727965	71801315	73589035	48572943	51900043	35622012	57494722	48278966	58490757	51887890	48972408	2332035	2417473	2189676	2458120	4980829
[L].NPTHQIYPVTQPLAPVHNPIS.[V]_	P05814	23715932	20189952	19035806	12147175	5820829	8853869	8866214	4376520	6519894	4689523	6112853	5922451	6712501	4484166	2792950	902727	1349817	911243	773846	528685
[L].NPTHQIYPVTQPLAPVHNPISV.[-]_	P05814	23424844	22371832	20836988	9920962	11071046	64482697	63114604	55886401	50736023	35119921	44579006	45062177	45363112	40399696	33047055	5839640	7004599	6121410	6423243	6961016
[A].VPVQALLLNQELLLNPTHQIYPVTQPLAPVHNPISV.[-]_	P05814	21697296	29625364	33514338	1376466	16726385	206059770	172379982	190454606	126068840	89103540	88224021	74666665	80289965	76369226	64099233					
[V].DSGSSEEQGGSSRALVSTLVP.[L]_	P01833	20763519	19350877	17928300	23024798	9298676															
[M].DPSKPSSNVAGVVIIV.[I]_	P22897	20328117	20368006	18737390	18561338	6441673	5224062	5234840	4880744	3221809	2028668	4414188	5069160	5311993	4166256	2138754	12112774	10597432	11407034	13975663	7179261
[R].DQADGSRASVDSGSSEEQGGSSRALVSTLVP.[L]_	P01833	19835251	16513521	16928452	18795796	7949947															
[R].DQADGSRASVDSGSSEEQGGSSRALVSTLV.[P]_	P01833	18492919	14637091	12719870	21813176	6585916	634692	620207	900885	483117	351256	419423	423796	388002	331779	139008	103657	91892	106707	76834	62880
[V].LPIPQQVVPYPQRAVPVQA.[L]_	P05814	17623054	17424430	16152286	9887854	4742707	29654445	26814028	28801413	17618586	11726470	23545679	27737151	24721127	20789093	16208081	568635	807104	647714	791240	571833
[I].HWESASLLR.[S]_	P01024	17195389	24330811	27244982	23498178	5307220	11228142	10462148	91132	5098989	2867742	192640	181263	393147	261325	332749					
[A].RETIESLSSSEESITEYK.[Q]_	P05814	16520879	12983650	12377153	15513449	7967481	41356096	37660908	48461810	46427066	15767719	19401705	20246540	18231308	26595816	21541699	4740889	4707912	4886489	6177534	7183034
[E].LLLNPTHQIYPVTQPLAPVHNPISV.[-]_	P05814	14869171	17724388	34816153	14744034	30669345	35368005	36164428	37376795	32456638	31706850	6656168	6332169	11055233	10098801	15580210	10401643	12020826	19029287	20823754	30125901
[K].VLPIPQQVVPYPQR.[A]_	P05814	14497230	13586445	17656933	28264061	19651256	32216642	28653688	38801926	40554704	67636663	12692227	14386296	14895235	10784862	6453036	444774	414807	509189	523736	471951
[R].DQADGSRASVDSGSSEEQGGSSRALVSTLVPLG.[L]_	P01833	14191234	11484503	9797634	2478646	578446	27861502	29404973	48105582	22579970	12887057	26591138	29252938	27077131	23272531	13725749	22607912	15394062	19180306	17340602	12239662
[L].LNQELLLNPTHQIYPVTQPLAPVHNPISV.[-]_	P05814	14169156	15343319	17910679	4523246	17799942	30737227	30437060	25610829	23462605	24610567	15276251	13750755	15363215	15130431	15668928	2013409	2058424	2062169	1960693	2322990
[R].DQADGSRASVDSGSSEEQGGSSRALVSTL.[V]_	P01833	13735451	8552733	8578534	13660752	3798615	122656	91992	132095	71975	41817	56730	46205	46829	31252		24999	26472	30854		
[L].LNQELLLNPTHQIYPVTQPLAPVHNPIS.[V]_	P05814	13314983	14251180	14875145	4199854	7496710	3005028	3318098	1717947	2253091	2783240	2071571	1789286	2136634	1762111	1362586	109402	151226	120888	326413	166645
[R].DQADGSRASVDSGSSEEQGGSSRALVSTLVPL.[G]_	P01833	13145490	12813291	12488665	15341401	7720251						3810432	4071024	4024229	3254264	1910769					
[V].MPVLKSPTIPFFDPQIPK.[L]_	P05814	13034263	12542206	13656391	7408672	34709947	115139700	118070883	104134581	86533887	76370823	1231951	1291001	1129600	1622355	3358303			29928	79168	42334
[K].GRVMPVLKSPTIPFFDPQIPK.[L]_	P05814	12805674	18010409	21992011	3212074	71280693	63997713	65889995	99576717	111545407	157869897	13617701	14482197	15803209	25278961	29532725	84438	188740	105812	127341	1289389
[L].LLNQELLLNPTHQIYPVTQPLAPVHNPISV.[-]_	P05814	12252878	13376576	16564312	1969048	55976458	31435232	31169407	21623167	40599403	82043266	5810561	5382510	6405281	8543984	25022486	663054	744900	611391	822007	2682564
[RK].DPNHFRPAGLPEKY.[-]_	P0DJI9; P0DJI8	11224398	13174293	12779435	8165085	5536100	3649552	3581237	2130085	4446209	2571106	186546	192940	254231	247229	160578					
[K].DTVYTKGRVMPVL.[K]_	P05814	11213220	12861619	14963653	68300841	32656728	17400342	16672447	28722417	32059436	33099334	6665796	9162980	7576498	14880531	20800751	332792	390497	361534	396389	1767157
[P].VQALLLNQELLLNPTHQIYPVTQPLAPVHNPIS.[V]_	P05814	10688673	12229577	12918876	1049801	4751344	3348675	3555403	2637980	2681393	1684054	4963089	4356183	4269126	4296483	4506705					
[I].YPVTQPLAPVHNPISV.[-]_	P05814	10405177	9621784	9777396	9094531	4981763	34711257	34981123	21984566	23734527	18534270	57649622	60009734	63575824	53698055	35704616	3032511	3157611	2698931	3683182	2609957
[K].GRVMPVLK.[S]_	P05814	10256609	8556220	7040294	47182049	20557418	21892279	21099825	41365165	35802385	21193971	11931443	15782771	12481105	16176785	11025708	1533724	1612450	1740255	1953277	3129278
[V].DSGSSEEQGGSSRALVSTLVPLG.[L]_	P01833	9793905	8296264	6721488	1403193	285149	41182951	42850137	44865660	28607029	13403283	27680737	29300738	28868590	24330421	13011337	8365039	6720697	6757189	7126926	4165773
[L].LLNPTHQIYPVTQPLAPVHNPISV.[-]_	P05814	8869683	8905096	10397108	5790373	18674654	26320930	24798312	25899877	22632510	25111443	7989592	8318250	7613949	10336115	20895857	5738004	6819517	8176041	10325568	9956415
[K].AVADTRDQADGSRASVDSGSSEEQGGSSRALVSTLV.[P]_	P01833	8834778	8341340	7458710	6001939	4166368	450575	470318	928385	274200	329398	224738	257314	221051	187605	88783					
[M].DPSKPSSNVAGVVII.[V]_	P22897	8732044	7801920	7650300	10600041	2286408	1038458	1014073	733418	698300	418371	1279622	1294802	1436042	1309674	662251	4157241	3783771	4406187	4857075	2569855
[V].TQPLAPVHNPISV.[-]_	P05814	8451300	8108472	11043395	20669167	4428366	8366458	6934810	4486618	7142189	4610293	4501902	5140790	5260358	8190741	9222893	3947491	3275970	3984160	8599241	12519471
[A].DSGEGDFLAEGGGVR.[G]_	P02671	8353752	6448724	4806311	12413705	1264907	2633766	2011436	5230961	807522	911422	1665340	2190095	1736433	1348238	657660	4803826	3802011	5157464	6791202	2620553
[K].AVADTRDQADGSRASVDSGSSEEQGGSSRALVSTLVP.[L]_	P01833	8079508	8366257	8061401	4203459	3768930	3504948	3567736	6585048	3223570	2312161	1081327	1200646	1224862	906059	525390	75318	69153	69395	65579	42605
[K].SPTIPFFDPQIPK.[L]_	P05814	7909270	8219598	9664118	14586524	15644335	53138804	57083079	38534083	71100419	74277875	5866426	6160392	6383589	5021433	4010015	176726	292344	189416	242289	176879
[K].VLPIPQQVVPYPQRAVPVQA.[L]_	P05814	7831171	7838729	8229157	9375686	23225656	16818283	14929136	17732353	19468982	34390346	10077571	11965487	11407888	11973626	18576249	1634750	1757203	1435131	1996447	1694313
[K].AVADTRDQADGSRASVDSGSSEEQGGSS.[R]_	P01833	7725906	6446840	4906194	18598930	3101711	6755487	10666093	15078495	6081498	3711773	2884022	2999191	3004288	2887496	949517	889347	767516	1053738	463171	436713
[R].DQADGSRASVDSGSSEEQGGSSRA.[L]_	P01833	7701760	5271876	5686327	27944648	2343134	3576098	3887766	7512369	2293137	1071090	2581790	3076835	3282743	3008744	1483789	1909163	1540108	1905686	1302563	1084229
[Q].DPRLFAEEKAVADTR.[D]_	P01833	7318991	6484748	6548689	3872810	5433615	9015533	8753782	6949732	10795324	11581625	4750876	4213975	3973057	3570488	2054437	1065918	1378651	1182854	518886	410953
[S].IPASSLPRLTPWIV.[A]_	Q13410	6911494	7196333	7205322	2365394	1834302	1263305	1466325	1347323	1016308	612518	276176	282010	323909	161606	62584	1481084	1188889	1254156	1582396	1025336
[N].PTHQIYPVTQPLAPVHNPISV.[-]_	P05814	6892565	6813686	7656013	3633537	6759334	14828818	15978227	11584008	13527379	12947039	5747363	5539733	6496877	7779290	9344742	4069825	4894332	4512077	5284325	6259536
[P].VQALLLNQELLLNPTHQIYPVTQPLAPVHNPISV.[-]_	P05814	6713143	7459625	9456457	411713	4141120	27565315	26141449	20436526	19476193	15835749	21056016	18010213	19886409	18149738	18431982	37277	36507	37762	51203	95185
[A].VADTRDQADGSRASVDSGSSEEQGGSSRA.[L]_	P01833	6231303	4736172	5396839	17388115	1556612	4681136	5949471	9910440	2453234	1102862	2543834	3304259	2700018	2391113	1672857	1519101	1269231	1360052	981180	992336
[V].LPVPQPEIMEVPKAKDTVYT.[K]_	P05814	6119046	5197048	5164110	2801109	1889317	8784346	8690892	9637357	5590072	4066265	3860607	3666265	3831937	3102442	1707790	59121	58933	56591		27825
[L].LLNQELLLNPTHQIYPVTQPLAPVHNPIS.[V]_	P05814	6093854	5602028	6308766	669293	8300605	2177044	2224668	909298	1932137	3641853	477408	443128	488614	489312	605997					
[R].ETIESLSSSEESITEYKQKVEK.[V]_	P05814	5831592	4575209	4081951	1635200	3541624	22567290	23644241	30701948	18202099	9688633	18710996	20255334	18665433	17504144	12000308	346082	277508	319106	211352	208693
[S].VPQPKVLPIPQQVVPYP.[Q]_	P05814	5769049	5302633	5663583	3066055	1405185	4382415	4443720	4713557	2556790	1801148	7699840	8494413	8160219	6651575	4263475	588544	686706	587122	639347	352853
[R].DGREQEAEQMPEYRGR.[A]_	Q13410	5764505	4958504	4580927	5217908	1040270	5656250	6720028	6929214	2311695	302458	5497041	5386275	4648211	3871933	2360193	7928087	7292366	7612209	4562994	4818575
[R].DGREQEAEQMPEYR.[G]_	Q13410	5709387	4343985	3462474	7697570	1298753	8265419	10086927	7257958	5927453	2479128	5973528	6213788	5673837	4933159	2209130	14854218	12377704	13557571	10128965	6545375
[K].AVADTRDQADGSRASVDSGSSEEQGGSSRALVSTLVPL.[G]_	P01833	5670863	6518466	5640766	2703223	3590182	3574015	3609831	6574425	2873983	1882703	58209	98159	76182	30499	66718	465231	399334	416023	303507	367037
[R].VMPVLKSPTIPFFDPQIPK.[L]_	P05814	5612975	6869661	9301649	5080404	30158752	12397610	14802333	13650084	30720105	59055093	685170	887307	1214011	2712627	6974347	45200	35238	35166		95828
[V].YTKGRVMPVLK.[S]_	P05814	5524887	5476765	4595879	10057021	11656470	10313036	9065596	13185691	17150430	7809340	3770969	4035705	3559017	5828373	5284728					
[A].LPIIQKLEPQIA.[V]_	Q99541	5439238	5463300	4635561	2185332	1054467	2983733	3152494	1602529	1341166	754528	7168733	7474507	9028055	6676185	3953536	526757	706599	644406	513129	181635
[R].DGREQEAEQMPEY.[R]_	Q13410	5282667	4209748	4193871	10935904	2767269	4263545	5496514	4419498	3783843	2905387	492463	470718	473576	701151	458807	549570	538276	579029	676307	849349
[K].AIQDPRLFAEEKAVADTRDQADGS.[R]_	P01833	5102340	5146403	4593625	2265078	3603390	3000429	3472549	6758730	2777504	3782728	1993725	2003584	1920911	1538848	937555	522167	555684	537348	382729	441371
[P].IPQQVVPYPQRAVPVQA.[L]_	P05814	5026216	4798255	4714806	4362697	1251450	7564467	6763847	5895893	3992642	3034890	6578406	8029777	7167883	5446767	3780284	148716	169196	147331	156223	86188
[V].LPIPQQVVPYPQRAVPVQ.[A]_	P05814	5007125	5829402	6246686	3907292	2073451	3945449	3577824	4325706	2273997	2431551	8643966	9790410	9331058	8197703	11838869	335962	376929	340945	529686	1966097
[S].IPASSLPRLTPWIVA.[V]_	Q13410	4998491	5738575	5431725	1356435	888993	3731117	3772027	3813555	2787018	1806015	2621703	3034500	2988292	1546171	668714	3837103	3076098	3183163	4135312	1613432
[R].DQADGSRASVDSGSSEEQGGSSRALVST.[L]_	P01833	4965628	4738651	3717594	8549577	1716969	2156821	3093303	3574420	1425574	834822	2361019	2260091	2284488	1919489	890671	913425	833268	976025	649335	525426
[L].AQPAVVLPVPQPEIMEVPKAKDTVYT.[K]_	P05814	4879221	5021545	4720492	1993850	2323165	8705680	8397490	12896801	6782641	4171829	5536468	5220055	5348997	4637268	3046918	259794	298122	224526	128547	201761
[A].SVDSGSSEEQGGSSRALVSTLVPL.[G]_	P01833	4827562	3586130	3927625	5344126	2518831											1371994	1200535	1192351	1245708	1017449
[M].DPSKPSSNVAGVVIIVI.[L]_	P22897	4819606	5268194	5868954	2722984	1339963	4428820	4298702	4444664	2523965	1588090	3783794	4166564	4033819	3468395	2299453	11470081	6385629	8160524	12275883	6215439
[Q].IPQRQYLPNSHPPTVV.[R]_	P07498	4799067	4366511	4362639	3529484	2701567	3817865	4289995	3772342	3177664	2524985	1037594	1096395	1075652	887580	645766					
[Y].GFLPQNILPLAQPAVVLPVPQPEIMEVPKAKDTVYT.[K]_	P05814	4717794	5536555	5619347	591063	2907236	17171141	15324240	25814346	12721685	9097360										
[V].DSGSSEEQGGSSRALVSTLV.[P]_	P01833	4681339	3925570	3226492	6890957	2260545	875163	773955	289398	652316	462683	89961	74147	80651	95131	32427	83637	77474	81659	56488	42361
[K].AVADTRDQADGSRASVDSGSSEEQGGSSRALVSTL.[V]_	P01833	4650142	5278419	4587932	4621831	1994707		57453		32587	24429										

Quantified modified peptide sequence	Protein ID	A_-80oC	A_-20oC_120h	A_4oC_6h	A_RT_6h	A_RT_24h	B_-80oC	B_-20oC_120h	B_4oC_6h	B_RT_6h	B_RT_24h	C_-80oC	C_-20oC_120h	C_4oC_6h	C_RT_6h	C_RT_24h	D_-80oC	D_-20oC_120h	D_4oC_6h	D_RT_6h	D_RT_24h
[A].LLLNQELLLNPTHQIYPVTQPLAPVHNPISV.[-]_	P05814	4629295	6368925	11027958	1740558	12875346	11836439	12311616	11013831	14637076	16138438	2602566	2416044	3403224	6217415	9711695	658303	611406	735309	1086021	3404496
[G].FPVSQDQERE.[K]_	Q8NFU4	4404948	3405117	2693438	5675258	358998											309172	412910	350176	370203	230096
[K].AVADTRDQADGSRASVDSGSSEEQGGSSRA.[L]_	P01833	4373450	4069794	4671386	10887578	1360398	2769562	3491116	6368533	1793925	852735	1952946	2382401	1911560	2070297	1337844	1126966	1012615	1157499	731279	757049
[L].NQELLLNPTHQIYPVTQPLAPVHNP.[I]_	P05814	4283437	4326351	3989224	1369084	1142651	841338	855932	589095	606842	287242	433822	418460	496395	395079	194726	85661	108899	83919	96217	49816
[V].LPVPQPEIMEVPK.[A]_	P05814	4267878	3566149	3731153	6476425	5808053	15340409	16684050	9358782	13770285	15289537	5498365	5476929	5927244	7093778	8575648	265977	268237	260330	312646	645135
[S].VPQPKVLPIPQQVVP.[Y]_	P05814	4180113	3546980	3145499	2789303	903779	622566	597575	663835	341379	189401	1210683	1474874	1354795	1090068	615532	342767	421189	367537	355364	226357
[L].NPTHQIYPVTQPLAPVHNP.[I]_	P05814	3987834	3840094	3713996	2409818	1128701	1428329	1665137	1290819	1020718	789951	881124	960018	924226	775361	477975	155881	207319	162765	103118	114300
[E].DEHQDKIYP.[S]_	P05814	3877090	3328043	2657004	12455608	3909306	5123320	5748591	4150984	4036438	2855033	2597938	3326345	3420040	3193504	1490836	2332612	2726573	2298121	1957414	1709072
[V].DSGSSEEQGGSSRALVSTL.[V]_	P01833	3793915	3032808	2569815	5773016	1412592	794461	866375	606197	513964	319955	122834	119162	111384	120693	73257	293147	292579	278753	231591	156704
[K].LTDLENLHLPLPLLQPLMQQVPQPIPQT.[L]_	P05814	3696174	4175500	4030510	529756	4179714	10339691	8108298	6403245	8636901	9744170	1511717	1336883	1620737	1426155	1620218	64212	57922			170198
[R].SPYEKVSAGNGGSSLSY.[T]_	P15941	3626083	3390477	2789380	3351509	746011	2733704	3417198	3709331	1758142	1082064	2518865	2978632	2569150	2260111	1218841	857761	1008227	1063883	844099	275533
[K].EIPLSPMGEDSAPRDADTLH.[S]_	Q13410	3566939	2891901	2180385	2002208	593329	1393119	1701700	1098004	490686	390689	2751187	2881394	3593329	2671041	1205371	455511	481693	486636	197438	107165
[K].SPTIPFFDPQIPKLTDLENLHLPLP.[L]_	P05814	3562878	8302607	8143336	687637	17097960	53543381	57307597	41814957	63087690	88580980	20014481	18447402	22202668	20580455	27208674	330210	262575	221636	190162	784705
[T].VYTKGRVMPVLK.[S]_	P05814	3534818	3672779	3113709	6845708	7933305	6575235	6074965	8472371	11262846	5357573	2468046	2626633	2203484	3735055	3467322	76917	88836	76119	63513	169414
[E].RLQNPSESSEPIPLESREEYMNGMN.[R]_	P47710	3461916	2607568	2677991	1277843	2259783	47208775	53073309	46322254	44596080	39500122	22741273	22658028	23023730	21600177	19690456	2453434	2105553	2669023	1066202	2073574
[V].LPIPQQVVPYPQR.[A]_	P05814	3434004	3602411	4618354	9322736	6818434	5317771	5042682	8910862	11514805	25656286	1077455	1314148	1437015	1069854	581779					
[V].SPAVLVHRDGREQEAEQMPEY.[R]_	Q13410	3426350	3237539	3501361	692919	1560898	6028001	7556777	10378442	7184068	5193352	1299253	1158413	1350011	693427	523560	62656	89873	70136		84648
[R].ETIESLSSSEESITEYKQKVE.[K]_	P05814	3319821	2466548	2370376	1502458	1021491	1865506	1701330	1709312	1344887	899891	3873834	4094277	3890199	2921197	1538896					
[R].YPERLQNPSESSEPIP.[L]_	P47710	3283938	2601942	2667351	2802982	832468	4347135	4838045	3289399	2885934	2005144	1789587	1673781	1868695	1678951	962361	478136	534355	482280	508299	283176
[S].IPASSLPRLTPWIVAVA.[V]_	Q13410	3261772	3613168	4052968	950288	650072	3025476	2715014	2743818	1612481	1013961	2074341	2395604	2140683	966754	244308	1750985	1503537	1508667	1929966	481465
[K].SPTIPFFDPQIPKLTDLENLHLP.[L]_	P05814	3253962	4951047	4290930	355039	1842078	2250595	2150149	1416681	1326136	2593102	1833650	1489465	1859350	1351287	904307					
[S].IPASSLPRLTPWI.[V]_	Q13410	3160200	3026048	2841918	1233388	1150779	501908	610485	812900	577738	252422	502039	571547	546587	381395	162496	1243508	857170	961269	1496946	915608
[K].VLPIPQQVVPYPQRAVPVQAL.[L]_	P05814	3149933	3395437	3609468	2177117	5238312	6287256	6132227	4615751	5405455	9633828	1586876	1585479	1782521	2442993	6191435	77615	99520	81831	157274	660345
[R].RPDIQYPDATDEDITSHMESEELNGAY.[K]_	P10451	3110194	2873013	1729995	859604	3939044	6169638	8056325	13801365	7504340	6637532	705916	714515	648203	505454	495488					
[Q].ELLLNPTHQIYPVTQPLAPVHNPIS.[V]_	P05814	3108005	3074367	3110956	1068159	794940	1194956	1180146	705463	688881	758869	2500942	2524342	2701175	2219697	1479893		66967			
[K].DDPDAPLQPVTPLQLFEGR.[R]_	P0C0L4	3082798	2670123	2818626	5240002	1266392	348970	338837	195623	276588	177583	1645459	1366207	1742614	1399265	1013268					
[V].DLSGHNLANPHPFAVVPSTAT.[V]_	O94985	3036811	2645649	2621033	1751715	559388	1762041	1643131	985054	1061240	790457	114692	99895	148560	123896	42107					
[R].SPYEKVSAGNGGSSLS.[Y]_	P15941	3034109	3079438	2425634	4348914	425452	2127022	2729447	3983995	1100154	565811	1651806	1851031	1867077	1548215	796506	801518	797858	992732	692619	262335
[Y].NPDESSKPNMIDAATLK.[S]_	P02679	3005407	2145715	2043204	2438550	834363	1216808	1517447	1116686	850487	416128	240232	206950	213456	216330	89961	61639	104377	89550	53750	33402
[L].TDLENLHLPLPLLQPLMQQVPQPIPQ.[T]_	P05814	2940235	2557509	2148532	324675	432920															
[T].DRSPYEKVSAGNGGSSLSY.[T]_	P15941	2936986	2945690	2631757	2482083	642085	1957473	2076955	2721391	963730	563185	2256223	2551134	2812028	1946468	1335743	557725	628215	645283	391889	194921
[K].LPLRYPERLQNPSESSEPIP.[L]_	P47710	2834223	2493735	2639705	859567	836251	3951988	4023150	4017437	3120047	2245518	967359	1052445	987159	821713	637730					
[A].QPAVVLPVPQPEIMEVPK.[A]_	P05814	2784863	2778578	2600790	2039033	1083927	15683117	17576094	11968484	10520549	6227248	9017278	7845087	8374114	7661402	5223410	248882	239356	234844	285832	148697
[Y].DAAKRGPGGVWA.[A]_	P0DJI8	2783918	2520007	2273308	3593981	265629	200923	243290	195608	96897	23093										
[A].PLQPVTPLQLFEGRRN.[R]_	P0C0L4	2738111	2420068	2632301	1814552	1303105	201626	236614	213137	180863	121111	2479538	2071469	2170053	2026039	1418585					
[V].LPVPQPEIMEVPKAKDT.[V]_	P05814	2686115	2386188	2209003	1678844	619626	2026705	2099930	1811892	1477616	719161	1302451	1249050	1261497	1004880	631185	121096	171769	133932	82030	74047
[S].KEIPLSPMGEDSAPRDADTLH.[S]_	Q13410	2645251	2282606	1587069	1282370	846379	1669566	1818180	4146056	1630137	795078	7535123	7441571	6807618	5665722	3238438	839202	961999	988541	377369	277475
[G].FPVSQDQEREKR.[S]_	Q8NFU4	2607216	2159020	1943082	4726848	582135	134277	169765	150864	141709	6864	43900	40340	43426	39134	17987	366638	510625	438560	232495	261657
[H].SKLIPTQPSQGAP.[-]_	Q13410	2550699	2134970	1143983	2771617	1231586	669398	901497	3414539	1050045	693156	11105709	11860531	3597293	7106995	4436744	3735862	2547675	2569097	2204750	789874
[G].FPVSQDQEREK.[R]_	Q8NFU4	2519036	1670170	1634069	6846216	526002												16782			
[L].NQELLLNPTHQIYPVTQPLAPVHNPI.[S]_	P05814	2515753	2147162	2053968	532729	17393						271607	236413	265330	229534						
[V].DSGSSEEQGGSSRALVST.[L]_	P01833	2513174	1887077	1552489	5725162	618019	1352831	1748005	1365706	1027562	532146	1511297	1528262	1606080	1401112	453347	875161	963211	911837	705493	308645
[V].MPVLKSPTIP.[F]_	P05814	2486220	2610916	2135439	3167782	4118512	4369264	3903228	3168294	5004390	8445130	4808587	6184575	6359355	6829015	8954691					
[E].IPLSPMGEDSAPR.[D]_	Q13410	2453236	2206778	1708447	3022706	476056	2015395	2078018	1814880	1137534	604983	2865878	3302100	2885590	2652190	1289704	1179711	1129655	1112244	540303	207221
[A].VPVQALLLNQELLLNPTHQIYPVTQPLAPVHNP.[I]_	P05814	2428737	3289940	3905001	388581	1718506	415226	347236		408532		187883	64750	95604	106153	64086					
[L].LNPTHQIYPVTQPLAPVHNPISV.[-]_	P05814	2267357	2488997	2309461	1087868	2778711	12386768	10820610	8401210	6892785	8471455	9958906	9403860	5492661	5973708	10107663	4007516	4470240	4737168	4207969	3807162
[S].TDRSPYEKVSAGNGGSSLSY.[T]_	P15941	2238916	2274447	1595186	1648905	616789	1827578	2002671	4119198	1502804	823389	4128229	4878806	3429841	3247281	1962891	1882729	2049957	1864876	1225329	466665
[D].PDAPLQPVTPLQLFEGRRN.[R]_	P0C0L4	2212900	2058857	2012798	1581708	1086584	162397	260862	206349	163952	121561	1862979	1656315	1666217	1603619	1180946					
[A].QPILSKLEPQIA.[S]_	O60664	2183417	1961192	1543070	1085292	257090	606028	681326	295067	321275	194016	639350	649418	881436	491460	251585	140725	195388	181719	109301	31000
[S].DISNPTAHENYEKNNVMLQW.[-]_	P47710	2150341	2115807	2441468	1769493	1744951	2302777	2171343	1516861	1977402	1283091	456647	418511	517933	467864	610025	172993	190507	200616	119457	451400
[L].TDLENLHLPLPLLQPLMQQVPQPIPQT.[L]_	P05814	2120646	2225348	2121494	396196	660048	3286020	3027360	1558676	2073011	1345060	3668539	3096151	3920137	3001160	2393450			24859		61590
[I].QDPRLFAEEKAVADTRDQADGS.[R]_	P01833	2115818	1956469	1696580	804354	1049476	1122073	1169892	2267623	873347	1015565	685198	710796	667920	504626	296734	128539	121702	122544	77864	107628
[P].LAQPAVVLPVPQPEIMEVPKAKDTVYT.[K]_	P05814	2089571	2223567	2196739	1137839	4658217	12439787	13930588	12491909	9937019	13545541	378218	365285	419096	465163	705274		23145	24225	26113	25620
[R].DGREQEAEQMPEYRG.[R]_	Q13410	2087515	2197699	1835258	3570915	1267675	3601333	4897435	3640115	3484636	1932471	1012451	1002214	1130152	1245074	816273	701789	786842	789208	727975	822959
[R].DADTLHSKLIPTQPSQGAP.[-]_	Q13410	2051594	1840335	1919652	1553549	906933	1963986	2236165	1813705	1181575	1264275	940823	915945	1200807	972473	774354	208853	240412	324429	152484	117479
[K].AVADTRDQADGSRASVDSGSSEEQGGSSRALVST.[L]_	P01833	2050441	2243765	1904001	2489912	873217	1099151	1408971	2283102	671032	336013	1058353	1298258	1068485	914270	566369	441863	402315	376385	246241	223263
[I].PFFDPQIPK.[L]_	P05814	2046679	2978399	3414284	386855	11577897	13233200	14245801	18071318	19791031	24571839	1498908	1681040	1727271	2963590	4738773		18566			123881
[S].EESITEYK.[Q]_	P05814	2025046	1634757	1273609	11592946	1541708	8254919	8118050	3547701	7695352	3867342	7521915	8844679	9604905	9075250	5751212					
[M].DPSKPSSNVAGVVI.[I]_	P22897	2023111	1438904	1298819	2124738	409401	145537	123353	133820	70160	66640	164200	156268	175415	125384	68447	634738	653815	634486	830328	441456
[R].ETIESLSSSEESITEYKQK.[V]_	P05814	2018586	1714931	1447760	2691163	1957255	5345554	5330381	5719387	6095848	4498214	5103861	5349613	5290703	4502781	4226166	715008	628650	679737	526621	619724
[Y].GFLPQNILPLAQPAVVLPVPQPEIMEVPK.[A]_	P05814	2012420	2208102	2214691	499711	660730	9239302	6974161	7448162	5153601	3992492	2623641	2402602	2637117	2220765	1497416	449399	474614	401040	496247	419083
[E].SLSSSEESITEYK.[Q]_	P05814	1998820	1495414	1612881	5603668	2245724	10131714	9840898	5610016	13204944	7616284	5071361	5018357	5181453	8518940	6963825	1051584	956513	1028628	1283238	1184564
[R].IHWESASLLR.[S]_	P01024	1994789	3408427	2819677	1189658	581932	1665915	1469798		745777	431020										
[S].VDSGSSEEQGGSSRALVSTLVPLG.[L]_	P01833	1985156	1899644	1479991	237234	62379	5309154	5477545	5114657	3836212	1683810	3721836	3808259	4083994	3214987	1652901	3865377	3689466	3275611	3755622	2176742
[A].DAAPDEKVLDSGFREIENK.[A]_	P01833	1980691	1943015	1794793	944308	727283	2377378	2429963	2922037	2149671	2236692	986777	888179	921810	730593	476936	298893	363303	314642	221103	182529
[E].IPLSPMGEDSAPRDADTLH.[S]_	Q13410	1979381	1556216	1284006	1265565	413722	646922	789800	921833	350899	274615	2379748	2362372	2453224	2060168	1103926	285802	275293	283604	169401	96352
[L].NPTHQIYPVTQPLAPVH.[N]_	P05814	1976729	1213576	1357199	1019919	760272	1742215	1403256	927308	1055065	902512	1249011	1105720	1367611	1520546	3891201	95856	169671	141221	139929	522988
[R].ASVDSGSSEEQGGSSRALVSTLVPLG.[L]_	P01833	1966624	1656055	1304649	163557	16069	3955054	4138839	5307236	2025168	802377	3535175	3844658	3741426	2927209	1430288	15442203	12507111	12919856	14368616	7834776
[S].TDRSPYEKVSAGNGGSSLSYTNPAVAATSANL.[-]_	P15941	1964271	1933312	1676107	759880	426799	1750444	1707997	2415800	1041478	625223	836812	890784	874358	590820	423025	740791	801193	848415	448188	190569
[Q].PLAPVHNPISV.[-]_	P05814	1928013	2987180	3223675	8321235	5276452	17629752	14023844	15053806	12494670	16051150	4653569	6522638	6666084	12807160	28841436	1254665	1512971	1714255	4730880	16159903
[V].LPIPQQVVPYPQRAVP.[V]_	P05814	1925437	2653789	2794646	2524422	3326406	4590615	4174116	4156725	3425393	6550685	3658959	4314227	3961612	4086588	5866708	303521	321706	268561	364939	298503
[R].DQADGSRASVDSGSSEEQGGSSR.[A]_	P01833	1919627	1344904	1029234	7139615	307231	3140375	3813457	5371691	1940486	416173	719594	925037	1053022	841228	310265	559844	499961	538502	304498	292257
[L].QPLMQQVPQPIPQT.[L]_	P05814	1887258	1762424	1650956	5327291	3444704	3724888	3315804	2061179	3453333	4305485	2032681	1865352	1968621	1973288	2510248	256454	293013	267282	350512	378907
[K].GRVMPVLKSPTIPFFDPQIP.[K]_	P05814	1885259	2942334	3154985	1349998	43512889	9812796	9456049	12763798	21486872	81014661	3959332	3644075	3887166	13409141	33528699	90175	126737	82988		907645

Quantified modified peptide sequence	Protein ID	A_-80oC	A_-20oC_120h	A_4oC_6h	A_RT_6h	A_RT_24h	B_-80oC	B_-20oC_120h	B_4oC_6h	B_RT_6h	B_RT_24h	C_-80oC	C_-20oC_120h	C_4oC_6h	C_RT_6h	C_RT_24h	D_-80oC	D_-20oC_120h	D_4oC_6h	D_RT_6h	D_RT_24h
[I].PLSPMGEDSAPRDADTLH.[S]_	Q13410	1878341	1514347	1157783	1204335	686929	303025	416395	256153	162836	101240	8875138	9504099	9780703	7688366	4967592	195180	206372	201035	108525	124228
[K].LPGQGVHSQGQGPGAN.[F]_	Q92896	1877988	1597512	1530354	3304381	317256	532583	720036	383762	336440	92186	1327745	1484418	1525887	1082358	351330	917282	1080583	975995	801131	332999
[D].ADTLHSKLIPTQPSQGAP.[-]_	Q13410	1875710	1802536	1457053	1016427	440456	1442609	1464192	1532456	770087	399320	1755695	1752671	1799213	1211124	705922	277670	306957	273230	150252	65379
[V].LPIPQQVVPYPQRA.[V]_	P05814	1864513	1474263	1474915	1947440	875029	1683579	1519210	1565400	1405976	2105859	2154837	2560292	2701563	2509196	3705446	176140	185021	186416	245774	285075
[V].LPVPQPEIMEVPKA.[K]_	P05814	1820839	1407170	1608067	2183135	1794538	3148403	3280260	2227086	2404400	3876005	1419574	1283206	1508001	1387274	1665811	61420	68411	62240	64509	77217
[K].GIPFAAPTKALENPQPHPGWQGTL.[K]_	P19835	1799386	2129691	2121706	1016242	5060056	16677592	15704807	18462458	16176734	23357794	703064	746407	758932	832168	1233204		15294			37207
[K].DDPDAPLQPVTPLQLFEG.[R]_	P0C0L4	1740251	1207378	1027901	475915	680606	239644	302155	210802	79889	158171	691987	617903	703759	559503	363581					
[N].PTHQIYPVTQPLAPVHNPIS.[V]_	P05814	1727574	1442007	1414669	690281	555880	242849	179917	139457	182704	284730	288015	301678	329603	243904	215226	45852	74538	52806	51500	64786
[D].PRLFAEEK.[A]_	P01833	1727242	1565880	1556882	3368676	988343	2094308	2364397	3049489	2527171	607729	622440	766417	754786	630404	165270	195149	457812	305624	109615	61351
[R].AVPVQALLLNQELLLNPTHQIYPVTQPLAPVHNP.[I]_	P05814	1721061	2227556	2709439		1607542	483997	406175	96179	154501	203899										
[N].DLEDKNSPFYYDWHS.[L]_	Q14802	1702149	1445489	1292899	849589	309100	153668	200909	127227	113225	41484	23927	25967	28512	22312		280977	227339	225097	199038	87828
[P].VPQPEIMEVPKAKDTVYT.[K]_	P05814	1689658	1501768	1131700	930839	546920	1715323	1770485	1616594	1005771	773951	730763	718134	746069	569510	269982					
[K].DTVYTKGR.[V]_	P05814	1673412	588952	1106205	4516428	791466	164617	143677	571905	373056	61351	25118	73506	110096	35892	44164					
[H].WESASLLR.[S]_	P01024	1638771	1797543	1672611	1753138	263317	509971	561160		265404	125276										
[Y].PVTQPLAPVHNPISV.[-]_	P05814	1630159	1480089	1434478	1174457	1017279	1704000	1621561	1267023	1049450	886405	989805	954916	1118418	994030	1001645	533247	629775	597519	601681	536440
[W].SVPQPKVLPIPQQVVPYPQ.[R]_	P05814	1626722	1609592	1455527	705101	457896	3325345	3303434	2917990	2110696	1248941	280745	339720	304569	295110	296267	50752	83517	55097	81961	41631
[M].KPVPDLVPGNFK.[S]_	P02671	1626548	1877935	2300678	1971391	344447	1593036	1293472	2754966	1205624	130470	196284	199147	317273	148294	75699	118919	120679	120757	109743	51743
[K].DTVYTKGRVMPVLK.[S]_1xOxidation [M10]	P05814	1622516	1772241	2532803	4792864	3551048	4710227	4593440	8448067	6039615	2270224	843046	1138932	1036479	1026646	1662883					
[K].SPTIPFFDPQIPKLTDLENLHLPLPLLQP.[L]_	P05814	1593249	4685055	2853695	239419	8973317	14767151	14782663	17359668	15599446	27484071	6927294	6257835	7220072	6178723	5800975	45329	39813	41692	24814	198124
[K].SPTIPFFDPQIPKLTDLENLHLPLPLLQ.[P]_	P05814	1582936	2929844	1557251	107278	1964875	2166887	1989243	2093053	1744367	3037780	1584626	1351574	1588583	1305093	1191648				38058	119493
[A].VPVQALLLNQELLLNPTHQIYPVTQPLAPVHNPI.[S]_	P05814	1572654	2158716	2306971	115852	1125324	104018	386849	322447	37270	174283	131764	140814	179300	147773	320412					
[F].DPQIPKLTDLENLHLP.[L]_	P05814	1558720	1446217	1459053	189554	690643	1288789	1476280	750137	915057	1003063	3427327	3340063	3321294	3516539	3231297	34592	43482	33936		41996
[I].ESLSSSEESITEYK.[Q]_	P05814	1557337	1120927	1106490	5108410	2775615	5417514	6031413	2384966	8730146	7232069	3188912	2916944	3447643	5160942	5396392	853195	726384	841492	885983	914011
[A].RPKLPLRYPE.[R]_	P47710	1552550	2133149	940194		680580	32271724	29131058	39783561	24505022	15957491	1884808	1708316	1232926	1868178	1130136	20269	230636	23099		19766
[L].LLNPTHQIYPVTQPLAPVHNPIS.[V]_	P05814	1524789	1583094	1585868	761071	2235518	699153	696082	462545	569258	1175470	164365	226188	199237	181733	250582	145604	202962	154519	148573	138711
[A].VVLPVPQPEIMEVPKAKDTVYT.[K]_	P05814	1517745	1621825	1536162	1618772	4898619	3499965	3569972	3606755	3763507	6618642	663609	827080	826289	1029466	2043508					167597
[R].RPDIQYPDATDEDITSHMESEELNGAYK.[A]_	P10451	1517223	1690837	1599394	516955	2607404	6400756	6670861	9590278	7187564	2880588	630644	716987	628063	674582	942149	54197	51796	54872	55826	52065
[K].SSSYSKQFTSSTSYNRGDSTFESKSY.[K]_	P02671	1509321	1537227	2534353	1260429	1813773	772688	861243	255605	1301486	1456763	132674	113438	148950	193261	239244	153529	200484	144094	152018	215778
[M].PVTVTRTTITTTT.[T]_	Q96S97	1472669	1191665	1036278	1162684	282025	490689	513169		298607	175134	1161947	1249708	1129837	859841	579911	273484	257243	289231	166770	57224
[M].EVPKAKDTVYT.[K]_	P05814	1449659	1228894	1008553	3232702	2589887	3352263	3536805	2716948	2902409	3940281	1079380	1200134	1025910	1926091	4065677	582377	725861	557777	509672	917816
[L].PLRYPERLQ.[N]_	P47710	1426620	1896849	1110427		3923127	4067384	3817879	2382023	4267450	2276264										
[N].DLEDKNSPFYYDWHSLQ.[V]_	Q14802	1420830	1252221	1123213	376538	368385	182761	205622	75091	119463	59636	46936	44708	37866	30328		185125	175724	116490	84276	64663
[T].DLENLHLPLPLLQPLMQ.[Q]_	P05814	1416474	20473	68391	73209	234161	2406502	2359772	1210206	2242930	1896033	5218161	4498678	5685262	5024641	4281218	145690	138127	129544	143940	131529
[Y].LPNSHPPTV.[V]_	P07498	1401407	1185801	950405	3510010	780526	557440	696739	400796	518885	466225	1108913	1235656	1193041	1698040	1133168	81899	103231	95401	144515	94247
[K].VLPIPQQVVPYPQRAVPVQALL.[L]_	P05814	1391251	42705	1269014	1712631	4250044	2383371	1974971	2547544	2442129	6753475	1116799	1233763	1360446	1536844	3121266					
[P].DESSKPNMIDAATLK.[S]_	P02679	1386454	1167945	1059315	1678714	454039	472245	563443	422357	342456	215204	26727	26597	27752							
[A].QPAVVLPVPQPEIMEVPKAKDTVYT.[K]_	P05814	1384157	1405145	1353864	685494	909034	3448869	3333882	4664096	3294853	2917764	3734650	3240590	3284822	3043547	2433387					
[V].LPVPQPEIMEVPKAKDTVY.[T]_	P05814	1378127	1369787	1278661	678007	315535	190330	204013	134203	117237	77701	123044	101366	81906	83462	45801					
[P].IPQQVVPYPQRAVPVQ.[A]_	P05814	1369794	1004024	1044279	1104072	381268	570855	556441	520828	332543	333998	903499	1057880	957485	836569	725769	26564	36478	30470	30978	25098
[T].EGGFVEGVNKK.[L]_	P19835	1322933	1018629	872398	8922336	1629075	4081057	4399563	3036868	8155345	3126308	541881	562895	586919	781063	727531		22563	24851		
[F].VEPIPYGFLPQNILPLAQPAVVLPVPQPEIMEVPK.[A]_	P05814	1316370	1497537	1434429	415302	325129	2026650	1722266	1665697	1240889	603453	4799032	4370444	5868351	3947684	2574646					
[V].LPIPQQVVPYPQRAVPVQAL.[L]_	P05814	1281695	1470005	1399588	574138	429673	2393960	2248719	2549514	1813118	1118968	406574	414666	438820	455641	635026					73602
[Q].ELLLNPTHQIYPVTQPLAPVHNPISV.[-]_	P05814	1280291	1359759	1500416	240178	1303973	11803827	10590888	8096738	8060840	5789149	18901128	17225841	18937292	19148841	16592215	182103	191870	182440	172608	282001
[S].KLIPTQPSQGAP.[-]_	Q13410	1279122	1044722	429164	1053309	317635	194759	259268	1776268	409400	242373	2871158	2761480	1947337	1997355	1041227	352061	291645	492475	181718	72737
[K].AIQDPRLFAEEK.[A]_	P01833	1271898	1334945	1043795	1577066	465900	1664038	1527136	3883501	1613173	266776	1740069	2408804	1601387	1504164	513292	1205628	2638510	1742538	818016	567064
[S].TDRSPYEKVSAGNGGSSLS.[Y]_	P15941	1269744	1110984	780333	1229159	266108	815768	1108141	2109266	651741	291600	1664162	1796907	1698076	1320850	741837	709923	815895	770665	495424	180797
[A].DTRDQADGSRASVDSGSSEEQGGSSRA.[L]_	P01833	1265305	1077642	1110916	4013977	442259	484322	523745	923435	330438	165144	864461	1007258	843479	937787	596631	139040	113370	116299	74733	84426
[S].IPASSLPRLTPWIVAVAV.[I]_	Q13410	1255242	1646845	1713614	372664	327586	4174239	3563638	6471856	2236616	1002836	1012406	970573	934422	610949	218718	661748	465074	635365	967071	455092
[S].STYDLMSSAYLSTKDQYPYLK.[S]_	Q99541	1253610	1416789	821011	61448	303222	929011	908555	654250	670243	335193	4312002	4829367	4656018	3380284	2119691	521159	568889	494147	198406	78303
[P].AVVLPVPQPEIMEVPKAKDTVYT.[K]_	P05814	1235781	1229831	1268643	557208	1441131	3082660	3190360	4004551	2592308	4319733	1750010	1816076	1839975	1623121	1157121	19597	22226			28153
[F].PVSQDQEREKR.[S]_	Q8NFU4	1230960	1110699	864779	2124112	271277	48307	52685	55420	46188							172695	241138	211436	104862	116452
[L].AQPAVVLPVPQPEIMEVPKAKDTVY.[T]_	P05814	1218846	1119027	1109234	376617	388543	162385	135301	245033	108835	114357	161853	146381	131178	112592	90453	30746	46027	28390		51101
[Q].NPSESSEPIPLESREEYMNGMN.[R]_	P47710	1211907	975374	765281	843705	421785	8872736	8591218	7113882	6988129	4163124	8898870	8305444	9127118	7448730	3132600	84757	62154	74131	40076	46947
[Q].YVPFPPFSDISNPTAHENYEKNNVMLQW.[-]_	P47710	1207192	1783098	1595559		911095	1455546	1628202	847992	1159394	955325	378539	359345	389341	354223	313095					
[M].PVLKSPTIPFFDPQIPK.[L]_	P05814	1204296	1452089	2022068	593098	7138190	14520679	14436456	11051746	15388352	22223978	120227	107149	223791	602363	1655148					
[S].APRDADTLHSKLIPTQPSQGAP.[-]_	Q13410	1186673	1480246	1139676	273740	479371	858844	818085	1492268	635454	306279	620762	649410	630523	466233	338759	96039	188879	135994	47699	54050
[M].DPAEEDTNVYTEKHSDSLF.[K]_	P34741	1186443	835977	798865	1090369	456039	1507856	1355048	893329	1090712	1153973	185084	216906	210034	145628	104268					
[L].LNPTHQIYPVTQPLAPVHNPIS.[V]_	P05814	1157655	1223569	1064891	459055	394489	265356	249840	181485	186064	330068	211951	195848	249346	176998	160422	115589	169166	130161	111362	100320
[T].DRSPYEKVSAGNGGSSLS.[Y]_	P15941	1155535	1189830	948110	1443725	269972	940752	1105952	1679038	521846	260350	934301	984879	1055887	804937	501411	230465	292618	279631	185246	70878
[N].QELLLNPTHQIYPVTQPLAPVHNPISV.[-]_	P05814	1145217	1066918	1105232		847789	11410941	10504300	5884791	5482951	4497812	26592467	21618028	19964008	19451711	18207596	2341081	2778851	2310462	1418885	1346178
[E].TIESLSSSEESITEYK.[Q]_	P05814	1142912	1139520	1084724	4002010	2367807	5320395	6681082	3646923	9406576	11735850	2626406	2564261	3360419	3922042	5817048	769088	728311	901967	987643	1400684
[A].DQRRDFIDIESK.[F]_	P06858	1139137	962748	1017984	3673330	2681908	1887656	1867607	3094126	5333131	5144663	334659	336352	414818	486640	296081	276572	353562	314996	265475	290940
[A].RETIESLSSSEESITEYKQK.[V]_	P05814	1113840	1265213	917661	647062	1110366	4459505	4224098	5615475	6831148	2052312	1752022	1739913	1640517	3285237	2479770	276596	369475	273212	222330	958630
[A].QPAVVLPVPQPEIMEVPKA.[K]_	P05814	1100960	1124313	962737	799426	478796	3671098	3790517	2480214	2379965	1741352										
[V].LPVPQPEIMEVPKAKDTV.[Y]_	P05814	1099160	876915	934049	537339	204512	539630	529992	639956	287136	180972	511885	478295	481529	415684	214881	20834	23696	18973		14279
[Q].DPRLFAEEKAVADTRDQADGS.[R]_	P01833	1097782	872040	864926	547791	493345	558040	153317	283595	131417	145865										
[R].DADTLHSKLIP.[T]_	Q13410	1086276	816217	646422	903899	176725	200983	277395	302750	225512	131311	1050356	1089332	497011	884818	670186					
[K].GIPFAAPTKALENPQPHPGWQGTLK.[A]_	P19835	1070604	1321167	1450880	281257	1913696	10200792	8603249	10748625	11607297	10771241	1765774	1736678	1640826	2262017	2019542	23083	54660	22766		57352
[V].MPVLKSPTIPFFDPQIP.[K]_1xOxidation [M1]	P05814	1063422	1160590	2341975	709609	905104	3813860	4085760	3030888	1815911	1490797	1063461	1303663	1162256	946193	1450208		75349	42497	154660	48383
[K].VLPIPQQVVPYPQRAVPVQ.[A]_	P05814	1058426	1053286	992136	1013940	2866133	3359700	2834802	2184315	2813168	7239427	4529330	5011633	4224130	6901969	15827570		343211		340740	701570
[A].VADTRDQADGSRASVDSGSSEEQGGSSRALVST.[L]_	P01833	1047360	917937	825663	1429363	346970	334665	455450	793641	212215	118908	542600	534835	442779	477452	264027	252997	207071	217752	156190	138133
[P].QPEIMEVPKAKDTVYT.[K]_	P05814	1046713	829230	723677	642479	215844	591188	643389	544724	369559	234553	487694	474666	487639	386699	205886					
[Q].GPPEEAEDRFSMPLIIT.[I]_	O00592	1042343	1025737	905683	567032	253505	1090961	1249087	953459	774151	455345	399084	377825	430024	381702	180124	67213	57588	64767	49336	41505
[A].LPPQPLWSVPQPKVLPIPQQVVPYPQRA.[V]_	P05814	1030035	1052872	1112173	288278	4321225						4764267	4872385	4626132	3747171	4464447					

Quantified modified peptide sequence	Protein ID	A_-80oC	A_-20oC_120h	A_4oC_6h	A_RT_6h	A_RT_24h	B_-80oC	B_-20oC_120h	B_4oC_6h	B_RT_6h	B_RT_24h	C_-80oC	C_-20oC_120h	C_4oC_6h	C_RT_6h	C_RT_24h	D_-80oC	D_-20oC_120h	D_4oC_6h	D_RT_6h	D_RT_24h
[I].QDPRLFAEEKAVADTR.[D]_	P01833	1020929	910353	1001773	632666	2499975	2213108	2074370	3254330	5453949	3948121	385497	401097	355245	590349	441267	822069	1108698	845399	524672	383236
[L].TDLENLHLPLPLLQPLMQQVPQPIPQTLALPPQP.[L]_	P05814	1019481	1154820	996235	259958	257415	945491	1047504	842405	608743	324244	2975973	2542265	3220255	2223034	1451465					
[R].AVPVQALLLNQELLLNPTHQIYPVTQPLAPVHNPI.[S]_	P05814	1014708	2107656	668151		1721330	404384	592271	632076	476978	550830	155338	108496	96915	158445	196514					
[W].SVPQPKVLPIPQQVVPYP.[Q]_	P05814	1011607	1054096	1227720	753660	737028	964802	940445	1191049	623897	705903	865418	966039	968803	821196	780113	525537	534866	521756	859694	385173
[G].RVMPVLK.[S]_	P05814	1011215	906315	640861	3241069	1770115	1177320	898079	940897	2144851	785362	610304	793721	631483	751789	175636					
[R].DTGAALLAESRAEKNRL.[W]_	O00391	1010347	696935	711621	256778	276121	472893	480590	711582	310667	140322	218531	211825	196314	134008	81748	200763	234138	248584	130694	111602
[L].NQELLLNPTHQIYPVTQPLAPVH.[N]_	P05814	1007143	814354	917315	378868	589173	1067681	1034129	458166	779669	877582	2547341	2458781	2837857	2708901	3920528		64956	30226		610712
[P].QPKVLPIPQQVVPYP.[Q]_	P05814	1004197	1010963	952984	634096	255470	901282	837728	880457	489407	241588	1118127	1283794	1265419	964402	515191	54147	54349	46687		28132
[L].NEYNQLQLQAAHAQEQIR.[R]_	P47710	1004005	974958	1621005	2740561	4305452	1095722	1061459	1343557	2691674	9216565	309133	327290	553341	993743	1604688	21136	26514	17301		41133
[R].YPERLQNPSESSEPIPLESREEYMNGMN.[R]_	P47710	996774	787719	755461	435652	381186	3418037	3445163	3624917	2909833	2396672	3836094	3781322	3806541	3297792	1787592	117645	41711	16157		6721
[K].SSSYSKQFTSSTSYNRGDSTFESK.[S]_	P02671	992064	944482	1210719	740811	315356	810323	838920	389888	910744	509724	201765	238354	263890	270282	179902	255823	310598	250612	236957	180766
[-].MEAARDYAGALIRPLTFMGSQ.[T]_1xAcetyl [N-Term]	O60543	991852	1088900	989675	169258	302605	808000	758403	563346	475408	337029	2295172	2171894	2482165	1873489	1335821	414480	475321	445611	377230	169826
[L].WSVPQPKVLPIPQQVVPYPQR.[A]_	P05814	983081	1071128	850491		1949620	1524259	1570608	1150227	1607597	2501207	2600413	2920341	2594746	1994758	1635326	437541	511886	386851	337509	275205
[P].DIQYPDATDEDITSHMESEELNGAYK.[A]_	P10451	968576	567848	685366	396955	567799	2716252	2923755	3478273	2140884	977349	1137494	1216570	1083783	932744	745776					
[N].DLEDKNSPF.[Y]_	Q14802	961389	729793	507584	704165	20399															
[P].VPQPEIMEVPKAKDT.[V]_	P05814	956835	670817	575521	680957	217907	568962	556736	450606	329676	191203	246231	216852	230548	184963	73651					
[Q].ALLLNQELLLNPTHQIYPVTQPLAPVHNPISV.[-]_	P05814	955963				1583092	11463866	9047042	6473350	5012867	6832227	22225438	18146097	11996408	12180845	20050274		1389095	1334657	29116	1700408
[I].QDPRLFAEEK.[A]_	P01833	955159	951849	713232	1374673	344592	1181331	1314330	3174371	1469440	136467	1051751	1380012	1043776	780878	265482	466377	1087257	713552	324185	243143
[Y].GFLPQNILPLAQPAVVLPVPQPEIMEVPKAKDTVY.[T]_	P05814	949181	1362919	1200033		488356	208231	198424	280843	145037	114709	68501	35332	22259	31548	31043					
[L].LNQELLLNPTHQIYPVTQPLAPVHNP.[I]_	P05814	929538	1045995	1038823	275180	383877	123591	133881	47993	85688	103157	35145	53071	25407	35684	41669					
[R].DLPAEPAAARGGGGGGGGGGGGRGPQA.[R]_	Q86Y38	927345	916145	873598	1530944	249733	362041	401937	617792	238616	122344	138918	124553	90115	118056	75499	123019	139473	136436	133767	92639
[S].STDRSPYEKVSAGNGGSSLSY.[T]_	P15941	926658	1040917	775725	556667	156942	461277	1120933	1672738	592202	123715	1942953	2155473	1559863	1494233	1085779	570164	698421	608715	469845	177739
[R].RPDIQYPDATD.[E]_	P10451	924747	514421	432529	803177	299984	13629888	11473094	3203045	10504613	6038451	915689	703267	787244	1032713	803861					
[K].AIQDPRLFAEEKAVADTRDQADGSR.[A]_	P01833	923175	1289496	1142646	191404	527765	2351731	2231305	4565320	2397817	2071276	729587	777427	699032	593128	456786	358626	443881	302631	97334	414371
[T].SSTSYNRGDSTFESK.[S]_	P02671	919825	1003257	643332	646701	422373	592387	728492	2446319	474088	357936										
[P].THQIYPVTQPLAPVHNPISV.[-]_	P05814	910543	868495	1046271	482083	714338	2776068	2746638	1516718	2380087	1734634	1381160	1304062	1348709	1466077	1713153	451613	523949	400500	544049	466670
[P].LLQPLMQQVPQPIPQT.[L]_	P05814	907477	987640	1245399	977680	1181894	1066185	1116411	1060423	1033235	1401971	304983	373188	581533	733921	790335	127940	155535	237990	411844	590987
[V].YTEGGFVEGVNKK.[L]_	P19835	900057	855652	1034842	2517439	738918	3039700	3500699	1805058	6040666	2626486	127497	110945	215305	664722	304132					
[R].DQADGSRASVDSGSSEEQGGSS.[R]_	P01833	897838	451066	267028	3210968	309200	427144	710815	693569	354040	156851	170823	188913	191908	163557	138088	62233	51699	84828		37667
[F].DPQIPKLTDLENLH.[L]_	P05814	879151	716381	730731	256725	253842	444210	420590	321143	289933	164724	2324001	2306779	2315341	1904623	1406872	18460	17179			12872
[K].VLPIPQQVVPYP.[Q]_	P05814	878365	830142	805109	976752	490019	3899350	3898587	1815043	2277470	1813080	996130	1103338	1149432	1065859	739270	30006	54847	23422	37213	27994
[D].PRLFAEEKAVADTR.[D]_	P01833	871814	696222	726339	423239	859045	1182059	1118859	1199119	1656792	1634013	545090	506004	429661	518003	245815	297321	425569	324826	132529	110808
[-].MEAARDYAGALIRP.[L]_1xAcetyl [N-Term]	O60543	871468	810646	643990	356081	228798	291336	295281	235300	172628	82815	916828	933401	504889	802199	556254	381363	216543	145147	210056	78693
[R].SPYEKVSAGNGGSSL.[S]_	P15941	868356	784104	665446	1102266	220399	845945	1008085	514952	364043	207444	402435	447474	573218	446931	266791	171768	306041	311147	185421	94084
[N].EYNQLQLQAAHAQEQI.[R]_	P47710	867662	886262	761547	676485	155490	184696	165468	137619	548889	929738				10788	16614					
[P].LWSVPQPKVLPIPQQVVPYPQR.[A]_	P05814	863441	1014122	1234380	1342999	14968116	3795608	4026549	2777411	6890494	15376884	729864	716997	813460	1268788	3037337	157429	136801	129397		236822
[K].AIQLTYNPDESSKPNMIDAATLK.[S]_	P10451	853727	789798	920557	827792	702917	677889	622777	778643	1012936	621284	56674	101336	61361	32194	47998	51286	68550	62126	34099	55890
[Y].GFLPQNILPLAQPAVVLPVPQPEIMEVPKA.[K]_	P05814	853107	858531	966576		261252	2550249	2011004	2007695	1380576	1086848	557525	512909	596194	490628	389408	89849	99120	52216	55753	87419
[L].PIPQQVVPYPQR.[A]_	P05814	842833	829010	1049230	1631684	1457060	2433652	2369946	2829629	2914155	5224130	848616	1034214	1036310	818673	464085		19914	21438	15417	16650
[L].SSSEESITEYKQKVEK.[V]_	P05808	829181	926773	769762	253127	1788508	5141053	5068046	6717967	8359485	4290755	4531152	4322580	3612758	4144493	4157980	36452	53137	26558		193113
[L].ALPPQPLWSVPQPKVLPIPQQVVPYPQ.[R]_	P05814	826525	979709	1020657	73025	1075619	2408505	2266079	1714036	1255730	1143723										
[L].DAPPPPAAPLPRWSGPIGVS.[W]_	Q99523	820650	698484	592462	556957	167910	245140	236764	232696	2133769	2771885	419593	354076	465994	321443	152361	399195	408148	370736	343757	174980
[L].AQPAVVLPVPQPEIMEVPKAKDT.[V]_	P05814	808536	755577	713796	424532							509601	456897	497258	446428	45578					
[K].DTVYTKGRVMP.[V]_	P05814	806735	867484	819626	6366389	3190367	1600753	2003336	1047639	2785712	2034923	527410	620322	460620	1003985	1143664		21454			44721
[D].SGSSEEQGGSSRALVSTLV.[P]_	P01833	806572	575560	592454	472115	320679	344168	263991	167510	167919	133831	78108	67105	90018	84781	49099					
[D].TVYTKGRVMPVLK.[S]_	P05814	800976	762887	655401	1066068	1584314	2514764	2284699	2271739	3753568	1606184	531017	599761	538582	820757	798717					
[R].LQNPSESSEPIPLESREEYMNGMN.[R]_	P47710	796599	704397	591274	582197	764507	7008769	7148216	6821268	7840555	8092312	3483697	3290758	3629299	3133161	1702058	118262	91767	104301		60638
[A].IQDPRLFAEEKAVADTRDQADGS.[R]_	P01833	787087	641389	647333	262444	398089	413447	470831	837065	367921	293890	104396	38387	60906	37311	34422					
[E].VPKAKDTVYT.[K]_	P05814	782298	496176	348860	1592780	238182	561495	516868	681570	390843	208250	363752	534098	426283	383050	303332	205606	190060	208853	104865	98376
[Y].TKGRVMPVLK.[S]_	P05814	780411	702987	685949	1622455	2123100	1707481	1439920	2261827	3164816	1389193	762205	871431	761737	1146363	1089583					
[A].PSGQGERVVNVPL.[C]_	A6ND01	772832	697150	721688	721542	216297											454674	352929	391668	450459	237498
[N].ILPLAQPAVVLPVPQPEIMEVPK.[A]_	P05814	766059	550930	832402	779421	383676	2059036	1985250	2104282	2427090	2737344	407014	988974	480721	453932	783005					
[L].GAVYTEGGFVEGVNKK.[L]_	P19835	765149	691789	852967	1471975	781455	2469155	3320515	3335030	6734222	3378265	275129	282708	319092	884102	577420	23614	32065	24940		33118
[A].VADTRDQADGSRASVDSGSSEEQGGSS.[R]_	P01833	759906	564173	463180	2263978	234793	651488	964296	1113759	471335	224665	232812	246463	254651	238492	96932					
[P].LLQPLMQQVPQPIPQTLALPPQPLWSVPQ.[K]_	P05814	757642	802259	929811	513212	2701668	9048477	8016783	6464469	5933546	7734677	1553639	1304947	1838441	3023399	5802042		10879			68907
[Q].IPQRQYLPNSHPPTVVR.[R]_	P07498	756727	875618	717118	136517	279022	720033	590798	775259	375971	128047	493721	538649	560911	429666	317225	20983	41228	34016		21397
[G].DFLAEGGGVR.[G]_	P02671	756643	535078	569145	838150	72869	200224	236216	93393	93065	24981						59096	57530	62216	37809	15695
[Y].TEGGFVEGVNKK.[L]_	P19835	756578	690043	749478	4871259	804487	2637446	2784162	1625326	5333273	2369557	134895	145156	128549	790555	523197		15745	17316		
[T].EGGFVEGVNK.[K]_	P19835	755462	741108	854713	9118101	1615201	1808251	1821196	1295941	4333437	3378922	267349	298317	345316	737781	548708	98318	123700	98126	100977	110308
[S].GSSEEQGGSSRALVSTLV.[P]_	P01833	754351	551882	508339	559135	343629															
[V].DSGSSEEQGGSSRALV.[S]_	P01833	748184	589991	598496	3164126	377846	774669	861497	714257	621339	199405	372686	411528	435351	576385	234280	904557	1100724	1051097	730386	642679
[V].ADTRDQADGSRASVDSGSSEEQGGSSRA.[L]_	P01833	730876	718524	583000	1819186	230777	279730	437089	788143	214379	92472	397627	462570	405924	374314	181228	126460	142922	170711		46409
[R].EKQTDEIKDTR.[N]_	P47710	719831	518446	449737	4269149	483319	280117	266288	232514	806120	75556	232880	359924	392705	501548	177551					
[K].EIPLSPMGEDSAPR.[D]_	Q13410	712189	520886	460375	723924	110003	355807	395095	288484	246645	147062	393930	382630	586500	411774	180673	150530	154193	215449	73790	28706
[Q].IPQRQYLPNSHPPTV.[V]_	P07498	709319	798182	761060	533456	301973	468923	534540	414522	326801	159630	428220	471554	492965	385759	272900	46026	52930	39364	40335	33920
[S].PMGEDSAPRDADTLH.[S]_	Q13410	707185	338719	362986	359034	161614	370432	331407	416714	346623	141672	1120932	1111641	1074154	821786	430825	62680	97350	98264		43609
[E].LLLNPTHQIYPVTQPLAPVHNPIS.[V]_	P05814	701868	710091	969124	614228	4483089	562344	532324	368414	783712	1765603	247282	215684	251315	360538	471945	33666	12410	48602	94389	341983
[I].PQRQYLPNSHPPTVVR.[R]_	P07498	698830	765542	655124	95016	290501	599028	540878	585729	416840	109972	494653	527123	450222	392370	302275	42586	60153	36231	35102	32306
[A].RETIESLSSSEESITEY.[K]_	P05814	694826	475226	424164	543679	269548	1570165	1816775	1643387	1731642	1055619	191864	271979	221625	272895	337705	112935	102198	111691	174435	200823
[A].NPAVVRPHAQIPQRQYLP.[N]_	P07498	692618	869905	771146	119069	416616	387779	331079	531363	311413	186600	946988	1056647	857974	396441	667818					
[P].QPKVLPIPQQVVP.[Y]_	P05814	688890	601132	554946	558207	133343	121471	129415	131487	108486		219192	239027	212951	187381	113741	19610	23064			
[R].EIENKAIQDPRLFAEEK.[A]_	P01833	685750	562935	618673	351666	145616	372935	364698	572658	264255		442731	470833	499232	337305	230294	646669	656779	657998	436448	347797
[Q].IPQRQYLPNSHPPT.[V]_	P07498	683013	590525	562334	487786	182579	231661	282117	159558	155613	98809	31670	29063	26670	20385	18124					17291
[L].NPTHQIYPVTQ.[P]_	P05814	682875	528538	520040	1324136	1092604	5662732	4891802	1964658	3551762	3328894	3323484	3434934	3649944	4130725	6916627	187797	254015	205469	342116	1139331

Quantified modified peptide sequence	Protein ID	A_-80oC	A_-20oC_120h	A_4oC_6h	A_RT_6h	A_RT_24h	B_-80oC	B_-20oC_120h	B_4oC_6h	B_RT_6h	B_RT_24h	C_-80oC	C_-20oC_120h	C_4oC_6h	C_RT_6h	C_RT_24h	D_-80oC	D_-20oC_120h	D_4oC_6h	D_RT_6h	D_RT_24h
[S].EEQGGSSRALVSTL.[V]_	P01833	682447	513487	469144	997930	196278						82521	112465	64978	110257	37192	24618	33196	28461	36772	24542
[R].VMPVLKSPTIPFFDPQIP.[K]_	P05814	677462	839745	1162149	245265	2792517	883540	941183	890629	1660547	5578400	328853	390105	494498	1475537	2965293				24859	39512
[D].TVYTKGRVMPVL.[K]_	P05814	677356	783926	552551	1578629	3307714	890384	738808	1455066	2536738	2444643	783821	915263	861238	1860783	2054895	59478	39707	46138	31338	109393
[K].DSHSLTTNIMEILRGDFSSANN.[R]_	P02671	676084	737512	709358		1474391	493754	483260	416835	674539	758848										
[K].AVADTRDQADGSR.[A]_	P01833	675322	726892	645713	105699	292470	1018834	1054923	2360218	884364	890568	471127	435899	422224	308729	191568	250293	407884	284544	70010	197973
[P].VPQPEIMEVPK.[A]_	P05814	669714	409424	370452	701949	396379	2193543	2393740	1414591	1356682	1323270	654755	621533	677151	577158	550603	16954	23213	15853	28632	22904
[A].VADTRDQADGSRASVDSGSSEEQGGSSRALVSTLV.[P]_	P01833	667974	723941	554404	378184	238003															
[L].WSVPQPKVLPIPQQVVPYPQRAVPVQA.[L]_	P05814	667422	905744	784177	142254	293432	1286302	1410875	1324834	724409	393650	706188	655303	350080	492074		534604	511339	476143	416142	319086
[E].DQQQGEDEHQDKIYPSFQPQP.[L]_	P05814	667248	472494	429229	624029	204381	516202	612411	438278	407460	295373	806025	746611	794883	571940	259187					
[R].DADTLHSKL.[I]_	Q13410	660932	655298	544398	925583	57424	200424	232954	164699		18317	273046	291680	523730	360507	174402	46422	101782	147191	39497	25678
[V].EISIPASSLPRLTPWIVA.[V]_	Q13410	660903	651314	740223	111176	130425	2951581	2468948	2006497	1818478	960338	429290	407643	441237	363604	251857	1850787	1912825	1661652	1729188	806014
[K].DTVYTKGRV.[M]_	P05814	650944	441372	318795	5326784	1557528	1828206	2006600	1001549	2756689	1007675	590544	801224	474761	940179	998083	22606	36703	22119		51191
[T].IPFFDPQIPKLTDLENLHLP.[L]_	P05814	647417	967675	844557		290389	1007830	1044379	318992	579084	535709	625463	501914	617581	853357	765011					
[K].AIQDPRLFAEEKAVADTR.[D]_	P01833	639638	820899	875029	388469	1337135	2095315	1845643	5191959	3683791	1893107	735244	1005530	831005	976954	558981	1031282	1430785	1143755	634255	565419
[R].AAPGQEPPEHMAELQRNEQEQPLGQWHLS.[K]_	O00391	636566	724439	646710		298298	1026840	916918	880398	970166	703259	396311	390633	410409	369442	284159	291909	455006	348864	128889	383696
[L].LNQELLLNPTHQIYPVTQPLAPVHNPI.[S]_	P05814	635992	665582	722945		310799	254601	186585	649526	111218	142899										
[R].DRLDEVKEQVAEVR.[A]_	P02649	635101	493870	508189	277726	275008	1643454	1568610	1956315	1278795	625089	56268	33278		41831						
[T].DLENLHLPLPLLQPLMQQVPQPIP.[Q]_	P05814	633826	740463	540811		205106						1573489	1345066	1656922	1228817	777510					
[R].DTGAALLAESRAEKNRLWGPLEV.[R]_	O00391	628426	956699	808292		826016	677050	699364	913357	574923	359864	109428	122161	110676	126330	97535					
[K].SPTIPFFDPQIPKLT.[D]_	P05814	626136	601273	636551	245005	232486	1036935	952768	485254	596331	505922	1228821	1172014	1226234	1153168	982301					
[F].PVSQDQEREK.[R]_	Q8NFU4	625451	439933	398150	1549829	130524															
[R].SFVDLSGHNLANPHPFAVVPSTAT.[V]_	O94985	623019	705635	625454		109749	465712	480143	208873	216463	120502						27039	48178	31814		19907
[T].IESLSSSEESITEYK.[Q]_	P05814	619049	591488	537039	2328486	1035226	3172072	3291723	7340484	4888657	4448642	915946	911642	1121446	1403673	1618890	352237	328486	360131	405360	388313
[I].PLSPMGEDSAPRD.[A]_	Q13410	618516	441099	341545	637872	74998	278556	342361	300095	166019	99240	953097	932726	980281	661750	302846	241404	239091	249543	107623	33155
[Q].DPRLFAEEKAVAD.[T]_	P01833	618318	487681	421684	425928	324729	2346126	2638121	1802316	2387889	3614375	142455	138301	158905	118525	68681	56042	66804	63016	41956	24617
[E].APSLRPAPPPISGGGY.[R]_	P02675	612383	443943	511621	587491	124331	508942	522208	438606	280028	193594										
[S].VPQPKVLPIPQQ.[V]_	P05814	608468	584238	516803	589095	222353	301951	271697	243259	169659	265370	242956	338732	277895	299401	700990	60683	76093	60306	64232	70429
[F].DPQIPKLTDLENLHLPLP.[L]_	P05814	607292	659078	637067	115732	686094	7877783	7181059	5255523	6321197	5341398	16914748	14562924	15736023	15391112	15277049	93168	120331	90502	100424	117016
[A].EQMPEYRGR.[A]_	Q13410	607154	480888	381584	324812	85064	495338	671183	647874	220128	34029	614876	519414	446302	416229	129633	737578	806188	832454	464463	390085
[S].GSSEEQGGSSRALVSTL.[V]_	P01833	592866	424299	314962	424056	175817	53818			60007	54550		27055		27694		107169	96019	92220	89563	52866
[D].DPDAPLQPVTPLQLFEGRRN.[R]_	P0C0L4	585766	494011	565457	202368	73981	176268	161291		95087	78232	1894908	1909887	1883868	1677404	1350667					
[L].FAEEKAVADTRDQADGSRASVDSGSSEEQGGSS.[R]_	P01833	583212	598986	394928	592249	315114	444531	497762	1482526	392737	285234	308593	262399	239541	230982	127123					
[R].DQADGSRASVDSGSSEEQGGSSRALVSTLVPLGLV.[L]_	P01833	579612	579868	583600	208349	93295	342887	434138	361760	63229											
[L].PVPQPEIMEVPKAKDTVYT.[K]_	P05814	578901	590631	613503	90192	1370628	2430517	2535809	10463443	2432206	3035028	688959	697159	739411	704416	655408					
[S].STDRSPYEKVSAGNGGSSLSYTNPAVAATSANL.[-]_	P15941	576200	476547	369415	92322	61697	439938	442407	573085	295296	126185	278489	328243	431654	176614	131786	217094	403496	265590		
[A].EEKAVADTRDQADGSR.[A]_	P01833	573188	371150	329047	587233	35237	545987	698978	630192	272018	96739	211841	202059	236661	188713	39417	556384	635499	503605	303763	258692
[T].DLENLHLPLPLLQPLMQQVPQPIPQTLALPPQP.[L]_	P05814	569331	610814	494009		190093	667346	719220	380132	355767	194311	2160999	2022676	2204714	1725510	991486					
[A].DGSRASVDSGSSEEQGGSSRA.[L]_	P01833	569193	424963	382557	2466853	190416	351428	479372	586077	277926	84953	743375	866967	963682	707710	433013	172817	127860	144918	89538	114459
[L].NPTHQIYPVTQPLAPVHNPI.[S]_	P05814	563698	505163	413396	241788	122916	163668	98539		97242								29905		33378	9648
[R].RPDIQYPDAT.[D]_	P10451	561907	439103	338583	1449364	558416	2515919	3264159	1667087	3006749	2958513										
[M].DPSKPSSNVAGVVIIVIL.[L]_	P22897	560657	646466	734251	415172	102657	322088	261144	681605	151958	57739	343331	468219	369397	242767	174910	450495	325855	583158	474165	337274
[S].STDRSPYEKVSAGNGGSSLS.[Y]_	P15941	556649	409477	275711	319645	70094	366723	483006	605779	233027	90148	773811	843811	730677	523853	318871	301048	320826	323212	173740	77412
[W].SVPQPKVLPIPQQVVPYPQRA.[V]_	P05814	556243	577775	539206	252656	121421	212745	178547	153384	79055	59426	230567	267069	278892	262048	259612	102305	152517	126292	84605	74177
[Q].YVPFPPFSDISNPTAHENYEKNNVMLQ.[W]_	P47710	554906	551857	478867	174752	223239	48759	54016		60000	32078										
[V].EISIPASSLPRLTPWIVAVA.[V]_	Q13410	549629	545205	587918	86698	231404	1423103	1085738	1058696	669712	286106	203183	198924	216727	110368	46334	789569	815033	752869	859190	394548
[Y].LPNSHPPTVVR.[R]_	P07498	545889	564670	724699	1324863	445647	347292	363536	308277	295730	382666	441547	443576	655524	646769	502192					
[K].DSHSLTTNIMEILRGDFSSANNR.[D]_	P02671	544674	733294	524655		876511	1554553	1410731	751164	3132399	1373361										24469
[L].SPMGEDSAPRDADTLH.[S]_	Q13410	538120	326094	255399	276397	53714	135840	180725	70883	44037	69935	658235	604724	746158	555885	188320	28065	32101	29444	17687	13392
[D].SGSSEEQGGSSRALVSTL.[V]_	P01833	536904	399528	294592	387849	103472	138495	124012	109569	88764	59012	52179	44024	47725	44980	27231	84573	104663	102147	99013	55116
[L].NPTHQIYPVTQPLAPVHN.[P]_	P05814	536857	519822	524195	407567	140125	345442	333262	166465	131320	91888	170586	169077	218749	160823	194885		28276	18268		16872
[L].NEYNQLQLQAAHAQEQIRR.[M]_	P47710	534944	620876	663031	422126	3225276	1731921	1652750	1293812	3674528	6042169	759381	738948	747898	1565226	2804143	22865	55978	41682		34623
[K].VLPIPQQVVPYPQ.[R]_	P05814	532602	476583	448565	595975	486060	3775565	3747728	2146622	2405465	2042182	215542	260869	209843	174215	128623		142123			
[W].SVPQPKVLPIPQQVVPYPQR.[A]_	P05814	527539	537400	525326	137400	286244	1125514	891308	1023653	386199	532697	5594538	5887678	5392058	4254181	3449977	623889	865038	618836	448276	362389
[K].LTDLENLHLPLPLLQPLMQQVPQPIP.[Q]_	P05814	525864	519430	731035	62493	446753	1183860	976200	686951	1171136	2489248	209324	187512	235948	215867	265037					
[S].ALPIIQKLEPQIA.[V]_	Q99541	522489	457897	248160	199270	159985	149129	168212	575438	442276	287312	1917796	2020439	1534652	1484713	937380	341169	370446	366862	268477	92151
[Y].VPFPPFSDISNPTAHENYEKNNVMLQW.[-]_	P47710	519318	826873	656270		242680	754019	813317	492384	575528	326466	137466	79587	103860	112281	80505					
[H].QIYPVTQPLAPVHNPISV.[-]_	P05814	517747	636670	1196266	1044161	361869	3727905	3032079	2188946	1264191	1235073	11644411	10846054	6706017	6855262	6065292	1733698	1686845	1431019	1192433	1831480
[V].VPYPQRAVPVQA.[L]_	P05814	517609	447567	502933	755372	457687	2755096	2164157	1168521	1781362	1910297	821491	919609	913603	1446265	3087969	101086	127509	134805	237965	391030
[M].DPAEEDTNVYTEKHSDSLFK.[R]_	P34741	516703	456743	349211	224954	119757	1344515	1522628	1588269	998475	551617	298459	266706	276802	196903	91094	86929	98811	82298	56529	34313
[A].LPPQPLWSVPQPKVLPIPQQ.[V]_	P05814	511157	406720	445872	649566	144460	314718	237158	471569	148369	96332	335352	411438	385785	325081	305914					
[RK].DPNHFRPAGLPE.[K]_	P0DJI9; P0DJI8	509624	553284	330392	507025	29473	772030	807351	303717	197354	87374										
[D].PRLFAEEKAVADTRDQADGS.[R]_	P01833	507546	351995	402824	172856	291519	205715	278492	533749	194319	269021	187059	138498	131209	123043	99193	41140	46496	49036		38029
[L].PVPQPEIMEVPKAKDTVYTK.[G]_	P05814	507418	360685	529136	304869	1503351	1631804	1733858	7887575	3330349	1295598	466368	413373	309542	387081	302931					
[R].ETIESLSSSEESITEYKQ.[K]_	P05814	505294	402058	479747	932190	1167503	1065415	1094199	832481	1721986	2069611	764894	776428	886297	802173	602539	71867	59184	79862	83363	146353
[N].QPSTQIVANAKGAVT.[G]_	Q99541	503236	468183	364583	383962	103680	295896	353121	250648	180635	124995	1615503	1595196	1364679	1207123	646206	197808	195256	231224	146385	45100
[E].AEQMPEYRGR.[A]_	Q13410	501800	316277	304247	295472	72617	404187	506833	465621	177759	20496	390131	344873	297730	264239	106765	630207	648796	648975	367949	301452
[V].MPVLKSPTIPFF.[D]_	P05814	500410	482263	605674	263224	610854	809110	915665	578541	1407449	1125144	334931	350144	429311	498453	794364					
[P].VQALLLNQELLLNPTHQIYPVTQPLAPVHNPI.[S]_	P05814	499689	514975	725407		212080															
[N].DLEDKNSPFYYDWHSLQVGGLI.[C]_	Q14802	498693	538487	358176		168605															
[G].EGDFLAEGGGVR.[G]_	P02671	498652	352246	379000	489581	39970	157598	175866	69792	63275		62851	57925	91478	61243	15176	139294	134042	140426	107509	56724
[S].FVDLSGHNLANPHPFAVVPSTAT.[V]_	O94985	496909	599740	425674	159416	118745	122684	112160													
[P].VQALLLNQELLLNPTHQIYPVTQPLAPVHNP.[I]_	P05814	493347	474239	598804		188146															
[R].VMPVLKSPTIPFFDPQIPK.[L]_1xOxidation [M2]	P05814	492542	621445	1247796	649111	1251845	975036	1221693	2026743	1510401	1521579		38399		113668	281549					
[D].SGSSEEQGGSSRALVSTLVPL.[G]_	P01833	491044	370080	409842	415111	244621	533319	595131	396411	502734	574623	188178	194429	248762	274587	291103	64699	60595	53291	87307	98864

Quantified modified peptide sequence	Protein ID	A_-80oC	A_-20oC_120h	A_4oC_6h	A_RT_6h	A_RT_24h	B_-80oC	B_-20oC_120h	B_4oC_6h	B_RT_6h	B_RT_24h	C_-80oC	C_-20oC_120h	C_4oC_6h	C_RT_6h	C_RT_24h	D_-80oC	D_-20oC_120h	D_4oC_6h	D_RT_6h	D_RT_24h
[S].EEQGGSSRALVSTLV.[P]_	P01833	490845	336896	342109	347622	161742															
[A].VPVQALLLNQELLLNPTHQIYPVTQPLAPVH.[N]_	P05814	489270	667744	716186	76918	301441	1520446	1265781	725390	1158186	578123	1911050	1406633	1715829	1509978	1433829					
[P].TIPFFDPQIPKLTDLENLHLPLP.[L]_	P05814	489052	670079	574790		781433	5411680	5745770	2707936	5259739	6351187	2756881	2391343	3208487	3057954	3896242					
[A].RPKLPLRYPERLQ.[N]_	P47710	487655	524514	343157		1929619	1133378	1379457	808402	1843331	536606	19408	66788	25007	69611	73647					
[G].IPFAAPTKALENPQPHPGWQGTL.[K]_	P19835	485725	572321	518027	246535	193679	5382282	5208790	4346069	3210380	2050457	172608	201746	196753	176488	104155					
[L].PNSHPPTVVR.[R]_	P07498	481686	538832	587706	670019	299012	276737	292750	239964	211008	259115	427630	553917	689104	554859	356730		19407	16248		12069
[K].EIPLSPMGEDSAPRDADTLHSKLIPTQPSQGAP.[-]_	Q13410	477046	615556	510051		163385	492289	419848	754569	280260	198067						79204	94721	95792		20146
[N].DLEDKNSPFYYDWHSLQVGGL.[I]_	Q14802	476584	493925	468346		211311	196324	211492		139482	72531										
[K].LPLRYPERLQNPSE.[S]_	P47710	474461	281313	311469	107702	432510	5184870	3903055	2751375	3242815	2775337	413198	335869	381167	529385	661075					
[K].GRVMPVLKSPTIPFFDPQIPK.[L]_1xOxidation [M4]	P05814	472451	1163209	2064707	277745	6813025	6926369	6975373	15989442	11755160	11833337	607188	961793	956059	1669316	2679922		43920		54248	184467
[A].RETIESLSSSEESITEYKQKVEK.[V]_	P05814	471982	748928	672843		1488115	2891927	3013896	4592061	5045868	3112815	1445483	1529472	1233292	1757660	2481860	75164	80356	50423		222027
[Q].DPRLFAEEK.[A]_	P01833	462421	410172	384912	742244	298820	519891	657753	673257	681899	174404	132434	156714	155415	138961	35019		24582	18047		
[A].VADTRDQADGSRASVDSGSSEEQGGSSRALVSTLVP.[L]_	P01833	458046	416168	390386	172031	171496	216504	199862	301822	185249	150104										
[A].SVDSGSSEEQGGSSRALVSTLV.[P]_	P01833	457207	338600	328653	454036	162956											26131	29963	19645	27166	21043
[A].VADTRDQADGSRASVDSGSSEEQGGSSRALVSTL.[V]_	P01833	455908	447072	463203	383678	185376															
[Y].PDATDEDITSHMESEELNGAYK.[A]_	P10451	454189	425991	341803	54975	680624	1125178	1165288	1882621	1930484	470764	142778	162570	176594	148587	138042					
[P].LWSVPQPKVLPIPQQVVPYPQRAVPVQA.[L]_	P05814	452265	449265	567088		1775133	862194	74906	568026		697551										
[I].LPLAQPAVVLPVPQPEIMEVPKAKDTVYT.[K]_	P05814	449889	692961	789931	1159218	365174	205722	279618	726871	368390	401751	422473	427476	443513	322310	325910					
[A].KDTVYTKGRVMPVLK.[S]_	P05814	446057	667063	613078		1591031	1104925	1048277	549002	2985472	756378	389772	621602	424111	1508580	1338870		37580			24078
[Q].IPQRQYLPNSHPP.[T]_	P07498	446033	413230	377366	485652	203936	32797	23276	35183	22749	22915	101256	99097	103434	103834	52404					
[N].PTAHENYEKNNVMLQW.[-]_	P47710	445745	612183	658161	287681	972528	383233	330346	231506	524377	871800	177995	194481	226161	355650	438819	104196	160647	97098	96988	283159
[A].LPPQPLWSVPQPKVLPIPQQVVP.[Y]_	P05814	443151	472204	562591	227091	154477						346803	358937	336581	290921	250576					
[L].SSSEESITEYK.[Q]_	P05814	442580	368655	307296	2175394	527606	1779133	1986219	796444	3081359	1531169	2544418	2479817	2465718	3364321	3138417	276981	244435	199598	345501	540398
[E].DQQQGEDEHQDKIYP.[S]_	P05814	442455	338013	325818	3710343	1120418	1030506	1375458	1068301	1189950	1738332	176087	180868	198898	228718	212896	638258	630380	661987	506318	500129
[G].SLSTKLHSRAYQQALS.[R]_	Q99541	441413	736882	435640		228478	365419	411093	285257	578057	276135	6911514	8824174	6730867	5618776	3893110	110251	254231	126559	18999	67604
[E].IPLSPMGEDSAPRD.[A]_	Q13410	436926	331038	244027	481602	65840	237867	268347	195125	155144	81682	788374	774533	798762	658873	297866	119805	128050	124312	72213	20648
[V].MPVLKSPTIPFFDPQIPK.[L]_1xOxidation [M1]	P05814	435026	442602	917054	498426	1016303	6171628	6252042	8680019	2524463	1903208	41832	38856	29810	31198	82177					
[L].ALPPQPLWSVPQPKVLPIPQQVVPYPQRAVPVQA.[L]_	P05814	428532	423003	491572		778411	792579	807689	1054423	590067	479920	933895	990472	907419	930798	2443474	184872	193139	172425		459480
[S].SSYSKQFTSSTSYNRGDSTFESKSY.[K]_	P02671	428101	557121	569073	332515	578984	226665	249401		298432	325705										
[A].EDSLADQAANKWGR.[S]_	P0DJI9	427731	383978	379186	89862																
[L].ALPPQPLWSVPQPKVLPIPQQVVP.[Y]_	P05814	426805	597963		260958	233064						367111	410919	395824	290381	267703					
[L].TDLENLHLPLPLLQPLMQQVPQPIP.[Q]_	P05814	422422	590771	457814	78260	153519						948850	780614	1132683	879651	560502					
[K].RLDINTNTYTSQDLKSALAKF.[K]_	Q9H173	418679	461451	535877		169787	261936	248666	735637	156299	139651										
[R].TYYANPAVVRPHAQIPQR.[Q]_	P07498	418298	1072617	1109035	246506	919339	727574	805752	1856598	1187199	1546609	675980	1009398	1446284	1027132	871987	45918	86289	102748		103994
[I].PQRQYLPNSHPPTVV.[R]_	P07498	418171	439491	413686	213903	236175	302609	333967	225932	224187	177900	74519	78871	72638	60785	42910					
[K].GAGSTTVKIVLK.[E]_	Q6WN34	415954	386767	407161	779280	972619	1293765	1278483	756467	2680952	2538808	47894	59054	52482	67453	53834	49373	74828	52555	59874	92339
[K].SPTIPFFDPQIPKLTDLENL.[H]_	P05814	411503	446344	449898		238968	750879	671286	313029	528293	593114	588581	488339	591559	569303	800000		25217		21586	59078
[I].YPVTQPLAPVH.[N]_	P05814	410164	292932	357314	389248	305691	241676	252634		183557	305042	355162	470775	479772	440117	435406					45361
[H].RIHWESASLLR.[S]_	P01024	408753	726740	241972		225692	342916	407006		300123	208182										
[R].NILREKQTDEIKDT.[R]_	P47710	407291	587600	776891	1504368	2166110	2129552	2295696	3679567	5743532	7384180	68047	88381	73174	195667	221743					
[S].YPARVPPPPP.[I]_	P07910	407111	361357	216177	228141	165309	156291	200305	42792	112421	22435	295767	316338	221467	237924	147342	274631	231436	160069	349994	137772
[K].VKHEDQQQGEDEHQDKIYPSFQPQP.[L]_	P05814	405734	813194	742295		1034831	3375026	3319547	5890975	3214353	3176378	797101	957124	832412	751757	817769		37730			
[N].ILPLAQPAVVLPVPQPEIMEVPKAKDTVYT.[K]_	P05814	405368	417956	586020	119949	522931	1756866	1932476	1757718	1561298	2007541										
[H].SKLIPTQPSQG.[A]_	Q13410	404585	262783	158622	365759	80455						2290535	2494326	570759	1624856	659106	831425	1698170	967613	814432	205485
[R].GRATLVQDGIAK.[G]_	Q13410	404309	374332	271369	480971	107855	641511	745208	998282	687020	251331	544826	563827	477298	437197	196211	1090844	1306458	1153977	622740	219502
[A].LPPQPLWSVPQPKVLPIPQQVVPYP.[Q]_	P05814	397736	435828	500358	120380	159432	403838	402590	256657	228990		1995554	1985504	2091509	1671857	1507226					
[M].KPVPDLVPGNF.[K]_	P02671	395901	381064	770382	1202962	346436	246772	282249	309648	551058	259576										
[R].SVDALDDLTPPSTAESGSRSPTSNGGR.[S]_	Q86X29	388163	219803	260676	273651	99617	206067	242768	342265	153250	102215						51866	45529	48326	53657	23598
[L].AQPAVVLPVPQPEIMEVPKAKDTV.[Y]_	P05814	386576	410424	341170	113058	142684	185794	221150	303094	101317		254331	219816	237372	205493	94074					
[A].VYTEGGFVEGVNKK.[L]_	P19835	385964	331433	453302	799585	287979	1251977	1273674	459239	2255608	975618	71519	66247	102745	319447	144097					
[R].DQADGSRASVDSGSSEEQGGSSRAL.[V]_	P01833	384089	243308	228722	641492	100247	108802	176101	171497	93718	56468	122940	133638	121917	118127	51452	52552	43044	51075	52556	40204
[A].DTLHSKLIPT.[Q]_	Q13410	382219	288541	248460	303263	70725	310216	333754	143808	128094	53597	1313531	1483049	2114800	1363040	667967					
[W].SVPQPKVLPIPQQVVPYPQRAVPVQ.[A]_	P05814	379365	316657	361964	152874	121359	272909	227793	426428	86502	92692	1218201	1316470	846701	984486	1808570	435750	394292	384047	284098	573863
[K].SSSYSKQFTSSTSYNRGDSTFES.[K]_	P02671	379287	416376	836843	422712	248558	131335	201714		206359	447662						60451	58365	45652	45212	103352
[K].SPTIPFFDPQIPKLTDLEN.[L]_	P05814	372460	332702	395768		545255	1629370	1498759	1076013	1163929	1128356	244776	244265	309333	222481	296249					
[R].NGFKSHALQLNNRQI.[R]_	P0C0L4	371176	685144	635902	70150	1047251	44487	57963	103117	79661	46609	2105704	2778590	2942180	2465310	2734941		16098			37981
[A].KLGAVYTEGGFVEGVNKK.[L]_	P19835	370696	627808	736827	82739	395150	2192659	2163468	7818590	4220670	2275961	440775	476378	625164	786026	333257		26525	13008		50641
[P].QPEIMEVPKAKDT.[V]_	P05814	370413	285875	232040	313222	101132	153222	184713	81186	116548	47654	61231	63159	60698	66659	21443					
[P].TIPFFDPQIPKLTDLENLHLPLPLLQP.[L]_	P05814	369816	606504	376361		342219	1947540	1972984	2020179	1556232	1925266	2279419	1847739	2372303	1918516	1497248					
[E].DSLADQAANKW.[G]_	P0DJI9	367037	344583	423108	692769	53408															
[F].DPQIPKLTDLENLHLPLPLLQP.[L]_	P05814	366668	427519	413584		154194	2251996	2024832	1412201	1570421	968610	5775089	5152154	5687560	4609040	2854741					
[A].DTRKMDPSKPSSNVAGVVIIV.[I]_	P22897	365766	374581	418447	148456	117780											109865	118106	93075	61064	81488
[K].TGDPNMGDSAVPTHWEPYTTENSGYLEITK.[K]_	P19835	364890	276691	323972	261297	1011984	2719885	2809817	2099003	3964638	5627310	271321	275023	302479	296816	276077					
[P].DEKVLDSGFREIENK.[A]_	P01833	364506	252215	244072	169698	138878	403730	366516	433815	315871	287016	101690	100143	96730	88412	61486		27527	12473		18046
[A].VPVQALLLNQELLLNPTHQIY.[P]_	P05814	363721	406117	401023	75105	317659	657402	527788	545134	700149	1014270	353868	277678	297493	362519	591242					
[A].QPAVVLPVPQPEIMEVPKAKDTVY.[T]_	P05814	363473	368171	313056		83916															
[Q].VPQPIPQTLALPPQP.[L]_	P05814	362281	690052	448676	667610	174680	607244	537521	410551	291526	251082	2040821	2595545	1649074	1438741	725849					
[Y].YLAGRDLSRLPQLVGVSTPLQGGSNSAAAIGQSSGEL.[R]_	P15291	359613	340022	208845		208558	134026			236257	138844										
[A].NPAVVRPHAQIPQRQY.[L]_	P07498	359502	490012	379764	85920	184032	160815	172093	155844	121387	71847	123254	157917	151502	131291	142356					
[Y].SGGGSALAGGAGGGAGRKEDWNEIDPIK.[K]_	Q10471	353980	438292	351863	57566	170504	247797	228741	310668	168002	148034										
[R].VMPVLKSPTIPFFDPQIPKLTDLEN.[L]_	P05814	353287	381931	568147		1106926	1344283	1243120	1436144	83462	1189487	59018	57481	98230	118054	198013					
[S].IPASSLPRLTPWIVAV.[A]_	Q13410	352660	421247	404447			465035	421593	351692	264108		94973	118139	110128			108875	128677	112361	151518	
[K].DSHSLTTNIMEILR.[G]_	P02671	352040	293607	215401		324223	288716	167715		404795	173604							43413			
[E].SLSSSEESITEYKQKVEK.[V]_	P05814	351243	293026	287225	244651	2567551	4595149	2839787	6452697	6332173	7495687	4389966	2840898	1901629	4541664	3410221					1451360

Quantified modified peptide sequence	Protein ID	A_-80oC	A_-20oC_120h	A_4oC_6h	A_RT_6h	A_RT_24h	B_-80oC	B_-20oC_120h	B_4oC_6h	B_RT_6h	B_RT_24h	C_-80oC	C_-20oC_120h	C_4oC_6h	C_RT_6h	C_RT_24h	D_-80oC	D_-20oC_120h	D_4oC_6h	D_RT_6h	D_RT_24h
[T].ADSGEGDFLAEGGVR.[G]_	P02671	350957	233921	156487	149949							51801	52719	36526	20722		95741	87106	58983		
[R].GWDQEPAREQAGGGWRA.[R]_	Q86X29	347751	312143	379969	229556	59315	202932	227605	156108	187376	50472	59868	58506	72786	61792	40130	147699	234040	241209	259813	270948
[R].SLAGPAGAAPAPGLGAAAAAPGALVRDVHS.[L]_	Q8NES3	346416	355373	362290	131022	80742	158418	121369	172722	71038	35262	63285	20603	43393	29550	15920	518199	465541	435877	444764	258383
[G].FLPQNILPLAQPAVVLPVPQPEIMEVPKAKDTVYT.[K]_	P05814	346174	471474	461407	90074	345456	702821	751723	735456	575000	300161	169166	105795	105017	183908	120040					
[S].SSEESITEYKQKVEK.[V]_	P05814	345709	468201	327103		790105	2133540	2062653	2907062	3517362	1927210	1935544	1948324	1711900	1847635	1653476	22492	37631			85313
[K].LPLRYPERLQNPSESSEPIPLESREEYMNGMN.[R]_	P47710	345473	400853	365485		246979	2024461	2026628	3432309	1826883	1570563	407836	441454	343467	292359	264868					
[S].SEESITEYK.[Q]_	P05814	344830	293405	206183	1011352	166021	5557236	5870649	2179293	5624914	1971609	4445552	4913084	4040849	4867450	2527009	305573	254262	295387	264554	220066
[P].TIPFFDPQIPKLTDLENLHLPLPLLQ.[P]_	P05814	344317	599375	279735		208857	387158	369892	331146	286412	304531	539473	429572	532591	414010	314015					
[G].LPFYNGFYYSNSANDQNLGNGHGK.[D]_	Q96S86	344213	477913	501032	355739	476458	1057284	1088338	970433	1664393	1623114	348787	359017	374470	343224	285953	80290	97205	81580	70682	73992
[S].KLIPTQPSQG.[A]_	Q13410	344136	212670	102472	340770	59980	33681	40057	163374	75711		588575	801456	632064	584110	215983	139429	276153	293634	97454	44551
[R].TLEIPGNSDPNMIPDGDFNSYVR.[V]_	P0C0L4	343003	289227	304830	195998	241216						712445	619646	739841	874053	637937	29807	35319	35319		36645
[A].QPAVVLPVPQPEIMEVPKAKDT.[V]_	P05814	342408			169230	14642		43912		44730	35972										
[L].QLQAAHAQEQIR.[R]_	P47710	342178	372216	295643		1069478	993209	924368	871592	1468927	2344455	169765	175198	178063	172931	186285					
[A].DTLHSKLIPTQPSQGAP.[-]_	Q13410	341517	374446	289378	201825	63723	506515	521142	190096	198590	117801	291785	323605	317343	246085	155728	57435	73591	66256	35394	18127
[S].SGVENALTKSELLVEQY.[L]_	Q99541	340035	311500	253719	115061	96872	224074	219719	186569	166674	95332	1328851	1414975	1378065	1141925	639137	132954	125802	117154		
[R].GAKTTFDVYTESWAQDPSQENK.[K]_	P19835	339866	360953	491866	715874	971586	1914014	1392870	1890060	6531200	6771153	240892	227508	300887	437450	368332	46881	45080	40796	42716	81249
[P].FPPFSDISNPTAHENYEKNNVM.[L]_	P47710	332268	446379	454221	142448		91654	79649	77183	69748	34777	74686	84544	89364	71689	52258					
[K].SPTIPFFDPQIPKLTDLENLH.[L]_	P05814	331776	375110	291015				268842			190595	469353	473936	298404	310287	361668					
[F].DPQIPKLTDLENLHLPLPLLQ.[P]_	P05814	330358	364251	344569		117559	446182	293009	436946	329626	265799	1199804	1073202	1147917	940745	568944					
[K].DTVYTKGRVMPVL.[K]_1xOxidation [M10]	P05814	329896	346040	827787	3397620	2282146	1067609	1394684	2200457	2274403	2123180	184306	294025	233398	818195	1419445		15398		56471	126782
[L].NQELLLNPTHQIYPVTQPLAPVHN.[P]_	P05814	329418	307357	238648		107423						524505	474896	581155	588343	388513					
[E].DGSYEEALVHLK.[V]_	Q13410	328331	319173	272933	560005	654686	804453	823412	637255	1626020	2114897										
[G].DIKVTESEIK.[R]_	Q7Z7D3	328175	341501	266661	529847	85468	269847	266151	289482	231909	156475										
[T].IPFFDPQIPKLTDLENLHLPLP.[L]_	P05814	327259	488628	376683		104633	4396036	4170810	2421797	2788785	1716422	3885859	3576337	4337522	3073069	2233088					
[R].RPDIQYPDATDEDITSH.[M]_	P10451	326047	321028	248239	272179	261132	2534862	3025625	2302526	1776612	1239712	5339526	5255637	4835612	4622602	2982174	202685	212045	189105	177920	177892
[P].LAQPAVVLPVPQPEIMEVPKAKDT.[V]_	P05814	326038	285607	240047	85287	104105	374335	370438	418164	164252	85915	48095	35758	36936	37097	21378					
[R].SVDALDDLTPPSTAESGSRSPTSNGGRS.[R]_	Q86X29	323903	259088	225933	276636	133219	154403	156866	297017	125738	90835										
[F].DVYTESWAQDPSQENK.[K]_	P19835	321903	275460	191618	841157	594186	1638266	1866688	1013644	1565063	2951827	152914	154607	156937	150799	107670					
[I].YPVTQPLAPVHNPIS.[V]_	P05814	321437	287187	210051	189414	179387	108209	117119	98387	109641	271920	124971	144965	130796	93823	67288					
[P].ERLQNPSESSEPIPLESREEYMNGMN.[R]_	P47710	320409	178131	191871		87534	379520	426219	436930	346360	242364	654335	559842	547072	443981	190419					
[C].APPAAGQQQPPREPPAAPGAW.[R]_	P28300	319911	270819	297247	319956	141642	255256	287496	197681	175848	124173	21281	29526	23789	21746	18388					
[A].LPIIQKL.[E]_	Q99541	319444	341362	219207	237974	27690	137634	130913	115460	59957	34504	450699	694927	530094	279445	129582	53890	69164	57091	57478	10067
[A].IESQDAGIKTITMLDEQK.[E]_	O00161	318063	332886	226110	147901	64680	136190	104488	149294	95193	46733	93882	91156	69063	53998	32794	22114	21997	23626		
[D].TRDQADGSR.[A]_	P01833	317356	307413	289879		135129	456887	532309	928641	461437	412662	196874	196837	176177	163363	85578	107693	162406	120338	21796	83092
[A].NPAVVRPHAQIPQR.[Q]_	P07498	316504	609466	644317	133242	700206	261151	293920	415957	458840	880220	202100	281210	348391	273574	239515					
[L].DINTNTYTSQDLK.[S]_	Q9H173	315848	287957	263305	581332	372087	727577	870088	635817	1498871	1077956	69614	67651	55734	65896	33881					
[RK].DPNHFRPAGLP.[E]_	P0DJI9; P0DJI8	314327	234970	131108	308215	60093															
[S].EESITEYKQKVEK.[V]_	P05814	313802	429757	182291	167485	593357	1849219	1760356	2891138	2932612	1253070	1006558	750953	770063	597015	559531		76508			35999
[Y].DGEEIATRGNVI.[V]_	P19835	309700	213516	211418	348742	39836															
[S].VPQPKVLPIPQQVVPYPQ.[R]_	P05814	307009	339411	321155	118517	65729	1693569	1465237	1584939	801106	446205										
[V].ADTRDQADGSRASVDSGSSEEQGGSSR.[A]_	P01833	306919	214766	171323	352406	51676	503409	643464	981452	312595	134889						36501	54450	52341	29285	45485
[L].WSVPQPKVLPIPQQVVPYPQ.[R]_	P05814	306268	300801	341744	197839	731753	709437	716655	591975	518024	968984	21867	79274	21497	46617	107421	27377		19045		41264
[L].GAVYTEGGFVEGVNKKLGLLGDSVDIF.[K]_	P19835	305944	447900	387791		420796	3256904	3086740	2698870	2780151	1449003										
[R].LNEYNQLQLQAAHAQEQIR.[R]_	P47710	302993	300427	575564	392399	928294	336454	285203	661446	1260546	1744307	182149	179116	275691	459414	288964		18328			18173
[G].APLQGQLVARPSSINY.[M]_	O00560	300671	228758	225813	422475	234827						246479	259226	228183	353262	492183	86444	94740	88100	132109	231762
[D].APPPPAAPLPRWSGPIGVS.[W]_	Q99523	298455	247521	251666	147927	33630						139195	157148	153014	122201	64793	33092	38771	29442	27569	16360
[I].YPFVEPIPYGFLPQNILP.[L]_	P05814	292843	321550	312044	101301	353296	566506	522782	246585	423435	339109	103378	87075	136582	142563	137259	42641	60017	60157	128675	118898
[R].DVVLFEKK.[V]_	P00747	291820	189497	312055	548568	29105	257830	231758	277786	199887	16241	136570	109039	144304	103017		22915	19336	23395	36352	8794
[Y].LPNSHPPTVV.[R]_	P07498	289587	366692	423639	589776	212806	404329	415600	263139	296419	477386	277270	337454	483740	418947	289074					
[K].DTVYTKGRVMPVLKSPTIPFFDPQIP.[K]_	P05814	286403	483753	423272		1671196	680400	687153	861797	781154	994530	1156895	1047851	935525	1132346	2648988					
[V].LPVPQPEIMEVPKAKDTVYT.[K]_1xOxidation [M9]	P05814	285942	269919	426083	204382	80261	436819	411438	730860	168737	133599	69599	130080	84378	79727	65894					
[L].GPNTGLSGGMPGALPSLPGKI.[-]_	Q9NS69	284520	195728	194289		57876	124676	131630	126959	62682	41543	54544	56788	60192	52652	18968	37954	50603	35486		19521
[N].TLPTKETIEQEKRSEIS.[-]_	P63313	284133	251948	132586		28861						1114085	935774	775872	568823	286514	136180	186303	151485	59656	38504
[S].TPAPEARPVIGALGL.[-]_	Q96AD5	283610	310610	190626	136532	52158	73195	73906		48653	15982	806817	894493	1080938	710423	398091	232062	235246	226401	143145	46761
[D].APLQPVTPLQLFEGRRN.[R]_	P0C0L4	283049	180901	212948	122476	120713						488365	413243	327505	403200	270022					
[G].DSAVPTHWEPYTTENSGYLEITK.[K]_	P19835	282995	323479	316772	247096	623021	1453154	1413933	943468	1715628	1904349	63867	68702	74445	54179	53023					
[R].TATSEYQTFFNPR.[T]_	P00734	281667	228299	188416	87023		241775	248254	156031	174671		43500	45089	59581	16913		294330	316237	286702	212649	17290
[F].DPQIPKLTDLENL.[H]_	P05814	281424	253995	247196	155060		384884	403375	267218			439133	416169	465213	492856			21916			69756
[V].LPIPQQVVPYPQRAVPV.[Q]_	P05814	280658	316848	355812	438250	776954	2132692	2007302	1996563	1284284	1784589	392436	419019	476149	715319	1106100					
[I].PFAAPTKALENPQPHPGWQGTLK.[A]_	P19835	280174	349031	328885	43752	414602	2292085	2236220	2158289	2934076	2797646	424083	414674	417257	513743	476124					
[P].FPPFSDISNPTAHENYEKNNVMLQW.[-]_	P47710	279819	647076	521893		241150	418942	440812	112736	375053	281917	186601	110201	133780	151261	102047					
[K].VSPAVLVHRDGREQEAEQMPEY.[R]_	Q13410	279624	237687	284287		458767	386378	369149	574103	851908	1378628	51696	54839	66725	65877	90182					
[T].VVDFETDVLFLVPTEIALAQHRANA.[K]_	P19835	278814	406689	273011		83963	3626641	3279534	4140645	2353366	1470847	318137	258701	265399	217334	128657					
[R].ISHELDSASSEVN.[-]_	P10451	277795	195159	164652	800731	122626	841661	980160	174337	689022	28513	1230069	1206291	1310554	2295950	790499					
[L].PLAQPAVVLPVPQPEIMEVPKAKDTVYT.[K]_	P05814	277477	177023	187952		183561	393666	457962	2999376	445781	432526	129662	182422	163999	124507	122732					
[I].PFAAPTKALENPQPHPGWQGTL.[K]_	P19835	277361	373051	364777	242537	996789	3460049	3405524	3910213	3488554	5097929	128625	143865	141516	171287	227171					
[S].VPQPKVLPIPQQVVPYPQRA.[V]_	P05814	275751	339891	403969	115918	65687						91088	119730	107616	97692	116694	30674	42783	36090	23230	29870
[K].LPLRYPERLQNPSESSEPIPLES.[R]_	P47710	275291	252856	276898	111493	74128	524137	457319	777324	375932	346557	324113	258204	270304	220226	132203					
[K].LTDLENLHLPLPLLQPLMQQVPQPIPQ.[T]_	P05814	272190	286965	330366		116142	423791	129333		235305	134410	277696	220826	302217	246584	252822					
[H].AQIPQRQYLPNSHPPTVVR.[R]_	P07498	271084	286965	279751	80911	164822	246731	221049	295672	216316	97710	90255	131449	101292	101746	132649	39921	76571	54579	23486	65170
[M].PVTVTRTTITT.[T]_	Q96S97	269801	197404	166441	313201	60085						100603	113506	141563	58294	44656		34003	38484		
[R].LNEYNQLQLQAAHAQEQIRR.[M]_	P47710	269398	370463	460966	168876	1269645	325820	342893	688796	1765760	2117575	293631	313288	437820	1037197	932475	22767	18254	16886		34797
[I].PQQVVPYPQR.[A]_	P05814	269126	237972	310292	489400	693048	743393	682470	735237	968558	1965387	349837	394024	346726	296886	166505					
[S].TYDLMSSAYLSTKDQYPYLK.[S]_	Q99541	268298	291906	236119		43409	150133	180654		64147	44036	50120	48554	66577	63290	32644					

Quantified modified peptide sequence	Protein ID	A_-80oC	A_-20oC_120h	A_4oC_6h	A_RT_6h	A_RT_24h	B_-80oC	B_-20oC_120h	B_4oC_6h	B_RT_6h	B_RT_24h	C_-80oC	C_-20oC_120h	C_4oC_6h	C_RT_6h	C_RT_24h	D_-80oC	D_-20oC_120h	D_4oC_6h	D_RT_6h	D_RT_24h
[K].AKDTVYTKGRVMPVLK.[S]_	P05814	268210	417724	496810		1536753	1297736	899436	1443659	3174995	856919	684341	963076	656305	1232711	1054867		60790			61591
[-].MHPEPAPPPSR.[S]_1xAcetyl [N-Term]	Q96D31	267730	197751	156845	459291	51140	114869	122175	64854	76902	21841	270588	296353	313273	281025	107374					
[A].AKLGAVYTEGGFVEGVNKK.[L]_	P19835	266805	298858	332134		331774	1978608	1981264	4958039	3923481	1649862	661429	677872	813779	714029	481379	34992	53149	29221		35573
[L].PAEPAAARGGGGGGGGGGGGRGPQA.[R]_	Q86Y38	266624	239013	173114	259597	54132	69419	140566	133638	50887							27209	41251	35085		15793
[K].SPTIPFFDPQIP.[K]_	P05814	265737	216353	312732	1440675	1292311	1338932	1214227	526851	1688738	2931848	464039	431157	532274	1309333	1606240					
[P].VTQPLAPVHNPISV.[-]_	P05814	263260	220198	219667	383510	119307						322481	392598	318427	255609	267758	132276	219046	212091	274950	697177
[P].VPQPEIMEVPKAKDTVY.[T]_	P05814	262007	283642	201911	141987	81182															
[P].THQIYPVTQPLAPVHNPIS.[V]_	P05814	261178	178916	239649	137393	201164	84110	53720	55187			49232		25998	59289	35430		20538			15981
[A].LLLNQELLLNPTHQIYPVTQPLAPVHNPIS.[V]_	P05814	260823	332629	402975	974175	963098	683556	494432	264358	197380	515688	177198	153400	131231	287011	171211					
[S].SSYSKQFTSSTSYNRGDSTFES.[K]_	P02671	260709	223096	323997	321853	190083	26798	63476	80389	79390	193613										
[T].HQIYPVTQPLAPVHNPISV.[-]_	P05814	257753	323682	351524	186008	380149	5602932	5058268	2201928	3346320	4733641	5598139	5476602	4805048	4413658	5169009	285547	518028	290459	348293	602069
[Q].DPRLFAEEKAVADTRDQA.[D]_	P01833	253162	251014	182022	99099	119536	443925	388885	455027	304647	162983	150087	127242	138445	111317	58607					
[T].HQIYPVTQPLAPVHNPIS.[V]_	P05814	251727	224399	167285	60122	109650	213716	179846	145257	155721	182913	660567	650701	621264	686378	447807		29839			
[Y].VPPSSTDRSPYEKVSAGNGGSSLSY.[T]_	P15941	251659	231460	245917	159093	58570	179334	230439	259068	120380	50345	52836	47358	76669	46961	29014					
[R].DTGAALLAESRAE.[K]_	O00391	251577	160241	181938	227401	37191	81588	115459		52637	55168	44234	50943	46208	58313	35613	34425	37068	40930	44843	22435
[S].LSSSEESITEYK.[Q]_	P05814	251230	226762	259358	1634235	354979	1662312	1852151	360012	2171720	1285409	568432	545931	359509	914840	913959	200596	211108	143768	330180	293309
[S].DISNPTAHENYEKNNVML.[Q]_	P47710	250686	208556	175467	127966	98819	119702	112097	106250	62515	70703					23410		18947	26853		27282
[V].LPVPQPEIMEVPK.[A]_1xOxidation [M9]	P05814	250444	247130	318103	456429	120144	739176	790593	951866	401091	445783	118476	143424	156339	144643	407379	18903	27619	14250	58628	62769
[T].IPFFDPQIPKLT.[D]_	P05814	250129	238996	254929		103250	529765	496094	302041	367158	351921	394071	446312	460688	391153	655032		25199	27529	63846	23497
[A].APTKALENPQPHPGWQGTL.[K]_	P19835	249331	194755	232168	117363	259428	2459580	2395594	2101216	2050386	1911890	341365	376864	356736	320240	340144					
[E].EQGGSSRALVSTL.[V]_	P01833	248505	196233	232937	1097182	132147	113994	141580		87343	90548	49062	50139		66790	45577					
[Y].LPLTEEELEK.[E]_	Q99541	248471	193694	151849	288702	49818	94781	108502		64926							24449	20075			
[V].YTEGGFVEGVNK.[K]_	P19835	248418	339569	328124	396028	82975	1951511	2190659	2116078	2278438	910969										
[N].QELLLNPTHQIYPVTQPLAPVHNPIS.[V]_	P05814	248288	269792	138523		57661	318826	313843		143964	314272	910092	782773	899054	831994	551531		54784			
[K].SPTIPFFDPQIPKLTDLENLHLPLPLL.[Q]_	P05814	248283	704981	167884		455773	440349	415699	457374	334518	348903	554266	481174	522962	463813	553233					
[Q].LQLQAAHAQEQIR.[R]_	P47710	247219	261108	244506		864456	784991	766449	800711	1180082	1928616	166643	171523	176076	149603	164938					
[R].DLSRLPQLVGVSTPLQGGSNSAAAIGQSSGELR.[T]_	P15291	247011	120741	104565		50790															
[Y].VPFPPFSDISNPTAHENYEKNNVM.[L]_	P47710	244603	143636	193617		93126	86890	98487	74244	82971	55713	41032	24729	28148	24473	12438					
[D].PRLFAEEKAVAD.[T]_	P01833	243250	221045	176543	204301	132365	312551	323550	304876	424659	948526	163728	188343	144352	124808	50368					
[K].SPTIPFFDPQIPKLTDLENLHLPLPLLQPLM.[Q]_	P05814	242700	677754	237704		1549102	3515729	3132438	5586248	3004271	5878275	6215690	4679458	5149692	4898480	4333489	16938				174230
[R].DQADGSRASVDSGSSEEQGGSSRALVSTLVPLGLVL.[A]_	P01833	242695	239134	206852			478862	465082		70930	47754	354339	312093	338483	250995	147171	178438	160769	196335	89261	102038
[A].DTLHSKLIPTQP.[S]_	Q13410	240917	195426	150373	142043	43739	83063	82002	78944	44621	27760	146317	152556	179864	126824	64561					
[Y].VPFPPFSDISNPTAHENYEKNNVMLQ.[W]_	P47710	239407	220177	178886		447437															
[N].QPSTQIVANAKGAVTG.[A]_	Q99541	238115	258578	203537	169554	69060	126881	149365	136723	73095	56647	125001	143953	129879	100423	56009	36805	51028	47337		
[Y].YANPAVVRPHAQIPQR.[Q]_	P07498	238080	283504	507715	59165	330366	193622	252239	643800	412915	584923	205891	219313	367055	232226	281669					
[L].TGRGAEDSLADQAANKWGR.[S]_	P0DJI9	237974	296826	206075		21953															
[V].DSGSSEEQGGSSRALVSTLVPLGLV.[L]_	P01833	237248	257351	241313		74572	225445			142072	91894										
[P].DIQYPDATDEDITSH.[M]_	P10451	234977	170219	156809	290016	101296	708606	817242	717279	399008	471462	2127105	1954389	1942339	1688606	638286	93150	80636	93880	79251	65931
[R].DQADGSRASVDSGSSEEQGGSSRALVS.[T]_	P01833	233866	216013	162505	283482	84943															
[L].NPTHQIYPVTQPLA.[P]_	P05814	233860	168291	136201	218328	220247	190606	177222	77092	157915	324073	100966	81158	122224	370619	1077184				24988	232355
[Q].YPDATDEDITSH.[M]_	P10451	233560	197858	148794	362075	41398	586813	825758	416869	372820	203236	2556187	2628570	3257935	2894038	1490120		32172	36208		27215
[A].DSHPSYHTDGFN.[-]_	Q9H3Z4	232953	179729	115474	243004	25590	197765	210264	175199	119261	35008	206591	206433	149413	166597	81428					
[V].EISIPASSLPRLTPWIVAVAV.[I]_	Q13410	231959	261217	316922		26867	759818	548792	435197	269740	126882	34516	47291	59667	23722						
[V].LPIPQQVVPYPQRAVPVQALL.[L]_	P05814	231105	253820	283772	122548	144495	962973	860270	768711	579694	550209	1992313	2075432	2198347	2077544	2031126					
[L].NPTHQIYPVTQPLAP.[V]_	P05814	230455	203198	194505	100343	106211	369573	364761	148131	240710	416007	559151	565747	630720	520261	508124	30160	84096	30549	55509	33986
[P].FPPFSDISNPTAHENYEKNNVMLQ.[W]_	P47710	230386	278902	179874	97281	82067															
[T].DLENLHLPLPLLQPLMQQVPQPIPQ.[T]_	P05814	228760	256859	216276		66327	105488	102094		248169	67632	219261	198897	286027	184034	124128					
[T].LPLDGETVKYCV.[D]_	Q86X10	228459	186807	190410	533889	454253						136847	140057	170188	623237	633235					
[S].VPQPKVLPIPQQV.[V]_	P05814	228264	234641	226119	300388	82397	381149	256911	328997	209698	231684	306134	355531	335848	461161	646577	109159	108745	109583	90975	100092
[T].DLENLHLPLP.[L]_	P05814	227187	331746	376054	283771	728973	1155598	1405763	717906	1562320	4732524	541769	574088	697774	528820	699462					
[A].EDSLADQAANKW.[G]_	P0DJI9	225332	132260	199679	226209																
[L].QNPSESSEPIPLESREEYMNGMN.[R]_	P47710	225100	174692	149660	103856	40474	1624615	1589832	1127222	1117611	459327	1720271	1691073	1695921	1304721	417569					
[P].FPPFSDISNPTAHENYEKNN.[V]_	P47710	223093	178124	209673	168624	58898	118076	149382	94391	135986	54011	119610	112411	269042	98270	59830					
[-].MEAARDYAGALIRPLTF.[M]_1xAcetyl [N-Term]	O60543	222537	301407	233503		63846						1499893	1521359	1758994	1505161	983620	161572	180429	173624	153017	53071
[S].KEIPLSPMGEDSAPRDADTLHS.[K]_	Q13410	222101	224057	150169	98547	57368	136749	118504	251732	109218	48488	149889	160223	135735	100495	53066	55100	89295	104218	39989	23241
[K].EIPLSPMGEDSAPRD.[A]_	Q13410	221297	217500	205357	361409	42634	104470	65596	319732	33707	35319	142349	166553	203061	122967	73876					
[S].SGVENALTKSELLVEQ.[Y]_	Q99541	219120	255208	202159	134823	40955	154516	152220	175557	128352	76568	1410267	1334168	1415237	1093247	579976	142730	137574	134796	92525	43822
[E].SSKPNMIDAATLK.[S]_	P02679	217888	166347	155654	218351	71873	85973			81064	47463										
[T].DRSPYEKVSAGNGGSSL.[S]_	P15941	217717	167911	171748	125999	19412	188878	228935	114626	75794	26809	103451	87445	158175	84941	64428		25350	29617		18369
[K].LGAVYTEGGFVEGVNK.[K]_	P19835	217101		172391	102463		338338	325518		302731	244695	40728	49832	63487	36395						
[A].LPPQPLWSVPQPKVLPIP.[Q]_	P05814	216995	211112	226221	79002	91321						244769	252636	247314	256919	358505					
[E].DTNVYTEKHSDSLFK.[R]_	P34741	216608	143418	102634	82110		551551	594215	422737	150036	113484	217884	135850	138269	108758	54703					
[Q].DGIAKGRVAL.[R]_	Q13410	216317	214661	189864	299360	76690	145835	144524	161738	155817	178899										
[H].EDQQQGEDEHQDKIYPSFQPQP.[L]_	P05814	216250	162280	130285	169672	54602	334761	324406	322325	238411	168953	457108	388389	435896	442225	223466					
[K].SPTIPFFDPQIPKLTDLENLHLPL.[P]_	P05814	215941	476894	252433		1002511	1724362	1961282	1005891	1610040	2564773	1116330	1055019	1235896	851772	671956					
[S].SSEESITEYK.[Q]_	P05814	215564	151042	162270	712785	168458	1486417	1664572	1054723	1523576	580447	3576411	3723030	3767147	4317612	1972715	147327	144425	128205	144487	139741
[R].TYYANPAVVRPHAQIPQRQYLPNSHPPTVV.[R]_	P07498	214938	416660	366485		347535	800177	702145	1096482	658831	727756	420482	609870	506247	407424	503271					
[K].SSSYSKQFTSSTSYNRGDSTFESKS.[Y]_	P02671	214287	176861	238329	176573	127271	80552	82213	104527	81263	73263	67378	50985	56386	59180	50290	79731	99272	90920	57716	69418
[R].RPAIAINNPYVPR.[T]_	P07498	213370	254609	311899		142927	999943	952361	2462814	831281	1120000										
[K].GIPFAAPTKALENPQPHPGWQGTLKA.[K]_	P19835	212091	234937	242600		86078	728690	674151	995557	492032	376882	138907	127849	126103	126268	123704					
[D].GREQEAEQMPEY.[R]_	Q13410	211791	187832	168736	429579	192522	318159	473664	393123	409338	437276	31826	36961	55598	59975	60044	37833	41597	59966	41977	81282
[P].TIPFFDPQIPKLTDLENLHLP.[L]_	P05814	211455	279453	220655		196412	217332	200699	250608	211988	377558	148077	131007	155843	191727	353900					
[A].TLVQDGIAK.[G]_	Q13410	211370	243238	192708	279207	44547	140138	148625	308025	118814	44577	176424	195243	246443	104507	29333	836507	1006591	902622	786295	220412
[A].APDEKVLDSGFREIEN.[K]_	P01833	210622	168923	142616	71811	67585	249473	284083	270437	184646	176888										

Quantified modified peptide sequence	Protein ID	A_-80oC	A_-20oC_120h	A_4oC_6h	A_RT_6h	A_RT_24h	B_-80oC	B_-20oC_120h	B_4oC_6h	B_RT_6h	B_RT_24h	C_-80oC	C_-20oC_120h	C_4oC_6h	C_RT_6h	C_RT_24h	D_-80oC	D_-20oC_120h	D_4oC_6h	D_RT_6h	D_RT_24h
[S].EESITEYKQKV.[E]_	P05814	209960	206524	201950	1237411	341501	735253	787643	710137	971366	511961	1816290	1847225	2365991	3779966	3550758	117251	143149	118974	131881	204281
[K].ADAAPDEKVLDSGFREIENK.[A]_	P01833	209802	326423	195058	104964	146857	320508	466428	556772	311374	813011	120305	254888	99491	146051	68550					
[D].PRLFAEEKAVADTRDQADGSR.[A]_	P01833	208998	222612	239922		112683	394716	425180	809094	380548	364156	153456	154217	145034	107850	64372	52772	88296	74047		64749
[D].RSPYEKVSAGNGGSSLSYTNPAVAATSANL.[-]_	P15941	208921	274298	204415		63602	265049	228780	497697	177880	89540	127397	116700	119998	80503	45793	96193	121685	118441	38948	27315
[P].IPQQVVPYPQRA.[V]_	P05814	207835	219830	190270		128692	362892	361692	284446	269196	278747	279567	285658	295575	389938	439574					
[S].VPQPKVLPIPQQVVPYPQRAVPVQ.[A]_	P05814	205522	181572	244052		120143	138627	117851	514125	91479	105157	113914	198866	138115	317857	955964	46405	60035	73545		187696
[L].VGVSTPLQGGSNSAAAIGQSSGELR.[T]_	P15291	204801	206224	183810	169193	60811	71437	80370		37397		75874	76554	65668	71163	48886	18147	23445	23299		
[K].GIPFAAPTKALENPQPHPGWQGT.[L]_	P19835	203981	203143	228100	97026	141307	253341	309997	197384	183550	569719										
[E].DTNVYTEKHSDSLF.[K]_	P34741	203856	143778	208449	451263	201919	287708	312120	204178	330461	493674										
[I].LREKQTDEIKDTR.[N]_	P47710	203389	333376	514800	156267	559818	453049	582983	940341	1662998	1115193	160508	172479	189284	554035	488468					
[K].SPTIPFFDPQIPKLTDLENLHL.[P]_	P05814	203306	317994	256035		206305	309892	289163		247234	353725	180318	157566	189372	247873	598126					
[K].LPGQGVHSQGQGPG.[A]_	Q92896	201267	155493	119825	448791	31928	58289	58673	48411	43793		196130	200166	230121	186021	77083	137079	173408	144555	161073	83294
[N].PAVVRPHAQIPQR.[Q]_	P07498	201083	173189	356494		250962	191722	253231	535689	314225	545006	29986	33210	41587	35727	56978					
[A].VVLPVPQPEIMEVPK.[A]_	P05814	200274	238413	204811	477413	153706	565684	590659	327521	798262	394881	312217	351599	400933	751758	934654					
[S].DMREANYIGSDKYFHA.[R]_	P0DJI9; P0DJI8	200085	163499	196481	58011	95946	106272	80331		75736	66932										
[S].DISNPTAHENYEKNNVMLQ.[W]_	P47710	198465	145313	130232	159615	121530					28699										
[L].NQPSTQIVANAKGAVT.[G]_	Q99541	197490	121752	87235	80621	44979	148691	164100	132615	102782	71518	1112586	1089470	1015438	781638	605638	122382	121075	122012	87016	34494
[A].GSTTVKIVLK.[E]_	Q6WN34	197482	187130	152203	316360	366198	674329	604567	166717	1014348	1033553										
[R].APWVEQEGPEYWDRETQKY.[K]_	P30510	197097	167133	181668	100883	74302											79978	89998	98899	60506	41267
[V].YTKGRVMPVL.[K]_	P05814	196781	251526	95895	596092	1046737	249407	268214	290583	693008	565526	148069	188614	187600	409578	428223					
[K].NPLPSKETIEQEKQAGES.[-]_	P62328	196683	152429	135738	114722	33560	77705	95612	123511	54437	22518	37340	41434	49770	36659	13811					
[K].AKDTVYTKGRVMPVL.[K]_	P05814	196439	299232	187273	159807	1552765	1189906	1171532	4352102	3439345	2048912	896034	938708	486556	839504	1088644	22185	28380	26224		135233
[R].DTGAALLAESRAEKNRLWGPLE.[V]_	O00391	195660	213540	192271		113522	41575	51340	63015	30805		41995		52413	37589		22104	25773			23131
[R].SPYEKVSAGNGGSSLSYTNPAVAATSANL.[-]_	P15941	195002	209589	167232	73683	35386	164057	177108	152081	62109											
[K].NTLPTKETIEQEKRSEIS.[-]_	P63313	194681	203858	148534		74284	182271	228137	312597	135648	96052	46709	49234	41245	40765	16034		41266	36004		
[L].AQPAVVLPVPQPEIMEVPKAKDTVYT.[K]_1xOxidation [M15]	P05814	194091	138662	230130	135634	49529	399038	452324	893698	222199	113020	101672	127069	113209	94262	96109					
[P].VPQPEIMEVPKAKDTV.[Y]_	P05814	193932	136238	140015	135962	40147															
[D].SAPRDADTLHSKLIPTQPSQGAP.[-]_	Q13410	193630	237338	161407	93884	72195	191820	163049	283176	220474	357337	982394	1034873	940577	729567	476008	73598	102994	81853	42366	30758
[A].LPPQPLWSVPQPKVLPIPQQVVPYPQ.[R]_	P05814	192639	213064	256977	97352	75033	1108152	994131	1516454	748620	253260										
[A].DTRDQADGSRASVDSGSSEEQGGSS.[R]_	P01833	192420	126905	95812	506460	67751	192358	342850	471232	190674	76659	327843	337161	394260	373975	95815	34384	23408	45162		17081
[-].MEAARDYAGALIRPLT.[F]_1xAcetyl [N-Term]	O60543	189848	200029	199712		77388	172573	164032		107891	66127	1151825	1112272	1260422	1189612	934301	164765	157601	136750		54757
[V].PPSSTDRSPYE.[K]_	P15941	189424	156846	102433	388835	29169	115632	134976	109572	62379	13660	50972	64439	59195	45829	32381	54575	59440	50957	30805	7887
[Q].EPAREQAGGGW.[R]_	Q86X29	189059	169071	114416	555539	42275															
[R].SPRGWDQEPAREQAGGGWRA.[R]_	Q86X29	188423	284505	211263		35868	250659	185982	233897	154208	30069		29888	28180				45220	17958		21965
[R].DLSRLPQLVGVSTPLQGGSN.[S]_	P15291	187189	180996	186326		46705															
[P].FPPFSDISNPTAHENYEKNNVML.[Q]_	P47710	186343	159423	153694	62804	47252						42905	23935	35949	27225	15931					
[F].AEEKAVADTR.[D]_	P01833	186120	152749	140871	92505	133259	174511	161803	134280	295203	274430	106106	112172	96339	105455	124166	82709	134698	75004		34231
[A].ENGVKTITSVAMTS.[A]_	Q99541	185912	143882	131940	99051	25509	88581	87987	76716	72263	28319	284356	276124	292721	208531	129303					
[A].GRDLSRLPQLVGVSTPLQGGSNSAAAIGQSSGEL.[R]_	P15291	185821	221653	206209		117044	114533	116101		62393											
[R].DLPAEPAAARGGGGGGGGGGGGRGP.[Q]_	Q86Y38	185331	154835	127882	334725	85811	53305	65139	42912	38265	33514	37765	46251	34213	41833	31110					
[P].NHFRPAGLPEKY.[-]_	P0DJI9; P0DJI8	185220	205778	210294		142256															
[S].SSYSKQFTSSTSYNRGDSTFESK.[S]_	P02671	185135	250511	209124	94483	73614	206720	188849		201246	129696	38809			23638	52104		32918	21462		
[P].VLKSPTIPFFDPQIPK.[L]_	P05814	183442	227364	342600	287274	2414353	4831416	4842912	4284403	7948904	10772972	64096	66316	55127	164435	226445					35027
[L].PLAQPAVVLPVPQPEIMEVPKAKDTVY.[T]_	P05814	182456	89732	163299		88818															
[R].SPYEKVSAGNGGSS.[L]_	P15941	182455	176709	122505	522577	53420	121099	164042	234467	85187	20808	35440	48373	85826	41476	34406	31466	36581	42152	24821	15684
[L].FAEEKAVADTRDQADGSR.[A]_	P01833	182056	156193	144540	78457	19773	156077	250828	262074	119843	60156	114764	185231	95469	85921	32471	554414	712463	588053	367380	178815
[Y].ANPAVVRPHAQIPQRQYLPNSHPPTVV.[R]_	P07498	181967	347966	248297		251297	387753	345148	489341	311713	269736	458286	580880	391794	389581	357432					
[K].AIQDPRLF.[A]_	P01833	181548	147396	147446	199255		162977	147608		87254	34977	204313	234287	210056	218627	69893	245350	237905	255517	203621	42715
[V].MPVLKSPTIPFFDPQIPKLTDLEN.[L]_	P05814	181348	279827			86381	776092	74103	357234	564226		156195									
[L].LNQELLLNPTHQIYPVTQPLAPVH.[N]_	P05814	180892	217787	198598		120407	137697	157128	362696	138734	108961	254322	204106	296229	278618	436690					37167
[R].NILREKQTDEIKDTR.[N]_	P47710	180281	329674	433513	207288	286508	360752	386640	667878	1058262	520204	131566	164474	177439	467180	586699		16673			24251
[K].YPDAVATWLNPDPSQKQN.[L]_	P10451	180197	179613	173143	134220	56519	188440	177909	92185	112156	90802	674355	660467	715233	547956	334400					
[R].EEAPSLRPAPPPISGGGY.[R]_	P02675	179707	147709	179306	392386	363508	228107	235659	151457	301187	587292		18622	28101	27739	31316					
[V].FPYPYPFRPLPP.[I]_	Q8NFU4	179449	150224	148346	69901	96301															
[T].DLENLHLPLPLLQ.[P]_	P05814	179280	161928	126662		222498	255353	228353		272429	442507	90982	62367	89369	175354	434912					36054
[P].AVVLPVPQPEIMEVPKAKDTVY.[T]_	P05814	179082		42000		124009															
[S].VPGSQTVVVKEITIH.[T]_	O00592	178558	127010	118510	126026	50465															
[L].SPMGEDSAPRD.[A]_	Q13410	177645	106414	75612	343792	34751	83501	91676	50470	57112	16364	352635	489183	414531	309641	141583	78981	69103	57971	38299	10303
[G].IPFAAPTKALENPQPHPGWQGT.[L]_	P19835	176726	144341	159470	75485	30199	136409	134926	76035	89971	83898										
[A].DAAPDEKVLDSGFREIEN.[K]_	P01833	176136	160928	147503	133172	125665	92166	132105	66387	102496	221905				59222	27353					
[S].SEESITEYKQKVEK.[V]_	P05814	176021	308789	330886		699805	883782	695654	1792527	4952355	804100	725140	630663	554748	684123	1084795					37887
[A].VADTRDQADGSRASVDSGSSEEQGGSSRALVSTLVPL.[G]_	P01833	175718	119856	190883	188691	113118	111089	166636	204142	231646	39656	152087	151813	147486	170156	43159					
[R].LFAEEKAVADTRDQADGSR.[A]_	P01833	175696	213215	126208	60082	25014	225452	249535	332280	138798	69360	141527	196804	137108	98249	36069	614709	812191	677149	251403	119881
[C].EVWTQRLHGGSAPLPQDRG.[F]_	Q92673	175665	164281	143083		46015	89578	121880		60628	26915	143904	143011	167038	100873	50421	209178	354068	223232	85886	174692
[R].YPERLQNPSE.[S]_	P47710	175632	101347	64814	90697	39420	3805405	3302547	653661	2696668	2366883	738463	562722	451408	804577	435448					
[E].RLQNPSESSEPIPLESREEYMNGMNR.[Q]_	P47710	175536	212595	141097			9316511	8827454	8587081	7033590	5166790	6079306	6993018	5792459	5741870	6611978	364804	592861	482595	168901	454801
[Y].NQLQLQAAHAQEQIR.[R]_	P47710	174601	196674	223332	96085	1054675	856450	861498	674681	1380595	2450893	128910	122774	126671	137264	156774					
[Y].TEGGFVEGVNK.[K]_	P19835	173817	166839	275718	760904	148563	368980	493558	312134	786854	423752	47021	49745	66181	92076	43935		19177		25823	23873
[K].HEDQQQGEDEHQDKIYP.[S]_	P05814	173767	173093	142536	881489	801208	602406	822832	1172865	1192944	1532035	130440	119703	130048	176841	222145	412334	568489	511376	392957	498297
[P].LWSVPQPKVLPIPQQVVPYPQRAVPVQALL.[L]_	P05814	173194	105060	107908	96278	59470															
[S].APRDADTLHSKLIP.[T]_	Q13410	172055	209263	145422	53134	43292						89867	105156	117438	89026	42811					
[T].DPEDVIRN.[A]_	P24844	171982	136619	108049	374554	38070	30336	31952				39963	46793	57639	44261	10834	28909	31512	30652	29049	14242
[A].LPPQPLWSVPQPKVLPIPQQVVPYPQR.[A]_	P05814	171966	194089	159287	58099	205874	482529	476905	448539	352068	377678	1329610	1424132	1282731	983617	1116165					
[L].NQELLLNPTHQIYPVTQ.[P]_	P05814	171930	198172	165384	276003	440089	3386713	2451328	1343846	3007306	3438751	629176	588436	652561	1879963	4742543	125902	153388	143817	212359	1196697

Quantified modified peptide sequence	Protein ID	A_-80oC	A_-20oC_120h	A_4oC_6h	A_RT_6h	A_RT_24h	B_-80oC	B_-20oC_120h	B_4oC_6h	B_RT_6h	B_RT_24h	C_-80oC	C_-20oC_120h	C_4oC_6h	C_RT_6h	C_RT_24h	D_-80oC	D_-20oC_120h	D_4oC_6h	D_RT_6h	D_RT_24h
[L].PLAQPAVVLPVPQPEIMEVPK.[A]_	P05814	170831	184519	56095	45357	233752	203442	1658592	2913929	748162	614077	214420	88443	167119	158844	307651					
[Y].PVTQPLAPVHNPIS.[V]_	P05814	169998	221253			52630															
[I].QLTYNPDESSKPNMIDAATLK.[S]_	P02679	169481	144783	143585	89296	129458	81163	68087		76281	66515						38716	44768	37905	39832	19270
[N].LHLPLPLLQPLMQQVPQPIPQT.[L]_	P05814	169425	175557	199412		471096															
[R].SPYEKVSAGNGGSSLSYTNP.[A]_	P15941	169299	164637	110960	85735	33114	69494	78049		48389	29535							17530	21761		
[R].EEYMNGMNR.[Q]_	P47710	169128	135258	90789	614146	185874	1753519	2075280	575112	2312986	870182	700852	773502	634648	830739	484195					
[Q].NPSESSEPIPLESREEYMNGMNR.[Q]_	P47710	169005	81659	100352	266132	430285	3167248	3551436	2577805	3422455	1975000	3537356	3327404	3675896	3336642	1916999	79488	55164	62900		26976
[A].VVLPVPQPEIMEVPKAKDTVYTK.[G]_	P05814	168905	196232	165292	97208	381731						173002	171835	152807	260351	625853					
[K].HEDQQQGEDEHQDKIYPSFQPQP.[L]_	P05814	168851	191182	155607		110851	575505	537965	581297	510701	331166	580130	460045	541784	423690	262840		32519	40531	34859	21618
[F].DQGADAIYDHINEGKLWK.[H]_	Q9UDW1	168527	203872	195995	70721	217828	417296	433272	402018	671235	414731										
[L].GAVYTEGGFVEGVNK.[K]_	P19835	168465	189763	239370	375968	130401	568911	526760	1366673	877200	660732	110151	103254	115490	99469		31197	35321	33772	77815	49413
[D].DPDAPLQPVTPLQL.[F]_	P0C0L4	168357	159543	187512	332106	51049						426705	404244	507749	417536	237368					
[P].LAQPAVVLPVPQPEIMEVPK.[A]_	P05814	168172	178404	245949	741626	1734456	1178288	1547274	1131967	2937710	6087469	144156	147017	161436	390616	800474	31073	30802	21984	62910	68583
[F].GPGKPPPPPPPP.[A]_	A0JLT2	168145	150672	110650	249878	33534	55764	74497		40055	16891	109873	114725	105253	101348	52229	37744	64735	43025	26913	20101
[V].LPIPQQVVPYPQRAV.[P]_	P05814	167893	83766	126861	94124	311042	1558935	1263477	654386	826426	1627854	203083	260504	284890	584485	779896					
[R].DPEAASPGAPSRDVL.[L]_	O43581	167861	85277	112206	177789	28440	44819	71844	73320	48839	27070							18922			13437
[P].LWSVPQPKVLPIPQQVVPYPQ.[R]_	P05814	167618	216354	370101	200812	4756379	1232018	1155526	893617	1507952	4236609	32552				276701					
[E].ESITEYKQKVEK.[V]_	P05814	167612	373673	356260	43693	384511	1621332	1615729	2629147	2220266	961477	1185173	1061767	985438	916469	703740	23814	16248			39793
[P].IPQQVVPYPQRAVP.[V]_	P05814	167130	130153	112143	132475	69325	208167	258443	183928	151933	220093	190680	241046	213817	187172	172078					
[Y].VPFPPFSDISNPTAHENYEKN.[N]_	P47710	166912	146371	118305	58356	24001	63452	68711	60664	38340											
[R].GLEEELQFSLGSKINVK.[V]_	P0C0L4	166270	189354	97970		452138	62357	53632		139983	251036	805316	850036	1173798	1425191	1249517					
[L].ENLHLPLPLLQPLMQQVPQPIPQT.[L]_	P05814	165791	293591	106140	63983	67300	740803	619192	301539	497261	267563	1369007	1080489	1416102	1181422	880611					
[V].PQPEIMEVPKAKDT.[V]_	P05814	165719	146265	149502	96158	36392						64595	64957	80856	55499	32927					
[R].LQNPSESSEPIPLESREEYMNGMNRQ.[R]_	P47710	165562		85591	115403	114286	638669	772886		737015	819565	190295	221587	187684	156445	99924					
[A].NPAVVRPHAQIPQRQYL.[P]_	P07498	165463	193090	168537		150693	48830			47756	55276			22208	40870	59473					
[M].GEDSAPRDADTLH.[S]_	Q13410	165152	162556	165793	257587	28095	101088	116573	236545	92835	49186	490051	502401	432130	406382	191697	51387	52716	54372		13066
[N].EYNQLQLQAAHAQEQIR.[R]_	P47710	164265	89626	176008	695827	874937	259688	253219	169815	694656	1569107	76807	71930	114684	325102	299084					
[K].AVADTRDQADGSRASVDSGSSEEQGGSSRAL.[V]_	P01833	164085	164419	154431	214057	52021	73123	87740	200556	81135	30157	73955	98811	80377	78389	61847		32377	28123		18167
[G].KPGDQILDWQYGVTQAFPHTEEE.[V]_	Q6UX71	163952	190867	155482		90553	93181	136806		106169	51436										
[C].APPAAGQQQPPREPPAAPGAWR.[Q]_	P28300	163561	110073	82929	66422	46270	155894	152204	168061	103691	56562										
[S].APRDADTLH.[S]_	Q13410	163481	41248	28689	252937	25490						14670	34703	33782	15869	17211					
[R].TYYANPAVVRPHAQIPQRQYLP.[N]_	P07498	162985	256711	239746		144517						344467	413707	370067	344443	427223	20889	50858	36241		57606
[Q].TDEIKDTR.[N]_	P47710	162377	220020	268879	130157	667871	324806	373591	970861	1016700	1270408	139112	171341	145218	162970	186387					
[A].APDEKVLDSGFREIENK.[A]_	P01833	162217	154109	178350		38189	222463	203229	291580	142832	145691										
[M].DPAEEDTNVYTEKHSDS.[L]_	P34741	160276	129363	85541	431495	31031	149491	171355	171733	95931	42693	55806	48763	66725	52669	19435	39985	36347	43577	28809	23509
[G].DSAVPTHWEPYTTENSGYLEITKK.[M]_	P19835	160265	144524	138090		163904	888845	845202	799175	908458	495534	288824	267467	317053	226429	146669					
[V].LPVPQPEIMEVPKAKDT.[V]_1xOxidation [M9]	P05814	160223	133621	172558	135960	42949	196274	238594	321247	125476	46098	28263	35208	27425	34708	22518					
[K].FRRPDIQYPDATDEDITSHMESEELNGAY.[K]_	P10451	159572	219647	181542		417912	1901935	1783558	2579018	2025471	2931435					131831					
[K].VLPIPQQVVPYPQRAVPVQALLL.[N]_	P05814	158922	215785	197681	181088	1045339	1718619	1597815	1191033	1804020	3520020	970778	910527	959942	1478604	4812731	149699	83277	125365	295988	255101
[K].DKWDELKEAGVSD.[M]_	O00592	158860	94592	96592	121882	40398	153625	159849	135168	103967	73498	78483	75498	67219	61063	32500	115309	115484	103260	83758	59331
[K].GRVMPVLKSPTIP.[F]_	P05814	158400	144863	161187	219806	703566	810682	657089	1930681	1052427	2296851	824442	976650	789320	1406517	4086617	35660	77533	45399	51683	355589
[R].ETIESLSSSEESITEYKQKV.[E]_	P05814	158130	114802	90316	45688	41727	291823	336398	305068	154777	87478	733245	734413	771098	587248	324415	32308	31830	26436		21415
[A].DGSRASVDSGSSEEQGGSSRALVST.[L]_	P01833	157891	138906	124718	221632	55198	126725	167437	181459	88872	58871	639385	603320	518691	620118	309596	29518	36492	43211		29064
[Y].VPFPPFSDISNPTAHENYEKNNV.[M]_	P47710	157773	137240	114669	49795	32683	105448	103787	80955	52957	27862										
[R].APWMEQEGSEYWDRET.[R]_	P13747	157587	140289	128821																	
[R].LFAEEKAVADTRDQADGSRASVDSGSSEEQGGSS.[R]_	P01833	157171	171198	174136	470697	108572	509927	517061	1075075	924491	959860	67813	76328	46560		48644					
[R].RPDIQYPDATDED.[I]_	P10451	157122	86960	111978	134350	15172	70913	85592	117196	37813	15849										
[A].PAPPPPPPP.[P]_	Q12830	156818	149186	122440	416732	45847	43970	71203	69390	34723	17914	67593	75827	100713	82374	33567	37564	59770	43243	39366	10860
[I].SHELDSASSEVN.[-]_	P10451	156313	148431	132624	335691	82668	394778	592194	432015	348967	33717	1141843	1139612	1246886	1843121	803701					
[P].VPQPEIMEVPKA.[K]_	P05814	156267	105379	155947	218713	102969	212636	230999	186926	168781	241574	67669	93057	64797	75646	61527					
[Y].DVEKNNSRIKL.[G]_	P10412	156187	157125	103019	210946	46224															
[S].SEEQGGSSRALVSTLV.[P]_	P01833	156017	120423	133184	84450	68186															
[E].RLQNPSESSEPIPLE.[S]_	P47710	155964	139459	62997		19383	4044474	4517213	3190723	2564532	2956563	3530208	4345654	4086412	2255148	1365348	1151655	2130119	1984745	1564739	569961
[K].CAEQFCRLNEYNQLQLQAAHAQEQIR.[R]_	P47710	155663	65162	289196		244188	824847	1017566	1676126	657808	394570	958022	1058158	1172757	1156428	1173382					
[E].PAAARGGGGGGGGGGGGRGPQA.[R]_	Q86Y38	155024	124507	112508	206697	38202	57424	78814										31693	20761		
[S].YPYYGTNLYQRRPAIA.[I]_	P07498	154706	138317	165757	169042	59523						327799	313485	347546	230299	181561					
[A].RPKLPLRYP.[E]_	P47710	153914	204606	81382		125135	766213	680645	300486	902508	732911	525777	727359	373592	565849	384032		19964			8481
[A].RETIESLSSSEESITEYKQKVE.[K]_	P05814	153758	242284	149443		1050671	442187	492790	628940	898562	1445127	460365	403793	359370	404973	389382	91315	75160	56672	55192	146666
[V].TQPLAPVHNPIS.[V]_	P05814	153648	131955	85600	251388	102636															
[Y].EKVSAGNGGSSLSY.[T]_	P15941	153312	120785	100870	305702	40956	45484	50312	288666	67070		42392	49384	61731	47969	26091	34378	28943	34570	30297	18761
[A].NPVEGSSGRQV.[T]_	Q15365	152937	126531	91238	407460	14504	21190	23181		13799		21337	24953	37427	18959		20436	19781	19346		
[A].LPIIQKLEPQ.[I]_	Q99541	152421	118089	103580	81548	25254	39482	45855				39294	47824	67917	46938	35889					
[K].DTVYTKGRVMPVLKSPTIP.[F]_	P05814	150522	162734	138833	178446	291648	361316	286911	400294	379763	357530	1141616	1339529	1062042	1362409	2790661	68415	106487	109120	107265	336795
[S].NPTAHENYEKNNVML.[Q]_	P47710	150322	121434	94656	115937	83988	233593	266039	96480	143753	126389	40956		42802	40890	46039					
[R].RPAIAINNPY.[V]_	P07498	150307	171872	222931	80095	45695	288379	371963	73113	214392	300334			28330							
[D].PNHFRPAGLPEKY.[-]_	P0DJI9; P0DJI8	150162	173679	193891	89604	80844	45092	50967		50294	32304										
[V].SSTYDLMSSAYLSTKDQYPYLK.[S]_	Q99541	149347	295347	32863		10901	117945	100752	289465	74744	43070	821492	1079740	1038670	728610	460175	101399	96428	66132		18616
[R].SPAEPEGGPASEGAARPLPR.[F]_	P09603	149304	93325	91721	609780	204590	62813	50610		89219	11586										
[V].DDTQFVRFDNDAASPR.[M]_	P13747	149276	92997	165698	322827	83385															
[M].VPRAPWMEQEGSEYWDRET.[R]_	P13747	149161	153410	159372	59692	52565															
[I].PFFDPQIPKLTDLENLHLPLP.[L]_	P05814	148541	522001	559393	42439	1304378	3822577	3701934	5209574	4414816	6409297	1977904	1415606	2012293	1928001	1975558					
[L].WSVPQPKVLPIPQQVVPYP.[Q]_	P05814	148053	140201	130400	334259	414115	102576	100482	169010	108381	322409	157640	171363	180849	195634	347211	83701	88244	99192	133615	83944
[P].LWSVPQPKVLPIPQQVVPYP.[Q]_	P05814	147622	159711	251291	107081	990641	222114	209046	258162	289231	605780	100370	131658	178292	265466	501257	27879	33359			52789
[R].GRATLVQDGIAKGR.[V]_	Q13410	145601	130634	114022	1113372	435224	99998	101257	161350	374685	215290	37422	60874	47128	73622	52704		20054	14595		

Quantified modified peptide sequence	Protein ID	A_-80oC	A_-20oC_120h	A_4oC_6h	A_RT_6h	A_RT_24h	B_-80oC	B_-20oC_120h	B_4oC_6h	B_RT_6h	B_RT_24h	C_-80oC	C_-20oC_120h	C_4oC_6h	C_RT_6h	C_RT_24h	D_-80oC	D_-20oC_120h	D_4oC_6h	D_RT_6h	D_RT_24h
[P].IPQQVVPYPQRAVPVQAL.[L]_	P05814	145418	162743	156568	87620	35656	272896	243751	323153	140986	91900										
[E].DVGSNKGAIIGL.[M]_	P05067	145097	129995	87723	177319	29185															
[T].DLENLHLPLPLLQPLM.[Q]_	P05814	144670	161055	157881	86917	931113	1378406	1289035	488947	1643618	3148273	998413	913806	1200722	2214759	3267043	30619	35884	26899	43332	240663
[D].DTQFVRFDNDAASPR.[M]_	P13747	144583	148428	114711	101205	17299															
[K].SPTIPFFDPQIPKLTD.[L]_	P05814	144146	153376	177014	221817	599704	3079366	2626703	1562402	2812321	3971356	875636	822587	1027647	1998668	6723531	40444	37577	32990	79682	445859
[I].DSLLENDRQQTHMLDVMQDHFS.[R]_	P10909	144097	81974	93082		62142	283912	289392	323260	205768	82419	276568	251835	245219	204626	94943					
[M].PVLKSPTIPFFDPQIP.[K]_	P05814	144093	125119	165118		201648	5235215	4704766	2600006	2777013	4005963	1184406	1297844	1382822	1450816	1884408		36796		35524	23863
[V].SPAVLVHRDGREQE.[A]_	Q13410	143756	112250	110644	85483	66248	275925	235801	172460	250388	99031	43777	45189	42417			36534	48626	31282		24789
[S].PTIPFFDPQIPK.[L]_	P05814	143542	114382	94853		370512	15003417	12748353	3388298	27475721	36082581	146301	115500	125528	511342	504529					
[A].IPVAQDLNAPSDWDSRGKDS.[Y]_	P10451	142988	107471	126866		12781	3137145	3064461	2254050	1216290	775874	7937805	7451340	7363247	6266158	5313481		26733	33305		
[V].LPVPQPEIMEVPKAKDTVYTK.[G]_	P05814	142664	136793	126723	138342	540248	388968	284224	2299538	730048	408885	280133	197697	258913	310848	495922					47485
[Y].VPFPPFSDISNPTAHENYEKNN.[V]_	P47710	142299	140072	164668	84790	41137	151794	117527	95516	81349	58656	52428	62808	57286	57700	28275					
[R].ETIESLSSSEESITEY.[K]_	P05814	141567	117831	139069	503076	616720	258631	229789	268840	485597	598089	76680	87534	78692	141033	161513					
[H].LPLPLLQPLMQQVPQPIPQT.[L]_	P05814	141157	153686	154741		45148	335587	318879	392179	112569	224630										
[A].SVDSGSSEEQGGSSRALVSTL.[V]_	P01833	140670	118523	130283	185826	36522											27319	17066		20970	18928
[A].LPPQPLWSVPQPKVLPIPQQVVPYPQRAVP.[V]_	P05814	140669	133657	187075	228588	54030						76609	76003	84259		77293					
[G].AQPILSKLEPQIA.[S]_	O60664	139909	144248	55395	111170	41630				46345	52342	25448	59296	63361		17942	98030	101198	117082	52452	29859
[L].PIPQQVVPYPQRAVPVQA.[L]_	P05814	139446	133904	151987	129690	327360	638548	539234	1555137	557226	658173	757905	865654	893028	1059025	1978866	138844	169444	166032	234477	418902
[R].DGREQEAEQMPEY.[R]_1xOxidation [M10]	Q13410	139353	114061	195527	546321	113482	126948	248895	402673	183670	72109						22820	27233	25715	77911	50488
[K].DKWDELK.[E]_	O00592	139340	127311	93794	164109	17102	73529	66783	79585	37330	14680						70485	91209	51248	36777	24342
[A].AKLGAVYTEGGFVEGVNK.[K]_	P19835	139085	102635	136412	199161	279532	1247469	1073121	1666760	1659220	1321867	151024	138182	176271	224802	202126	19225	72982	32234		23577
[C].EVWTQRLHGGSAPLPQDR.[G]_	Q92673	138159	130083	122522		85159	129873	137125	117780	117515	83068	94098	93649	84244	78076	36534	98244	160108	105638	46053	82211
[S].AGNGGSSLSYTNPAVAATSANL.[-]_	P15941	138096	119502	94941	96981							33439	34999	36987	24518	14153	40674	40160	34702	29145	
[D].SGEGDFLAEGGGVR.[G]_	P02671	138030	112794	95127	130630		94569	101400	85534	35583		58544	50334	52224	47550		145273	141649	165957	123619	53166
[G].DSVDIFK.[G]_	P19835	137860	108388	142211	1238263	349978	454365	429246	360217	1382329	1245309	113188	120029	133319	178820	87461					
[Q].ADGSRASVDSGSSEEQGGSSRA.[L]_	P01833	137742	105467	103030	562652	40788	65555	110526	127108	54884	19804	51736	56252	94120	99989	77251	49024	49756	58282	48384	28675
[P].IPLESREEYMNGMN.[R]_	P47710	137395	112243	108885	77170	93062	860393	1136449	590580	673479	908479	767077	784410	858712	504520	332398					
[R].TYYANPAVVRPHAQIPQ.[R]_	P07498	136826	183208	382757	119641	151265	474092	621762	391874	354620	649365	242318	273834	253257	157707	53187	35012	45200	46615		
[D].DEEENKLVKKI.[K]_	Q16181	136823	120276	96531	150228	41257											41824	38794	52613		17035
[K].VLPIPQQVVPYPQRAVPV.[Q]_	P05814	136713	133003	189952	368972	463432	440302	420552	379535	899070	1168533	106986	133582	157013	328584	1271710	38506	31680	29427	99028	120739
[Q].ELLLNPTHQIYPVTQ.[P]_	P05814	136524	100605	103287	277813	759685	1198184	1006735	359609	1037958	2156345	501450	450114	551435	1471593	4211729					
[Q].DAGIKTITMLDEQK.[E]_	O00161	135741	107927	97307		24405	57724	59102		91840	486847										
[E].DSLADQAANKWGR.[S]_	P0DJI9	135562	112462	124286																	
[T].KGRVMPVLK.[S]_	P05814	135100	154089	148732	229987	340332	304723	262308	349582	634642	216929	132781	147188	147421	250227	230768					
[S].TDRSPYEKVSAGNGGSSLSYTNP.[A]_	P15941	135003	114570	86649	54422																
[L].LQPLMQQVPQPIPQT.[L]_	P05814	134719	179023	190515	458394	286780	133083	141711	110116	183545	293775	49708	62764	68341	194363	193160	35183	42848	58695	79973	136344
[F].PVSQDQERE.[K]_	Q8NFU4	134381	88381	79580	93907																
[A].NPAVVRPHAQIPQRQ.[Y]_	P07498	134152	156929	127662		62704						124345	142419	117116	122106	137791					
[A].DTKELVSSKVS.[G]_	O60664	133865	108357	113660	135459	17258						108220	113645	130421	94871	64681	15287	19744	21545		
[P].EIMEVPKAKDTVYT.[K]_	P05814	133455	192281	143770	390519	1155064	380922	453238	447755	930805	3070023	47731	71865	68222	79275	94494					
[K].SGACEDHSMAQAGEVVL.[K]_1xOxidation [M9]	Q04656	133235	99403	102303	230808	36047															
[I].PFFDPQIPKLTDLENLHLP.[L]_	P05814	132955	297605	265353		112408															
[Y].VPPSSTDRSPYEKVSAGNGGSSLS.[Y]_	P15941	132641	133550	140654	126672	38175															
[F].DPQIPKL.[T]_	P05814	132624	119810	109886	255296	179330	530665	556995	250394	405527	712304	629718	690646	776062	1023229	1987233					
[V].MPVLKSPTIPFFDPQIPKL.[T]_	P05814	132598				181621	1653058	1572599	1109279	1096094	735956	2314028	2307266	2314090	1859914	1673598					
[N].QLQLQAAHAQEQIR.[R]_	P47710	132554	155648	130795		449873	448572	405433	388522	587520	1011574	84264	80830	72714	78586	77327					
[R].RNFPIPIPESAPTTPLPSEK.[-]_	Q8NFU4	132536	148789	104524	90872	48697															
[R].DTGAALLAESRAEKN.[R]_	O00391	132437	96148	75007	46838	9991						160155	158652	144047	178969	78933	36491	47196	28759		
[L].NQELLLNPTHQIYPVTQP.[L]_	P05814	132395	113520	73391		64850	128320	126202		80931	117434	163713	150147	179901	220795	455670					
[R].DVSLAKADAAPDEKVLDSGFREIEN.[K]_	P01833	132230	115413	147559		60164	89237	74173	218843	52276	26351										
[R].DADTLHSKLIPTQP.[S]_	Q13410	132209	110542	99477	84419	22175	87638	79341	82569	35738	26746	54967	65733	66709	54247		26074	26402	23085		
[K].EAKKVEGFDLV.[Q]_	Q99541	132124	116114	52111	91600	34269						316603	330816	125371	177851	122778	44453	60750	39540	37638	19782
[K].RLDINTNTYTSQDLK.[S]_	Q9H173	132075	110089	108822	96189	215653	374551	449316	469648	598584	348783										
[L].DDILEKLKINEYGQIH.[R]_	O15232	130759	134262	91267		65819	75124	76832	72464	52890	42829										
[M].YSGGGSALAGGAGGGAGRKEDWNEIDPIK.[K]_	Q10471	129618	124589	108545		33536	82410	61400	76080	32239								23245			10635
[K].YPDAVATWLNPDPSQK.[Q]_	P10451	128716	140863	132925	276284	193970	382162	366739	153425	430164	1067786	3975320	3955311	4858396	4953522	5273928					
[A].IQDPRLFAEEKAVADTRDQADGSR.[A]_	P01833	128653	122838	92576		43829	309879	234367	528736	268382	152570	39102	27771		15428						
[L].AGRDLSRLPQLVGVSTPLQGGSNSAAAIGQSSGEL.[R]_	P15291	128458	88806	109786																	
[A].IQLTYNPDESSKPNMIDAATLK.[S]_	P02679	128036	124093	86880	114527	122928											39717	38532	39453		22636
[L].DPDTAHPHLF.[L]_	Q13410	127151	127210	95519	241606	28679	81502	89394	130867	46566	44422	922442	969088	1270868	885435	442926	28703	44344	55751		14648
[A].PTKALENPQPHPGWQGTLK.[A]_	P19835	126778	36702	76184		54900	476449	450330	309539	562475	411418	122916	145978	53888	111347	87150					
[S].KEIPLSPMGEDSAPRDADTLHSKLIPTQPSQGAP.[-]_	Q13410	126423	145290	113398		44074	134083	138738	277721	127480											
[T].DPEKLDQIRQ.[R]_	P17987	125979	92258	81653	199759	27045	30833	39950		26344	13910	46090	45440	42861	41225	35797	41524	41154	34876	26774	17242
[L].PIPQQVVPYPQRAV.[P]_	P05814	125778	111418	65849	80726	52312	944610	717160	3075030	392797	399742	265569	336190	304979	493919	467783	99951	53598	143754	182671	89321
[V].EPIPYGFLPQNILP.[L]_	P05814	125689	124697	127729		61439	293534	286693	124488	175207	148708	152590	148302	174615	158708	139608		30935			74760
[Q].PLAPVHNPIS.[V]_	P05814	125439	113255	99220		27799	34012					106664	101759	129278	177670	92065					
[M].PRNQGFSEPEYSAEYSAEYS.[V]_	O60266	125347	129322	78923		37117						76769	69739	80994	57303	36870					
[V].PKAKDTVYT.[K]_	P05814	125223	79836	41345	219460	93210	170782	153969	115318	97701	129373	87349	95518	69456	112607	143287	21247	37445	28664		22179
[R].RPDIQYPDATDEDIT.[S]_	P10451	124457	82826	89601	68598	24280	1273148	1413629	958473	455617	371131	1382254	1356867	1193865	944243	491860	112386	108274	125764	128262	110226
[G].DPQELNGITR.[A]_	P49327	123198	66968	73952	120688	12826	39614	53314		41177	41606	79673	83335	78343	83189	49097	97462	111559	104208	84785	51676
[K].DTVYTKGRVMPV.[L]_	P05814	123084	102843	101453	920175	735721	528654	695136	525044	1283651	1448198	24616	34227	17528	38200	89256					
[R].TYYANPAVVRP.[H]_	P07498	122640	96377	97434	147586	34016	169173	169141	81523	137272	60934	105554	141615	143098	121238	146842	88239	141244	94664	126736	51419
[N].DEELNKLLGKV.[T]_	P20671	122419	114369	107986													22838	24687			
[V].DSGSSEEQGGSSRALVS.[T]_	P01833	122322	89660	71003	275959	25384	254291	248897	105365	119964	50628	81605	84861	86818	67996	27250	23510	29588	27611	18481	9799

Quantified modified peptide sequence	Protein ID	A_-80oC	A_-20oC_120h	A_4oC_6h	A_RT_6h	A_RT_24h	B_-80oC	B_-20oC_120h	B_4oC_6h	B_RT_6h	B_RT_24h	C_-80oC	C_-20oC_120h	C_4oC_6h	C_RT_6h	C_RT_24h	D_-80oC	D_-20oC_120h	D_4oC_6h	D_RT_6h	D_RT_24h
[R].EIENKAIQDPRLFAEEKAVADT.[R]_	P01833	122300	134081	111823		83855	155108	127203	198086	116275	72416										
[Y].NPDESSKPNMIDAATL.[K]_	P02679	122132	77923	94220	155806	85492	73666	76333	61711	69380	103303										
[L].NQELLLNPTHQIYP.[V]_	P05814	121984	124398	126447	85620	39753	145710	126132	60561	66121	84398	186987	196867	178759	175657	202271	24353	31368	20099	38991	116962
[S].SEEQGGSSRALVSTL.[V]_	P01833	121304	78421	72059	73505	25267															
[V].PPSSTDRSPYEKVSAGNGGSSLSY.[T]_	P15941	120978	115577	106682		36819											36228	24347	29707		
[M].DPSKPSSNVAGVV.[I]_	P22897	120172	126848	86491	239117	40969											23320	26684	22553		15237
[K].VLPIPQQVVPYPQRAV.[P]_	P05814	120126	84724	48684	87660	55193	2021143	1475843	837252	1376468	1577529	363860	366843	401638	827664	939885					
[M].RPVSDQVQIKV.[T]_	Q96A33	119669	118743	102090	88422												51223	68529	53448	43163	21317
[K].TTFDVYTESWAQDPSQENK.[K]_	P19835	119372	164032	196334	332021	266043	517727	552507	532685	1607734	1797842	82035	88102	120354	224986	115827					
[N].ILPLAQPAVVLPVPQPEIMEVPKAKDTVY.[T]_	P05814	118991	139576	89212	223132	41098															
[V].DPQPSVVTR.[V]_	Q99541	118701	94001	75665	368126	23836	41364	40783	51047	26398		94908	133633	152179	118152	49331					
[Q].DLNAPSDWDSRGKDSYETSQL.[D]_	P10451	118524	109051	95906	69222	35116	56761	60198	73410	37520	27017	211667	182495	218267	157489	89021					
[T].DEDITSHM.[E]_	P10451	118522	81856	72324	296668	51372	396888	475031	224718	305987	246182										
[T].LALPPQPLWSVPQPK.[V]_	P05814	118048	152655	182961	377128	1183275	865407	840705	448630	2662026	3825782										
[A].QPAVVLPVPQPEIMEVPK.[A]_1xOxidation [M14]	P05814	118030	86843	167324	109723	34154	795258	967187	813806	388042	237171	291355	361573	405941	532513	408710		17326		42736	20381
[A].SVDSGSSEEQGGSSRALVST.[L]_	P01833	117613	97720	77523	141287		81251	125432	63355	47604	28242	125190	114679	135030	101572	46676	322297	346990	356732	344036	197279
[A].IPVAQDLNAPSDWDSR.[G]_	P10451	117360	84499	141940	171479	178539	1151842	1267841	852849	2809483	4005893	349898	337332	375110	401764	279577	17328	16815	13750		14552
[N].HFRPAGLPEKY.[-]_	P0DJI9; P0DJI8	116476	139752	147343	73193	64781															
[S].ESSEPIPLESREEYMNGMN.[R]_	P47710	116221	156921	82469	143962	94134	1350838	1415856	785092	1420697	1642867	1021166	967326	1150953	1167000	788942	52480	62831	64315		36333
[R].AVPVQALLLNQELLLNPTHQIYPVTQPLAPVH.[N]_	P05814	116177		360870			657613	491524	474736	630476	592488	728487	781548	563478	672680	793783					
[N].DLEDKNSPFYYDWHSLQVGG.[L]_	Q14802	115869	92108			16533															
[S].DEFKVETSNKVLD.[Y]_	O14672	115647	70023	72808	69418	38698						35508	37786	30282	31352		32632	24883	20844		16977
[L].NPDPSQKQNLLAPQN.[A]_	P10451	115586	101660	92673	133071	26055						129208	122554	108624	110022	63879					
[L].ERGEKLDDLVSKSEVLGTQ.[S]_	O15498	114989	107923	92972								99973		73296	71485	46988					
[A].IAINNPYVPR.[T]_	P07498	114665	122996	125762	121097			21760	78932	36743	34287										
[E].SREEYMNGMN.[R]_	P47710	114063	76171	45491		68528	1894541	2172642	1802801	1887080	1310583	1458128	1293368	917535	1421727	715217	186539	348569	226962	92718	36361
[S].NPTAHENYEKNNVMLQW.[-]_	P47710	113957	139636	123064	113968	279207	323374	226059	61850	335382	541263	66212	77648	199178	130042	267149					
[E].DQQQGEDEHQDKIYPSFQ.[P]_	P05814	113914	93305	73122	141780	32217															
[A].VADTRDQADGSRASVDSGSSEEQGGSSRAL.[V]_	P01833	113786	104919	109677										37781							
[K].DKWDELKEAGVSDMK.[L]_	O00592	113779	86126	80891		14846	171392	105602	150729	85617	54704										
[K].LPLRYPERLQ.[N]_	P47710	112955	72979	50082	49122	142080	621086	557805	131686	418744	294631		43170	35810	33295	33157					
[L].NEYNQLQLQAAHAQEQI.[R]_	P47710	112893	70771	141663	276378	491859	454966	446881	281836	1434335	3615239	127509	106290	99919	106882	88908					
[S].DIDDAVRKL.[-]_	P53999	112878	88523	84758	74618	12869						41676		34130	30821		39351	40955	37012		
[A].DTLHSKLIPTQPS.[Q]_	Q13410	112602	112328	89636	57454	23900	94484	94745		36171											
[L].FPNTSDPQKENRNTG.[I]_	Q8N387	112423	89077	72261	98440	10230	301259	383557	232528	146649	32091	31129	30706	43316	30252	14571	43998	63191	48968	38846	24311
[Q].LQAAHAQEQIR.[R]_	P47710	112117	120500	102960		409728	417903	422603	375197	513450	941229	58087	66232	73706	64333	70915					
[V].DFETDVLFLVPTEIALAQH.[R]_	P19835	111682	114200	105116		61827	1035078	1357685	855736	897259	715784	33270	21422	41054	29403						
[V].PQPEIMEVPKAKDTVYTK.[G]_	P05814	111497	181461	111386		1223712	1044303	982836	4946006	1886524	866561	910646	849130	726152	852556	960256		20304	32577		15078
[M].PVTVTRTTITTTTT.[S]_	Q96S97	111483	92657	80223								82776	86913	88733	67234	36439		19211	22764		
[E].DITSHMESEELNGAY.[K]_	P10451	111304	26534	25715	85443	55449	312588	338706		179533	362905	111672	106924	110891	103750	82724					
[K].SPTIPFFDPQIPKLTDLENLHLPLPLLQPLMQ.[Q]_	P05814	111214	584097	86710		410503	1914056	1546657	2131779	1320179	1145013	5205791	4561689	4073710	4328326	3032710	32914	32875	46730	56771	56884
[R].HPDEAAFFDTASTGK.[T]_	P02671	111109	111676	119742	158287	87430	157336	149639	90878	223364	303997	17760		21687	36961	25775					
[V].VPYPQRAVPVQALLLNQE.[L]_	P05814	110457	91302	62189		52896	64245	41267	70557		53153	58926	87017	72428	69900	163878		35794			68332
[A].IQDPRLFAEEKAVADTR.[D]_	P01833	110261	137370	180080		326097	411160	435412	519065	693872	303692	76887	163545	70672	140278	72767	230340	304510	214444	101906	83966
[K].VKHEDQQQGEDEHQDKIYP.[S]_	P05814	110102	166184	165950	75884	946946	561296	707166	1089872	1487762	1309196	76119	78087	76347	165956	334055	47227	53682	46175		88115
[V].MPVLKSPTIPFFD.[P]_	P05814	109846	105115	151935	92669	599824															
[P].LAQPAVVLPVPQPEIMEVPKAKDTV.[Y]_	P05814	109289	151909			38863	117847	185256		101440	51987										
[P].VLKSPTIPFFDPQIP.[K]_	P05814	109058		229661	135255	469723	754590	839936	657997	638207	810084	110195	137072	191314	181425	249925					
[Q].ELLLNPTHQIYPVTQPLAPVH.[N]_	P05814	108828		84937		34193						271297	261251	297508	285160	293950					
[A].TLVQDGIAKGRVA.[L]_	Q13410	108192	144936	108498		35814	47298	43880		33042		245326	276253	252508	279250	57490	400328	514209	384883	314530	176569
[T].DEIKDTR.[N]_	P47710	108032	38645	85789	1089328	255572	101311	91631	255835	248465	276956	55481	43424	34029	79452	90628					
[Y].NKYPDAVATWLNPDPSQKQNLLAPQN.[A]_	P10451	108005	179210	178142		91370						1074934	1014672	1031603	951223	749163	56861	83273	52200	31086	52691
[A].LPIIQKLEPQI.[A]_	Q99541	107908	114827	91109	67662	24592	96896	92168		35621	30165	181217	188332	219045	147019	96035					
[T].EGGVLKKVI.[R]_	P24821	107772	84814	88780	280023	87838															
[D].DPDAPLQPVTPLQLFEG.[R]_	P0C0L4	107767	78830	95454	38811	27795															
[Q].NPSESSEPIPLESREE.[Y]_	P47710	107373	71370	97433	186048	25619	439980	539417	421390	311312	135810	656203	741292	741124	708033	275748	34396	29332	31776		16031
[I].PQRQYLPNSHPPT.[V]_	P07498	107077	89983	70932	106794	28131	44531	46340		29936	18891										
[A].DGPERVTVIANAQDLS.[K]_	Q13410	107040	109962	160043	195749	127021	136749	183828	104933	83147	219826	330869	325408	413347	386391	252129					
[V].EISIPASSLPRLTPWIV.[A]_	Q13410	106554	125797	109067				324168		215276	150464						85777	80216	73567	96056	91126
[S].SGVENALTKSELL.[V]_	Q99541	106146	110024	89773	91188		96845		69064			1720712	1866370	1279926	1518758	779078	193766	188836	160276	147385	54646
[P].FFDPQIPK.[L]_	P05814	106119	121638	108509	660067	257585	1540883	1491365	601500	1047019	1728603	146750	150960	130826	298491	286084					
[S].DSDELASGFFVFPYPYPFRPLPP.[I]_	Q8NFU4	105798	194318																		
[Y].APSNDIYGGEMHVRPML.[S]_	Q16625	105796	104577									351376	312791	308282	195212	133665	19141	25970	30190		
[Y].VPNSYPYYGTNLYQRRPAIA.[I]_	P07498	105766	172079	129051		67649						278513	260352	200015	147735	169005	26774	34588	29610	25680	35334
[R].GRATLVQDGIAKGRVAL.[R]_	Q13410	105692	105586	73512		103515	64503	99090	215734	181924	278844	63282	92838	83970	54207	30422	26047	60521	16354		20514
[A].DQAANEWGRSGKDPNH.[F]_	P0DJI8	104785	88055	96335	106869	12893	29341	43020	48432	20638											
[E].SITEYKQKVEK.[V]_	P05814	104489	142563	110713		176775	736049	924072	728954	905158	513768	619539	644153	473547	489373	426735					
[A].LPPQPLWSVPQPKVLPIPQQVVPYPQRAVPVQ.[A]_	P05814	104453	127785	153731		126586	613988	548930	755029	457521	253985	184575	231875	178017		280561					
[P].MGEDSAPRDADTLH.[S]_	Q13410	103827	86060	45684		30157	60246	57892	152853	50382	36422	259187	253372	229509	180038	103588	25382	26742	34841		13413
[E].DEHQDKIYPSF.[Q]_	P05814	103546	72935	85208	116479	59387	1022295	893778	219107	666452	404697	325177	276351	253345	293450	240794	24289	43041	28650		17393
[G].FLPQNILPLAQPAVVLPVPQPEIMEVPK.[A]_	P05814	103380	91588	127329		31667	389380	358445	185423	263234	199741	262018	218000	265164	225809	182427					
[F].RRPDIQYPDATDEDITSHMESEELNGAY.[K]_	P10451	103139	130950	108135			394368	450262	889413	136068	84373	198286	250455	183630	164466	124930					
[K].SPTIPFFDPQIPKLTDLENLHLPLPL.[L]_	P05814	103019	296947	92140		175947	382587	339672	202544	259185	136972	528355	531280	597105	492527	446439					32006
[P].YPYPFRPLPPIP.[F]_	Q8NFU4	102841	95069	87531		45782											54440	53275	46643	81010	62374

Quantified modified peptide sequence	Protein ID	A_-80oC	A_-20oC_120h	A_4oC_6h	A_RT_6h	A_RT_24h	B_-80oC	B_-20oC_120h	B_4oC_6h	B_RT_6h	B_RT_24h	C_-80oC	C_-20oC_120h	C_4oC_6h	C_RT_6h	C_RT_24h	D_-80oC	D_-20oC_120h	D_4oC_6h	D_RT_6h	D_RT_24h
[K].IVRGDQPAASGDSDDDEPPPLPRL.[K]_	O00264	101616	82112	79784	63953	17998	35312	56498	95671												
[V].DWGKIQRPP.[E]_	Q16851	101214	110970	83291	126393	27280						45669	43643	45871	41926	23685	39095	48083	41935	31885	20115
[Y].DESGPSIVHR.[K]_	P60709	101197	68596	60748	262740	25793						73971	109630	101259	104824	48379	33482	44664	35088	30250	15213
[K].SPTIPFFDPQIPKL.[T]_	P05814	100933				218067	3712598	2564780	1614865	1740528	2202620	899531	798846	912575	1889066	3601204		43661	34481		189639
[K].APGRVLVHTSVSPSPDNLR.[R]_	Q6WN34	100690	142545	133558		147512	610803	564806	698915	627711	503925	35951		30199	39512	30528	15332	22376	22846		31068
[K].DDPDAPLQPVTPLQ.[L]_	P0C0L4	100629	82582	64386	256791	73976						103677	89924	97267	88342	43114					
[L].ALPPQPLWSVPQPK.[V]_	P05814	100095	109414	126899	261744	302725	801017	1263680	1415367	2089664	3523168	76296	56973	93151	98116	99207					
[E].DEHQDKIYPSFQPQP.[L]_	P05814	99125	99105	84326	174662	169068	771040	737713	383881	610130	822464	187264	149460	175509	144225	144056					
[R].AQGGGALRAPSQGSGAAARS.[-]_	Q9GZN4	98826	79217	107300			21728	38245	49905	16267		33759	66957	65707	41912	24240	63346	90283	82815	54136	27057
[K].SPTIPFFDPQIPKLTDL.[E]_	P05814	98750	105991	85485		184796	530984	454414	294940	380184	486942	196428	183300	232646	276780	850272					50529
[A].VADTRDQADGSRASVDSGSSEEQGGSSR.[A]_	P01833	98604	57447	43306	248958		149052	183256	286321	76975	20358	64818	80073	79202	59539	36898	30587	26220	25298		29974
[L].DDQSAETHSHKQSRLY.[K]_	P10451	98417	135050	101016	105671	240430	229128	213164	139171	226702	119838	2025565	2597813	2021867	1777454	1196486					
[V].MPVLKSP.[T]_	P05814	98179	103108	81442	245574	176992	111359	130867		124605	115517	233645	270435	310293	344097	428485					
[R].LFAEEKAVADTR.[D]_	P01833	97985	61899	33775			49748	37414		31492	51768	52444	92114	66577	69707		256839	432856	295962	77439	9899
[S].DISNPTAHENYEKNNVM.[L]_	P47710	97581	85057	86696	88772	100407	59363	93714	53926	83005	69886										
[R].YPERLQNPSESSEPIPL.[E]_	P47710	96962	71810	79306	113633	136792	506426	403746	318312	515271	491327	50385	57013	62138	70407	93544	17918	20860	17346	26707	50226
[K].VLPIPQQVVPYPQRAVPVQALLLNQE.[L]_	P05814	96825	161228	140271	92761	966599	3592270	3376696	3439213	4063255	7218569	635243	800990	773558	1010778	1626520					
[E].IPLSPMGEDSAPRDADTLHS.[K]_	Q13410	96812	89121	61505	68150	26042						40007	39720	21966	23537	25936					
[V].PQPEIMEVPKAKDTVYT.[K]_	P05814	96614	64025	41829	63589	209847	544613	557936	1006089	448538	455343	23984	21538	23599	27259	63590					
[S].VPQPKVLPIPQQVVPYPQR.[A]_	P05814	95464	99847	83173		41708	233677	174889	357121	115067	133152	681716	907755	689038	576085	420291					
[P].DAVATWLNPDPSQKQNLLAPQN.[A]_	P10451	95375	70361	97133		33957						52453	38278	46704	48380	24511					
[W].DELKEAGVSD.[M]_	O00592	95336	81122	44349	231383	26116															
[Q].LQAAHAQEQIRR.[M]_	P47710	95235	183484	146667		1007093	723840	745965	749386	2025852	2652174	160076	130969	170649	334119	468485					
[P].DVDIKGPKV.[D]_	Q09666	94888	64084	58433	132321	21986						56938	56878	48957	41727	18353					
[Y].DGEEIATRGNVIV.[V]_	P19835	94367	71513	39517	67793																
[I].PASSLPRLTPWI.[V]_	Q13410	94146		71097																	
[G].KPPPPPPPP.[A]_	A0JLT2	93715	82334	67558	196504	31702	34878	43738		24296											
[V].EISIPASSLPRLTPWI.[V]_	Q13410	93657	94985														59205	77694	67629	58983	60839
[A].PDEKVLDSGFREIENK.[A]_	P01833	93360	98783	76145		48970	198076	81765	77941	197729	303019	61289	69394			25934					
[V].VPYPQRAVPVQ.[A]_	P05814	93249	90706	153986	291853	84804	530475	409623	367297	352201	323235	264060	337256	350646	668616	1022300	26306	28634	38137	59553	77567
[L].STKDQYPYLK.[S]_	Q99541	92844	72323	21517	121305	31434				49921		2082366	2397787	1294249	1674994	814619	111350	178630	150975	38878	29255
[Q].LQLQAAHAQEQIRR.[M]_	P47710	92736	281920	273765	537581	681007	564772	530872	659088	1431200	1891902	130301	136026	122321	162316	280559					
[K].VEISIPASSLPRLTPWIVAVAV.[I]_	Q13410	92682	124269	89885	78771	41466	187918	161484	229160	62866	72828						263285	216147	288045	291607	143758
[E].RLQNPSESSEPIPLESREEYMNGMN.[R]_1xOxidation [M]	P47710	92644		360270		67015	7603884	8540400	14116044	4763353	3384131	1013343	1594991	1326994	1136189	2587892	190284	200536	210132	1257123	718619
[S].PTIPFFDPQIPKL.[T]_	P05814	92579				79677	2995893	2249524	1290516	1773890	2528465	561839	608994	692543	1207774	1731865		59867	66616	62520	210150
[L].TYNPDESSKPNMIDAATLK.[S]_	P02679	92513	49718	72229			45457	57447	49107								27049	19210	18686		13417
[V].LPVPQPEIMEVPKAKD.[T]_	P05814	92438	97602	79619																	
[R].EGTINVHDVETQFNQYK.[T]_	P15941	92397										51602	50578	41377	25018		106597	125699	100926	56116	23182
[L].SPMGEDSAPR.[D]_	Q13410	92349	66640	47730	187260	16216	63533	62974	31627	37548	6087	103159	129211	143484	103177	38810	36777	32301	26541	16033	5944
[L].FAEEKAVADTRDQADGSRASVDSGSSEEQGGSSRA.[L]_	P01833	92337	113988	143352	100565	58943	40764	89039	249949	58829	18393	68678	88788	50863	50071	41032	27543	21903	26624	58353	
[A].DSGSSEEKQLYNKYPDAVAT.[W]_	P10451	92184	90826	82113	86015	30003						154152	158037	171546	130450	95862					
[R].NILREKQTDEIK.[D]_	P47710	91762	113606	129084		56174	208773	208396	324954	146980		88119	106571	106299	140503	50089		20064	35142		18175
[I].PLSPMGEDSAPRDADTLHSKLIPTQPSQGAP.[-]_	Q13410	91548	101570	55326			70112	99650	128789	35729	33669										
[A].EEKAVADTR.[D]_	P01833	91497	75496	79853		66927	88013	89579		103310	119050	52635	50516	49545	29725						
[I].THRIHWESASLLR.[S]_	P01024	91497	402365	224100		29575															
[I].PFAAPTKALENPQPHPGWQGT.[L]_	P19835	91477	86495	82317		35483	64126	68407	40501	47643	128913										
[E].DQQQGEDEHQDKIYPSFQP.[Q]_	P05814	91354	67643	42295	84493	15265															
[L].HSKLIPTQPSQGAP.[-]_	Q13410	91136	154991	79706	73013	32660						127525	147645	93771	87812	56853	38440	48388	31351		
[S].IPASSLPRLT.[P]_	Q13410	91049	64109	36250		19069	590355	416288	137168	204412	82955	179938	219089	182981	129430	31682	81517	120324	90019	80033	21508
[R].NPDEEGLFTVAASVIIR.[D]_	Q13410	91042		125043													89826	68520	56296	127819	82656
[G].SSIQGLPTNVKDQVQQA.[R]_	O60664	90985	94848	66361								37585	48337	57906	32315		14801	17818			
[A].QGGGALRAPSQGSGAAARS.[-]_	Q9GZN4	90846	105486	61478	69354	8352	33072	28609		13667		18064	23847	22540	18924	11484					
[L].PLAQPAVVLPVPQPEIMEVPKAKDTVYTK.[G]_	P05814	90599	167330	165750	56815	493045	232476	320884	4892341	916726	541055	73785	68173	82783	63862	73757					
[V].DLQTRIME.[L]_	Q8NBJ4	90599	61113	50250	130319	14480															
[A].LPPQPLWSVPQPKVL.[P]_	P05814	90555	98957	92430	57997	177964	420387	410623	218184	241436	248976	240510	258957	278831	713106	1425632	35817	41830	31868	85375	194326
[R].YPERLQNPSESSEPIPLES.[R]_	P47710	90497	81588	75659	70730	32170	178427	173740	138023	128560	113471	248112	243274	239834	224123	133153					
[T].TEPLPEKTQESL.[-]_	P55327	90390	56750	25946			154450	111067	131104	67268		238905	245063	196193	196024	60063	36303	37220	37001		
[L].MQQVPQPIPQTLALPPQPLWSVPQPK.[V]_	P05814	90386		110621		105516	245223	245504	174203	393220	1401901										
[K].LPLRYPERL.[Q]_	P47710	90032	92576	99266	129531	160775	335238	284715		243471	203812										
[L].VYYLAGRDLSRLPQLVGVSTPLQGGSNSAAAIGQSSGELR.[T]_	P15291	89594				137332		215057													
[R].SVDALDDLTPPSTAESGSRSPTSNGGRSR.[A]_	Q86X29	89261	85001	85518		35499	47398	68517	111771	41105	23989										
[L].VGVSTPLQGGSNSAAAIGQSSGEL.[R]_	P15291	89025	70133	68332	81230	21838															
[L].QLQAAHAQEQIRR.[M]_	P47710	88810	175210	186880		999088	849921	817799	807140	2232178	2899574	20217		84392	180294	223317					
[K].VPPEWKALTDMPQM.[R]_	P02671	88763	102828	92086	156590	299843	96689	116217	71577	201576	207091										
[V].DSGSSEEQGGSSRAL.[V]_	P01833	88396	69076	59500	458830	43611	123246	98704	92818	78634	19057	67494	69609	78227	57799	35936	57113	47636	53129	46981	49394
[I].PKLTDLENLHLP.[L]_	P05814	88207	72408	55253		42180	69093	68284		55994	41593	213797	190696	164886	206801	164721					
[K].LGAVYTEGGFVEGVNKK.[L]_	P19835	87959	107444	132543	85825	83369	82760	85569	146652	210968	80863										
[L].SSSEESITEYKQK.[V]_	P05814	87877	57418	54401	68768	86190	450219	477839	386983	590238	224749	354592	286875	231311	433484	292861					
[V].MPVLKSPTIPFFDPQ.[I]_	P05814	87749						82785													
[L].DPDTAHPHLFLYEDSK.[S]_	Q13410	87413	119895	91834	56471	35776	121193	100947	98634	56120	44491	137921	115357	158827	115984	72773		20434	22681		
[E].IPLSPMGEDSAPR.[D]_1xOxidation [M6]	Q13410	87389	71124	107969	119176	22476	100263	124070	162731	45088	24936	85895	127221	90939	71093	74975	52764	56408	41020	117186	31342
[L].QPLMQQVPQPIPQTL.[A]_	P05814	87291	126057	103761	140163	130601	567456	505737	412479	321631	362414	232609	252162	225324	273369	305487					
[Y].DAAKRGPGGAWAAEVISNARENIQ.[R]_	P0DJI9	87071	130249	135552		29871															
[I].MEILRGDFSSANNR.[D]_	P02671	86922	152624	106183		91213	84940	85219	100642	167330	92039										

Quantified modified peptide sequence	Protein ID	A_-80oC	A_-20oC_120h	A_4oC_6h	A_RT_6h	A_RT_24h	B_-80oC	B_-20oC_120h	B_4oC_6h	B_RT_6h	B_RT_24h	C_-80oC	C_-20oC_120h	C_4oC_6h	C_RT_6h	C_RT_24h	D_-80oC	D_-20oC_120h	D_4oC_6h	D_RT_6h	D_RT_24h
[I].ESQDAGIKTITMLDEQK.[E]_	O00161	86759	54757	59190																	
[K].LTDLENLHLPLPLLQPLMQQVPQPIPQTL.[A]_	P05814	86133	179331	161476		157723	793718	732539	437129	632612	649547	673982	602955	749624	639107	1070613					
[V].PPSSTDRSPYEKVSA.[G]_	P15941	85880	78532	48549			85752	99063	93014	35386		45284	50575	32148	40193		25284	23414	22632		
[G].LPTNVKDQVQQ.[A]_	O60664	85627	73011	47697	107338	17448	42686	54495		21182	15272	56221	61033	67806	62550	34675					
[S].VDSGSSEEQGGSSRALVST.[L]_	P01833	85596	64267	89228	101141		27228	38040				58325	42911	82953	55105		195448	162542	187158	133487	84429
[K].AKDTVYTK.[G]_	P05814	85493		18143	350023	91701	35506	19176	57487	130542		19387	26379	17522	21839	21025					
[E].DQQQGEDEHQDKIYPSFQPQPLIY.[P]_	P05814	85315		40568		19782						62035	55589	61132	46258	21886					
[L].PNSHPPTV.[V]_	P07498	85287	72003	45825	181930	45845	31546	42906		25551	23909	71891	71159	79295	100060	73929					
[Q].PEIMEVPKAKDTVYTK.[G]_	P05814	85073	125701	108262	50652	453915	402402	391693	749810	605203	341879	424416	359353	351284	401696	563131					25065
[V].ENALTKSELL.[V]_	Q99541	84943	61604	52422	59892		46837					337374	407731	676666	259296	120871					
[S].HPSYHTDGFN.[-]_	Q9H3Z4	84857	66406	53303		23055	114041	131607	78574	71398	14254	66850	60834	68728	50927	31566					
[H].EDQQQGEDEHQDKIYP.[S]_	P05814	84825	56871	57110	887701	297159	233581	298665	134001	353384	393031	32677	30298	43447	52412	29467	154922	146436	159921	83958	98544
[K].VPPEWKALTDMPQ.[M]_	P02671	84611	102910	108707	103942	40874	82722	96086	71126	48307	34437										
[A].RETIESLSSSEESITEYKQ.[K]_	P05814	84497	67810		93124	81661	188298	248388	265874	356998	188360	50880	33193	82051	118762	194141			19105		89495
[E].YNQLQLQAAHAQEQIR.[R]_	P47710	84437	26158	42583	110159	618593	415413	377582	330724	666417	1341025	35433		50079	44158	58282					
[E].GGFVEGVNKK.[L]_	P19835	84385	52493	71556	636899	113030	330962	364626	178999	741883	286197	15710	20690	23096	76421	49487					
[V].SPAVLVHRDGREQEAEQMPEY.[R]_1xOxidation [M18]	Q13410	84302	45749	214177		35136	503173	625428	1399172	450600	183627										
[G].FPVSQDQER.[E]_	Q8NFU4	84289	86070	77368	291562																
[T].GRGAEDSLADQAANKWGR.[S]_	P0DJI9	84271	98631	68525																	
[I].SNPTAHENYEKNNVMLQW.[-]_	P47710	84242	78875	103513		25738	162519	112585	196457	113259	45976	17372	38526	46349	53259	51868	15724	34329	27875		118355
[S].TPLQGGSNSAAAIGQSSGELR.[T]_	P15291	84228	48679	51599																	
[K].FRISHELDSASSEVN.[-]_	P10451	83839	101086	92284	117396	93347	2090277	2213854	1543674	1484976	104896	2088544	1981022	1832171	2486073	1711250					
[I].YPFVEPIPYGFLPQNI.[L]_	P05814	83664	80313	107889		92242	556248	426490	125564	217702	180227	95678	99295	131331	234428	257367		22902	25929		41036
[A].VYTEGGFVEGVNK.[K]_	P19835	83481	75368	163255	123910	36989	164514	211018	125354	224545	137287										
[F].DPQIPKLTDLENLHLPLPLLQPLMQ.[Q]_	P05814	83325	197935	103352		45804	1997238	1771340	1204581	1247649	645125	11746596	10634961	11503769	8884624	6618287	50620	46648	33483		30803
[Q].PEIMEVPKAKDTVYTKGR.[V]_	P05814	83257	95243	133212		54626		40342	94484	33872		36542	30180		60565	89811					
[A].RPKLPLRYPERLQNPSE.[S]_	P47710	83133	70548	79923	390450	309168	2356168	1535351	2628776	2288067	1477171	89784	201542	62251	198241	499863					
[I].YPSFQPQPLIYPFVEPIPY.[G]_	P05814	82617		58899		56967	435078	387088		460995	585337		24470	60814	65019	50972					
[K].LPSDGKEVPF.[V]_	Q8N9U0	82460	46456	48728	98088	23737															
[F].AEEKAVADTRDQADGSR.[A]_	P01833	81813	50209	51638		9977	144899	224118	158283	91010	69194	93317	82708	51072	32661	14917	519316	614099	547624	222409	152325
[G].EDSAPRDADTLHSKLIPTQPSQGAP.[-]_	Q13410	81621	63592	102900		80280															
[P].QTLALPPQPLWSVPQPK.[V]_	P05814	81494	144193	252468	74327	377426	559989	711605	665783	1292440	960862										
[A].QIPQRQYLPNSHPPTVVR.[R]_	P07498	81203	175096	171859	65175	67474	130299	104811	84392	119275	55490	89167	106649	82904	92325	97853	23547	45993	31215		10481
[I].ESLSSSEESITEYKQK.[V]_	P05814	81096	76238	60334	93880	97713	280328	350434	252451	333347	251499	263726	303302	315802	350941	329610	27528	26560	23219		26850
[K].ALENPQPHPGWQGTLK.[A]_	P19835	81080	88310	72097	82488	60810	465956	420185	321502	788058	273512	235749	234246	247962	235257	108618					
[K].LTDLENLHLPLP.[L]_	P05814	81017	115082	132902		139561	893250	1014224	372598	806085	1024966	277347	300860	277269	255537	385835					
[N].QELLLNPTHQIYPVTQPLAPVHNP.[I]_	P05814	80966	74313	74800								45795	62933	55369	43112	25772					
[Y].YANPAVVRPHAQIPQRQYLP.[N]_	P07498	80645	110184	87514		62587	49950	43787	84964	41681	57144	124247	137513	103469	105528	116619	12741	23403		29878	24431
[S].SYSKQFTSSTSYNRGDSTFESK.[S]_	P02671	80616	65990	84930		21230		46026		77459	22362										
[Q].IYPVTQPLAPVHNPISV.[-]_	P05814	80357	104143	176846	172020	57879	429293	379962	214725	168934	151828	546891	614740	613859	518018	502765	120610	155146	135970	259831	438132
[F].RPKGAGSTTVKIVL.[K]_	Q6WN34	80199	116705	110806		340084	1194407	1205076	472016	1909332	1661172	50054	65278	33370	45132	64234					
[Q].NPSESSEPIPLESREEY.[M]_	P47710	79804	53213		66493	28979	196082	255193	218727	124863	85108	164850	163276	165307	172910	69262	29474	18733	18838		
[K].VEISIPASSLPRLTPWIVA.[V]_	Q13410	79557	78061														503906	564742	478231	583795	177398
[I].PQRQYLPNSHPPTV.[V]_	P07498	79539	90787	85460		34227	61823	76702		43567	20469	96583	100831	95872	103749	93862					
[P].FAAPTKALENPQPHPGWQGTLK.[A]_	P19835	79532	93021	82340		135047	721180	692462	706372	847232	774030	116477	105757	116887	144277	113416					
[Y].ANPAVVRPHAQIPQR.[Q]_	P07498	79312	162367	176246		152866	66346	79574	299493	172824	291330	126532	192684	275075	160592	142986					
[R].ATLVQDGIAK.[G]_	Q13410	79289	122716	47342	158589		70488	72880		81563	30571	80285	89146	96019	117419	39846	258047	270062	161318	142273	49928
[S].TDRSPYEKVSAGNGGSSL.[S]_	P15941	79057	78545	58286		17141	85177	131909	103919	42846	23200	70044	91595	98338	47081	18696	37618	82186	69560		15073
[L].SKEIPLSPMGEDSAPRDADTLH.[S]_	Q13410	78933	56998	51030	50654	13605	43329	69712	103055	47862	23575										
[S].KEIPLSPMGEDSAPRDADTLHSKLIPTQPSQGAP.[-]_1xOxidation [M	Q13410	78932	61027	82835		47610	98572	87601	197733	124377	63044	73481	56675	78938	52276	38412					
[P].QPKVLPIPQQVVPYPQRA.[V]_	P05814	78930	54552																		
[E].DKNSPFYYDWHS.[L]_	Q14802	78369	69061	75136		18148											11353	16250	12855		
[P].LSPMGEDSAPRDADTLH.[S]_	Q13410	78328	79986	56190	64578	14670	36662	49224	57929	23775	23052	113273	101370	126356	100470	56452					
[F].DVYTESWAQDPSQENKK.[K]_	P19835	78034	37831	65183	53218	65239	630855	690809	334670	442529	251460	73517	98176	72142	78727	27283					
[Q].NPSESSEPIPLESRE.[E]_	P47710	77884	55812	50097	68258	16028	147053	170928	94810	108580	48707	147914	143616	164429	141399	60909	38621	29203	26809		20735
[A].DGSRASVDSGSSEEQGGSS.[R]_	P01833	77718	32676	29527	290621	25443	87299	82199	139952	69499	15992	126283	124876	174029	117508	92030	26340	23950	21957	19718	13770
[V].SQDQEREK.[R]_	Q8NFU4	77428	57716	56827	233727	11655															
[K].APGRVLVHTSVSPSPDNL.[R]_	Q6WN34	77338	80089	78640	74899	87532	801973	802208	733408	652982	548945										
[I].PLSPMGEDSAPR.[D]_	Q13410	77010	77869	64159	88629		53261	72362		40133	21375	85412	95995	90486	76720	43834	59840	75749	59203		
[I].DELFQDRFFTREPQDTYH.[Y]_	P10909	76773	84279	73480		32111	79613	95236	66433	67750	44658	263361	255761	230858	181421	106614					
[K].KVEISIPASSLPRLTPWIVA.[V]_	Q13410	76612	103629	150136		23064						317130	311600	303454	198423	144525	707663	741326	528413	266153	278801
[E].SLSSSEESITEYKQK.[V]_	P05814	76589		56840	93570	73392	422614	375646	292280	419373	208084	373143	409469	378906	627091	444346	23062	25400	21535		25620
[D].GREQEAEQMPEYRGR.[A]_	Q13410	76485	85112	68047	94352		242620	263639	256941	53275		211743	213304	199835	171781	110519	199687	247666	243602	51573	204106
[T].IPFFDPQIPKLTDLENL.[H]_	P05814	76274	102029	93853		41501	247118	251413	225058	144634	44408	163238	123489	182356	145678	109445					
[E].DEHQDKIYPSFQPQPLIY.[P]_	P05814	76015	70550	73434								51013	31569	36311	34131						
[L].NQELLLNPTHQIYPVTQPLA.[P]_	P05814	75927	53010	70205		106504	69685	71392		95695	168739	20306			120522	559937					138480
[E].DVSNKVSMSSTVQG.[S]_	P31431	75760					101890	105520		55551		112749	97104	106197	113169	50111					
[A].QPILSKLEPQIASASE.[Y]_	O60664	75631	82646	68382								65834	67332	82681	53050	28993					
[S].QGNTNGFPATASNDLK.[A]_	Q9UNQ0	75507	61106	39994	78887	16910	37712	36321		23918	16584	53056	70070	49745	44461	25836					
[S].SGVENALTKSELLVE.[Q]_	Q99541	75453	101830	78843		24014						69352	89442	91528	69442	37932					
[G].EPLDDYVNTQGASLFSVTKK.[Q]_	P00747	75049	93971	47294			80202	88923	89880	52579		113190	130767	111078	81938	36545	122756	130954	113128	60768	54594
[H].AQIPQRQYLPNSHPPTVV.[R]_	P07498	74886	77887	106867	49465		96140	99407	122722	60726	37333										
[K].GRVMPVLKSPTIPFFDPQIP.[K]_1xOxidation [M4]	P05814	74457	97932	186444		2453539	574568	791022	825319	1169189	3307194	102945	129531	105279	434383	1614796					95956
[Y].PVTQPLAPVH.[N]_	P05814	74222	52246	52690													58211	66511	55220		

Quantified modified peptide sequence	Protein ID	A_-80oC	A_-20oC_120h	A_4oC_6h	A_RT_6h	A_RT_24h	B_-80oC	B_-20oC_120h	B_4oC_6h	B_RT_6h	B_RT_24h	C_-80oC	C_-20oC_120h	C_4oC_6h	C_RT_6h	C_RT_24h	D_-80oC	D_-20oC_120h	D_4oC_6h	D_RT_6h	D_RT_24h
[I].ESLSSSEESITEYKQKVE.[K]_	P05814	74205	54207	47548		22923	40745	47370				101566	55339	69079	56406	28177					
[A].DTRDQADGSR.[A]_	P01833	74176	56229	41082	100188	22726	109337	123751	208363	80340	57623	27512	26871	23605	22551		70111	90949	71612	32367	23466
[E].PIPLESREEYMNGMNR.[Q]_	P47710	74072	67647	57460		24678	3448937	3390681	3207827	4360270	3074840	2221359	2446132	2054313	2214095	1959844	104932	192752	157818	51185	136065
[R].DALSQLMNGPIRK.[K]_	Q9HB40	73786	71367	90253	51096	57607	79252	77622	51188		81932	63292			62603	52162					
[V].MPVLKSPTIPFFDPQIPKLTD.[L]_	P05814	73394		34730	50509	37680	1018509	948769	763889	636086	383145	737860	791811	675857	611968	530781					
[Q].NPSESSEPIPLESREEYMN.[G]_	P47710	73320	146076	98266		43169	90506	120404	365558	108861	125799	74279		333758		26315	30483	25392	29351		
[A].DIAKEDQIKKL.[H]_	Q14839	73282	50797	50155														24235	23958		
[D].APPPPAAPLPRWSGPIGV.[S]_	Q99523	73015	77047	54719		15135															
[R].VMPVLKSPTIPFFDPQIPKLTDLENLHLP.[L]_	P05814	72936	167314	97197		136649															
[V].APEEHPVLLTEAPLNPK.[A]_	P60709	72582	76150	70321	68973	40857	338204	288974	191221	257196	402713	103230	75650	105374	89765	180572					
[A].DTKELVSSKVSGAQ.[E]_	O60664	71913	70665	61389	97907	11411						88584	111677	145667	98495						
[M].DPAEEDTNVYTEK.[H]_	P34741	71367	54434	38161	160116	15400	92270	127565	111098	75920	37328										
[G].DSAVPTHWEPYTTENSGYLEIT.[K]_	P19835	71293	52740	35358		66793	584081	607094	404052	568549	610358										
[F].MEALQAGADISMIGQFG.[V]_2xOxidation [M1; M12]	P07900	70920	81275	101489	122953	46961															
[L].APVTTFGKTNGYIA.[-]_	Q12913	70860	91791	51167		17739						107801	143699	114876	110916	64216	38790	33630	32742	21373	
[S].APRDADTLHSKLIPT.[Q]_	Q13410	70748	80191	39621								53950	58435	69744		28524		16063	16463		
[L].GPNTGLSGGMPGALPSLPGKI.[-]_1xOxidation [M10]	Q9NS69	70740	58132	50576		14804															
[A].PAPPPPPPPP.[T]_	Q12830	70726	58104		175388	14797						32124	34711	73490	32543	10740	23390	24229	16726	22973	
[D].DPDAPLQPVTPLQLFEGR.[R]_	P0C0L4	69959			72414								109444		161000	102777					
[K].AKDTVYTKGRVMP.[V]_	P05814	69931	124415	71400	135957	153890	219933	239874	272432	302057	135977	132238	169260	109595	159464	181143		16690	14211		46193
[G].AVYTEGGFVEGVNKK.[L]_	P19835	69843	75036	83786	201378	89686	656877	582074	749639	1428469	681670		22369		124287	48736					
[Q].TLELKVTASPDKVTKT.[-]_	Q6WN34	69813	52528	60527		41865	58902	63448	79404	51765	51141										
[A].DTLHSKLIPTQPSQ.[G]_	Q13410	69710	57207	53321		19830	41521	39926		24799		38725	47325	61113	37397	24698					
[R].DVAVVAGGLGRQLL.[Q]_	Q15904	69570	45215	56542			65708	71690		71948	114613	71279	76855	134975	73891		44046	55213	65853	60230	56257
[I].YPVTQPLAPVHNPI.[S]_	P05814	69423	55361	54498		14259						32910	30334	33595	24236	13391					
[A].APGQEPPEHMAELQRNEQEPLGQWHLS.[K]_	O00391	69151	65946	102831		24581	174786	197540	226411	87879		65504	66087	73483	41954	45415					
[Q].PEIMEVPKAKDT.[V]_	P05814	68901	46147	36753	53871	17105															
[S].SYSKQFTSSTSYNRGDSTFESKSY.[K]_	P02671	68731		156961		50314					54046										
[G].RVMPVLKSPTIPFFDPQIP.[K]_	P05814	68608	134630	43493	118648	796817	222632	206487	95022	307017	1203515	447479	423408	376847	1070438	1998692					
[A].YLSTKDQYPYLK.[S]_	Q99541	68595	58996			22912						2773211	3469319	2171651	2114675	1203312	189927	264015	353262	52721	41621
[R].TYYANPAVVRPHAQIP.[Q]_	P07498	68548	88976	92185	50816	99263						114412	146552	151618	140053	172580					
[P].RDADTLHSKLIPTQPSQGAP.[-]_	Q13410	68193	88079	68291		25435	59590	48930	87415	38220	22083										
[M].PVLKSPTIPFFDPQIPKLTDLENLHLPLP.[L]_	P05814	68184	226997	329427		451352	888205	965580	982306	1453181	1607700	161514	164316	178867	191119	307356					
[L].ELKVTASPDKVTKT.[-]_	Q6WN34	68176	53091	47904	139648	229735	64989	67423	112851	48735	46195										
[I].DELFQDRF.[F]_	P10909	67970	44516	60384	136149	23192	116719	121504	57747	102922	85830	108818	105694	132910	153559	65924					
[E].EELEKEAKKVEGFDLVQ.[K]_	Q99541	67670		79897								648261	553927	554923	377769	247444	30865	44117	33063		12589
[F].AAPTKALENPQPHPGWQGTLK.[A]_	P19835	67664	83269	97712		134614	775925	663724	939308	948724	716259	190385	188551	165958	215200	174792					
[K].CAEQFCRLNEYNQLQLQAAHAQEQIRR.[M]_	P47710	67555	19302	32526		24285	219830	280818	87703	414364	220659	72097		103943	54016	85885					
[E].DKTERELLESYIDGR.[I]_	P00734	67292		49070		207466	302974	302686	255313	888820	1268503	104658	96488	82400	75134	106279	31032	51505	28722		34502
[-].MDNLSSEEIQQRAHQITDESLE.[S]_1xAcetyl [N-Term]	O00161	67129	46421									57780	48833	62764	40984						
[M].PVLKSPTIPFFDPQIPKLTDLENLHLP.[L]_	P05814	66974	294484	293850		220636	140257	110497	270137	128173	108761										
[A].DGSRASVDSGSSEEQGGSSRALVSTL.[V]_	P01833	66120	58211	65982	56713	21607															
[V].DLSGHNLANPHPFAVVPSTA.[T]_	O94985	66039	99267	87254	63671	30658															
[E].APVTKIELLPSY.[S]_	O95436	65984	54322																		
[G].QPLQERAQAWGERL.[R]_	P02649	65731	51317	39335		15549	68383	72411		36938	21288										
[A].DGVLVGTDGRYS.[S]_	Q13113	65477	52423	45090	117370			31119													
[L].PIPQQVVPYPQRAVPVQ.[A]_	P05814	65249	64777	71841	103826	145234	316662	228839	578317	212430	271548	278880	328760	372647	707382	2152881	79917	93352	103122	177029	441675
[L].SSSEESITEYKQKVE.[K]_	P05814	65200	50438	54138		19263			44710		23731	174574	161838	172269	174802	75886					
[K].YPDAVATWLNPDPSQKQNL.[L]_	P10451	64932	79479	58242	62035	30754	107616	103285	83378	85160		543665	514269	561980	485998	288623					
[G].DDGNLIIK.[K]_	Q9GZU8	64749	54793	43281	141423	15942						33894	31847	36762	25060	16121	23728	30694	26122	33083	18276
[K].LPSDGKEVPFVVPK.[F]_	Q8N9U0	64712	44293	52923	41941																
[N].ILREKQTDEIKDTR.[N]_	P47710	64583	115749	188951		134290				66949	54972	72599	74451	71342	169179	184234					
[S].IPASSLPRL.[T]_	Q13410	64557	69716	43541	41040		146865	137267	342967	49206	21895	189146	270915	130533	72856		247605	498171	142211	43924	14092
[K].LPSDGKEVPFVVPKF.[K]_	Q8N9U0	64501	86201	41183		19797								38290							
[F].DPQIPKLTDLENLHLPLPL.[L]_	P05814	64496	54288	38616		152833	624443	560026	226220	470475	437370	827306	598589	732182	802751	993728					147461
[A].DTRDQADGSRASVDSGSSEEQGGSSRALVST.[L]_	P01833	64451		55006		22683						78733	79271	97440	78350						
[A].VPFKILYNGQSVEVDGHSM.[R]_	P10586	64427	94110			56329	111346	97655	123141	119403	126563										
[R].RPDIQYPDATDEDITSHMESEELNGAY.[K]_1xOxidation [M18]	P10451	64247	47358	86033	63563	69511	250086	245668	434210	219206	144845										
[K].AIQDPRLFAEEKAVADT.[R]_	P01833	64068	133845	137724	150507	344540	1055323	1142896	2948359	2989580	3110883	119702	155818	96841	112937	51358	80538	158244	83376	55466	33249
[I].SPVEESEDVSNK.[V]_	P31431	64023	50371	40823	155865	10547	137066	165394	161413	93631	23193						22966	23351	20521		
[S].VPQPKVLPIPQQVVPYPQRAVPVQA.[L]_	P05814	63709	39209	62279		52084	125554	130616	861773	70028	44773	168555	217979	197367	233903	536046	28302	30351	38413		97491
[P].PQSPWDRVKD.[L]_	P02647	63496	63460	61519	95150	20827	50030	52322		33775	19377	168589	173574	157351	135889	87170	43521	47861	39931	30247	27008
[D].QADGSRASVDSGSSEEQGGSSRALVSTL.[V]_	P01833	63365	57257	57664	93651	28541															
[A].LPPQPLWSVPQP.[K]_	P05814	63235	90701	98374	424062	569811	277469	262880	155772	506814	849161	227215	243969	166366	389438	372513					
[T].TFDVYTESWAQDPSQENK.[K]_	P19835	62958	62914	97507	395648	325104	275051	325552	190850	1309682	2082005	30916		24338	114074	104359					
[Y].VPPSSTDRSPYE.[K]_	P15941	62764	48955	51589	113254	13601	41008	54636		28776	12142										
[S].EDVSNKVSMSSTVQGSN.[I]_	P31431	62753	69013	44773		17634	75192	49290		24191		31049	42025	45402	31337						
[G].RVMPVLKSPTIPFFDPQIPK.[L]_	P05814	62633	107085	102273		913698	1065359	1131793	1178996	2005281	3190444	59845	76666	63322	161577	198721					
[T].DEDITSHMESEELNGAYK.[A]_	P10451	62538	57508	59620		141091	1111224	1032060	1240818	1013074	640069	113828	97195	77752	57891	25771					
[I].PKLTDLENLH.[L]_	P05814	62456		32313		18343	39966	42795		36142	43875	258791	255295	284458	254199	203081					
[L].PLRYPERLQNP.[S]_	P47710	62406	98682	61322		134692	471961	395074	390724	402427	267424	191384	233047	125325	211868	251982					
[R].SNTLSSPNSKNEKALGR.[K]_	P50591	62382	92524	74618		24238						29331	55083	42135	29954	25258					
[L].DINTNTYTSQDLKSALAKF.[K]_	Q9H173	62266	75793			52396	78450	72042		61903	43464										
[V].ADTRDQADGSRASVDSGSSEEQGGSS.[R]_	P01833	61532	36583	58710	195730	17523	43295	72543	172869	46898	10293	41029	49453	58790	64028	24302					

Quantified modified peptide sequence	Protein ID	A_-80oC	A_-20oC_120h	A_4oC_6h	A_RT_6h	A_RT_24h	B_-80oC	B_-20oC_120h	B_4oC_6h	B_RT_6h	B_RT_24h	C_-80oC	C_-20oC_120h	C_4oC_6h	C_RT_6h	C_RT_24h	D_-80oC	D_-20oC_120h	D_4oC_6h	D_RT_6h	D_RT_24h
[A].NPAVVRPHAQIP.[Q]_	P07498	61490	68374	75808	96366	125505	18896	35831		24427	124634										
[V].DLSGHNLANPHPFAVVPS.[T]_	O94985	61312	74818	95283			98676	82463	62175	71918	41692										
[E].YNQLQLQAAHAQEQIRR.[M]_	P47710	61090	143224	137266		861303	509185	440242	403086	1419340	1955499	172279	77150	84382	275571	454701					
[P].LWSVPQPK.[V]_	P05814	61072	65143	93673	386189	303897	354543	420613	363224	823630	1239052										
[S].DMREANYIGSDKYFH.[A]_	P0DJI9; P0DJI8	60990	48452	52088		41444															
[R].TYYANPAVVRPHAQIPQRQ.[Y]_	P07498	60903	107950	96642		46745	61711	66925		48022	37709	157220	154568	115659	154608	266535		20485			18525
[T].LALPPQPLWSVPQPKVLPIPQQVVPYPQR.[A]_	P05814	60731	68558	205143	221328	3630363	478067	479099	546047	1201389	2708834	61112	124845	138294	270034	662282					66438
[R].TPKIQVYSRHPAEN.[G]_	P61769	60528	57734	54021		22137															
[M].SSAYLSTKDQYPYLK.[S]_	Q99541	60477	84039			37783	77542	75969	105097	55209	104169	4630167	5081593	1350153	3223629	2408445	802874	857582	585764	537591	207920
[K].VKHEDQQQGEDEHQDKIYPSFQPQPLIY.[P]_	P05814	60327	114470	112952		38185						41315	23255		36609						
[Y].EKVSAGNGGSSLS.[Y]_	P15941	60260	41002	29398	220699	14809	31966	32067	130435	34999		14444	15235	31487	17689	13526					
[P].DESSKPNMIDAATL.[K]_	P02679	60165	52891	40090	85557	31499															
[P].LAQPAVVLPVPQPEIMEVPKAKDTVYT.[K]_1xOxidation [M16]	P05814	60164	70185	102213		155919	741954	883882	901626	324976	369766										
[V].LPVPQPEIMEVPKAKDTVY.[T]_1xOxidation [M9]	P05814	60126	32662	97631																	
[I].ESLSSSEESITEYKQKVEK.[V]_	P05814	59967	33210	84369		126404	1442904	1507321	1487098	1214677	564364	2134657	1963408	1914514	2043284	1420272					
[Q].GPPEEAEDRFS.[M]_	O00592	59790	46423	43279	131239	17729	44647	51990	100613	36611	26909	19963	21670	27048	28960	9860					
[R].TNKLPGSPGLS.[K]_	Q8N9U0	59644	47491		86252	11034															
[G].SSEEQGGSSRALVSTL.[V]_	P01833	59642	35529	35193																	
[E].DQQQGEDEHQDKIYPS.[F]_	P05814	59627	61835	53065	176322	23403	414751	621500	499523	344251	141958	1222480	1285990	1519531	1280701	386019	163159	205890	243297	109344	109290
[L].PIIQKLEPQIA.[V]_	Q99541	59056	73101	37352		30159						277541	299514	225265	215813	135488	58074	48480	69287	49461	20155
[S].KEIPLSPMGEDSAPRDADTLH.[S]_1xOxidation [M8]	Q13410	58884	45883	69393		20376	67058	91544	329479	58116		214329	246941	192794	174478	135111	15838	30707	19855	89576	24682
[A].GGAGGGAGRKEDWNEIDPIK.[K]_	Q10471	58740	45320	52479		20384												16599	15985		
[R].RPAIAINNPYVPRTY.[Y]_	P07498	58336	56428	64240			216123	175246	145801	62367	92378	26643	27012	40974	47148	51768					
[D].IQYPDATDEDITSHMESEELNGAY.[K]_	P10451	57929	46431	50422		39733	239604	265534	211968	225880	214937										
[K].VLPIPQQVVPYPQRAVPVQALLLN.[Q]_	P05814	57919	100480	98756		210085	783948	693158	539788	660938	1545987	1761145	1867895	1825926	2070627	5206465					
[F].FDPQIPK.[L]_	P05814	57785	83987	29973	379229	326653	1128699	1051962	1038578	1973865	2465751	115894	128045	101381	216659	224661					
[E].ESITEYKQKV.[E]_	P05814	57271	52591	46289	388503	141816	276334	365511	252994	396237	191773	662402	648717	737720	1151263	1063676	37923	54862	45438	45144	53870
[R].GAEDSLADQAANKWGR.[S]_	P0DJI9	56874		60966																	
[Q].ALLLNQELLLNPTHQIYPVTQPLAPVHNPIS.[V]_	P05814	56821		75328	425856						301113										
[V].LPIPQQVVPYPQ.[R]_	P05814	56640	71690	42553	156902	206961	1460393	1573632	840604	1288788	2460731										
[L].LLNPTHQIYPVTQ.[P]_	P05814	56580	54507	30760	51778	25198	657543	444095	485925	492059	568329	230909	268861	215060	296160	315454	28426	32236	34496	41833	85118
[I].YPSFQPQPLIYPFVEPIPYGFLPQNILPLAQP.[A]_	P05814	56462	205005	72559		9163						207201	88920	130418	160307	79314					
[P].TIPFFDPQIPKLTDLENLHLPLPLLQPLM.[Q]_	P05814	56127	214350	62875		130701	324304	269024	361421	222882	186272	1306808	944273	1126529	983722	648684					
[K].DSHSLTTNIMEILRG.[D]_	P02671	55929				131437	80441	69062		147990	232965										
[I].AINNPYVPRTYYANPAVVRP.[H]_	P07498	55795	76325			62454						39441	45813	46396	35957	74306					
[C].DAAEKGVRTLT.[A]_	O60664	55641	58230	37199	98441							72175	78249	66065	60407	38392					
[L].RPAVSSSDQQSLIRKLQ.[K]_	Q8IZA0	55447	64400	70077	102614	74811	90000	85672	118696	94140	61609										
[E].SLSSSEESITEYKQKVE.[K]_	P05814	55063	50168	46994		22373	63447	82377	67232	46671	66914	95080	105754	33375	96001	129117					50263
[E].IPLSPMGEDSAPRDADTLH.[S]_1xOxidation [M6]	Q13410	54967	60480	65252	47324	29195						77091	99500	75228	63006	58964					
[S].SIQGLPTNVKDQVQQ.[A]_	O60664	54686	61914	44126								18520		54019	18508	8773					
[S].GEGDFLAEGGGVR.[G]_	P02671	54345	41831	36996	50686	10947											39289	38052	40007	29300	16734
[H].ENYEKNNVMLQW.[-]_	P47710	54255	60984	104800	177298	21315	42549	41696		22676	36334										
[Q].DLNAPSDWDSRGK.[D]_	P10451	54135	46755	50019	127316	56882	834116	887615	613221	1048362	1243037	2933015	3064481	2878527	3776651	2021053					
[K].DLYANTVLSGGTTMYPGIADR.[M]_	P60709	54059	57611	60413		30623	87533	83367	79471	120929	80649	95266	104831	114840	121956	89956					
[R].GLEEELQFSLGSKINVKVGGN.[S]_	P0C0L4	53997	38503																		
[S].DISNPTAHENYEKNNV.[M]_	P47710	53950		44815		31943	91666	124329	84558	69280	22536										
[P].IPQQVVPYPQRAVPV.[Q]_	P05814	53935	60973	46400			295000	296122	176974	183046											
[Y].VPFPPFSDISNPTAH.[E]_	P47710	53846	40486	40744		11200	65150	55222	37730	24781											
[P].VPQPEIMEVPKAKDTVYT.[K]_1xOxidation [M7]	P05814	53528		112027	55904	28103	111433	144285	258481	67568	32828										
[T].VYTKGRVMPVLK.[S]_1xOxidation [M8]	P05814	53128	61501	104554	183912	148493	244850	240763	450003	320476	83897										
[I].PFAAPTKALENPQPHPGWQGTLKA.[K]_	P19835	53115	44741	81175		17671	181887	146761	221804	98801	49203		36871		24845	26558					
[R].RPDIQYPDATDEDITS.[H]_	P10451	53010	33589	45429		18833	344394	304587	142928	116380	57196	216456	191897	191601	161041	109785					
[I].PFFDPQIPKLTDLENLHLPLPLLQ.[P]_	P05814	52965	61328	44989		76830						119259	95334	138131	92375	95941					
[P].SESSEPIPLESREEYMNGMN.[R]_	P47710	52915	61069	64694	78968	69415	438701	464444	413258	588816	689006	354793	331865	412189	486385	407811					
[W].LPSASKEPDNHVYT.[R]_	P19440	52883					127514	136273	64428	69603	35101										
[T].EGGFVEGVNKKLG.[L]_	P19835	52484	54118	63590	102928	163761	728513	581834	557847	832748	715255	106351	122780	110365	81332	86108					
[L].ENPQPHPGWQGTLK.[A]_	P19835	52450	39969	70590	202760	101834	720346	859721	418690	1635063	1306364	183425	134605	199024	446703	209878					
[P].EIMEVPKAKDTVYTK.[G]_	P05814	52396			95381	219346	315388	303844	368989	1153337	1000801	37888	35645		53812	79981					
[N].EITRGGSTSYGTGSETESPRNPSSAGSWN.[S]_	P02671	52367	67488	42865																	
[A].DTKELVSSKVSGAQE.[M]_	O60664	52267		61208				46452		16741				70340	40266	27709					
[G].QLQQPTTGAASGGYIK.[R]_	O00161	52129	42105	30465																	
[A].QPAVVLPVPQPEIME.[V]_	P05814	51929	59779				75587	82584		47968	37635	49146	44438	52558	49140	23697					
[S].DMREANYIGSDKYFHARGNYDAA.[K]_	P0DJI9; P0DJI8	51375	132148	92434		53856															
[K].TTFDVYTESWAQDPSQENKK.[K]_	P19835	51246	42054	73661	120736	97679						46951	37452	77609	105462	80108					
[R].YPERLQNPSESSEPIPLESREEYMN.[G]_	P47710	51116	47931	36596										30450	20856	96398					
[L].PAEPAAARGGGGGGGGGGGGRGP.[Q]_	Q86Y38	50846	38737	39125	71648	20231															
[R].VMPVLKSPTIPFFDPQIPKLTDLENLHLPLP.[L]_	P05814	50406	304615	386137		497311	1020608	1234966	1456432	1853826	2089952	156937	178721	65465	227926	351889					
[H].QIYPVTQPLAPVHNPIS.[V]_	P05814	50376	43162	73671								315880	437257	304598	515338	362875					
[T].VYTKGRVMPVL.[K]_	P05814	50060	58792	60297	418208	340972	78510	73051	116930	252710	268521	28786	33900	28498	133277	80504					
[R].DTGAALLAESRAEK.[N]_	O00391	49816	37140		152528	24833	40001	44649		67261	59451	34069	33587	39341	28255						
[S].VPGSQTVVVKEIT.[I]_	O00592	49770	50459	46411	69062							36954	34906	27368	31076						
[T].DRSPYEKVSAGNGGSS.[L]_	P15941	49675	35663	35453	170465	17683															
[R].DGREQEAEQMPEYR.[G]_1xOxidation [M10]	Q13410	49333	33488	74374	130283	16743	209093	296073	444283	143284	45729	50482	111799	84541	76558	41996	332180	384987	303574	1139558	338393
[T].IESLSSSEESITEYKQKVEK.[V]_	P05814	49315	65704			66183	688971	546545	581422	353077	192665	756057	751665	665501	991637	1307571					

Quantified modified peptide sequence	Protein ID	A_-80oC	A_-20oC_120h	A_4oC_6h	A_RT_6h	A_RT_24h	B_-80oC	B_-20oC_120h	B_4oC_6h	B_RT_6h	B_RT_24h	C_-80oC	C_-20oC_120h	C_4oC_6h	C_RT_6h	C_RT_24h	D_-80oC	D_-20oC_120h	D_4oC_6h	D_RT_6h	D_RT_24h
[E].NPQPHPGWQGTLKAKNF.[K]_	P19835	49295	73451	79501		44321	452122	449841	402561	311107	118143	77237	80271	65716	60735	60037					
[I].NTVLGSRMM.[Q]_	Q99541	49268	49744	45832	83637							129817	196621	118317	100311	37497		47665	40302		
[K].LTDLENLHLPLPLLQP.[L]_	P05814	49232	77974	89410		116154	332647	321500	215036	372509	482408	91564	95351	122070	108982	163539					
[L].FSGPRQAFEFPQINYQKYCGKPYTYA.[Y]_	Q16518	49220	71588			96816	1448901	1311575	816218	1298139	1667275	341117	372372	363174	282439	308243	44031	76231	40645	49258	18311
[R].NGFKSHALQLNN.[R]_	P0C0L4	49038	51833	49663	94999	102682						146194	157322	213515	204060	160057					
[L].ADQAANKWGR.[S]_	P0DJI9	48987	73390	51768																	
[Y].DAAQRGPGGVWAAKLISRS.[R]_	P35542	48373	62020	56308	42165	18894	65246			61316	30548										
[A].SVDSGSSEEQGGSSRA.[L]_	P01833	48359	32972	13994	107890							40360	43334	50798	27003	18178	104419	85037	101810	51759	40444
[C].HENDERPFYQK.[T]_	P07498	48239	47460	67934		33517	148822	151891	240236	220235	159866	122941	132051	114226	111925	89017					
[R].SPYEKVSAGNGGSSLSYTNPAVA.[A]_	P15941	48223	42866	40775			45820	56116	186502	27738	15036										
[G].STTVKIVLK.[E]_	Q6WN34	48054	34017	27217	144287	47145	148402	132069		159971	140358										
[A].TLVQDGIAKGRVAL.[R]_	Q13410	47882		45028													19780	37714	22384		
[V].LKSPTIPFFDPQIPK.[L]_	P05814	47873	36663	79498	43490	400684	766169	925554	1290797	1043282	955023					26797					
[I].QDPRLFAEEKAVADTRDQA.[D]_	P01833	47762	80647	40206		104089		57681		60701	191655										
[P].EIMEVPKAKDT.[V]_	P05814	47750	34471		136188	71906	48569	42427		62720	70532										
[V].DDTQFVRFDSDAASPR.[G]_	Q07000; P30510	47695	53462	45506	65132	20475															
[D].QADGSRASVDSGSSEEQGGSSRA.[L]_	P01833	47204	40597	43923	175302	11600	119638	170702	155125	59321	15758	94914	97794	152409	125185	79775	43463	29299	43238	28596	28392
[Q].DLNAPSDWDSRGKDS.[Y]_	P10451	46958	50258	37516	69697		714788	808909	618170	517625	379411	2867499	2997137	3327514	3008811	1900313					
[A].TDEDITSHMESEELNGAYK.[A]_	P10451	46705	64432	54698		91282		74534	424173												
[G].DIKVTESEIKR.[R]_	Q7Z7D3	46677	55953		50817		196749	234044	205383	135863	19170	74120	77943	64251	63403	31368	39094	63448	59212		25237
[I].AINNPYVPR.[T]_	P07498	46511	75357	35684		34007															
[F].VEPIPYGFLPQNILPLAQPAVVLPVPQPEIMEVPKAKDTVY.[T]_	P05814	46486	283870			18016															
[K].VKHEDQQQGEDEHQDKIYPSFQP.[Q]_	P05814	46090	71900	59699		41155															
[K].LTDLENLH.[L]_	P05814	46031	56600		101405	47722	273783	249271	121846	245605	148728	37152	51226	46868	70850	166972					
[A].DTKELVSSKVSGAQEMVS.[S]_	O60664	45964	53099	35997								50019	59121	78338	51251						
[P].LAQPAVVLPVPQPEIMEVPKAKDTVYTK.[G]_	P05814	45930	51247	97428		537917	354384	311489	715066	544253	489796	36744			52581	103209					
[V].PPSSTDRSPYEKV.[S]_	P15941	45734	33527				32315	42432													
[Y].NPGNPHNVYMPTS.[Q]_	Q969T9	45538			83937							107983	103211		66380	44290					
[Q].PAVVLPVPQPEIMEVPKAKDTVYTK.[G]_	P05814	45182	49310	35919		542272	403070	377160	3668074	834029	351295	234187	207371	188510	235089	160033					
[V].DATRGAVQSGVDKTKSV.[V]_	O60664	45163	41859																		
[R].GAKTTFDVYTESWAQDPSQENKK.[K]_	P19835	44835	65321	75689		95327	258937	263099	438091	587706	473654	36117	38952	74175	82027	60944					
[R].LFAEEKAVADTRDQADGS.[R]_	P01833	44694		31294														15314			11889
[K].AVADTRDQADGSRASVD.[S]_	P01833	44591	31415	28119		10882	429882	366401	228353	278082	63816	91604	70755	61600	99504	48174	39594	33288	52309	25248	
[R].LFAEEKAVAD.[T]_	P01833	44402	53494				34403	33395					26113	38029	29597		39738	49043	33986	30245	
[R].NGFKSHALQLN.[N]_	P0C0L4	44125	52175			77506						149719	165946	174917	110660	80555					
[S].EYDYVSFQSDIGPYQSGR.[F]_	Q8N474	44108	51535	89784		64285	64384	56375	93994	45507	75194			93501							
[M].EVPKAKDTVY.[T]_	P05814	43858	45569	27693	175255	91388	143296	146799		189396	79071	76613	72475		92023	177825					
[N].EYNQLQLQAAHAQEQIRR.[M]_	P47710	43755	41134			162907	156126	151446	135472	445513	557477	130123	110731	112618	152106	256633					
[A].GRDLSRLPQLVGVSTPLQGGSN.[S]_	P15291	43689	48566	35353		17376															
[R].DALSQLMNGPIR.[K]_	Q9HB40	43498	37737	35670	77802	21951	21936	17783	68011		23133										
[L].FAEEKAVADTR.[D]_	P01833	43337	47488	40504		37008	51167			50177	61855	26199					60831	118070	73939		
[R].NGFKSHALQLNNRQ.[I]_	P0C0L4	43088	81594	62901		45728						700402	896152	850341	719883	462574					
[R].DPEAASPGAPSRDVLL.[V]_	O43581	42898		44001	65180																
[Q].ELLLNPTHQ.[I]_	P05814	42852	39368	28391	84268	38025	145216	138033	65940	74215	85261	81842	89687	116031	129338	131708					
[T].SYNRGDSTFESKSY.[K]_	P02671	42532		55014		27554				20922	22388										
[P].LAQPAVVLPVPQPEIMEVPKA.[K]_	P05814	42400		49389	104028	687964	299655	349718	88285	612977	1570001					134655					
[Y].DESGPSIVH.[R]_	P60709	42277		34696	112529																
[S].IPASSLPRLTP.[W]_	Q13410	42156	59700	33937		10040						83310	82194	60833	32199		47772	183494	31614	28482	7171
[V].LPVPQPEIMEVPKAKDTVYTKG.[R]_	P05814	41818				17229															
[P].LESREEYMNGMN.[R]_	P47710	41406	30888	35695	124224	117140	452733	504849	166811	423308	517316	259397	243433	181107	196891	133170		30478	20602	23985	
[A].DGSRASVDSGSSEEQGGSSRALVSTLV.[P]_	P01833	41193	48233	44943		20689															
[P].LMQQVPQPIPQTLALPPQPLWSVPQPK.[V]_	P05814	41154	57311	44340	103375	1163872	847552	867219	935692	1511634	4259123										
[I].LPLAQPAVVLPVPQPEIMEVPK.[A]_	P05814	41014	78895			83908	321041	267255	1030314	209816	357245	381565	391342	410562	398701	252167					
[I].MEVPKAKDTVYT.[K]_	P05814	40981	48799	35299	180810	369381	1120298	951068	4786252	879761	1611177	304172	258822	279568	677189	751834					158366
[K].LTDLENLHLPLPLLQPLM.[Q]_	P05814	40900	70328	105069		82822	545793	443354	256755	328034	641711	578972	541453	467153	365810	338927		40338		18517	19394
[R].LTGRGAEDSLADQAANKWGR.[S]_	P0DJI9	40771																			
[E].DEHQDKIYPSFQ.[P]_	P05814	40735		36383		11099															
[T].IESLSSSEESITEYKQK.[V]_	P05814	40688				39569	132056	111349	112093	155966	100742	70287	125742	124493	166346	141681					
[C].DAAEKGVRTL.[T]_	O60664	40606	38886	31117		23855						27887	32059	44553	24481						
[M].EVPKAKDTVYTK.[G]_	P05814	40494	43325	29766	347951	356340	278878	240606	443870	625764	281115	107094	113591	68305	201596	362943					74869
[S].VDSGSSEEQGGSSRALVSTLV.[P]_	P01833	40419	42856	60954	81761	51224															
[D].TRDQADGSRASVDSGSSEEQGGSSRA.[L]_	P01833	40084	31707	29288	159963	13552	20839	32182	75925	15986	5760	153339	157719	137406	131314	106927	29506	28878	24086		21368
[G].GAAAPYPASQPPYNPAYMDAPKAAL.[-]_	Q8N114	40065	52895	44756																	
[P].TIPFFDPQIPKLTDLENLHLPLPLL.[Q]_	P05814	40056	108056									108876	79077	21284	91855						
[S].GGGGGGGLGSGGSIRSSY.[S]_	P35527	39883																			
[P].EIMEVPKAKDTVYTKG.[R]_	P05814	39446	43825	36426		114802	44435	58627	77197	66298	66601										
[P].VPQPEIMEVPK.[A]_1xOxidation [M7]	P05814	39368	35442	44829	49462	18764	175375	204687	178243	104353	55159										
[L].PQNILPLAQPAVVLPVPQPEIMEVPKAK.[D]_	P05814	39202	52917	65862		48644	45904	25692	839867	291378	43984										
[I].TSHMESEELNGAYK.[A]_	P10451	39195	32780	29088		39977	305739	289780	369577	397746	143313	26573	31394	20985	46914	33798					
[P].DAVATWLNPDPSQKQN.[L]_	P10451	39193	49353	62081	55804		78324	35999	43905	47691	37635	74211	77146	107064	66280	39876					
[P].LAQPAVVLPVPQPEIMEVPKAK.[D]_	P05814	38764		41378	73480	185052	43165	169143	325031	563501	107416	31362	35226	35788	70734	148151					
[N].QLQLQAAHAQEQI.[R]_	P47710	38716	50007	34543		19280	33623	17628			74875										
[E].PIPLESREEYMNGMN.[R]_	P47710	38431	55658			49173	236526	273108	299881	283974	308654	68821	71554	68529	64070	55584					
[T].TFDVYTESWAQDPSQENKK.[K]_	P19835	38333		29922	100415	150830	77253	97807	71774	125676	262022		32763	17950	43913	51915					

Quantified modified peptide sequence	Protein ID	A_-80oC	A_-20oC_120h	A_4oC_6h	A_RT_6h	A_RT_24h	B_-80oC	B_-20oC_120h	B_4oC_6h	B_RT_6h	B_RT_24h	C_-80oC	C_-20oC_120h	C_4oC_6h	C_RT_6h	C_RT_24h	D_-80oC	D_-20oC_120h	D_4oC_6h	D_RT_6h	D_RT_24h
[V].DALRTHLAPYSDELRQ.[R]_	P02647	38294		32173		341895	50291	43508		49621	123718	46948	30890	39764	33541	36276					
[T].YDLMSSAYLSTKDQYPYLK.[S]_	Q99541	38290	487315			12294						116812	119928	125459	107136	54991					
[I].MEVPKAKDTVYTK.[G]_	P05814	38246			269969	525769	640090	467837	2250366	1223435	717677	130771	136051	142212	510456	530478		10482		31106	149344
[P].AVVRPHAQIPQR.[Q]_	P07498	38211	131587	181650		172811	69283	82680	143763	144111	243304	54253	68593	113524	55912	76243					
[M].PVDLELVAKQNPNVK.[M]_	P15291	38022	37627			92477	36299	28175		48445	49260				23248	24596					
[I].QDPRLFAEEKAVADT.[R]_	P01833	38004		33621	47960	212166	687676	694151	738114	2254699	2342609										
[E].NPQPHPGWQGTLK.[A]_	P19835	37521			71289	170629	700081	542207	290314	1029177	1104390	34562	47400	43177	102328	59184					
[-].MDNLSSEEIQQR.[A]_1xAcetyl [N-Term]	O00161	37419	32482	35159		15296															
[E].RLQNPSESSEPIPLESREEYMNGMN.[R]_2xOxidation [M21; M24]	P47710	37108		65646	43231	16188	1148664	1288502	2512733	629696	456973	310108	396839	349743	333657	512219	52815	49740	54707	500004	121260
[S].DISNPTAHENYEKNN.[V]_	P47710	36845	42847		166578	17724	44296	69998		47974											
[R].AAPGQEPPEHMAELQRNEQEQPLGQWH.[L]_	O00391	36651		63621		23370															
[K].QTDEIKDTR.[N]_	P47710	36400	17286	63397	225618	132404	87048	79792	197296	239038	239707			15575	31736	34964					
[C].HENDERPFYQKTAPYVPMY.[Y]_	P07498	36363	58834															29211			
[Y].NQLQLQAAHAQEQIRR.[M]_	P47710	36188	129603	93808		722763	595405	621749	447734	1598877	2047835	125680	78645	90459	233551	361967					
[L].YQRRPAIAINNPYVPR.[T]_	P07498	36150	41270	43755	31363	49149	116068	112242	180711	99179	81444										
[K].HATKTAKDALSSVQESQVAQQA.[R]_	P02656	36104		27335		27741	43999	69900	102636	82487	57790										
[A].HAQEQIRR.[M]_	P47710	36089				89579	70883	80384	111565	199369	233177										
[P].YGFLPQNILPLAQPAVVLPVPQPEIMEVPK.[A]_	P05814	36010	80843	52291		97292	436878	287945	324278	208553	237839	248360	231291	266604	230776	183403					
[E].GPEYWDRETQ.[KN]_	Q95604	35998			61963	16422															
[L].SRSGGGGGGGLGSGGSIRSSY.[S]_	P35527	35986	59038						267963		14123	16799	39340	44622	26685		34568	12593	75892	29611	13888
[K].GLRGAKTTFDVYTESWAQDPSQENK.[K]_	P19835	35740	28944	62689		154708	315046	55903	245179	878650	985158	14388		19153		74329					
[Q].GGGALRAPSQGSGAAARS.[-]_	Q9GZN4	35662	38228	43467																	
[E].SREEYMNGMNR.[Q]_	P47710	35640				47416	1309553	1335865	1595679	1498743	349294	1899375	1514637	1045076	1737831	979837	269234	699862	322069	69906	56279
[G].FLPQNILPLAQPAVVLPVPQPEIMEVPKA.[K]_	P05814	35564	34915	51709		35665	78964	117235	187620	124563		47752	57816	71084	52090	46608					
[R].DFIDIESK.[F]_	P06858	35545	29808		131250	40809	41746	52553		102616	103188	19444	20213	19584	22812						
[A].IPVAQDLNAPSDWDSRGK.[D]_	P10451	35446	30354	41390	62931	72341	669210	681583	607806	1557998	1973474	388405	446044	418845	472734	362758					
[G].AVYTEGGFVEGVNK.[K]_	P19835	35440	39279	70644	84645	22901	118962	131338	240237	169819	116053	50034	41738	56631	69431	21747					
[L].TDLENLHLPLP.[L]_	P05814	35421	55430	78964		60645	234356	265461		269836	467345	195672	228691	247567	305744	265697					
[P].QRQYLPNSHPPTVVR.[R]_	P07498	35292	46199	37020		16467	34830	30681		34406	10298	33876	34932	37131	29884	19992					
[A].NPAVVRPHAQIPQRQYLPNSHPPTVV.[R]_	P07498	35268	44967	53609		52429	93351	133307	151358	106531	107969	109772	107907	92731	58260	70422					
[E].SQDAGIKTITMLDEQK.[E]_	Q99541	35229	46670	39842		47019															
[Q].YPDATDEDITSHMESEELNGAYK.[A]_	P10451	35203	25990			43604	73878	94412	165006	119086	94389	124766	120775	175173	138296	64754					
[P].LAQPAVVLPVPQPEIMEVPK.[A]_1xOxidation [M16]	P05814	35195				41900	35583	35659		67176	116974										
[S].DWLDETNKGTKT.[E]_	Q96SI9	35157	29825	26991	47561																
[F].DPQIPKLTDLENLHLPLPLLQPLMQQVPQPIP.[Q]_	P05814	35004	116969	46239		9917	257094	207516	121612	422293	481095	1186029	1066628	1186291	911343	630542					
[I].YPVTQPLAPVHN.[P]_	P05814	34798	33416		42645	15296	57814	56928		40958	37894	36652	43852	50431	68819	59025					
[Q].TLALPPQPLWSVPQPK.[V]_	P05814	34459		28569	47573	242271	374321	465217	204017	708268	1281266										
[M].ESSISSSSEEMSLSK.[C]_	P47710	34390	30194	41351	137107	51490	59169	80901	101009	206517	112122	26469	30819	30063	73097	36036					
[R].DQADGSRASVDSGSSEEQGGSSRALV.[S]_	P01833	34218	86572	31311		14197						12485	59333	77400	48196	24567					
[S].EPIPLESREEYMNGMNR.[Q]_	P47710	33652			56374	128449	2082576	2111931	1406344	2141496	1982737	2805486	3048985	3289801	2248377	2214857	80685	87306	88173	39721	100991
[Q].PSTQIVANAKGAVTG.[A]_	Q99541	33243	50247	35824	159077	22951						25581	35643	73883	48008	15362					
[K].KVSPAVLVHRDGREQEAEQMPEY.[R]_	Q13410	33221	36076			62092	84771	58137		119092	196497					19595					
[A].PQKMAGASPTKDDSKDSDFWK.[M]_	Q13438	33209		37525		67744	22175	97977	188845	88372	77504										
[S].VDSGSSEEQGGSSRALVSTL.[V]_	P01833	33149	32366	37365	58309	15472															
[Y].NGFYYSNSANDQNLGNGHGK.[D]_	Q96S86	32850		82423		55136	94435	66384		82359	88785										
[L].ENLHLPLPLLQPLMQQVPQPIPQTL.[A]_	P05814	32692	43651	54259		172818															
[L].PPQPLWSVPQPK.[V]_	P05814	32555	40158	88088	102612	351564	1832987	1699360	2548626	3090360	5401953	23728	30904	20514	58781	110872					
[T].SSTSYNRGDSTFESKSY.[K]_	P02671	32458		49523		34396		32491		25613	29523										
[V].DFETDVLFLVPTEIALAQHR.[A]_	P19835	31903	76665				633613	713572	671005	383117	781051	68416		83513	62279						
[L].DKLTVTSQNLQLENL.[R]_	P04233	31692	33101	70589	74928	66645															
[D].ATDEDITSHMESEELNGAYK.[A]_	P10451	31594		34339		53273	130096	97016	163711	108380											
[T].DEIKDTRNESTQN.[C]_	P47710	31521	23011		118891	12070															
[K].ITHRIHWESASLLR.[S]_	P01024	31464	74975	79035		39719															
[F].FDPQIPKLTDLENLHLPLPLLQPL.[M]_	P05814	31372	30649	45674		212199	269583	268514	55045	334147	457324						50520	75580	45486	27540	595949
[Y].YANPAVVRPHAQIPQ.[R]_	P07498	31347	31357	68759		24249	75620	89239	76298	58527	107324	45051	48984	37274		25024					
[P].YEKVSAGNGGSSLS.[Y]_	P15941	31243	30048		184121	16958	19400	17282	176253	46054	14331						22687	22500			
[D].EDITSHMESEELNGAYK.[A]_	P10451	31056	24494	32253		44894	967264	768958	518892	897013	449153	101094	65660	126633	162624	185943					
[R].GDSVVYGLRSKSKKF.[R]_	P10451	29908	67334	53274		18464															
[T].IPFFDPQIPK.[L]_	P05814	29875	292860	403056	386233	640592	7643726	7363497	3122098	7617879	12698365		135584	110713	70200	120678					
[P].DATDEDITSHMESEELNGAYK.[A]_	P10451	29799	61939	49013		78125	189685	172307	290379	183742	52784	57575	48159	46053	25662						
[Q].GEDEHQDK.[I]_	P05814	29674	27021	35990		44256	47651	69147	212540	93625	27814	23839	38707	28927	20622	14432					
[L].PQNILPLAQPAVVLPVPQPEIMEVPKAKDTVYT.[K]_	P05814	28991	97319	66892		226261	203433	314472	1978308	382921	624892										
[K].GIPFAAPTKALENPQPHPGWQGTLKAKNF.[K]_	P19835	28760	38673				199597	233162	447875	174238	83012	15208	119475	172975	28492	40976					
[N].LHLPLPLLQPLMQQVPQPIPQTLALPPQPLWSVPQPK.[V]_	P05814	28724	32243	32770		203952															
[S].ITEYKQKVEK.[V]_	P05814	28598	35366			44889	50973	94890	117805	273946	86926	27838	30767	56108	77664	47352					
[K].IYPSFQPQPLIYPFVEPIPYGFLPQNIL.[P]_	P05814	28530	30148			24882															
[H].GRYVPPSSTDRSPYEKVSAGNGGSSLSY.[T]_	P15941	28089	58815	47476		15734	57143	75050	127777	19070	18830	37684	45127	21605	32999	28325					
[K].GRVMPVLKSPTIPFFDPQIPKLTDLENLHLP.[L]_	P05814	28009				88806															
[L].SPWVIQK.[N]_	P19835	27998				28170	124245	123354	120836	91989	117498	40491	53476	38229	45521	63149					
[D].LENLHLPLPLLQPLMQQVPQPIPQT.[L]_	P05814	27787	87969	102909		265844	906537	686735	775571	886115	903460	938519	679788	1076487	1713328	4017685					593690
[L].NEYNQLQLQAAHAQEQ.[I]_	P47710	27560	34096	38561	207297	236435	28735	41061	48058	119694	363866				30207	62208					
[K].LTDLENLHLPLPLLQPLMQ.[Q]_	P05814	27493	58454	37019		80011	554334	469934	161145	240038	354694	1014874	847355	1074029	903128	666451		57047	30064	27472	132870
[V].EQAVETEPEPELR.[Q]_	P02649	27102			292280	35799	98750	119230	101154	260218	100605										
[K].RGPGTPAPTGLSAPLSFIPR.[H]_	Q6WN34	26979	30206	103297		218587	361207	358945	459784	634508	573606										

Quantified modified peptide sequence	Protein ID	A_-80oC	A_-20oC_120h	A_4oC_6h	A_RT_6h	A_RT_24h	B_-80oC	B_-20oC_120h	B_4oC_6h	B_RT_6h	B_RT_24h	C_-80oC	C_-20oC_120h	C_4oC_6h	C_RT_6h	C_RT_24h	D_-80oC	D_-20oC_120h	D_4oC_6h	D_RT_6h	D_RT_24h
[R].HQGVMVGMGQKDSYVGDEAQS.[K]_2xOxidation [M5; M8]	P60709	25604	28908			17554		27126		26055	21597										
[V].PQPIPQTLALPPQPLWSVPQPK.[V]_	P05814	25416		25621		27809	80431	159985	1839200	441958	500793										
[G].EDEHQDKIYPSFQPQP.[L]_	P05814	25119	46044	66222	108777	85410	101839	156771		136354	221307				31054	32180					
[P].AVVLPVPQPEIMEVPKAKDTVYTK.[G]_	P05814	25096	65472			140320	209852	205557	333051	252009	145033	113510	127479	130270	123599	100179					
[S].NPTAHENYEKNNV.[M]_	P47710	24879	20349	21838	90199	13298															
[K].VKHEDQQQGEDEHQDKIYPSFQPQPLIYP.[F]_	P05814	24808		81503		27105	244447	240966	339138	283970	201211	162414	200023	146524	140823	97204					
[K].DTVYTKGRVMPVLKSPTIPFFDPQIPK.[L]_	P05814	24067		47518		351900	271296	246768	269717	682659	366578	35282	23527	25484	54428	136278					
[A].APTKALENPQPHPGWQGTLK.[A]_	P19835	23824				49830	832429	856947	939483	835381	490110	394764	404813	399611	346008	226585		41493			
[S].PTIPFFDPQIPKLTDLENL.[H]_	P05814	23778	26113			26374	146015	204782	247027	122486	353624	29961	25240	35293	93078	271163					
[A].LENPQPHPGWQGTLKAKNF.[K]_	P19835	23722	21668	19603	67226	20847	20716			47159	72688										
[R].SGRDPNHFRPAGLPEKY.[-]_	P0DJI9	22672	44271																		
[F].PPFSDISNPTAHENYEKNNVMLQW.[-]_	P47710	22415				26664															
[A].DGSRASVDSGSSEEQGGSSR.[A]_	P01833	21752	15976		112379		106197	141321	142278	81780	7270	410808	427086	442229	335578	80517	155402	141284	161696	99103	99258
[F].DPQIPKLTDLENLHLPLPLLQPLMQQVPQPIPQ.[T]_	P05814	21706	107576	41984		17775	96789		410878	92346	74698	760680	657844	771150	565270	421978					
[V].PKAKDTVYTKG.[R]_	P05814	20661				15804	19521	23096		33901											
[P].IPLESREEYMNG.[M]_	P47710	20429				28966	218122	290110	193038	233463	316215	265153	296234	300301	206067	144842					
[K].MESSISSSSEEMSLSK.[C]_	P47710	19599				20319	45653	57591		115378	39147	31101	24838	23231	30831	22109					
[S].PTIPFFDPQIPKLTDL.[E]_	P05814	19495	33949			29446	120807	77942	309416	129278	209509			10571	14116	134986		8892	20012	21246	154335
[L].FAEEKAVADTRDQA.[D]_	P01833	19172	141684			12312		115704		15674	23359										
[S].EETKENEGFTVTAEGK.[G]_	P01024	17829				37620	16108			26946	124129										
[-].MDEDVLTTLKILIIGESGVGK.[S]_1xAcetyl [N-Term]	Q9NP72						90168	87591	116782	89455	73140										
[-].MDKQNSQMNASHPETNLPVG.[Y]_1xAcetyl [N-Term]	O15162											103033	91380	104876	91424	50483	20141	17914	23576		
[-].MDNLSSEEIQQRAH.[Q]_1xAcetyl [N-Term]	O00161											55740	75448	65577	49093	37853					
[-].MDNLSSEEIQQRAHQIT.[D]_1xAcetyl [N-Term]	O00161											100880	122911	113726	94323	47746					
[-].MDNLSSEEIQQRAHQITD.[E]_1xAcetyl [N-Term]	O00161											58205	72800	58814	51381						
[-].MEAARDYAGALIR.[P]_1xAcetyl [N-Term]	O60543						45987	33235				78491	88360	65757	89883	45290					
[-].MEAARDYAGALIRPLTFMGSQT.[K]_1xAcetyl [N-Term]	O60543						59554		179000		73814			56399		34484					
[-].MEAARDYAGALIRPLTFMGSQTK.[R]_1xAcetyl [N-Term]	O60543			120971								163880	223888	285185	225542	170611					
[A].AAVSGAQPILSKLEPQIASASE.[Y]_	O60664											39481	43118	39661	30591						
[A].ADQRRDFIDIESK.[F]_	P06858						390892	447601	462460	789747	212473										
[A].AEDDEDDDVDTKK.[Q]_	P06454						30770														
[A].AKLGAVYTEGGFVEGVN.[K]_	P19835						117947	125674	72378	147138	125924						21760	33206	22031		
[A].AKLGAVYTEGGFVEGVNKKLG.[L]_	P19835																	46207			13679
[A].AKLGAVYTEGGFVEGVNKKLGLLGDSVDIF.[K]_	P19835									145243	177591										
[A].APAPGLGAAAAAPGALVRDV.[H]_	Q8NES3																56008	49997	45339	55664	
[A].APDEKVLDSGFR.[E]_	P01833											22721	25403		12285						
[A].APGQEPPEHMAELQ.[R]_	O00391											44419	36400	44131	45336	10722	33684	39859	27139	46841	17211
[A].APTKALENPQPHPGWQ.[G]_	P19835						49676	61670													
[A].APTKALENPQPHPGWQG.[T]_	P19835						78026	83848	73134	55135	47348										
[A].ASAYYNPGNPHNVYMPTS.[Q]_	Q969T9											17243	32341	31893		12860					
[A].DAVTLDGGFIYEAGLAPYK.[L]_	P02788						66483	75687		204252	459374										
[A].DEDSSRPVWLK.[A]_	P49327						39839	41059		43095	44838	37464	45946	40293	32082	19266					
[A].DGPERVTVIANA.[Q]_	Q13410											187445	241791	140336	204956	107376					
[A].DGSRASVDSGSSEEQGGSSRAL.[V]_	P01833											47774	56413		57247						
[A].DGSRASVDSGSSEEQGGSSRALVSTLVP.[L]_	P01833											100658	101335	114058	97445	52378					
[A].DGSRASVDSGSSEEQGGSSRALVSTLVPLG.[L]_	P01833											1551892	1665935	1814629	1665774	1140935					
[A].DHADDIQYVFGKPFATPTGYRPQD.[R]_	P19835						129200	150859		127976	64170										
[A].DIEEIKSGRLS.[K]_	P80303									29946	129765										
[A].DQRRDFIDIESKFA.[L]_	P06858					39357															
[A].DQRRDFIDIESKFAL.[R]_	P06858							51922	140865	217042	109872										
[A].DSGSSEEKQLY.[N]_	P10451											64548	62480	73282	111622	48086					
[A].DSGSSEEKQLYNKYPDAVATWLNPDPSQK.[Q]_	P10451											183936	159357	168093	162029	110701					
[A].DTLHSKL.[I]_	Q13410											36028	44978	66042	25772						
[A].DTLHSKLI.[P]_	Q13410						105315	102585	72894	87389	34946	233905	296928	213707	177438	75430					
[A].DTLHSKLIPTQ.[P]_	Q13410											1265754	1353997	1650675	1006636	474695					
[A].DTLHSKLIPTQPSQG.[A]_	Q13410						54030	56206		32046	12297	51762	56960	49490	46169	31906		17524			
[A].DTRDQADGSRASVD.[S]_	P01833						115683	82906		39984		57943	36771	100916	50104	34460					
[A].DTRDQADGSRASVDSGSSEEQGGSSR.[A]_	P01833						90219	163103	211988	48814		86493	80547	101711	105064	56316					
[A].DTRDQADGSRASVDSGSSEEQGGSSRALVSTLVP.[L]_	P01833											118064	140120	118891	88635	61885					
[A].DTRDQADGSRASVDSGSSEEQGGSSRALVSTLVPL.[G]_	P01833											103017	96634	38637	120898	141696					
[A].DTRDQADGSRASVDSGSSEEQGGSSRALVSTLVPLG.[L]_	P01833											276915	293263	283842	345764	152436					
[A].DTRKMDPSKPSSNVAG.[V]_	P22897																105602	143996	116015	77233	77310
[A].DTRKMDPSKPSSNVAGVVIIVI.[L]_	P22897											106953	120422	91601	75515	45985					
[A].EDDEDDDVDTKK.[Q]_	P06454						64191														
[A].EDVGSNKGAIIG.[L]_	P05067																80450	84970	72860	53540	25784
[A].EEDTNVYTEKHSDSLFK.[R]_	P34741						68691	56840		84902	62972										
[A].EEKAVADTRDQ.[A]_	P01833											70435	65876	64115	58719	31619					
[A].EEKAVADTRDQADG.[S]_	P01833																110515	100073	93981	68105	90937
[A].EKLPGQGVHSQGQGPGAN.[F]_	Q92896											182490	190643	131074	131877	26321	802225	1014568	795703	555475	388907
[A].ENFRALSTGEKGFGYK.[G]_	P62937							40222		67401	64525										
[A].EQMPEYR.[G]_	Q13410						80471	65861		42163											
[A].ESIRETEVIDPQDLLEGR.[Y]_	P31431						20036	55108	60454	27537	38880										
[A].ETHSHKQSRLY.[K]_	P10451						44874	34051		45143	9378	171251	177171	134373	174366	116489					
[A].GDDAPRAVFPSIVGRP.[R]_	P60709; P68133															27289					

Quantified modified peptide sequence	Protein ID	A_-80oC	A_-20oC_120h	A_4oC_6h	A_RT_6h	A_RT_24h	B_-80oC	B_-20oC_120h	B_4oC_6h	B_RT_6h	B_RT_24h	C_-80oC	C_-20oC_120h	C_4oC_6h	C_RT_6h	C_RT_24h	D_-80oC	D_-20oC_120h	D_4oC_6h	D_RT_6h	D_RT_24h
[A].GPAGAAPAPGLGAAAAAPGALVRDVHS.[L]_	Q8NES3																	18028	11306		
[A].GVVHGAIGGAGVTA.[L]_	P20138																30185	30264	23354	29694	11400
[A].HENYEKNNVML.[Q]_	P47710														25812	55077		26814			45552
[A].HPHLFLYEDSK.[S]_	Q13410							23154		47720	10748										
[A].IESQDAGIKTITMLD.[E]_	O00161											72187	77633	44070	52705	25071					
[A].IPVAQDLNAPS.[D]_	P10451						384750	463400	186031	165601	76650	184492	168801	182143	127322	74628					
[A].IPVAQDLNAPSD.[W]_	P10451						344969	304871	74700	229263	152778										
[A].IPVAQDLNAPSDWDSRGKDSY.[E]_	P10451											47857	66900	41199	27703	28659					
[A].IPVAQDLNAPSDWDSRGKDSYETSQ.[L]_	P10451											43213	41989	56508	36244	30242					
[A].IPVAQDLNAPSDWDSRGKDSYETSQL.[D]_	P10451											87515	76933	87568	57067	48752					
[A].IPVAQDLNAPSDWDSRGKDSYETSQLD.[D]_	P10451											55932				21450					
[A].IPVKQADSGSSEE.[K]_	P10451											18089	19763	29322	16904	17910					
[A].IPVKQADSGSSEEKQ.[L]_	P10451											23570	35631	28079		28358					
[A].IPVKQADSGSSEEKQL.[Y]_	P10451											35670	43735		32336	24762					
[A].IPVKQADSGSSEEKQLY.[N]_	P10451											1886656	1761385	1777974	1781376	1389073					
[A].IPVKQADSGSSEEKQLYN.[K]_	P10451						35352			60148	49901	207987	221641	191217	178420	215159					
[A].IPVKQADSGSSEEKQLYNKYPDAVATWLNPDPSQ.[K]_	P10451											1479319	1723991	1506775	1406062	675899					
[A].IQDPRLFAEE.[K]_	P01833																18582	28805	20629	21854	
[A].IQDPRLFAEEK.[A]_	P01833						107049	105564	206726	112799	17430										
[A].IQDPRLFAEEKAVADT.[R]_	P01833					48471	143825	200025	96304	598572	431489										
[A].IQDPRLFAEEKAVADTRDQ.[A]_	P01833											83031	60990	55671	45911	77540	35723	32833	34511	30624	21300
[A].IQDPRLFAEEKAVADTRDQADG.[S]_	P01833											30151	31426	23919	21608	31890					
[A].KDTVYTKGRVM.[P]_	P05814									29744											
[A].KDTVYTKGRVMP.[V]_	P05814											44819	65054	51345	75598	58176					
[A].KDTVYTKGRVMPV.[L]_	P05814						24535			72662	40528										
[A].KDTVYTKGRVMPVL.[K]_	P05814																	23369			137655
[A].KDTVYTKGRVMPVLKSPTIP.[F]_	P05814															21698					
[A].KLGAVYTEGGFVEGVN.[K]_	P19835						134719	101129	221994	187061	100833						14727	16629	17450		
[A].KLGAVYTEGGFVEGVNK.[K]_	P19835						1287849	1234074	1567695	1547487	1304799		53355	82074	82572	16891					
[A].KLGAVYTEGGFVEGVNKKLG.[L]_	P19835											21526									
[A].KPALEDLR.[Q]_	P02647							21373		21092	19147										
[A].KPALEDLRQGLLPVLESFK.[V]_	P02647							30523		41682	58972										
[A].KTTFDVYTESWAQDPSQENK.[K]_	P19835						177425	236093	193393	775017	726912										
[A].KVEQAVETEPEPEL.[R]_	P02649						33906	59508	83477	57098	37245										
[A].KVEQAVETEPEPELR.[Q]_	P02649						105792	104843	245013	190204	88106										
[A].LENPQPHPGWQG.[T]_	P19835						81768	73006		76030	80764										
[A].LENPQPHPGWQGTL.[K]_	P19835						134409	104408		64038	96619										
[A].LENPQPHPGWQGTLK.[A]_	P19835									50625	104578										
[A].LGSDPHISMQVQE.[N]_	Q13410						80199	56687		52240	61198										
[A].LLLNQELLLNPTH.[Q]_	P05814						58902	62062	92737	56538	56816	23285		40040	58928	61854					
[A].LLLNQELLLNPTHQ.[I]_	P05814						35191	46777				18383	33185	32614	21328	25278					
[A].LLLNQELLLNPTHQIYPVTQ.[P]_	P05814						227787	196591	708331	316722	723297	62039	77841	104154	295647	504736					
[A].LLLNQELLLNPTHQIYPVTQPLAPVH.[N]_	P05814											39131	111840		96125	203020					65881
[A].LLTQQTELQSLR.[R]_	O75888																52595	100745	43665	22452	
[A].LPGDNVGFNVK.[N]_	P68104						41036	31085	55517	26881	26337										
[A].LPIIQKLEP.[Q]_	Q99541											26876	33192	16468	12472						
[A].LPIIQKLEPQIAVA.[N]_	Q99541											64088	66446	78984	55672	28178					
[A].LPIIQKLEPQIAVANT.[Y]_	Q99541											141481	152901	170537	138688	113890					
[A].LPIIQKLEPQIAVANTY.[A]_	Q99541						380052	352293		338991	164499	1330192	1365887	1525086	1254722	867359					
[A].LPPGAFAGAGALQRLD.[L]_	Q2I0M4												38443	40485	58053		45644	48421	39276	33613	23671
[A].LPPQPLWSVPQPK.[V]_	P05814		171141		606585	716994	3447317	3606873	2059038	5814009	11689258										
[A].LPPQPLWSVPQPKV.[L]_	P05814				105490	384996	239203	242129		267438	514154	343314	370474	461192	1769289	5757475		12546			495689
[A].LPPQPLWSVPQPKVLPIPQQV.[V]_	P05814											95602	83233	77375	91233	67030					
[A].LPPQPLWSVPQPKVLPIPQQVVPYPQRAVPVQA.[L]_	P05814						581221	522761	577978	432217	433302	1121470	1071303	857626	1227125	5038656					
[A].LPPQPLWSVPQPKVLPIPQQVVPYPQRAVPVQALL.[L]_	P05814											77250	113447	100017	69279	168130					
[A].MTSALPIIQKLEPQIA.[V]_	Q99541											51691	47996	32887	29293						
[A].NAKGAVTGAKDAVTT.[T]_	Q99541											21881	29572	25715	23285						
[A].NAKGAVTGAKDAVTTT.[V]_	Q99541											34032	28083	29291							
[A].NAKGAVTGAKDAVTTTVT.[G]_	Q99541						104778			59381	36238	654752	677117	604911	558783	295893	46778	65182	72190		
[A].NAKGAVTGAKDAVTTTVTGAK.[D]_	Q99541											28534	35251	31926	18864	11053					
[A].NAKGAVTGAKDAVTTTVTGAKDS.[V]_	Q99541											63177	62315	72948							
[A].NAKGAVTGAKDAVTTTVTGAKDSVA.[S]_	Q99541											129249	138912	112935	89707	60586					
[A].NAKGAVTGAKDAVTTTVTGAKDSVASTIT.[G]_	Q99541												32189		35557						
[A].NAKGAVTGAKDAVTTTVTGAKDSVASTITGVMD.[K]_	Q99541											65867	71446	56645	48182	34404					
[A].NDESNEHSDVIDSQELS.[K]_	P10451											17719				35214					
[A].NDESNEHSDVIDSQELSK.[V]_	P10451						29958	35023				32421	36746	33304	33026	21948					
[A].NDESNEHSDVIDSQELSKVS.[R]_	P10451											472440	426109	469497	413789	204870					
[A].NDESNEHSDVIDSQELSKVSREF.[H]_	P10451											111909	86934	110077	72362	55267					
[A].NDLEDKNSPFYYDWHSLQ.[V]_	Q14802																	18461			
[A].NPAVVRPHAQIPQ.[R]_	P07498		37751	57356		40649	291344	325144	192714	185484	320692										
[A].NPAVVRPHAQIPQRQYLPNS.[H]_	P07498											290884	320701	287429	194637	148754					
[A].PGQEPPEHMAELQRNEQEQPLGQWHLS.[K]_	O00391						73048	73992										39210	21277		32040
[A].PSDWDSRGK.[D]_	P10451						36240	37487		50260	58307	189545	163232	156456	207760	103806					
[A].PSDWDSRGKDS.[Y]_	P10451						82171	105459	79609	63374	39747	372835	406619	416343	378408	229597					

Quantified modified peptide sequence	Protein ID	A_-80oC	A_-20oC_120h	A_4oC_6h	A_RT_6h	A_RT_24h	B_-80oC	B_-20oC_120h	B_4oC_6h	B_RT_6h	B_RT_24h	C_-80oC	C_-20oC_120h	C_4oC_6h	C_RT_6h	C_RT_24h	D_-80oC	D_-20oC_120h	D_4oC_6h	D_RT_6h	D_RT_24h
[A].PTGLSAPLSFIPRHF.[R]_	Q6WN34									42247	28795										
[A].PTKALENPQPHPGWQG.[T]_	P19835						49769	40338		57183	31258										
[A].PTKALENPQPHPGWQGTL.[K]_	P19835			25795		42697	192849	166985	203718	178967	285083										
[A].PVHNPISV.[-]_	P05814						488539	445060	205254	518957	916554	241563	315991	377016	1226126	3047337					
[A].QDLSKEIPLSPMGEDSAPRDADTLH.[S]_	Q13410											94283	72182	79472	74508	26421					
[A].QLGGPEAAKSDETAAK.[-]_	P04792											14527	16977	21181	27026	12808					
[A].QPAVVLPVPQPEI.[M]_	P05814						66802	51721		35453		33665	21588	34704	62047	69322					
[A].QPAVVLPVPQPEIMEVPKAK.[D]_	P05814						137128	153738	144702	113528	86222	375227	318542	255933	301983	257329					
[A].QPAVVLPVPQPEIMEVPKAKDTVYT.[K]_1xOxidation [M14]	P05814						132228	100615	317233	43188	33302	66078	109602	84065	78166	68902					
[A].QPAVVLPVPQPEIMEVPKAKDTVYTK.[G]_	P05814					58672	220008	189953	706040	232145	95752	563558	463389	413701	466724	463012					
[A].QPGQSQVSYQGLPVQK.[T]_	Q14766											100469	111315	109028	75279	44169					
[A].RETIESL.[S]_	P05814						881093	602778	222017	1592570	381673										
[A].RETIESLSSSE.[E]_	P05814						63349	33913		39443	18346	22126	15121	14654	33285	44295					
[A].RETIESLSSSEE.[S]_	P05814						48085	48145		54818					28795	28797					28872
[A].RETIESLSSSEESI.[T]_	P05814															35671					37829
[A].RETIESLSSSEESIT.[E]_	P05814															89032					
[A].RETIESLSSSEESITE.[Y]_	P05814											58858	62919	45017	37975	21077	118328	102856	126292	68695	65122
[A].RPKLPL.[R]_	P47710						3294121	2986140	2548249	3275316	2116347	45940	45457	30819	58396	27657					
[A].RPKLPLR.[Y]_	P47710						40335	38567		37599	9019										
[A].RPKLPLRY.[P]_	P47710						75559	61096	124166	88455	40137										
[A].RPKLPLRYPERLQNPSESSEPIPLE.[S]_	P47710						74941	100779	233537												
[A].SASEYAHRGLDKLEENLPIL.[Q]_	O60664											42840	48511	35156	44809	27939					
[A].SASKFPSSGPVTPQPTALTF.[A]_	Q9Y679											55216	60892	50856	40822	25940					
[A].SAYYNPGNPHNVYMPTS.[Q]_	Q969T9											48179	53217		28575	16418					
[A].SEAEDASLLSFMQGYMKHATKTA.[K]_	P02656		24788			33881															
[A].SEYDYVSFQSDIGPYQSGR.[F]_	Q8N474							33093	93081			44221	46590	159253	54172	24197					
[A].SPTKDDSKDSDFWK.[M]_	Q13438							52805		84415	63884										
[A].SRELPPPPPPP.[P]_	B1AK53						33546	45551		17657	11347	33911	39765	35679	39454	17924					
[A].SSIIDELFQDRFFTREPQDTYHYLP.[F]_	P10909										209869										
[A].STITGVMDKTKGAVTG.[S]_	Q99541											141087	218305	217998	123674	71774					
[A].SVAVDPQPSVVTRVVN.[L]_	Q99541													32775	28302						
[A].SVDSGSSEEQGGSSRAL.[V]_	P01833																	24379	26915		24438
[A].SVDSGSSEEQGGSSRALVS.[T]_	P01833						26742	38970		34342	16244							22445	18083		9639
[A].SVDSGSSEEQGGSSRALVSTLVPLG.[L]_	P01833						22935428	23288544	26736365	16300068	8905151						20554128	16099529	17679998	20333603	11051529
[A].TIGGVAWIGGKSLE.[V]_	Q8WUH6											22754	31574	35101	19509						
[A].TLVQDGIAKG.[R]_	Q13410																34348	60038	59907		
[A].TPRYDSKNGTLV.[-]_	Q96A22											349244	390714	326720	319787	192154					
[A].TQLSEAVDATRGAVQ.[S]_	O60664											43042	44052	24705	28091	25689					
[A].TSEVSPNSKPSPNT.[K]_	P12272				145080	11049	11752	17788		61238	47904										
[A].TVGSLAGQPLQERAQAWGERL.[R]_	P02649						36974	40210	56653												
[A].VADTRDQADGSRASVD.[S]_	P01833						50704	45111		25068		24875	19768	23508	22300	18066					
[A].VADTRDQADGSRASVDSGSSEEQGGSSRALVSTLVPLG.[L]_	P01833											659114	775451	762805	629514	314924	233740	216839	214834		113626
[A].VDPQPSVVTRVVN.[L]_	Q99541											640703	705598	645558	569759	335991	76705	72669	86955	57053	26806
[A].VDPQPSVVTRVVNLPLVS.[S]_	Q99541											181952	241893	265394	224677	144038		22764			
[A].VEVQNQKQPACHENDERPFYQ.[K]_	P07498							44198			76346										
[A].VGYVDDTQFVRFDSDAASPR.[G]_	Q07000; P30510																	14083			7249
[A].VLVHRDGREQEAEQMPEY.[R]_	Q13410						57928	58935	85487	68310	80108										
[A].VLVHRDGREQEAEQMPEYR.[G]_	Q13410						64322	144556	128204	59814	28938										
[A].VPVQALLNQE.[L]_	P05814					25844	48422	55963		33493	54067	42162	52137	36877	23888						
[A].VPVQALLNQELL.[L]_	P05814												53568								
[A].VPVQALLNQELLLNPT.[H]_	P05814						1132093	1031445	731240	667410	317059	3449001	3196772	3336769	3082775	1978801					
[A].VPVQALLNQELLLNPTHQ.[I]_	P05814			203138	59278	118634	792381	749384	383684	541814	429211	587882	588246	647177	672477	553299					
[A].VPVQALLNQELLLNPTHQIYP.[V]_	P05814						1412348	1260570		841382	696998										
[A].VPVQALLNQELLLNPTHQIYPVTQPLAPVHN.[P]_	P05814											375668	180844	317119	339453	257162					
[A].VTYVYFTNELKQMQDKYSK.[S]_	P50591									59660											
[A].VVLPVPQPEIMEVPKA.[K]_	P05814									273369	430449										
[A].VVLPVPQPEIMEVPKAKDT.[V]_	P05814																59185	68824	49589		51830
[A].VVRPHAQIPQR.[Q]_	P07498						38888	50131	136470	69815	166179										
[A].VYTEGGFVEGVNKKLGLLGDSVDIFK.[G]_	P19835						79818	52094		80708											
[A].WLDSGVTGSGLEGDH.[L]_	P12272																27162	30762	33410		
[A].YKAIPVAQDLN.[A]_	P10451																	25687			
[C].EMAENGVKTITS.[V]_	Q99541											86212	60861	116449	90069						
[C].EMAENGVKTITSVAMTS.[A]_	Q99541											43194	57265	57807	28674						
[C].EVWTQRLHGGSAPLPQDRGF.[L]_	Q92673																33835	67255	27321		
[C].HENDERPFYQ.[K]_	P07498						83895	122087		100619	100134	94031	53199	83336	71914	29788					
[C].RLNEYNQLQLQAAHAQEQIR.[R]_	P47710						91714	94018	72257	164848	219404										
[C].VVAEPEKMESSISSSSEEMSLS.[K]_	P47710											41587	48281		48788	16823					
[D].AAPDEKVLDSGFREIENK.[A]_	P01833						60465	82913	158822	128630	205186										
[D].ADTLHSKLI.[P]_	Q13410																45340	49567	38325	19985	
[D].ADTLHSKLIPT.[Q]_	Q13410											208195	250687	189723	195822	83816	38303	51772	33270	44812	10076
[D].ADTLHSKLIPTQPSQ.[G]_	Q13410											38507	42388	40286							
[D].APPPPAAPLPRWSGPIG.[V]_	Q99523											312632	364916	332423	275070	170657	263022	262773	231594	246197	127377
[D].DIQYVFGKPFATPTGYRPQD.[R]_	P19835											13338		16248		22484					
[D].DLYSTIKV.[-]_	P33121											93089	110947	121261	91823	96027					

Quantified modified peptide sequence	Protein ID	A_-80oC	A_-20oC_120h	A_4oC_6h	A_RT_6h	A_RT_24h	B_-80oC	B_-20oC_120h	B_4oC_6h	B_RT_6h	B_RT_24h	C_-80oC	C_-20oC_120h	C_4oC_6h	C_RT_6h	C_RT_24h	D_-80oC	D_-20oC_120h	D_4oC_6h	D_RT_6h	D_RT_24h
[D].DPDAPLQPVTPLQ.[L]_	P0C0L4											119181	124701	130764	128209	48505					
[D].DQSAETHSHKQS.[R]_	P10451											221835	243803	108586	178563	152620					
[D].DQSAETHSHKQSRLY.[K]_	P10451					16969				17251		386562	507929	362342	307022	209519					
[D].EDITSHME.[S]_	P10451						1871628	1499366	303303	1638364	433202	294160	201466	224980	384252	222578					
[D].EDITSHMESE.[E]_	P10451											30695		26741	38871	37610					
[D].EDITSHMESEELNGAY.[K]_	P10451						515951	299242	159307	425900	425029	110443	76616	130754	93469	70402					
[D].EERQGPPLGGQQ.[S]_	P02810																	28535			
[D].EPPQSPWDRVKD.[L]_	P02647											152683	184267	180129	146563	86121					
[D].ERPFYQK.[T]_	P07498						123565	119238	230037	190607	99219										
[D].GPERVTVIANAQDLS.[K]_	Q13410											133596	154489	180638	130895	86856					
[D].GREQEAEQMPEYR.[G]_	Q13410						233853	297684	259570	238717	127552	189587	141583	129042	149200	98190	328264	327670	398756	212555	268110
[D].GREQEAEQMPEYR.[G]_1xOxidation [M9]	Q13410																	6561		29978	13378
[D].GREQEAEQMPEYRG.[R]_	Q13410					16012	131027	178907	189182	163856	114202	30482	31584	33376	31953	25343					
[D].GSYEEALVHLK.[V]_	Q13410						23575			63039	71965										
[D].IQYPDATDEDITSH.[M]_	P10451						89087	112225		43335	34927	499427	478623	359376	314052	137871					
[D].IQYPDATDEDITSHMESEELNGAYK.[A]_	P10451											409690	407351	442095	487446	502214					
[D].ISNPTAHENYEKNNVMLQW.[-]_	P47710											88396	37415	90935	50334	32493	19573	25176	29931		16907
[D].KIYPSFQPQP.[L]_	P05814						53970		99753	40462	33339										
[D].KIYPSFQPQPLI.[Y]_	P05814						895967	577589	2348891	385297	172035	506206	495259	486324	671481	455534	75339	26196	101211	111142	114744
[D].KIYPSFQPQPLIY.[P]_	P05814						39434	29876	112512	41414	43962	28149	33929	29538	33979	26232					
[D].KIYPSFQPQPLIYPFVE.[P]_	P05814						257863	214586	631095	265266	217299		17876		49380	41187					
[D].KIYPSFQPQPLIYPFVEPIPYGFLPQNI.[L]_	P05814																22776				46070
[D].KTKGAVTGSVEKTKSVVSG.[S]_	Q99541											23697	59524	29429	36722	37137					
[D].LENLHLPLP.[L]_	P05814						277899	210612	188975	300286	448827	173328	154840	131828	263861	290466					
[D].LENLHLPLPLL.[Q]_	P05814						291976	198376		276962	321963	79850			242749	222121					
[D].LENLHLPLPLLQ.[P]_	P05814						455005	313688	336844	353655	774282	102229	108127	117517	265371	289923					72210
[D].LENLHLPLPLLQPLM.[Q]_	P05814						430552	248862	526993	205295	223378	140376	181145	267132	619947	596010					227070
[D].LENLHLPLPLLQPLMQ.[Q]_	P05814						212995	134874	367831	172527	141401	93808	92992	161474	395785	432899					255239
[D].LENLHLPLPLLQPLMQQ.[V]_	P05814												14533	18084	25169	54411					
[D].LENLHLPLPLLQPLMQQVP.[Q]_	P05814											59305	54784	50259	35411	16684					
[D].LENLHLPLPLLQPLMQQVPQPIPQTL.[A]_	P05814					256317	449581	417273	465232	349190	476550	230536	57640	308105	1115898	3845669					1104641
[D].LENLHLPLPLLQPLMQQVPQPIPQTLAL.[P]_	P05814						55483	43362		26309		64233	77129	42771	120987	377287					124833
[D].LMSSAYLSTKDQYPYLK.[S]_	Q99541											44058	44478	43802	44877	16166					
[D].LNAPSDWDSRGK.[D]_	P10451											21888		26114	23779	16501					
[D].LNAPSDWDSRGKDS.[Y]_	P10451											83397	89610	24084	26795	39646					
[D].LPAEPAAARGGGGGGGGGGGGRGPQA.[R]_	Q86Y38						50731	59432	74656	37205	23002										
[D].MLVVDPKSKEED.[K]_	P10451						46712	64756		58013	37284										
[D].PDTAHPHLF.[L]_	Q13410											22892	31438	40132	35451	18974					
[D].PDTAHPHLFLYEDSK.[S]_	Q13410											25466		20956							
[D].PQIPKL.[T]_	P05814						38603	32672	67724	20506	47976	46950	67348	76809	93037	130611					
[D].PQIPKLTD.[L]_	P05814						43762	43283	120216	27478	37396	32072	45121	54836	85565	330639					
[D].PQIPKLTDLEN.[L]_	P05814								84184	59729	70355										
[D].PQIPKLTDLENL.[H]_	P05814																				43010
[D].PQIPKLTDLENLH.[L]_	P05814											26126	19199	28623	26178	18259					
[D].PQIPKLTDLENLHLP.[L]_	P05814		123900	117826		114262															
[D].PQIPKLTDLENLHLPL.[P]_	P05814					35220															
[D].PQIPKLTDLENLHLPLP.[L]_	P05814		35624	39765	46401	303732	316497	359957	934784	636411	1265661	223521	123309	57415	167038	203089					
[D].PQIPKLTDLENLHLPLPL.[L]_	P05814														15163	31596					
[D].PQIPKLTDLENLHLPLPLLQPLM.[Q]_	P05814														36565	103191					
[D].PQIPKLTDLENLHLPLPLLQPLMQQVPQPIP.[Q]_	P05814												19323	20223	29474	14214					
[D].PQIPKLTDLENLHLPLPLLQPLMQQVPQPIPQT.[L]_	P05814											34208	51418	27767	45132	39250					
[D].PRLFAEEKAVADT.[R]_	P01833						105334	93532	83947	184770	326600										
[D].PRLFAEEKAVADTRDQ.[A]_	P01833											299080	408739	355659	355952	184703	456862	635104	505529	271520	252509
[D].PRLFAEEKAVADTRDQA.[D]_	P01833					25338	35633			32547	44229										
[D].QADGSRASVDSGSSEEQGGSSR.[A]_	P01833						80017	87320	97010	39695	5636	22004	25368	38052	30294	22861	12360	16454			7960
[D].QADGSRASVDSGSSEEQGGSSRALVST.[L]_	P01833						50447	79835		36585	37250										
[D].QADGSRASVDSGSSEEQGGSSRALVSTLVPLG.[L]_	P01833																197503	184723	216233	163365	241362
[D].QQQGEDEHQDKIYPSFQPQPLIYP.[F]_	P05814											75758	77847	67839	36896	18387					
[D].QRRDFIDIESK.[F]_	P06858									36775	27555										
[D].QSAETHSHKQSRLY.[K]_	P10451											64954	50528	71359	68356	35614					
[D].RLDEVKEQVAEVR.[A]_	P02649						28045	38294		16490											
[D].RSPYEKVSAGNGGSSLS.[Y]_	P15941								227012	38813	11214						31626	26924			
[D].RSPYEKVSAGNGGSSLSY.[T]_	P15941							39102	889444	23940		37023	52182	41967		27927	53451	37053			
[D].SAPRDADTLHSKLIPTQ.[P]_	Q13410											531776	571157	606338	452430	299108					
[D].SAPRDADTLHSKLIPTQPS.[Q]_	Q13410											69040	73279	76632	63664	27076					
[D].SDPITVTVPVEVSRKNP.[K]_	P10909											23888	27398	27751	19806	14429					
[D].SGSSEEQGGSSRALV.[S]_	P01833						96155	105137	54740	52117	11912	74567	81430	85437	111720	46801	168113	175571	186544	145860	73825
[D].SGSSEEQGGSSRALVS.[T]_	P01833						60882	42783		41395	9512										
[D].SGSSEEQGGSSRALVST.[L]_	P01833						202726	241933	170323	84954	61860	179919	188866	229730	197450	97974	54885	52472	71089	41409	24876
[D].SGSSEEQGGSSRALVSTLVPLG.[L]_	P01833						3333518	3304829	2807352	2567347	2282758	2701100	2852994	4025053	3936127	4128947	918504	813198	858909	1082419	915462
[D].SQELSKVSREF.[H]_	P10451											37618	36701	20235	38758	17339					
[D].SVDIFK.[G]_	P19835						41719	38123		99068	72592										
[D].TLHSKLIPTQ.[P]_	Q13410											49054	53918	45651	22013	15370					
[D].TLHSKLIPTQPSQGAP.[-]_	Q13410											31917	37995	35072	30872	20152					

Quantified modified peptide sequence	Protein ID	A_-80oC	A_-20oC_120h	A_4oC_6h	A_RT_6h	A_RT_24h	B_-80oC	B_-20oC_120h	B_4oC_6h	B_RT_6h	B_RT_24h	C_-80oC	C_-20oC_120h	C_4oC_6h	C_RT_6h	C_RT_24h	D_-80oC	D_-20oC_120h	D_4oC_6h	D_RT_6h	D_RT_24h
[D].TRDQADGSRASVDSGSSEEQGGSS.[R]_	P01833						23056	41959	58649	20160	10807	45485	49381	71664	68556	26748					
[D].TRDQADGSRASVDSGSSEEQGGSSRALVST.[L]_	P01833											31545	48197	48262	63019	16307					
[D].TVYTKGRVMP.[V]_	P05814				64131	33750						19925	25864	29456	62919	53928					
[D].TVYTKGRVMPVL.[K]_1xOxidation [M9]	P05814					88134	33470	30569	83443	72175	61635	13027		19680	37291	74756					
[D].TVYTKGRVMPVLKSP.[T]_	P05814											35159	46788	48434	48878	118754					
[D].TVYTKGRVMPVLKSPTIP.[F]_	P05814											182369	188194	193276	151153	173125					
[D].VYTESWAQDPSQENK.[K]_	P19835						104769	133959	102930	85681	155864	67413	110006	52559	55449	25007					
[D].WDSRGKDSYETSQL.[D]_	P10451											29182	33293	18176	35645	26407					
[D].WDSRGKDSYETSQLD.[D]_	P10451						356788	295890	105820	347069	180917										
[D].WDSRGKDSYETSQLDD.[Q]_	P10451											46858	32869	30134	62236	62145					
[D].WDSRGKDSYETSQLDDQSAETHSHKQS.[R]_	P10451											40978	35947	44374	33896	21873					
[E].AEQMPEYR.[G]_	Q13410						36475	32271		50340		42545	39293	15731	49901	19215	309923	273932	222269	90233	29816
[E].DEHQDKIYPS.[F]_	P05814						888907	1064033	706970	663209	108088	221862	302357	232709	124030	70435					
[E].DEHQDKIYPSFQPQPLI.[Y]_	P05814						745792	624097	272311	471196	288361										
[E].DEHQDKIYPSFQPQPLIYP.[F]_	P05814						64617	64612		52797		58359	50134	35032	42432	28335					
[E].DEHQDKIYPSFQPQPLIYPFVEPIPY.[G]_	P05814										34267										
[E].DENFILK.[H]_	P62937						77226	96291		86733	109474										
[E].DEVERVITIMQNPRQY.[K]_	P62269						48471	41957					14703	26454	14817						
[E].DFYKLVSEFTITK.[G]_	P19835						146700	156687		221617	485050										
[E].DFYKLVSEFTITKG.[L]_	P19835						92455	51801													
[E].DFYKLVSEFTITKGLRGA.[K]_	P19835						41224	79535	45685		18023										
[E].DGESSGANDSLRTPEQGSNGTDGA.[S]_	Q12913						81001	103481	157517	57397	24487										
[E].DGPKFLKSGDAAIVD.[M]_	P68104								196508			101785	130470	103046	77472	52437					
[E].DGSYEEALVHL.[K]_	Q13410						50980	54688		126633	280042										
[E].DITSHME.[S]_	P10451						334546	264329		106248	48684										
[E].DITSHMESEELNGAYK.[A]_	P10451		34932	31422		80455	941586	1050587	688791	990535	491471	290921	193040	262032	190218	267717					
[E].DKTERELLESYIDG.[R]_	P00734										116898										
[E].DMLVVDPK.[S]_	P10451											70104	65406	80990	104100	67787					
[E].DMLVVDPKSKE.[E]_	P10451											22734	21518		30070	15111					
[E].DMLVVDPKSKEEDKHL.[K]_	P10451						50989	51363	90723	47106	30466	64366	56249	50809	50573	44074					
[E].DQQQGEDEHQDKIYPSF.[Q]_	P05814											120640	127533	95860	82267	35029	21077	20490	20128		15571
[E].DQQQGEDEHQDKIYPSFQPQPLI.[Y]_	P05814											43673	49794	58199	42104	23209					
[E].DQQQGEDEHQDKIYPSFQPQPLIYP.[F]_	P05814						80149	126996				152728	109204	170581	136737	45993					
[E].DVSNKVSMSSTVQGSN.[I]_	P31431						41977	46098		19029	17896										
[E].EDFYKLVSEFTITK.[G]_	P19835							39337		103138	163353										
[E].EDFYKLVSEFTITKGLR.[G]_	P19835					51206	113569	128156		130461	294841										
[E].EDFYKLVSEFTITKGLRGA.[K]_	P19835						85419	116978			73656										
[E].EDTNVYTEKHSDSLFK.[R]_	P34741						75795	70161		62628	44814										
[E].EELEKEAKKVEGFDL.[V]_	Q99541											95287	84559	73208	66281	30783					
[E].EELEKEAKKVEGFDLV.[Q]_	Q99541											3736643	3114312	3695931	2601102	1395178	223259	210491	167378	80855	72833
[E].EELQFSLGSKIN.[V]_	Q99541											71711	73700	80371	190120	215824					
[E].EELQFSLGSKINVK.[V]_	P0C0L4											61092	83005	91529	113180	74224					
[E].EELQFSLGSKINVKVGGNS.[K]_	P0C0L4											53477	66082	54593	56277	32285					
[E].EELQFSLGSKINVKVGGNSKGTL.[K]_	P0C0L4											95745	84591	75158	63288	62481					
[E].EELQFSLGSKINVKVGGNSKGTLK.[V]_	P0C0L4											45912	61744	32610	33254	33316					
[E].EKAVADTR.[D]_	P01833									27936	35443										
[E].EKAVADTRDQADG.[S]_	P01833																32118	32903	20953	17616	16880
[E].EKAVADTRDQADGSR.[A]_	P01833						155669	161797	311456	155976	136761						26677	55878	47591		23317
[E].ELEKEAKKVEGFDLV.[Q]_	Q99541											563566	510837	628933	364322	250418					
[E].ELQFSLGSKIN.[V]_	P0C0L4														62334	86136					
[E].ELQFSLGSKINVK.[V]_	P0C0L4											38254	35807	33573	49515	38863					
[E].ELQFSLGSKINVKVGGNS.[K]_	P0C0L4											20866		34870							
[E].ELQFSLGSKINVKVGGNSKGTL.[K]_	P0C0L4											42693	31075	35934	43660						
[E].ELQFSLGSKINVKVGGNSKGTLK.[V]_	P0C0L4											55752	69588	46615	43770	46222					
[E].EQGGSSRALVSTLV.[P]_	P01833			94722	127845	48483															
[E].EQGGSSRALVSTLVPLG.[L]_	P01833						506064	487185	378790	369885	211646	518468	683819	654587	636503	418819	269082	212014	220111	205673	129847
[E].ESITEYKQKVE.[K]_	P05814											28462	26313		31289	24458					
[E].EYMNGMNR.[Q]_	P47710						126497	93732		134309	25261	38770	59414	34733	68889	33829					
[E].GFDLVQKPSYYVRLG.[S]_	Q99541											44322	66887	57568	32186	18695					
[E].GGFVEGVNK.[K]_	P19835					35763	28078	39042		117670	81889										
[E].GGFVEGVNKKLGLLGDSVDIFK.[G]_	P19835						157773	189652	233911	392523	182771										
[E].GPPLDAYSIQGQH.[T]_	Q15365											93445	140157	28490	107534	31566					
[E].HQDKIYPSF.[Q]_	P05814						27041			54160	35357										
[E].HQLLHDKGKSIQDLR.[R]_	P12272									24622	23477										
[E].IMEVPKAKDTVYT.[K]_	P05814					70387		39209		66982	240765										
[E].IMEVPKAKDTVYTK.[G]_	P05814					45406															
[E].IPLSPMGEDSAPRDA.[D]_	Q13410											63879	117747	82561	62547						
[E].IPLSPMGEDSAPRDADT.[L]_	Q13410											30647	31547	33112	29406						
[E].IPLSPMGEDSAPRDADTLHSKLIPTQ.[P]_	Q13410											36561	37923		31890	23907					
[E].IPLSPMGEDSAPRDADTLHSKLIPTQPSQGAP.[-]_1xOxidation [M6]	Q13410											52993	41824		62902	23558					
[E].ISIPASSLPRLTPWIVA.[V]_	Q13410																125187	90043	96479	111047	
[E].KLPGQGVHSQGQGPGAN.[F]_	Q92896																15343	21748	23843		13134
[E].KQTDEIKDTR.[N]_	P47710									20242											
[E].KVKHEDQQQGEDEHQDK.[I]_	P05814							21556		43802	6081										

Quantified modified peptide sequence	Protein ID	A_-80oC	A_-20oC_120h	A_4oC_6h	A_RT_6h	A_RT_24h	B_-80oC	B_-20oC_120h	B_4oC_6h	B_RT_6h	B_RT_24h	C_-80oC	C_-20oC_120h	C_4oC_6h	C_RT_6h	C_RT_24h	D_-80oC	D_-20oC_120h	D_4oC_6h	D_RT_6h	D_RT_24h
[E].KVLADTKELVSSKVS.[G]_	O60664											25504									
[E].KVLADTKELVSSKVSGAQ.[E]_	O60664											41913	43085	42148	27990	25836					
[E].KVLADTKELVSSKVSGAQEMVS.[S]_	O60664											46295	50208	54301	40263	28281					
[E].LLLNPTHQIYPVT.[Q]_	P05814											22856	25038	39316	195079	421502				101550	366424
[E].LLLNPTHQIYPVTQ.[P]_	P05814											50135	70263	50870	125201	206955	12369	10122		45233	107956
[E].LLLNPTHQIYPVTQPLAPVH.[N]_	P05814						53063	56404		44414	45328				330781	1039904					490877
[E].LLLNPTHQIYPVTQPLAPVHNPI.[S]_	P05814					36084															
[E].LQFSLGSKINVK.[V]_	P0C0L4											18803	19040		20375	17978					
[E].LQFSLGSKINVKVGGNSKGTLK.[V]_	P0C0L4											58279	69701	38699	52475	41715					
[E].MAENGVKTITS.[V]_	Q99541											23026			12728						
[E].NALTKSELLVEQ.[Y]_	Q99541											58894	55788	46028	49760	23802					
[E].NALTKSELLVEQY.[L]_	Q99541											69647	78224	50065	40322	36606					
[E].NDERPFYQ.[K]_	P07498						107899	78092		62438	36477	66167	71176	42747	73579	51542					
[E].NDERPFYQK.[T]_	P07498						74408	63430	122089	80920	57487										
[E].NGVKTITSVAM.[T]_	Q99541											62846	74613	106973	41642						
[E].NGVKTITSVAMTS.[A]_	Q99541											179796	172397	105717	126990	63797					
[E].NLHLPLPLL.[Q]_	P05814													48133	144882	328926					36508
[E].NLHLPLPLLQ.[P]_	P05814														63241	134460					
[E].NLHLPLPLLQPLM.[Q]_	P05814														149627	254559					170847
[E].NLHLPLPLLQPLMQ.[Q]_	P05814						50510				84242	58810		70928	124961	392125					
[E].NLHLPLPLLQPLMQQVPQPIPQT.[L]_	P05814						256777	210684		159079	93129	196319	162868	202489	188357	225596					
[E].NLHLPLPLLQPLMQQVPQPIPQTL.[A]_	P05814											69063	52017	68004	86834	207049					
[E].NLHLPLPLLQPLMQQVPQPIPQTLALPPQP.[L]_	P05814											39927	43948	57890	24331	15489					
[E].NLPGVDPNNEAIRN.[A]_	P55036						186581	165811	110577		13292	90749	61315	62015	87460	27948					
[E].NPQPHPGWQG.[T]_	P19835				94571	22508	429272	271443	87166	220984	143985										
[E].NPQPHPGWQGTL.[K]_	P19835						437141	465535	64379	244130	392409										
[E].NYEKNNVMLQW.[-]_	P47710											45913	53053	59703		145133	10270			23188	57751
[E].PIPLESREEYMNGMNR.[Q]_1xOxidation [M]	P47710						611569	591421	1182842	687285	289087	287299	336407	288588	348625	299198					
[E].PIPLESREEYMNGMNRQ.[R]_	P47710						137464	134235	120596	172071	163937	21486			589598	167576					
[E].PIPYGFLPQNI.[L]_	P05814						73951	41646	323207	47217	53317										
[E].PIPYGFLPQNILPLA.[Q]_	P05814						58701	36713													
[E].PIPYGFLPQNILPLAQ.[P]_	P05814						168826	121836	198304		98830										
[E].PIPYGFLPQNILPLAQPAVVLPVPQPEIMEVPK.[A]_	P05814						77790	94826	453008	151159	524429										
[E].PPQSPWDRVKDLATVYVDVLK.[D]_	P02647											43192	60829	58450	55465	25610					
[E].QALQEMGLHLSQSKLKMEDIKE.[V]_1xOxidation [M6]	Q96T51																	29981			18676
[E].QEAEQMPEYR.[G]_	Q13410																45935	48699	28132		
[E].QMPEYRGR.[A]_	Q13410						342557	424192	363563	152796	21465										
[E].RGEKLDDLVSKSEVLGTQ.[S]_	O15498											49082		43177		21728					
[E].RLQNPSESSEPIP.[L]_	P47710						514604	563188	415384	264155	278636	555348	618153	497605	400042	275638	266099	482682	454242	164877	72176
[E].RLQNPSESSEPIPL.[E]_	P47710						44764	51116	49413	36080	34564										
[E].RLQNPSESSEPIPLES.[R]_	P47710						364409	409729	234862	143867	162725	231397	239730	215889	137411	57648	37750	53771	41112	36535	14785
[E].RLQNPSESSEPIPLESREE.[Y]_	P47710						135652	117400	75591	38025	85064	101719	135516	81881	103950	101340		19790			
[E].RLQNPSESSEPIPLESREEY.[M]_	P47710						92626	161330	83523	68184	55053	47464	75598	60760	49418	57761	29342	43319	43803		36434
[E].RLQNPSESSEPIPLESREEYMN.[G]_	P47710						53679	96338		44167		88869	75740	69025	45181	52242	158840	172023	209237	82437	104558
[E].RLQNPSESSEPIPLESREEYMN.[G]_1xOxidation [M21]	P47710																			50799	20449
[E].RLQNPSESSEPIPLESREEYMNG.[M]_	P47710						108510	112918	172220	105103	155158	353493	328908	308213	277185	199327	51037	35188	51240		42081
[E].RLQNPSESSEPIPLESREEYMNGM.[N]_	P47710						228135	227088	179704	193390	146414	330028	266356	272024	305587	268121		41987	23335		26667
[E].RLQNPSESSEPIPLESREEYMNGMNR.[Q]_1xOxidation [M]	P47710						1421401	1310947	2312105	889598	319292	225913	304429	231546	268525	721202		28036	17731	167074	144718
[E].RLQNPSESSEPIPLESREEYMNGMNRQ.[R]_	P47710						333587	301071	290113	204894	192806	142404	167113	161181	136538	159233		30736	11980		38778
[E].SEELNGAYK.[A]_	P10451						113613	72229		107560		110359	110076	95043	132691	74059					
[E].SITEYKQKV.[E]_	P05814		46529	32508	147664	63052	212194	228084	163529	258776	133656	390569	422895	369617	719801	587354					
[E].SLSSSEESITEYKQ.[K]_	P05814						68851	73861		64913	75377	37378	43062		49451	67507					
[E].SLSSSEESITEYKQKV.[E]_	P05814											152062	248817	287465	443879	386404		17162			46089
[E].SPPPYRPDEFKPNH.[Y]_	Q16625											47512	55073	42822	40466	32019					
[E].SQNAQDQGAEMDKSSQETQR.[S]_	Q99541											57530	73641	78255	63576	25755					
[E].SREEYMNGM.[N]_	P47710						150920	111965	129991	232283	47520	142139	145048	83710	187009	90160	44761	136326	45221		
[E].SREEYMNGMN.[R]_1xOxidation [M]	P47710						143531	124330	294121	82933	35924	23739	41739	23880	12463	44879					
[E].SREEYMNGMNRQ.[R]_	P47710						52410	40787	97696	37375	9213	28655	45303		33434						
[E].SREEYMNGMNRQR.[N]_	P47710						133477	91278	191939	117083	16761	38764	40511	29950	54475	49702		38749			
[E].SREEYMNGMNRQRNIL.[R]_	P47710						266685	227267	466329	341251	187088	39609	37028	40701	43201	74503					
[E].SSEPIPLESRE.[E]_	P47710						199942	176271	100423	212680	85789	53882	47306		66983	60646					
[E].SSEPIPLESREE.[Y]_	P47710						139700	100219	66789	180703	87686										
[E].SSEPIPLESREEY.[M]_	P47710						218202	143246	93579	416773	152742				27988	13751					
[E].SSEPIPLESREEYM.[N]_	P47710						254585	154076	193110	297707	246600										
[E].SSEPIPLESREEYMNGM.[N]_	P47710						176504	98101	206827	348491	101458										
[E].SSEPIPLESREEYMNGMN.[R]_	P47710						2984446	2381422	2326736	3680806	4575260	298252	279307	322172	380320	329312	15453				33921
[E].SSEPIPLESREEYMNGMN.[R]_1xOxidation [M]	P47710						300093	256618	450087	234133	233619										
[E].SSEPIPLESREEYMNGMN.[R]_2xOxidation [M14; M17]	P47710						35424	50126			31630										
[E].SSEPIPLESREEYMNGMNR.[Q]_	P47710						3394901	2649595	3759475	5042216	2512967	498180	468864	381718	643027	780275		23799			21409
[E].SSEPIPLESREEYMNGMNR.[Q]_1xOxidation [M]	P47710						216432	266222	605785	384971	75151										
[E].SSEPIPLESREEYMNGMNRQ.[R]_	P47710						194208	203802	356584	175604	167541										
[E].SSEPIPLESREEYMNGMNRQR.[N]_	P47710						327249	198823	178344	235717	35073										
[E].SSEPIPLESREEYMNGMNRQRNIL.[R]_	P47710						212469	105607	320366	118056	124531										
[E].SWAQDPSQENK.[K]_	P19835						55656	57911		87189	41889										

Quantified modified peptide sequence	Protein ID	A_-80oC	A_-20oC_120h	A_4oC_6h	A_RT_6h	A_RT_24h	B_-80oC	B_-20oC_120h	B_4oC_6h	B_RT_6h	B_RT_24h	C_-80oC	C_-20oC_120h	C_4oC_6h	C_RT_6h	C_RT_24h	D_-80oC	D_-20oC_120h	D_4oC_6h	D_RT_6h	D_RT_24h
[E].THSHKQSRLY.[K]_	P10451						37788			26928	10811										
[E].TIESLSSSEESITEY.[K]_	P05814											34792	36438	33925	29472	23992					
[E].TIESLSSSEESITEYKQ.[K]_	P05814					32443	52062	63788		80864	150857	49726	32825	66198	62467	70852					
[E].TIESLSSSEESITEYKQK.[V]_	P05814						280785	242021	127533	336832	341881	194924	138345	211051	307886	427554					
[E].TIESLSSSEESITEYKQKVE.[K]_	P05814						75672	98923		54772	76362	173343	140664	155756	176054	148696					
[E].TIESLSSSEESITEYKQKVEK.[V]_	P05814		45486	47464		43518	657449	625949	718905	491594	344602	805449	800913	735682	677475	1884445					
[E].TPTSRQLSEYL.[K]_	P22079										126132										
[E].TSQLDDQSAETHSHKQSRLY.[K]_	P10451											152078	159180	121478	120983	168998					
[E].VPKAKDTVYTK.[G]_	P05814						27631	34663	138672	46543					25137	24423					
[E].YMNGMNRQRNILR.[E]_	P47710						134683	90526	123862	231735	125066										
[E].YPTYHTHGRYVPP.[S]_	P15941											100769	149868	163584	118671	101392					
[E].YPTYHTHGRYVPPS.[S]_	P15941											186808	278790	262227	184534	247901					
[F].AAPTKALENPQPHPGWQGTL.[K]_	P19835						186898	170705	149970	166701	270608										
[F].AEEKAVADTRDQ.[A]_	P01833											102565	110749	96643	86149	56004					
[F].AEEKAVADTRDQADGSRASVDSGSSEEQGGSS.[R]_	P01833											48511	47821	56892	48707	40167					
[F].APVNVTTEVKSVE.[M]_	P68104							33019	113255			66397	69495	70158	75068	33775					
[F].APVNVTTEVKSVEMH.[H]_	P68104											51275	53655	48390	41141						
[F].DLVQKPSYYVRLG.[S]_	Q99541											319950	374855	486527	236735	140944	35242	35421	38655	35138	16376
[F].DPQIPK.[L]_	P05814						2310727	2086378	1403987	2143892	1097687	20283	24193	33977	42875	45893					
[F].DPQIPKLTD.[L]_	P05814						224575	187579	46241	94667	93615	223928	183276	188371	264706	341526					
[F].DPQIPKLTDL.[E]_	P05814						42859	45085		26457	38898					49602					
[F].DPQIPKLTDLE.[N]_	P05814						84220	92658	45449	54867	52233	212593	217912	223177	213090	435937					
[F].DPQIPKLTDLEN.[L]_	P05814						96207	99332	43420	42848	46324	64764	65135	65790	57196						
[F].DPQIPKLTDLENLHL.[P]_	P05814			73921								53564	52214		70454	92740					
[F].DPQIPKLTDLENLHLPL.[P]_	P05814						185792	454544	207065	448888	205233	689642	503015	647358	685122	549999					
[F].DPQIPKLTDLENLHLPLPLL.[Q]_	P05814						106828	59364				472673	445257	438122	340187	266293					
[F].DPQIPKLTDLENLHLPLPLLQPL.[M]_	P05814		188812	45644		97248	1276316	1265339	855803	942848	542809	4349546	3604614	4197861	3282462	2314617		43418			64115
[F].DPQIPKLTDLENLHLPLPLLQPLM.[Q]_	P05814						3269689	2687658	2323959	2045666	1295492	13366615	12232302	13266343	10124792	8040587	64993	58267	66440		64827
[F].DPQIPKLTDLENLHLPLPLLQPLM.[Q]_1xOxidation [M24]	P05814						256797	291369	95153	183463		1400394	1249322	1554836	1493045	1852228					
[F].DPQIPKLTDLENLHLPLPLLQPLMQ.[Q]_1xOxidation [M24]	P05814											323119	481191	385614	251920	321336					
[F].DPQIPKLTDLENLHLPLPLLQPLMQQV.[P]_	P05814											108125	102650	100795	71284	44972					
[F].DPQIPKLTDLENLHLPLPLLQPLMQQVPQPIPQT.[L]_	P05814											7640831	6949767	7700309	6000041	3922892					
[F].DPQIPKLTDLENLHLPLPLLQPLMQQVPQPIPQTL.[A]_	P05814											794024	684885	771452	630370	411217					
[F].DQGADAIYDHINEGKLW.[K]_	Q9UDW1					107200	95660	55339		145851	277861										
[F].DSDPITVTVPVEVSRK.[N]_	P10909											232149	241160	222631	170845	73125					
[F].DSDPITVTVPVEVSRKNP.[K]_	P10909											219286	197715	197953	149154	79854					
[F].DVYTESWAQDPSQE.[N]_	P19835						75882	78178	93240	50359	43562										
[F].DVYTESWAQDPSQEN.[K]_	P19835					27652	33501	52415		32103	84131										
[F].DVYTESWAQDPSQENKKK.[T]_	P19835						162786	74928	487584	45479	31792										
[F].ERAFDQGADAIYDHINEGKLW.[K]_	Q9UDW1							54302		59902	96473										
[F].ESRAQLGGPEAAKSDETAAK.[-]_	P04792											27970	18755	22898	19944						
[F].ETDVLFLVPTEIALAQH.[R]_	P19835							21589	118226	64332	47624										
[F].ETDVLFLVPTEIALAQHRANA.[K]_	P19835						58961														
[F].FDPQIPKLT.[D]_	P05814											23195	29444	23766		21608					
[F].FDPQIPKLTD.[L]_	P05814											47024	35106	35053	62181	236518					
[F].FDPQIPKLTDLE.[N]_	P05814															88926					
[F].FDPQIPKLTDLENLHLP.[L]_	P05814											219897	204247	206080	73619	70169					
[F].FDPQIPKLTDLENLHLPLP.[L]_	P05814					86585	1289668	1248777	527527	792509	1111395	1584429	1278268	1537696	2147113	2693105		34878			126374
[F].FDPQIPKLTDLENLHLPLPL.[L]_	P05814					86284	156712	139992		174686	139295	248458	248015	241641	653466	974608	37660	107617	73568	60705	756607
[F].FDPQIPKLTDLENLHLPLPLL.[Q]_	P05814											31321	20768	16716	16952	72391					
[F].FDPQIPKLTDLENLHLPLPLLQ.[P]_	P05814											100526	62499	75800		165168					
[F].FDPQIPKLTDLENLHLPLPLLQP.[L]_	P05814					162432	551038	549436		383177	672144	544121	454382	555525	700040	986173					55873
[F].FDPQIPKLTDLENLHLPLPLLQPLM.[Q]_	P05814					41911	773947	690578	377992	433232	483304	2325463	1942862	2461406	2677228	3198168	128749	58415			149683
[F].FDPQIPKLTDLENLHLPLPLLQPLM.[Q]_1xOxidation [M25]	P05814						98360	122831		42267		145342	165226	162148	194478	309629					
[F].FDPQIPKLTDLENLHLPLPLLQPLMQ.[Q]_	P05814		18725	58365			474133	416882	211103	242837	176606	1870519	1597410	1949702	1904964	2285250		77701	32899		126538
[F].FDPQIPKLTDLENLHLPLPLLQPLMQ.[Q]_1xOxidation [M25]	P05814											44436	96889	57833	86946	173722					
[F].FDPQIPKLTDLENLHLPLPLLQPLMQQ.[V]_	P05814															581656					
[F].FDPQIPKLTDLENLHLPLPLLQPLMQQV.[P]_	P05814											57304	75686	50301	64784	43739					
[F].FDPQIPKLTDLENLHLPLPLLQPLMQQVPQPIP.[Q]_	P05814						43633	44531	384366	56951	146838	173837	175758	199028	193433	219385					
[F].FDPQIPKLTDLENLHLPLPLLQPLMQQVPQPIPQT.[L]_	P05814						882355	1106593	306954	523078	482898	2164308	1953882	2227490	1899780	1652650					
[F].FSYQAWRGVGSNAATSQMAGGYA.[-]_	Q9Y342											36389	35400	45665	43980						
[F].HGMNPSKDTSTEYSEVRTQ.[-]_	P20138											74854	89656	66991	49452	38285	38665	44093	32684		11177
[F].KSHALQLNNRQI.[R]_	P0C0L4											19440	40252	34002	25360	30383					
[F].LPQNILPLAQPAVVLPVPQPEIMEVPK.[A]_	P05814											289174	277732	308947	193908	143456					
[F].LPQNILPLAQPAVVLPVPQPEIMEVPKAKDTVYT.[K]_	P05814									406676					439892	379723					
[F].LSALEEYTKKLNTQ.[-]_	P02647							24807		32718											
[F].LVPTEIALAQHRANA.[K]_	P19835							20954			83282										
[F].LYEDSKSVRLE.[D]_	Q13410											89645	85554			43364					
[F].QLFGSPSGQKDLLFK.[D]_	P02788						77365			52305											
[F].QPQPLIYPFVEPIPYGF.[L]_	P05814											113137	127625	110822	134894	74205					
[F].QPQPLIYPFVEPIPYGFLP.[Q]_	P05814											708322	749901	926443	655882	303372	31601	53878	40451		27577
[F].QPQPLIYPFVEPIPYGFLPQ.[N]_	P05814											482664	443542	595098	415798	199414		17261	17797	25399	
[F].QPQPLIYPFVEPIPYGFLPQN.[I]_	P05814											67061	61435	63315	71459	28446					
[F].RISHELDSASSEVN.[-]_	P10451					26285	144452	133112	148015	155007		203735	203561	195983	277323	224733					

Quantified modified peptide sequence	Protein ID	A_-80oC	A_-20oC_120h	A_4oC_6h	A_RT_6h	A_RT_24h	B_-80oC	B_-20oC_120h	B_4oC_6h	B_RT_6h	B_RT_24h	C_-80oC	C_-20oC_120h	C_4oC_6h	C_RT_6h	C_RT_24h	D_-80oC	D_-20oC_120h	D_4oC_6h	D_RT_6h	D_RT_24h
[F].RRPDIQYPDATD.[E]_	P10451						478438	368497	216975	612816	339445	218307	149426	116744	202373	179927					
[F].RRPDIQYPDATDEDIT.[S]_	P10451						60942	85644				221583	224494	143137	86415	50171					
[F].RRPDIQYPDATDEDITSH.[M]_	P10451						214542	213808	114965	88277	32955	660986	680173	530652	237311	142395	16720	57744	32265	21961	13444
[F].RRPDIQYPDATDEDITSHMESEELNGA.[Y]_	P10451											27054	101729	16654		28883					
[F].SDISNPTAHENYEKNNVMLQW.[-]_	P47710														59462		21361	36779	37231		99551
[F].SDISNPTAHENYEKNNVMLQW.[-]_1xOxidation [M18]	P47710																				28745
[F].SEYASVQVPRK.[-]_	P78324											70003	76150	51673	56311	33402					
[F].SGMNSVSSIQNQMQSKGGYGGGMPANVQMQLVDTKAG.[-]_	Q9NRR3											97826	127024	118442	61939	55190		28699	15992		
[F].SLGSKINVKVGGNSKGT.[L]_	P0C0L4											27227	34826	28305	30058	24758					
[F].SLGSKINVKVGGNSKGTL.[K]_	P0C0L4											67594	91183	63345	51648	47326					
[F].SLGSKINVKVGGNSKGTLK.[V]_	P0C0L4											44608	73025	50997	46623	50626					
[F].VEGVNKKLG.[L]_	P19835						34927	31792		38153	46471										
[F].VEPIPYGFLPQNILPLAQPAVVLPVPQPEI.[M]_	P05814											69050	38093	53250	44422	22211					
[G].AGSTTVKIVLK.[E]_	Q6WN34					40846	65850	64920		140139	137852										
[G].AKTTFDVYTESWAQDPSQEN.[K]_	P19835									22180	99055										
[G].AKTTFDVYTESWAQDPSQENK.[K]_	P19835					80358	73853	69056		293190	346415										
[G].DIGKWLPNGTLK.[I]_	P33121															42550					
[G].DIVDIKGMGTVQK.[G]_	P46778						23759	18490			31112	25255	39149		45232	35524					
[G].DLPYDGRLLEEAVRK.[K]_	Q86X29								62455	123626	196030										
[G].DPNMGDSAVPTHW.[E]_	P19835				57523	44135	45252	32294		33477	96712										
[G].DQTVSDNELQEMSNQGSK.[Y]_	P10909						80448	116431	96514	88734	79628	78026	76566	71209	66453	28203					
[G].DQTVSDNELQEMSNQGSKYVN.[K]_	P10909											59623	42298	58363	47177	36623					
[G].DQTVSDNELQEMSNQGSKYVNK.[E]_	P10909						96322	106875	165182	75956	107814	181108	175401	169358	106399	73722					
[G].DQTVSDNELQEMSNQGSKYVNKEIQNAVN.[G]_	P10909											30819		17646							
[G].DQTVSDNELQEMSNQGSKYVNKEIQNAVNGVK.[Q]_	P10909						68092	48013	114653		42062		66634								
[G].DSAVPTHWEPYTTENSGYLEITKKMGSSSM.[K]_	P19835					111766	168025	153283		124797	53553										
[G].DSVDIFKG.[I]_	P19835						52061	58669		71750	131596										
[G].EDEHQDKIYP.[S]_	P05814						362583	367436	322462	416197	344008										
[G].EDEHQDKIYPSFQPQPLIYPFVEPIPY.[G]_	P05814						158031	152929	182951	179043	123214										
[G].EDSAPRDADTLHSKLIPTQ.[P]_	Q13410											47498	51832	52020	41774						
[G].EIDYEAIVKL.[S]_	P62333						40070	27665													
[G].EVQAMLGQSTEELRVRLASHL.[R]_	P02649						39931	42440													
[G].FDLVQKPSYYVRLG.[S]_	Q99541											114138	124243	59988	59155	42204	29211		29099		
[G].FKSHALQLNNRQI.[R]_	P0C0L4											18559		28017	26524	22291					
[G].FPATASNDLK.[A]_	Q9UNQ0											53800	68476	69581	46917	26021					
[G].FVEGVNK.[K]_	P19835						14753	18911		59673	43296										
[G].FVEGVNKK.[L]_	P19835						17149	28092		43186	13018										
[G].GFVEGVNKK.[L]_	P19835						43884	51802		98679	35479										
[G].GQQQQGPPPPQGKPQ.[G]_	P02810																	29359			
[G].GQYGNPLNKYI.[R]_	O14672																17669	22010	21126		
[G].GVQSVMGSRLG.[Q]_	O60664											23974	31384								
[G].HLQSLQRLIDSQMETSCQITFE.[F]_1xOxidation [M13]	P09603						60235	64883	129222	86580	64131										
[G].IGLVKGGVSAVAGGVTAVG.[S]_	Q8WUH6											87727	95873	44311	61957	28652					
[G].IGLVKGGVSAVAGGVTAVGSAVVN.[K]_	Q8WUH6											119756	161275	148174	91478	48999					
[G].IPFAAPTKALE.[N]_	P19835						254398	173946		75227	106993										
[G].IPFAAPTKALENPQPHP.[G]_	P19835						70498	87313		49620	31246										
[G].IPFAAPTKALENPQPHPG.[W]_	P19835						155691	114676		62469	40094										
[G].IPFAAPTKALENPQPHPGWQG.[T]_	P19835						241272	234489	203641	137843	83120										
[G].IPFAAPTKALENPQPHPGWQGTLK.[A]_	P19835						144726	161221	160089	90911											
[G].IPPSGPAGELKFEPHI.[-]_	P80303						29819	36315		109233	346814										
[G].KPFATPTGYRPQD.[R]_	P19835						123768	155034	236485	193225	303229										
[G].KPGDQILDWQYGVTQ.[A]_	Q6UX71							25339	119762	25796	20056	148702	51992	46136	99899	80482	222222	238758			
[G].KPGDQILDWQYGVTQAFPHTEE.[E]_	Q6UX71							105348		58886	37336										
[G].KSGKQGEKGRTGAKGAKGYQ.[G]_	Q8IZC6																174005	114158	150824	460494	175153
[G].LLGDSVDIFK.[G]_	P19835						36303	40108		53410											
[G].LPFYNGFYYSNSANDQNLGNGHG.[K]_	Q96S86					47079	68561	72470		106037	178549										
[G].NEIDAQNPQIKRITDKADTN.[R]_	O00161											32402	27317	25000							
[G].NPLNKYI.[R]_	O14672						54936	59032		46231	18079										
[G].NTNGFPATASNDLK.[A]_	Q9UNQ0							154209		24080	16348										
[G].RATLVQDGIAK.[G]_	Q13410																68104	98460	46380	29635	
[G].REQEAEQMPEY.[R]_	Q13410																40349	44240	29230	23864	
[G].REQEAEQMPEYR.[G]_	Q13410																43043	216614	72499		
[G].REQEAEQMPEYRGR.[A]_	Q13410																	59359	23116		
[G].RVMPVLKSPT.[I]_	P05814						252033	187916		202933	192431	67422	102362	48024	91449	168673					
[G].RVMPVLKSPTIP.[F]_	P05814						129663	117765	100405	158306	306639	274571	335644	223814	360683	747698					
[G].SINTVLGSRMM.[Q]_	Q99541											140891	204223	31860	120755	61393	33000	16519	16162	35328	
[G].SLSTKLHSRAYQ.[Q]_	Q99541											163151	195098	72082	76147	27810					
[G].SLSTKLHSRAYQQA.[L]_	Q99541											393625	563095	284930	275205	148289					
[G].SRASVDSGSSEEQGGSSRALVST.[L]_	P01833											76820	58836	15448	72548	49671	27588	45369	34590	41408	27706
[G].SRASVDSGSSEEQGGSSRALVSTLVPLG.[L]_	P01833											1004581	1146799	1046832	887482	481367	592056	723839	536307	413025	351984
[G].SRMMQLVSSGVENALT.[K]_	Q99541											44948									
[G].SRMMQLVSSGVENALTK.[S]_	Q99541											141008	119215	60519	107852	49673					
[G].SRMMQLVSSGVENALTKSELL.[V]_	Q99541											65264	50524		63437						
[G].SSEEQGGSSRALVSTLVPLG.[L]_	P01833						84960	100066	66886	59422		275362	318528	415841	213129	76104	67963	78302	62943		37039

Quantified modified peptide sequence	Protein ID	A_-80oC	A_-20oC_120h	A_4oC_6h	A_RT_6h	A_RT_24h	B_-80oC	B_-20oC_120h	B_4oC_6h	B_RT_6h	B_RT_24h	C_-80oC	C_-20oC_120h	C_4oC_6h	C_RT_6h	C_RT_24h	D_-80oC	D_-20oC_120h	D_4oC_6h	D_RT_6h	D_RT_24h
[G].SSIQGLPTNVKDQVQ.[Q]_	O60664											124549	129285	58498	102426	41681	31150	27280			
[G].SSIQGLPTNVKDQVQQ.[A]_	O60664						48647	57301		40959	32846	110745	109214	176204	84350	48302					
[G].SSIQGLPTNVKDQVQQARRQVEDLQ.[A]_	O60664		61697	55644								187615	208129	107390	153655	86958	17851	41561	23333		12363
[G].SVEKTKSVVSG.[S]_	Q99541											27069	34285		31621						
[G].SVEKTKSVVSGSINTVLG.[S]_	Q99541											44571	52948	58629	32540						
[G].TPAPTGLSAPLSFIPR.[H]_	Q6WN34						74921	76047		68550	39071										
[G].VDKTKSVVTGGVQ.[S]_	O60664											24744		25571							
[G].VENALTKSELL.[V]_	Q99541											82446	81457	106697	45803	18015					
[G].VENALTKSELLVEQ.[Y]_	Q99541												33152	44412							
[G].VENALTKSELLVEQY.[L]_	Q99541											27011	38935	57974							
[G].VNDNEEGFFSAR.[G]_	P02675																35948				
[G].VPANRIVV.[R]_	P47989						197603	240781	181567	117634	27472										
[G].VPQNIQDQAKHMG.[V]_	Q99541											32618	49191	63295	29518	12488					
[G].VPQNIQDQAKHMGVM.[A]_	Q99541											119548	131781	84990	101165	50628					
[G].VPQNIQDQAKHMGVMA.[G]_	Q99541											276316	286016	350959	258761	152476					
[G].YVDDTQFVRFDNDAASPR.[M]_	P13747																	21026	20611		
[H].ALQLNNRQIR.[G]_	P0C0L4											227450	326828	197959	281659	88695					
[H].AVDVTLDPDTAHPHLF.[L]_	Q13410											320501	336648	132778	252790	131718					
[H].AVDVTLDPDTAHPHLFLY.[E]_	Q13410											124227	103597	52802	85205	70263					
[H].DKGKSIQDL.[R]_	P12272						97227	96608	105268	50960	31552	41874	50942	46651	22685	25980					
[H].DKGKSIQDLR.[R]_	P12272											54966	93263	79480	97080	58351					
[H].EDQQQGEDEHQDK.[I]_	P05814						20622		90149												
[H].EDQQQGEDEHQDKIYPS.[F]_	P05814											171627	191793	193719	215669	58575	42167	37264	42464	37975	31589
[H].EDQQQGEDEHQDKIYPSFQPQPLIYP.[F]_	P05814						211387	236785	199156	147511	36645	181475	179199	181790	148602	104818					
[H].ELQEKLSPLGEEMRDR.[A]_	P02647							30866		44784	44742	28532	33828	27992	39537	72194					
[H].ENDERPFYQK.[T]_	P07498						157490	172432	264618	253366	181423	132280	147447	120271	112211	81207					
[H].ETYGHKTPY.[-]_	Q9NRJ3						25223	31113		37932											
[H].FRPKGAGSTTVKIVL.[K]_	Q6WN34					50511	44951	41296	55781	88001	125106										
[H].GEIDYEAIVKL.[S]_	P62333						58603	51205		34174		39465	27805	14769	35444	18733					
[H].GRYVPPSSTDRSPYEKVSAGNGGSSLS.[Y]_	P15941											18172		26891	24377	12202					
[H].HLIAEIHTAEIR.[A]_	P12272						16249			34771	35562										
[H].LPLPLLQPLMQQVPQPIPQTL.[A]_	P05814											158690	124295	137407	165164	250198	57543	15984	20942	39790	34180
[H].LPLPLLQPLMQQVPQPIPQTLALPPQPLWSVPQP.[K]_	P05814											234492	273304	262173	210946	187966					
[H].MESEELNGAYK.[A]_	P10451						99917	142611		44339	24184	284435	252166	225260	151947	60590	19737	53352	26429		
[H].QDKIYPSFQPQPL.[I]_	P05814																	14921	15366		16004
[H].QIYPVTQPLAPVH.[N]_	P05814						68257	46171				51524	52102	52438	80391	105581					79254
[H].RDGREQEAEQMPEYR.[G]_	Q13410																68465	122895	88680		
[H].REFPFYGDYGSNYLYDN.[-]_	P15515											50138	47018	23086	14193						
[H].SHEDMLVVDPK.[S]_	P10451											106607	93765	81313	94386	34858					
[H].SKLIPTQPSQ.[G]_	Q13410											184290	199714	34575	100191	48192	76992	73301	44573	46354	22563
[H].SKVVSEQKDTLGN.[T]_	Q14512																18859	25648	24462		
[H].SKVVSEQKDTLGNTQIK.[Q]_	Q14512																	15613	17203		
[H].SKVVSEQKDTLGNTQIKQ.[K]_	Q14512											75246		92503	90479	65823					
[H].SKVVSEQKDTLGNTQIKQK.[S]_	Q14512												62048	26402							
[H].SPAFQHPPTE.[F]_	P10909											35096	23594	30355	28634	25802					
[H].SPAFQHPPTEFI.[R]_	P10909											40547	47656	47828	44471	27810					
[H].STVHLIEFARKN.[V]_	Q99541											33009	45070	14454							
[H].STVHLIEFARKNVY.[S]_	Q99541											24745	30851			39825					
[H].STVHLIEFARKNVYSANQ.[K]_	Q99541											184895	216178	216186	95689	86584					
[H].YPVSAGYTKLVGQD.[V]_	P06858											37947	49578	48288	37164	16069					
[H].YPVSAGYTKLVGQDV.[A]_	P06858											53817	75697	53730	65650	38153					
[I].ASFDKAKLKKTETQEKN.[T]_	P63313											42129	74339		35488						
[I].DDLYSTIKV.[-]_	P33121											131485	141219	165139	192822	129918	143105	169537	155204	174672	137047
[I].DELFQDR.[F]_	P10909						65910	80691	56145	80386	76819	34784	36696	38521	21165	12256					
[I].DELFQDRFF.[T]_	P10909												31617	34494							
[I].DELFQDRFFTREPQD.[T]_	P10909						347859	282943	84137	180045	132252	135043	140629	150531	166780	155623					
[I].DELFQDRFFTREPQDTYHYLPFSLP.[H]_	P10909											173850	96348	297016	262121	206420					
[I].DIDKTKVQNIHPVES.[A]_	P80303											45914	26482	20104							
[I].DSQELSKVSREF.[H]_	P10451											69572	62614	80281	79308	54614					
[I].ENKAIQDPRLFAEEK.[A]_	P01833						244328	265495	259419	113333	24792										
[I].ESLSSSEESITEYKQ.[K]_	P05814					22928	38060	40904		41817	80509	35727	34347		53097	63723					
[I].ESLSSSEESITEYKQKV.[E]_	P05814											94354	83876	113392	99667	69960					
[I].ESQDAGIKTITMLD.[E]_	O00161											57594	60645	100261	48286	25291					
[I].FLSKGKDAMQSFMMPFYLMKDC.[E]_2xOxidation [M9; M13]	Q969T9						63807	86171	105563	75007	73190										
[I].GGIGTVPVGRVETGVLKPGMVVT.[F]_	P68104											304470	328232	278229	210229	122456					
[I].GLVKGGVSAVAGGVTAVG.[S]_	Q8WUH6											57640	58397	77775	67612						
[I].LPLAQPAVVLPVPQPEI.[M]_	P05814											50682	44592	56752	63427	121024					
[I].LPLAQPAVVLPVPQPEIMEVPKA.[K]_	P05814											103368	82856	110015	90471	49403					
[I].LPLAQPAVVLPVPQPEIMEVPKAKDTVYTK.[G]_	P05814								883854	855619											
[I].LREKQTDEIKDT.[R]_	P47710					81759	115986	119709	90960	247520	318299										
[I].MEVPKAKDTVY.[T]_	P05814						50374	38279		63423	15596	25657	22425	19861	53351	48248					
[I].MEVPKAKDTVYTKG.[R]_	P05814			27894		40596	24184			23312	29354							18532	30585		60039
[I].MEVPKAKDTVYTKGR.[V]_	P05814															41508					21085
[I].NVKVGGNSKGTLKVL.[R]_	P0C0L4														21555	34061					

Quantified modified peptide sequence	Protein ID	A_-80oC	A_-20oC_120h	A_4oC_6h	A_RT_6h	A_RT_24h	B_-80oC	B_-20oC_120h	B_4oC_6h	B_RT_6h	B_RT_24h	C_-80oC	C_-20oC_120h	C_4oC_6h	C_RT_6h	C_RT_24h	D_-80oC	D_-20oC_120h	D_4oC_6h	D_RT_6h	D_RT_24h
[I].PASSLPRLT.[P]_	Q13410						63569	70245													
[I].PASSLPRLTPWIV.[A]_	Q13410																	24323			26267
[I].PASSLPRLTPWIVA.[V]_	Q13410							131071													
[I].PFAAPTKALENPQPHPGWQG.[T]_	P19835						48502	55267		52665	60212										
[I].PFFDPQIPKL.[T]_	P05814						62891				58526	191562	150955	200401	338060	850921		10978			37105
[I].PFFDPQIPKLTD.[L]_	P05814															70489					
[I].PFFDPQIPKLTDLENLHL.[P]_	P05814														14471	30717					
[I].PFFDPQIPKLTDLENLHLPL.[P]_	P05814			32089		37394	79458	105758		141512	84569										
[I].PFFDPQIPKLTDLENLHLPLPL.[L]_	P05814											66308			16695	27841					
[I].PFFDPQIPKLTDLENLHLPLPLLQP.[L]_	P05814		33559			70937	139031	162459	100537	149158	212399										
[I].PKLTDLENLHLPLP.[L]_	P05814						152993	202951	193986	107788	144590	314687	270610	297708	330679	227981					
[I].PLESREEYMNGMN.[R]_	P47710		54347			409148	1088311	1221778	2543094	1716515	1775998	151604	137756	147104	117035	110402	22279	11022	18993		14587
[I].PLESREEYMNGMN.[R]_2xOxidation [M9; M12]	P47710						57385	52521	417802	47914	45043										
[I].PLESREEYMNGMNR.[Q]_	P47710		32268	64748		261985	4091607	4736506	8862038	7637081	3657136	2140050	2371432	2174194	2079302	2357252	72572	130936	100399		103979
[I].PLESREEYMNGMNR.[Q]_1xOxidation [M]	P47710					20877	795849	1329738	7681001	1951250	491603										
[I].PLESREEYMNGMNR.[Q]_2xOxidation [M9; M12]	P47710						47362	36613	308101	74528											
[I].PLESREEYMNGMNRQ.[R]_	P47710						326402	443114	1721633	884182	739571			24867		33242					
[I].PLESREEYMNGMNRQR.[N]_	P47710						121728	36328	156790	172869	33344										
[I].PLESREEYMNGMNRQRNIL.[R]_	P47710						60141	65477	111742	75969											
[I].PLSPMGEDSAPRDADTLHSK.[L]_	Q13410											63297	43010	45784	41668	36555					
[I].PLSPMGEDSAPRDADTLHSKLIPTQ.[P]_	Q13410												122881	52040	29697	152331					
[I].PQQVVPYPQRAVPVQ.[A]_	P05814										27469					45282					
[I].PQQVVPYPQRAVPVQA.[L]_	P05814		35580			74011	41490	52664		41332	98649	36768		47578	34910	64916					
[I].PQTLALPPQPLWSVPQPK.[V]_	P05814						44865	38436	249764	83611	163210										
[I].PTAPPSHTGRHL.[W]_	Q9NZH0											32218	61466	58725	40332	26831					
[I].PVAQDLNAPSDWDSR.[G]_	P10451						71378	108092		140394	378833		36602	51125	36115	40979					
[I].PVAQDLNAPSDWDSRGK.[D]_	P10451						67560	90813	70985	122196	236222	33517	33210	32685	29426	85108					
[I].PVAQDLNAPSDWDSRGKD.[S]_	P10451						43079			71203	42396										
[I].PVAQDLNAPSDWDSRGKDS.[Y]_	P10451						687796	632140	236011	189794	134362	2552025	2613029	2822872	2266860	1925428					
[I].PVAQDLNAPSDWDSRGKDSYETS.[Q]_	P47710															48366					
[I].PVKQADSGSSEEKQL.[Y]_	P10451											25372			20509						
[I].PVKQADSGSSEEKQLY.[N]_	P10451											130136	106434	96811	107635	75900					
[I].PYGFLPQNILPLAQPAVVLPVPQPEIMEVPKAKDTVYT.[K]_	P05814					153686	216395	68456	1682049	146386	335589										
[I].QDPRLFAEEKAVAD.[T]_	P01833																50348	54688	55556	31817	19221
[I].QDPRLFAEEKAVADTRDQ.[A]_	P01833											211376	192450	168617	166389	89565	108253	114329	111398	56582	48990
[I].QDPRLFAEEKAVADTRDQADG.[S]_	P01833											108761	91760	89427	97748		34067	36071	27511	22202	
[I].QYPDATDEDITSH.[M]_	P10451											63806	57132	48381	38815	15328					
[I].RGLEEELQFSLGSKINVK.[V]_	P0C0L4					22934															
[I].SIPASSLPRLTPWIVAVA.[V]_	Q13410																61602	48256	48799	50574	31785
[I].SNPTAHENYEKNN.[V]_	P47710						27326	35426													
[I].SPLDLAKLN.[Q]_	Q15365											27159		22552							
[I].SPVEESEDVSNKVS.[M]_	P31431											103626	95038	109168	90762	56824	62731	55475	56733	55883	34641
[I].SVGYVDDTQFVR.[F]_	P13747																24658	16200	13631		
[I].TEYKQKVEK.[V]_	P05814						351141	508579	288419	662975	389514	237708	141281	81196	222484	138016					
[I].YPFVEPIPYGFLPQ.[N]_	P05814						67251	62001		35367											
[I].YPFVEPIPYGFLPQNIL.[P]_	P05814						269071	145666		130530	124160	35437	33813	54075	91886	87178					
[I].YPFVEPIPYGFLPQNILPL.[A]_	P05814						31745	27072		25397	58208										
[I].YPFVEPIPYGFLPQNILPLA.[Q]_	P05814						147419	162043	85631	78283	48115	351218	305607	211610	188229	136004		32504	24567		23567
[I].YPFVEPIPYGFLPQNILPLAQP.[A]_	P05814						71304	95971	161537	39948	28870										
[I].YPFVEPIPYGFLPQNILPLAQPAVVLPVPQPEI.[M]_	P05814						137255	116517	130090	73914		86714	77002	50570	88554	55208					34940
[I].YPFVEPIPYGFLPQNILPLAQPAVVLPVPQPEIMEVPKAK.[D]_	P05814						151071		143983			70900									
[I].YPSFQPQPLI.[Y]_	P05814						213934	174009	83187	126914	147751	105247	120753	126465	207991	147922					
[I].YPSFQPQPLIYPFVEPIPYG.[F]_	P05814											39899	33461	51129	52518	83214					
[I].YPSFQPQPLIYPFVEPIPYGF.[L]_	P05814											43577	78043	58995	72162	38914					
[I].YPSFQPQPLIYPFVEPIPYGFLPQNILP.[L]_	P05814						133590	117933	114257	33044	77519	59877	115555	92661	52919	23030					
[I].YPSFQPQPLIYPFVEPIPYGFLPQNILPLA.[Q]_	P05814											155623	179051	181985	106959	38890					
[I].YPSFQPQPLIYPFVEPIPYGFLPQNILPLAQ.[P]_	P05814												51238	54962							
[I].YPSFQPQPLIYPFVEPIPYGFLPQNILPLAQPA.[V]_	P05814						164885			63192	8376										
[I].YPVTQPLAPVHNP.[I]_	P05814											51929	111855	50225	37217						
[K].AAVPNSQNATISVPPLTSVSVKPQL.[G]_	Q8N9U0						59343	51705	69374	26252	59438										
[K].ADTRKMDPSKPSSNVAGVVIIV.[I]_	P22897																27645	34743	27261	21674	16650
[K].AIGYLNTGYQR.[Q]_	P01023																22643		20310		10646
[K].AIPVAQDLNAPS.[D]_	P10451						149730	163215	54160	42363	14694	59862	54414	54857	31793	16324					
[K].AIPVAQDLNAPSD.[W]_	P10451						138719	148724	74766	209928	172851										
[K].AIPVAQDLNAPSDWDSR.[G]_	P10451						275354	305992	241092	430424	351248	128307	134599	117271	87230	45051					
[K].AIPVAQDLNAPSDWDSRGK.[D]_	P10451									72900	44061										
[K].AIPVAQDLNAPSDWDSRGKD.[S]_	P10451						35538			53981	30840										
[K].AIPVAQDLNAPSDWDSRGKDS.[Y]_	P10451						1270986	1078997	617389	190628	95132	4084556	4025281	4109876	2811443	1727643					
[K].AIPVAQDLNAPSDWDSRGKDSYETS.[Q]_	P10451														39186	663676					
[K].AIPVAQDLNAPSDWDSRGKDSYETSQ.[L]_	P10451											67140	88663	85965	61758	65036					
[K].AIPVAQDLNAPSDWDSRGKDSYETSQL.[D]_	P10451						332779	346669	337872	362415	409308	328309	323040	327679	352249	514929					
[K].AIPVAQDLNAPSDWDSRGKDSYETSQLD.[D]_	P10451											201804	192277	203378	194056	204916					
[K].AIQDPRLFAEE.[K]_	P01833						101008	74750	95962	80495	46699	123635	105560	68992	70075	28048	149042	151988	155173	55068	20087
[K].AIQDPRLFAEEKA.[V]_	P01833						181464	77090		255069	163490	18724	32510	35249	13078	15882					

Quantified modified peptide sequence	Protein ID	A_-80oC	A_-20oC_120h	A_4oC_6h	A_RT_6h	A_RT_24h	B_-80oC	B_-20oC_120h	B_4oC_6h	B_RT_6h	B_RT_24h	C_-80oC	C_-20oC_120h	C_4oC_6h	C_RT_6h	C_RT_24h	D_-80oC	D_-20oC_120h	D_4oC_6h	D_RT_6h	D_RT_24h
[K].AIQDPRLFAEEKAVAD.[T]_	P01833						37635			54831	50357										
[K].AIQDPRLFAEEKAVADTRDQ.[A]_	P01833											776865	847898	703665	654423	334933	601627	889308	609284	451392	508252
[K].AIQDPRLFAEEKAVADTRDQA.[D]_	P01833		202924	61292		209331	72973	113059	133189	145721	466229		239380		14415						
[K].AIQDPRLFAEEKAVADTRDQADG.[S]_	P01833											288118	274044	251597	199342		101153	90120	100274	61613	60449
[K].AIQLTYNPDESSKPNMIDAATL.[K]_	P10451							75529	57158	48462	51175										
[K].AIQLTYNPDESSKPNMIDAATLK.[S]_1xOxidation [M16]	P10451																	24080	17821	48312	31712
[K].AKDQTKAQAAAPASVPAQAPK.[R]_	P10451											79241	59417			32801					
[K].AKDTVYTKGR.[V]_	P05814						11975	9145	46822	11008											
[K].AKDTVYTKGRVM.[P]_	P05814									30685											
[K].AKDTVYTKGRVMPV.[L]_	P05814						40365	47084	122913	112159	72684										
[K].AKDTVYTKGRVMPVLKSPTIP.[F]_	P05814											27230	69078	29426	77118	226105					
[K].AKDTVYTKGRVMPVLKSPTIPFFDPQIP.[K]_	P05814												40552		58466	155944					
[K].ALENPQPHPGWQGTL.[K]_	P19835						59180	64869		66712	139309										
[K].ALENPQPHPGWQGTLKAKNF.[K]_	P19835					62149	44601	31442		148607	216122										
[K].ANDESNEHSDVIDSQELS.[K]_	P10451									30686	73268										
[K].ANDESNEHSDVIDSQELSK.[V]_	P10451						38642	39198	63757	107162	141392	38916	37817	36057	67620	94978					
[K].ANDESNEHSDVIDSQELSKVS.[R]_	P10451											194232	187039	182498	195178	108468					
[K].ATLHAVDVTLDPDTAHPHLFLYEDS.[K]_	Q13410										25326										
[K].ATLHAVDVTLDPDTAHPHLFLYEDSK.[S]_	Q13410						263964	406205	400615	285152	298381										
[K].AVADTRDQADGS.[R]_	P01833											22381	21918	15908	17062		53980	87276	30469	15705	
[K].AVADTRDQADGSRASV.[D]_	P01833						27588	29370		23575		15063			15217						
[K].AVADTRDQADGSRASVDSGSSEE.[Q]_	P01833		69366					41002													
[K].AVADTRDQADGSRASVDSGSSEEQGGSSR.[A]_	P01833																389614	333949	400401	227485	211735
[K].AVADTRDQADGSRASVDSGSSEEQGGSSRALVSTLVPLG.[L]_	P01833						14377876	13221863	29794847	8881285	5194191	10513627	12580805	10339068	9160067	5281495	6748440	5768151	6836615	4669557	4012003
[K].CAEQFCRLNEYNQLQLQAAHAQEQ.[I]_	P47710						76070	74172													
[K].DAMAKIQAKIPGLK.[R]_	Q9NQC3											50056	41177	51690	37875	31352					
[K].DGWLHTGDIGKWLPNGTLK.[I]_	P33121							36858		41474	27073	117690	134278	110452	141546	173679					34719
[K].DHEELSLVASEAVRAIEK.[I]_	Q96IY4						73258	95416													
[K].DLKEGLLLYHTENNVGLK.[N]_	O75954											118130	99481	107800	70718	48628	109279	125570	76897	52109	62719
[K].DLSRFDASFFGVHP.[K]_	P49327									28138	24072										
[K].DLSRFDASFFGVHPK.[Q]_	P49327						54198			47092	41657										
[K].DLYANTVLSGGTTMYPGIADRMQ.[K]_	P60709						41796				65085										
[K].DLYANTVLSGGTTMYPGIADRMQK.[E]_	P60709											110856	162504	116692	133356	123269					
[K].DPPADVQLFQEVPK.[G]_	P47989						110400	120073	66493	89415	88022										
[K].DQYPYLK.[S]_	Q99541											616651	761777	1407381	692427	250717					
[K].DSGRDYVSQFEGSALGK.[Q]_	P02647											88010	85748	67927	83440	9726					
[K].DSGRDYVSQFEGSALGKQLNLK.[L]_	P02647											113578	164181	120789	69773	40097					
[K].DSHSLTTNIMEILRGDFSSAN.[N]_	P02671									34569											
[K].DSSSKNLIPR.[L]_	P15907						58826	49501	54800	25592	19441										
[K].DSYETSQLDDQSAETHSH.[K]_	P10451											75282	83436	85822	90845	33186					
[K].DSYETSQLDDQSAETHSHK.[Q]_	P10451											96145	92080	78808	104007	63916					
[K].DSYETSQLDDQSAETHSHKQS.[R]_	P10451						264900	308947	215505	325988	132961	286347	266854	253974	295371	98264					
[K].DSYETSQLDDQSAETHSHKQSRLY.[K]_	P10451						32334	33123	41305	103136	106645	144742	137907	138534	109549	63662					
[K].DTGKTPVEPEVAIHR.[I]_	P60866						83220	68811	235383	66716	87857	69241	56749	58237	79679	53930					
[K].DTLGNTQIKQ.[K]_	Q14512											173126	185668	190795	194299	77085					
[K].DTVYTKGRVM.[P]_	P05814						2447774	1896179	526745	3337207	673529	233999	263145	194846	719663	497406					
[K].DTVYTKGRVM.[P]_1xOxidation [M10]	P05814				114037	19279	100891	78229		117832	13949				22478	22144					
[K].DTVYTKGRVMP.[V]_1xOxidation [M10]	P05814			25859	137018	40978	40912	48828	77285	46854	28209	9448	20690		11263	26562					
[K].DTVYTKGRVMPVLKSP.[T]_	P05814					37375	47048	45500		42454	43032	112789	148082	131291	190914	370754					
[K].DTVYTKGRVMPVLKSPT.[I]_	P05814												16092		10543	41723					
[K].DTVYTKGRVMPVLKSPTI.[P]_	P05814															37711					
[K].DTVYTKGRVMPVLKSPTIP.[F]_1xOxidation [M10]	P05814											21691	22627	31982	18380	90918					29068
[K].DTVYTKGRVMPVLKSPTIPF.[F]_	P05814						96651	77919	141961	105130	32699										
[K].DTVYTKGRVMPVLKSPTIPFF.[D]_	P05814						3937822	3375359	8405186	1740388	1472135										
[K].DTVYTKGRVMPVLKSPTIPFFD.[P]_	P05814					37191										77624					
[K].DVTVLQNTDGNNNEAWAKDLK.[L]_	P02788			30287		28157	141565	114979	95828	131045	188016	38608	69395	62440	49589	38483					
[K].EAKKVEGFDLVQ.[K]_	Q99541											79410	89858	74066	66820	32113					
[K].EAKQKSQQTISQLH.[S]_	Q99541											30670	44403	43174	33834	14707					
[K].EAQLPVIENK.[V]_	P00747																	16949			
[K].EAQPGQSQVSYQGLPVQK.[T]_	Q14766											26695		26244	45058	35093					
[K].EDGLAQQQTQLNLR.[S]_	P49327						63182	59046		97934	71684	65665	57181	70330	74860	23613					
[K].EEDKHLKF.[R]_	P10451						177709	181453	97037	559959	176128										
[K].EEDKHLKFRIS.[H]_	P10451											248331	302931	338126	242775	106507					
[K].EGAEMESSKEDKARQAEVK.[R]_	Q9H173						21192	31916	51871	46107	35840										
[K].EIPLSPMGEDSAPRDADTLH.[S]_1xOxidation [M7]	Q13410											59809	69993	87779	67174	61431					
[K].EIPLSPMGEDSAPRDADTLHSKLIPTQ.[P]_	Q13410											165754	142429	163489	137913	68208					
[K].EVSDSLLTSSKGQLQ.[K]_	Q99541						30612	18683		26976	66494										
[K].FKEGAEMESSKEDK.[A]_	Q9H173									20337	10054										
[K].FMETVAEKALQEYR.[K]_	P10909						14667		94171	32854	29452										
[K].FQLFGSPSGQKDLLFK.[D]_	P02788						98442	67171	202771			32041	147139								
[K].FRISHELD.[S]_	P10451						181168	146825	138107	408438	77410										
[K].FRRPDIQYP.[D]_	P10451						87444	88805	100592	81651	154814										
[K].FRRPDIQYPDATD.[E]_	P10451						1569020	1374194	731527	1959378	2047358	90718	66281	67446	119140	171520					
[K].FRRPDIQYPDATDEDITSH.[M]_	P10451						163038	172910	105294	79743	51982										

Quantified modified peptide sequence	Protein ID	A_-80oC	A_-20oC_120h	A_4oC_6h	A_RT_6h	A_RT_24h	B_-80oC	B_-20oC_120h	B_4oC_6h	B_RT_6h	B_RT_24h	C_-80oC	C_-20oC_120h	C_4oC_6h	C_RT_6h	C_RT_24h	D_-80oC	D_-20oC_120h	D_4oC_6h	D_RT_6h	D_RT_24h
[K].FRRPDIQYPDATDEDITSHMESEELNGA.[Y]_	P10451						49090	44986	87712	41774	17607	69209	44039	58117	71545	22367					
[K].FRRPDIQYPDATDEDITSHMESEELNGAY.[K]_1xOxidation [M20]	P10451							45401	85828												
[K].FRRPDIQYPDATDEDITSHMESEELNGAYK.[A]_	P10451						131213	151847	192676	250445	77008				16918	28332					
[K].GAGSTTVKIVL.[K]_	Q6WN34						78087	45569		59740	97525										
[K].GAGSTTVKIVLKEK.[H]_	Q6WN34							19316		34468	31868										
[K].GAGSTTVKIVLKEKH.[K]_	Q6WN34					25715		15440		21887	42062										
[K].GAVTGAKDAVTTTVTGAKDSVASTITGVMDK.[T]_	Q99541						136211		225671		148661	69118	53646	78700	43630	64504					
[K].GIPFAAPTKALE.[N]_	P19835						104587	93538		104676	94937										
[K].GIPFAAPTKALENPQPHPGWQ.[G]_	P19835						64720	60686			92422										
[K].GIPFAAPTKALENPQPHPGWQG.[T]_	P19835						202238	250197	119147	137321	240607										
[K].GKRLDINTNTYTSQDLK.[S]_	Q9H173						31806	40431		93180	62882										
[K].GLDRIEERLPIL.[N]_	Q99541											122558	125497	98380	124086	79940					
[K].GLDRIEERLPILNQPSTQIVA.[N]_	Q99541											175680	158391	168507	139485	100173					
[K].GLDRIEERLPILNQPSTQIVANA.[K]_	Q99541						69595	67117	63897	50994	120677										
[K].GLRGAKTTFDVYTESWAQDPSQEN.[K]_	P19835						182143	215570	156841	497866	892653										
[K].GLVQALQTKVTQQGLK.[M]_	P49327										57462						15044	13293	20288	38554	23718
[K].GNKKVTEEDFY.[K]_	P19835						18810			47908	46876										
[K].GNKKVTEEDFYK.[L]_	P19835						41109	42775	234014	120202	81675	52954	52132	11439	29173	25654					
[K].GNKKVTEEDFYKLVSEFTITK.[G]_	P19835					42945					136004										
[K].GRVMPVL.[K]_	P05814						1273421	1197328	1147145	2064242	3730374										
[K].GRVMPVLKS.[P]_	P05814											142500	170955	141399	323243	231629					
[K].GRVMPVLKSPTI.[P]_	P05814														33260	51297					
[K].GRVMPVLKSPTIPF.[F]_	P05814						324246	332399	533548	440524	583543	100722	69922	69914	56456	71810					
[K].GRVMPVLKSPTIPFFD.[P]_	P05814					150361	74046	147275	171882	165157	374716				30742	174616					21774
[K].GRVMPVLKSPTIPFFDPQ.[I]_	P05814															101893					
[K].GRVMPVLKSPTIPFFDPQI.[P]_	P05814						36171	37082	52108	94806	200089					184854					
[K].GRVMPVLKSPTIPFFDPQIPKL.[T]_	P05814					427415	403853	488836	669109	734670	1349407	268221	255201	276901	556077	3503700	122114	84406	102709	393971	155996
[K].GRVMPVLKSPTIPFFDPQIPKL.[T]_1xOxidation [M4]	P05814															142548					
[K].GRVMPVLKSPTIPFFDPQIPKLTD.[L]_	P05814			36111		807502	1075182	1234159	1819323	1858783	3157527	942954	914835	788994	1137731	6945964					
[K].GRVMPVLKSPTIPFFDPQIPKLTD.[L]_1xOxidation [M4]	P05814											22996	32670		34419	283133					
[K].GRVMPVLKSPTIPFFDPQIPKLTDL.[E]_	P05814					126615	181936		320865	218174	303342	104895			164471	702499					
[K].GRVMPVLKSPTIPFFDPQIPKLTDLEN.[L]_	P05814		42442			399416	509541	559321	1058928	697738	940590	38862			43172	240736					
[K].GRVMPVLKSPTIPFFDPQIPKLTDLENL.[H]_	P05814					181238					357277	50315		42447	55529	448115					
[K].GRVMPVLKSPTIPFFDPQIPKLTDLENLHLPLP.[L]_	P05814		36942		87536	71359	111954	99830	715094	357146	283437										
[K].GRVTGWGNLKETWTANVGK.[G]_	P00734					22026				33007	55133										
[K].HEDQQQGEDEHQDKIY.[P]_	P05814					30520															
[K].HEDQQQGEDEHQDKIYPS.[F]_	P05814						249243	310027	349289	282308	110988	663679	678491	612058	782152	308604	173879	220471	213022	161503	170162
[K].HEDQQQGEDEHQDKIYPSF.[Q]_	P05814											117091	122030	86262	90964	47501		26465	17061		
[K].HEDQQQGEDEHQDKIYPSFQPQPLI.[Y]_	P05814											71374									
[K].HGSYEDAVHSGALND.[-]_	P17987						36877	52448		73096	71039										
[K].HIGKVVVQVLAEEPEAVLK.[G]_	P49327						22918	26405		45724	41814										
[K].IGGIGTVPVGRVETGVLKPGMVVT.[F]_	P68104														36137	70096					
[K].IYPSFQPQPLIYPFVEPIPY.[G]_	P05814						138573	109425		99609	73715										
[K].IYPSFQPQPLIYPFVEPIPYGFLPQNILPLA.[Q]_	P05814									26764	126506	26953			61090	176136					
[K].IYPSFQPQPLIYPFVEPIPYGFLPQNILPLAQ.[P]_	P05814															104580					
[K].IYPSFQPQPLIYPFVEPIPYGFLPQNILPLAQP.[A]_	P05814					107978			66951		112999										
[K].KLGLLGDSVDIFK.[G]_	P19835							90275	89160	183567	102175	35376									
[K].KPPPSPQPTEEDPEHR.[V]_	Q13438							32649		32740	19801										
[K].KSADTLWDIQKDLKDL.[-]_	P07195								107224	55459											
[K].KTVVDFETDVLFLVPTEIALAQH.[R]_	P19835						29344		253465		146564										
[K].KTVVDFETDVLFLVPTEIALAQHR.[A]_	P19835						44032	65891		32888	119615										
[K].KTVVDFETDVLFLVPTEIALAQHRANA.[K]_	P19835							19462		186226	258072										
[K].KVEISIPASSLPRLTP.[W]_	Q13410											56387	50092	51879	50306		41654	79216	47187	40770	26753
[K].KVEISIPASSLPRLTPWIV.[A]_	Q13410																	52553	39019		32401
[K].KVEISIPASSLPRLTPWIVAVA.[V]_	Q13410																345927	319462	256676	99548	153037
[K].KVSPAVLVHRDG.[R]_	Q13410											34027	31808				18238	106582	42474		
[K].LGAVYTEGGFVEGVNKKLGLLGDSVDI.[F]_	P19835							30562	125898	60399	75341										
[K].LGLLGDSVDIFK.[G]_	P19835					41153	188933	221506		462844	249741										
[K].LGLLGDSVDIFKGIPFAAPTK.[A]_	P19835						91526			320617	133919										
[K].LGLLGDSVDIFKGIPFAAPTKALENPQPHPGWQGTL.[K]_	P19835									522424	950477										
[K].LGLLGDSVDIFKGIPFAAPTKALENPQPHPGWQGTLK.[A]_	P19835										361937										
[K].LPGQGVHSQGQGPGANF.[V]_	Q92896											31535	35926	41782				25621	20951		
[K].LPLRYP.[E]_	P47710						31646	42165		18305	16299										
[K].LPLRYPE.[R]_	P47710						1138567	1304944	419116	359609	275717	41941	38548	34468	20077						
[K].LPLRYPERLQN.[P]_	P47710						62901	39953		29344	28091										
[K].LPLRYPERLQNP.[S]_	P47710						447797	405275	125298	131747	127444	315399	349854	281242	187547	116940					
[K].LPLRYPERLQNPS.[E]_	P47710						870447	862630	250841	163523	136387										
[K].LPLRYPERLQNPSESSEPIPLE.[S]_	P47710						979300	914370	1380659	615945	386510	587043	675044	637003	525507	435190					
[K].LRPVAAEVYGTERQPR.[T]_	P02788									36423	26328										
[K].LRRELDESLQVAER.[L]_	P10909						36525	29130		21891											
[K].LTDLENLHLP.[L]_	P05814						40444	47647		33089	49393	36938	43229	29488	23113	20887					
[K].LTDLENLHLPLPLL.[Q]_	P05814							49699		51506	58152										
[K].LTDLENLHLPLPLLQ.[P]_	P05814						57010	39393		66623	84394					51627					
[K].LTDLENLHLPLPLLQPL.[M]_	P05814						191789	121145	230350		125241	92060	89670	80367	50480	51294					

Quantified modified peptide sequence	Protein ID	A_-80oC	A_-20oC_120h	A_4oC_6h	A_RT_6h	A_RT_24h	B_-80oC	B_-20oC_120h	B_4oC_6h	B_RT_6h	B_RT_24h	C_-80oC	C_-20oC_120h	C_4oC_6h	C_RT_6h	C_RT_24h	D_-80oC	D_-20oC_120h	D_4oC_6h	D_RT_6h	D_RT_24h
[K].LTDLENLHLPLPLLQPLM.[Q]_1xOxidation [M18]	P05814						111835	74534		114937	142822		51168	51076	94887	129181					
[K].LTDLENLHLPLPLLQPLMQ.[Q]_1xOxidation [M18]	P05814											21740	31173	24042	19799	32873					
[K].LTDLENLHLPLPLLQPLMQQ.[V]_	P05814															63431					
[K].LTDLENLHLPLPLLQPLMQQVPQPIPQTLA.[L]_	P05814						93732	80679		75054				53643	47237						
[K].LTDLENLHLPLPLLQPLMQQVPQPIPQTLAL.[P]_	P05814						136347	123844	169954	126218	259539	29977		33912	33922	114923					
[K].LVSEFTITKGLRGAKTTFDVYTESWAQDPSQEN.[K]_	P19835									46658	176891										
[K].LVSEFTITKGLRGAKTTFDVYTESWAQDPSQENK.[K]_	P19835						52700			31353	69746										
[K].LYGSAGPPPTGEEDTAEKDEL.[-]_	P11021						40673		82524	18864	11452	42850	25613	59299	61477	37373					
[K].NGLHSKVVSEQKDTLGNTQIKQ.[K]_	Q14512						83625	56354	157157	86353	84307	95024	116711	118645	99682	99927					
[K].NHPVRFGSDDEGRYLTQETNKVETY.[K]_	P12272									43974	102071										
[K].NNRQPYAVSELAGHQTSAESWGTGR.[A]_	P36578							31246		29245	44539										
[K].NPKFMETVAEKALQ.[E]_	P10909						56744	53198		54580	67985	18187		22866	22572	15582					
[K].NPKFMETVAEKALQEY.[R]_	P10909						74943	28102	60760	82416	134769										
[K].NPKFMETVAEKALQEYR.[K]_	P10909											42374	38899	33852	34720						
[K].PSYYVRLG.[S]_	Q99541											265149	324981	267257	230702	75296					
[K].QKVEKVKHEDQQQGEDEHQDKIYP.[S]_	P05814							53015	147271	33712	28933										
[K].QLYNKYPDAVATWLNPDPSQK.[Q]_	P10451						22985	27327		128277	426469	125732	141488	76771	58412	39729					
[K].QNLLAPQNAVSSEETNDFK.[Q]_	P10451											18393	20268	19570	21463						
[K].REEAPSLRPAPPPISGGGY.[R]_	P02675									35240	37786										
[K].REEAPSLRPAPPPISGGGYR.[A]_	P02675						51788	59272		77716	36280										
[K].RGPGTPAPTGLSAPLSFIPRHF.[R]_	Q6WN34					132011	80547	82961	85265	230681	431358										
[K].RKANDESNEHSDVIDSQELS.[K]_	P10451						30799			38756	31804										
[K].SADTLWDIQKDLKDL.[-]_	P07195						88399														
[K].SGVAPQKMAGASPTKDDSKDSDFW.[K]_	Q13438					20714		201497			150160										
[K].SGVAPQKMAGASPTKDDSKDSDFWK.[M]_	Q13438						43829	53530	97951	82548	70249										
[K].SHALQLNNRQIR.[G]_	P0C0L4											330064	546423	367008	490726	241121					
[K].SKEEDKHLKF.[R]_	P10451						32450	31229		33025	8354										
[K].SKKFRRPDIQYPDATD.[E]_	P10451						257576	191838	223838	533586	227688										
[K].SLEDKTERELLESYIDGR.[I]_	P00734						73603	63687	377362	128791	50027										
[K].SPTIPFFDPQIPKLTDLE.[N]_	P05814						236347	156973		116828		451507	448563	433807	390458	401254					
[K].SPTIPFFDPQIPKLTDLENLHLPLPLLQPL.[M]_	P05814					572133	1617824	1658554	1853940	1260851	1651991	1921243	1602503	1597470	1419110	1276299				36472	60822
[K].SPTIPFFDPQIPKLTDLENLHLPLPLLQPLM.[Q]_1xOxidation [M31]	P05814		53376			157867	566616	720908	359144	500973	435129	447155	651660	580272	399948	686043					75085
[K].SPTIPFFDPQIPKLTDLENLHLPLPLLQPLMQ.[Q]_1xOxidation [M31	P05814					36319	277954	315134	260895	156913	99205	260147	441261	335756	264339	409640					
[K].SPTIPFFDPQIPKLTDLENLHLPLPLLQPLMQQ.[V]_	P05814						68631	27573		41998	42793	118104	64397	57725	96396	184431					
[K].SQLQKVPPEWK.[A]_	P02671					23315	95431	64799		148430	132567										
[K].SQLQKVPPEWKALTDMPQM.[R]_	P02671						32044	31997		58452	51588										
[K].SQQTISQLH.[S]_	Q99541											41883	48787	26110	33333	16632					
[K].SSLVTSKLAGGQVE.[-]_	O43768						30919	42085		43698											
[K].SSSYSKQFTSSTSY.[N]_	P02671						52262	47770	54911	60050	33701						32134	30243	23346	33359	10028
[K].SSSYSKQFTSSTSYN.[R]_	P02671																129868	109697	199651	72552	
[K].SSSYSKQFTSSTSYNRGDST.[F]_	P02671																	16751	18945		19244
[K].SSSYSKQFTSSTSYNRGDSTFESKSYK.[M]_	P02671		45981	60546	52090		29926			30290											
[K].SVRLEDSRQKLPEK.[T]_	Q13410											75702	121410	58565	50004	40032					
[K].TAPYVPMYYVPNSYPYYGTNLYQR.[R]_	P07498					565530		190083	1653753	374858	990790	553676	504911	770187	927914	1004595					
[K].TAPYVPMYYVPNSYPYYGTNLYQR.[R]_1xOxidation [M7]	P07498															214709					
[K].TAPYVPMYYVPNSYPYYGTNLYQRRPAIA.[I]_	P07498											39823	33797	27294	59669	91469					
[K].TGDPNMGDSAVPTHWEPYTTENSGYLEITK.[K]_1xOxidation [M6]	P19835						180626	197068	298088	250659	219387										
[K].TGDPNMGDSAVPTHWEPYTTENSGYLEITKK.[M]_	P19835					118707	568373	491402	536420	948588	745432	106945		101726	102115	119635					
[K].TGTVVALEVDHFSNVGNTQDLSQSIMER.[A]_	P47989						95429	97987	67798	74634											
[K].TLIEKTNEERKTLLSNLEEA.[K]_	P10909												37599		40977	46336					
[K].TPALIVYGDQDPMGQTSFEHLK.[Q]_	Q96IU4												23016	31359	14050	34966					
[K].TTFDVYTESWAQDPSQENKKK.[T]_	P19835						65831	37731		184215	151526										
[K].TVVDFETDVLFLVPTEIALAQH.[R]_	P19835						66165	35975	179453	64176	317115										
[K].TVVDFETDVLFLVPTEIALAQHR.[A]_	P19835					223642				424435	1597166				57673	70048					
[K].TVVDFETDVLFLVPTEIALAQHRANA.[K]_	P19835			151216		320997	761262	776468	976877	732513	2208878			119781	107643	162668					
[K].VAALGSDPHISM.[Q]_	Q13410											30390	35680	23051	12267		14576	61453	27454		
[K].VAALGSDPHISMQVQE.[N]_	Q13410							13850			56319										
[K].VEGFDLVQKPSYYVRLG.[S]_	Q99541											27392	41839	43703	42275	26450					
[K].VEISIPASSLPRLTPWIVAV.[A]_	Q13410																89698	77981	98120	326783	44619
[K].VEISIPASSLPRLTPWIVAVA.[V]_	Q13410			159445				129667									683043	659970	691771	530639	307384
[K].VEKVKHEDQQQGEDEHQDK.[I]_	P05814					25409	39191	42944	120194	95254	17734										
[K].VEKVKHEDQQQGEDEHQDKIYP.[S]_	P05814					19633	47006	45137	105786	65544	35819										
[K].VEKVKHEDQQQGEDEHQDKIYPSFQPQP.[L]_	P05814								92968	52783	16283										
[K].VEQAVETEPEPELR.[Q]_	P02649							45720	83204	102973											
[K].VETYKEQPLKTPGKK.[K]_	P12272											30371	54520	33962	46398	37321					
[K].VKHEDQQQGEDEHQD.[K]_	P05814								36613		7644										
[K].VKHEDQQQGEDEHQDKIYPS.[F]_	P05814					26281	213765	246205	321120	205074	107852	471491	559122	473215	519514	485617		46532	25763		32403
[K].VKHEDQQQGEDEHQDKIYPSF.[Q]_	P05814						78233	36372	94727	72532	87824	72286	88607	55132	74008	77545					
[K].VKHEDQQQGEDEHQDKIYPSFQPQ.[P]_	P05814		43502	87239		52947		48362		51809	72819										
[K].VKHEDQQQGEDEHQDKIYPSFQPQPLI.[Y]_	P05814		86284	61583		17433						66649	76166	65889	58880	53700					
[K].VLDSGFREIENK.[A]_	P01833						129528	96238	96194	192056	141263	69875	279481	47888	50684	18156	82812	152119	148486	36471	63773
[K].VLPIPQQVVPYPQRA.[V]_	P05814											140304	164170	171619	322758	475311	50030	21201	37913	73751	164360
[K].VLPIPQQVVPYPQRAVP.[V]_	P05814																35457	37202	30441	62882	199931
[K].VLPIPQQVVPYPQRAVPVQALLLNQEL.[L]_	P05814								70469	83707	146031		122035			264015					

Quantified modified peptide sequence	Protein ID	A_-80oC	A_-20oC_120h	A_4oC_6h	A_RT_6h	A_RT_24h	B_-80oC	B_-20oC_120h	B_4oC_6h	B_RT_6h	B_RT_24h	C_-80oC	C_-20oC_120h	C_4oC_6h	C_RT_6h	C_RT_24h	D_-80oC	D_-20oC_120h	D_4oC_6h	D_RT_6h	D_RT_24h
[K].VLPIPQQVVPYPQRAVPVQALLLNQELL.[L]_	P05814												131739	96525		261401					
[K].VLPIPQQVVPYPQRAVPVQALLLNQELLL.[N]_	P05814						54860	36916			95933	125702	163060	127637	107341	190036					
[K].VLPIPQQVVPYPQRAVPVQALLLNQELLLNPT.[H]_	P05814						59139	49096	178487	47671	40637	218530	230849	206946	176003	434063					
[K].VLPIPQQVVPYPQRAVPVQALLLNQELLLNPTH.[Q]_	P05814		39646			214724		166516	202110	359050		445852		414953		1393677					
[K].VLPIPQQVVPYPQRAVPVQALLLNQELLLNPTHQ.[I]_	P05814											63743	51051	68299		59168					
[K].VPEWVDTVKL.[A]_	P39019											16253	26003	10911	24135	10231					
[K].VPPEWK.[A]_	P02671						90682	84774	69770	126899	56454										
[K].VQAAVGTSAAPVPSDNH.[-]_	P02649								91660	47161											
[K].VSFLSALEEYTKKLNTQ.[-]_	P02647							76703	184634	197820	178832										
[K].VSPAVLVHRDG.[R]_	Q13410																18089	46094	19578		
[K].VSPAVLVHRDGREQEAEQMPEYR.[G]_	Q13410									63376	36964										
[K].VTEEDFYK.[L]_	P19835									50285	18536										
[K].VTEEDFYKLVSEFTITKGL.[R]_	P19835						22764	30376		41267	115072										
[K].VTEEDFYKLVSEFTITKGLRGA.[K]_	P19835										610302										
[K].VVSEQKDTLGNTQIKQK.[S]_	Q14512											55280	75413	63087	77559	37216	18140	21027			18120
[K].YPDAVATWLNPDPSQKQNLLAPQ.[N]_	P10451											156930	137894	190310	161169	85559					
[K].YPIEHGIITNWD.[D]_	P68133											16017	24630		18249	35842					
[KA].LPIIQK.[RL]_	Q99541											537051	693748	856088	637336	228664					
[KA].LPLIQK.[RL]_	Q99541						65693	73939	51553	31188	12711										
[L].ADTKELVSSKVSGAQ.[E]_	O60664											29629	25407		48758						
[L].ADTKELVSSKVSGAQEMVS.[S]_	O60664											104481	106742	145990	143994	122549					
[L].AIESQDAGIKTITMLD.[E]_	O00161											21699	23995	23290	21202						
[L].ALPPQPLWSVPQP.[K]_	P05814											149349	123508	97541	222590	318682					
[L].ALPPQPLWSVPQPKV.[L]_	P05814																		18391		185791
[L].ALPPQPLWSVPQPKVL.[P]_	P05814						208433	125753	576945	69385	50661										
[L].ALPPQPLWSVPQPKVLPIPQQ.[V]_	P05814													308457	322998	1319390					
[L].ALPPQPLWSVPQPKVLPIPQQV.[V]_	P05814											35497	39605	52160	122365	348422		10334	17444		44739
[L].ALPPQPLWSVPQPKVLPIPQQVVPYP.[Q]_	P05814						56563	36616	122623	259984	27468										
[L].ALPPQPLWSVPQPKVLPIPQQVVPYPQR.[A]_	P05814						2076057	2211052	1956214	2090041	4743781		46992		8356	24843	353050	333147	325725	295425	286917
[L].ALPPQPLWSVPQPKVLPIPQQVVPYPQRA.[V]_	P05814											125767	174988	141490	145070	288676					
[L].ALPPQPLWSVPQPKVLPIPQQVVPYPQRAV.[P]_	P05814						114395	78871	130701	105315				50687	207429	612121					113035
[L].ALPPQPLWSVPQPKVLPIPQQVVPYPQRAVPVQ.[A]_	P05814																72598	97255	54653	70308	833129
[L].APQNAVSSEETNDFK.[Q]_	P10451											78239	88933	67859	85160	76152					
[L].APVHNPI.[S]_	P05814						131158	165974	101656	31123	27849										
[L].APVHNPISV.[-]_	P05814						12241895	12025568	9081944	9424618	8871386	11283883	12390129	12874609	12046359	18659908	5766202	5506024	5554716	7071399	5825784
[L].AQPAVVLPVPQPEIMEVPK.[A]_	P05814				467592	1087148	51229	68801	977548	1585968	2911984	1913499	2226493	2378942	2590039	1326032		151183		309013	118785
[L].AQPAVVLPVPQPEIMEVPKA.[K]_	P05814		25866			172926	434514	442860		324141	483238	145404	96056	92611	147740	175914					
[L].AQPAVVLPVPQPEIMEVPKAK.[D]_	P05814						76848	70794	81520	81544	78013	86051	83714	66167	82454	72765					
[L].AQPAVVLPVPQPEIMEVPKAKDTVYTK.[G]_	P05814						214376	193947	596824	136665	67309	353811	372023	307371	308201	228344	22227	38332	20481	23948	40478
[L].AQPAVVLPVPQPEIMEVPKAKDTVYTKG.[R]_	P05814						88894	95078	191212	51623											
[L].DAPPPPAAPLPRWSGPIGVSWG.[L]_	Q99523																	44358			
[L].DDFQKKWQEEMELYR.[Q]_	P02647											29934	30637								
[L].DDQSAETHSHK.[Q]_	P10451											148638	128493	67808	103483	108267					
[L].DDQSAETHSHKQS.[R]_	P10451											569881	585790	427800	412512	298253					
[L].DDYVNTQGASLFSVTKK.[Q]_	P00747											28048	34671	28831	20838	20680					
[L].DESLQVAERL.[T]_	P10909						23166	34370		23185	46937		37195	30136	28196						
[L].DESLQVAERLT.[R]_	P10909		54554	49533	121657	22468	96885	107962	66705	101882	178532	77402	78926	84015	63050	54032					
[L].DESLQVAERLTRKYNE.[L]_	P10909						50537	39905		28203	26163	72203	68726	63578	67169	49685					
[L].DESLQVAERLTRKYNELL.[K]_	P10909							372804	2591603	304507	138584										
[L].DEVKEQVAEV.[R]_	P02649						57388	108638		111174	37957										
[L].DEVKEQVAEVR.[A]_	P02649						74231	93337		38585	18863										
[L].DGAQIPRDPSQQELPR.[L]_	P49327						145054	189399	67598	41377	36469										
[L].DGGFIYEAGLAPYK.[L]_	P02788							25357	34109	36362	98565										
[L].DIKGIDYW.[L]_	P00709						28114	43834		60005	40644										
[L].DIKGIDYWLAHK.[A]_	P00709									69435											
[L].DKIDVIKQ.[A]_	Q5JWF2																21351	28864	26550		11954
[L].DKLEENLPIL.[Q]_	O60664											34652	39572	59650	31349						
[L].DKLTVTSQNLQLENLR.[M]_	P04233		56051			26215															
[L].DPDTAHPHL.[F]_	Q13410											61469	73966	89508	59420	35704					
[L].DPDTAHPHLFLY.[E]_	Q13410											483452	567441	311122	399090	187151					
[L].DRIEERLPIL.[N]_	Q99541						176428	210275	119484	130005	76278	1602468	1706924	1954743	1349324	923175					
[L].DRIEERLPILN.[Q]_	Q99541											947942	976786	1185195	957606	601857					
[L].DRLAYIAHPKLG.[K]_	P47914										32964										
[L].DSGFREIE.[N]_	P01833						26717	41314		103837	72836										
[L].DSGFREIENK.[A]_	P01833						526811	641584	810177	863449	507239	102879	349185	120474	119433	42239					
[L].DSGFREIENKAIQDPR.[L]_	P01833						265750	262876	244270	612872	1204972										
[L].DSGVTGSGLEGDH.[L]_	P12272																29125	28308	28314		
[L].DSGVTGSGLEGDHLS.[D]_	P12272																30598	31538	35992	38738	18161
[L].DTAVENMPSLK.[M]_	P49327							25140		36065	44363										
[L].DTSPSEETDQEVFLGPPEAQSFLSSHTRIPR.[A]_	O14669						64284	54049													
[L].EDKNSPFYYDWHS.[L]_	Q14802																32500	24292	23180		
[L].EDKTERELLESYIDGR.[I]_	P00734						84231	86004	169625	454180	252962							11719			18154
[L].EEELQFSLGSKIN.[V]_	P0C0L4											102573	90201	135314	326577	415321					
[L].EEELQFSLGSKINVK.[V]_	P0C0L4									24833	51063	184263	138485	226547	258580	191917					

Quantified modified peptide sequence	Protein ID	A_-80oC	A_-20oC_120h	A_4oC_6h	A_RT_6h	A_RT_24h	B_-80oC	B_-20oC_120h	B_4oC_6h	B_RT_6h	B_RT_24h	C_-80oC	C_-20oC_120h	C_4oC_6h	C_RT_6h	C_RT_24h	D_-80oC	D_-20oC_120h	D_4oC_6h	D_RT_6h	D_RT_24h
[L].EEELQFSLGSKINVKVGGNSK.[G]_	P0C0L4											25530		20678	41416	48389					
[L].EEELQFSLGSKINVKVGGNSKGTL.[K]_	P0C0L4											134540	127975	107990	103634	246775					
[L].EEQAQQIRLQAEAFQARLK.[S]_	P02649						56806		67152												
[L].EEYTKKLNTQ.[-]_	P02647						25239	30914		59436	26900										
[L].EIPGNSDPNMIPDGDFNSYVR.[V]_	P0C0L4											56454	40401	57337	43869	28369					
[L].ENLHLPLPLL.[Q]_	P05814						386473								166516	262898					56627
[L].ENLHLPLPLLQ.[P]_	P05814						292835	235360		222663	298539	32730			264353	569671					110932
[L].ENLHLPLPLLQPLM.[Q]_	P05814					27746	246007	170588		145476	166635	55212	63216	96821	394773	516060					244810
[L].ENLHLPLPLLQPLMQ.[Q]_	P05814						93325			80953	7402192				277834	973142					232861
[L].ENLHLPLPLLQPLMQQ.[V]_	P05814											16154	32696	19173	17767	47085					
[L].ENLHLPLPLLQPLMQQV.[P]_	P05814						75468					23331	35322		40129	37680					
[L].ENLHLPLPLLQPLMQQVPQPIPQTLA.[L]_	P05814											23414		25867							
[L].ENLHLPLPLLQPLMQQVPQPIPQTLAL.[P]_	P05814											143466	120182	153083	119773	23697					
[L].ENLHLPLPLLQPLMQQVPQPIPQTLALPPQPLW.[S]_	P05814											174009	58218	184957	135462	104890					
[L].ENPQPHPGWQG.[T]_	P19835						161591	173414	46245	161650	150087										
[L].ENPQPHPGWQGTLKAKNF.[K]_	P19835					50795	93113	86530	91325	144974	162912					30527					
[L].EPAALGALPLLR.[S]_	Q2I0M4											65598	75003	66761	15989			37950	30086		
[L].EPSANMPWFK.[G]_	P68104											72507	78973	68248	46810	36136					
[L].ERGEKLDDLVSKSEVLG.[T]_	O15498											28718	25034	36364	21518	18142					
[L].ESREEYMNGMN.[R]_	P47710					24614	88977	114305		84834	68678	58387	77914	47300	49799	35659					
[L].ESREEYMNGMNR.[Q]_	P47710						154540	445240	979253	1171844	351148	28149	101408	27368	73973	67862					
[L].ESREEYMNGMNRQRNIL.[R]_	P47710					47325	66449	71924		119453	55881										
[L].FAEEKAVADTRD.[Q]_	P01833						22674	26123				28705	25340		33231						
[L].FAEEKAVADTRDQADGSRASVDSGSSEEQGGSSR.[A]_	P01833						35443	53149	179676												
[L].FDSDPITVTVPVEVSRKNP.[K]_	P10909											47052	48931	55931	73174	36011					
[L].FLVPTEIALAQHRANA.[K]_	P19835					18588	37836	30032		40389	96499										
[L].FPNTSDPQKE.[N]_	Q8N387						36278	55915	58257	31081	33029										
[L].GAVYTEGGFVEGVNKKLG.[L]_	P19835						92073	103020		71109	95240										
[L].GAVYTEGGFVEGVNKKLGLLG.[D]_	P19835						39263	22576	83259	51968	53329										
[L].GAVYTEGGFVEGVNKKLGLLGDSVDI.[F]_	P19835							119963		106461	69660										
[L].GAVYTEGGFVEGVNKKLGLLGDSVDIFK.[G]_	P19835					58041	831611	855104	971465	717289	239166										
[L].GDSVDIFKGIPFAAPTK.[A]_	P19835									205269	246823										
[L].GLLGDSVDIFK.[G]_	P19835						86222	96103	104290	215304	134324										
[L].GLLGDSVDIFKGIPFA.[A]_	P19835						38967				33879										
[L].GLLGDSVDIFKGIPFAAPT.[K]_	P19835						42807	42122		84763	190731										
[L].GLLGDSVDIFKGIPFAAPTK.[A]_	P19835							151438	105900	832252	994008										
[L].GLLGDSVDIFKGIPFAAPTKALENPQPHPGWQGTL.[K]_	P19835						1482620	1296896	597529	2255354	2993014										
[L].GPPEAQSFLSSHTRIP.[R]_	O14669											36448	31954	33971	34963	18095					
[L].GSDPHISMQVQE.[N]_	Q13410						23445	44100		36983	30710										
[L].HAVDVTLDPDTAHPHLF.[L]_	Q13410											76521	73072	96231	44110	29960					
[L].HAVDVTLDPDTAHPHLFLY.[E]_	Q13410													55157	22078	16675					
[L].HLPLPLLQPLMQQVPQPIPQT.[L]_	P05814			200731		300446	1124843	1267347	769885	695616	543727	611401	477772	561196	568046	668801					
[L].HLPLPLLQPLMQQVPQPIPQTL.[A]_	P05814						568851	486024	557973	526555	663144	370258	305535	363621	591154	1914825	59485	29697	67188	84050	281030
[L].HLPLPLLQPLMQQVPQPIPQTLA.[L]_	P05814											735370	556066	507137	118796	221747					
[L].HLPLPLLQPLMQQVPQPIPQTLAL.[P]_	P05814						93326	53354				105762	98369	104912	119716	288904					
[L].HLPLPLLQPLMQQVPQPIPQTLALPPQP.[L]_	P05814						141349	136450		73355	60944	149550	165964	159635	121235	128112					
[L].HLPLPLLQPLMQQVPQPIPQTLALPPQPLW.[S]_	P05814											104836	95306	105713	97499	172388					
[L].HLPLPLLQPLMQQVPQPIPQTLALPPQPLWSVP.[Q]_	P05814											357127	236446	384542	224787	142487					
[L].HLSGIDANPNALFPPVEFPAPR.[G]_	P49327						65226	68375	116965	50445	24846										
[L].IEKTNEERKTLLSNLEEAK.[K]_	P10909											59493	55791	52410	40655	26177					
[L].IYPFVEPIPYGFLPQNILPLA.[Q]_	P05814											77771	84202	26901	16801	21239					
[L].KSPTIPFFDPQIPK.[L]_	P05814		32309	30710		189204	506636	564019	357499	1120861	1528667	23695			24135	52674					
[L].LERGEKLDDLVSKSEVLGTQ.[S]_	O15498											73224	66292	84812							
[L].LGDSVDIFK.[G]_	P19835						82587	73840		144671	74772										
[L].LGDSVDIFKGIPFAAPTK.[A]_	P19835						68812		210468	97756	143339										
[L].LLNPTHQIYPVT.[Q]_	P05814											1177159	2020178	1473885	1078510	921689					
[L].LLNPTHQIYPVTQPLAP.[V]_	P05814											35750	37436	41624	51325	74630					
[L].LLNPTHQIYPVTQPLAPVH.[N]_	P05814						102094	55790		82576	116007	24879	56933	41112	256992	931275	27320	11192	14851	26344	138971
[L].LLNPTHQIYPVTQPLAPVHN.[P]_	P05814											25186	14845	25361	16138	103024					
[L].LLNQELLLNPTH.[Q]_	P05814							78794			82632										
[L].LLNQELLLNPTHQIYPVT.[Q]_	P05814												19705	38378	179470	432367				34705	330835
[L].LLNQELLLNPTHQIYPVTQP.[L]_	P05814											17968				119514					
[L].LLNQELLLNPTHQIYPVTQPLAPVH.[N]_	P05814		44353	69567		134012	106378	94016	49139	117126	166210	55294	57640	96380	116684	684607					
[L].LLQCLYLFFVFPLDKDELL.[E]_	Q6ZS81						23947	28085	110374	73372	93459										
[L].LNPTHQIYPVTQ.[P]_	P05814						366443	317963	142922	244774	213454	101425	88465		134031	163039		12425			44242
[L].LNPTHQIYPVTQPL.[A]_	P05814															54550					
[L].LNPTHQIYPVTQPLAPVH.[N]_	P05814					23001					36111	35497			48890	204287					38196
[L].LNQELLLNPTHQIYPVT.[Q]_	P05814											39225	21500	39823	205171	373236					324329
[L].LNQELLLNPTHQIYPVTQ.[P]_	P05814							95279		112405	128871										
[L].LNQELLLNPTHQIYPVTQP.[L]_	P05814															84588					
[L].LNQELLLNPTHQIYPVTQPLA.[P]_	P05814														17031	41524					
[L].LNQELLLNPTHQIYPVTQPLAP.[V]_	P05814						56828	53869	96791	79856	124255				158397	149457					
[L].LNQELLLNPTHQIYPVTQPLAPVHN.[P]_	P05814											46626	51408	63483	46556	31747					
[L].LQPLMQQVPQPIPQTLALPPQP.[L]_	P05814						175073	140369	102051	104633	164649										

Quantified modified peptide sequence	Protein ID	A_-80oC	A_-20oC_120h	A_4oC_6h	A_RT_6h	A_RT_24h	B_-80oC	B_-20oC_120h	B_4oC_6h	B_RT_6h	B_RT_24h	C_-80oC	C_-20oC_120h	C_4oC_6h	C_RT_6h	C_RT_24h	D_-80oC	D_-20oC_120h	D_4oC_6h	D_RT_6h	D_RT_24h
[L].LQPLMQQVPQPIPQTLALPPQPLWSVPQP.[K]_	P05814						831133	730917	663229	575161	807725	959705	827064	957557	804059	839297					
[L].LQPLMQQVPQPIPQTLALPPQPLWSVPQPK.[V]_	P05814						1085970	894276	674091	757799	2592679										
[L].LSPKHKYKSGIKSTETQPSSTT.[P]_	Q9P2B7											398526	354303	388219	348428	255736					
[L].LTQQTELQSLR.[R]_	O75888																53012	139518	46080		
[L].MPLPDGNGLEDGNVRH.[L]_	Q9ULZ1																15431	18774	15490		
[L].MSSAYLSTKDQYPYLK.[S]_	Q99541											87466	69026	91927	85869	42615					
[L].NAPSDWDSRGK.[D]_	P10451						82341	77872	56586	110244	122291	328031	349321	307513	396458	213960					
[L].NAPSDWDSRGKDS.[Y]_	P10451											365055	368903	381014	357388	210491					
[L].NEYNQLQLQAAHAQE.[Q]_	P47710									30804	55375										
[L].NGHEFDFIHDVNK.[K]_	Q08431					39011				24148	56480										
[L].NGHEFDFIHDVNKK.[H]_	Q08431					44614				52657	68013										
[L].NPDPSQKQNLL.[A]_	P10451											44574	69397	41774	40820	23069					
[L].NPTHQIYPVT.[Q]_	P05814						768915	814577	458109	407914	366714										
[L].NPTHQIYPVTQP.[L]_	P05814											103698	122012	89292	96613	241371					
[L].NPTHQIYPVTQPL.[A]_	P05814						460545	428764	240586	169894	134288	38175	37869	40393	24023	106974					
[L].NPTHQIYPVTQPLAPV.[H]_	P05814											268640	274231	328604	250462	175152	16172	17923	15930		31816
[L].NQELLLNPTH.[Q]_	P05814						350428	270169	109503	140882	109227	160833	182714	123496	293356	251637					
[L].NQELLLNPTHQ.[I]_	P05814						149802	122538	51427	62129	51996	57739	60785	55151	63117	56730					
[L].NQELLLNPTHQIYPVT.[Q]_	P05814				615565	809545	784545	713421		731173	957212				2270786	6286397		12807		341346	3060239
[L].NQELLLNPTHQIYPVTQPL.[A]_	P05814											33975			76138	742345		12402			74020
[L].NQELLLNPTHQIYPVTQPLAP.[V]_	P05814						609187	572955	235265	430828	333635	627335	557829	657881	717046	679385					
[L].NQELLLNPTHQIYPVTQPLAPV.[H]_	P05814						373020	399542		306054	102844										
[L].NQPSTQIVANAKGAVTG.[A]_	Q99541												99305								
[L].NSGKKSLETEHKA.[L]_	P04843						34947	29460		43137	111146										
[L].PIPQQVVPYPQRA.[V]_	P05814											41309	87684	96284	298781	344412		19238	21618	39964	61250
[L].PIPQQVVPYPQRAVP.[V]_	P05814											21576	24006	20297	44014	79416					
[L].PIPQQVVPYPQRAVPVQALL.[L]_	P05814											53689	48618	54812	77399	168478					
[L].PLAQPAVVLPVPQPEI.[M]_	P05814						120139	101560	161193	86551				34158	76617	292968	19203				88216
[L].PLAQPAVVLPVPQPEIMEVPKA.[K]_	P05814						60763	61920	124171		145625										
[L].PLAQPAVVLPVPQPEIMEVPKAK.[D]_	P05814		28675			53370	95296	43834	643628	125297	112325										
[L].PLAQPAVVLPVPQPEIMEVPKAKDTVYTK.[G]_1xOxidation [M17]	P05814						79085	56429	408942	86518	31484										
[L].PLPLLQPLMQQVPQPIPQT.[L]_	P05814						60595	64178		103160	44509	175804	191709	220409	202314	572407					
[L].PLPLLQPLMQQVPQPIPQTL.[A]_	P05814											312713	259332	333515	661975	827049		16295			157701
[L].PLPLLQPLMQQVPQPIPQTLAL.[P]_	P05814												26074		53789	123157	19596			42708	42933
[L].PLPLLQPLMQQVPQPIPQTLALPPQPLWSVPQP.[K]_	P05814											247441	263221	291317	222867	371901					
[L].PLRYPE.[R]_	P47710						1248619	1155335	1234617	920010	584484	49209	45005	27531	48529	31836					
[L].PLRYPER.[L]_	P47710						104419	177180	167218	230332	153799										
[L].PLRYPERL.[Q]_	P47710						756216	703741	217279	925997	747161										
[L].PLRYPERLQNPSE.[S]_	P47710					65829	941797	691922	555498	912288	719524	19437	35305		24644	123599					
[L].PLTEEELEKEAKKVEGFDLV.[Q]_	Q99541											86172	118266	74681	77058	56630					
[L].PNSHPPTVV.[R]_	P07498						50448	42754		24910		22741	35418	35108	50075	20921					
[L].PPQPLWSVPQPKV.[L]_	P05814					43406	117468	105534	96621	69817	138526	86785	128340	142384	486554	972749	12186	22507	34129	59639	180672
[L].PPQPLWSVPQPKVL.[P]_	P05814																27038	29458	53910	91194	98383
[L].PPQPLWSVPQPKVLPIPQQ.[V]_	P05814															157244					
[L].PPQPLWSVPQPKVLPIPQQV.[V]_	P05814											53773	60874		79529	159565					
[L].PPQPLWSVPQPKVLPIPQQVVPYPQR.[A]_	P05814					94470	127592	62142		94827	108801	447611	450929	361321	353218	310965					
[L].PPQPLWSVPQPKVLPIPQQVVPYPQRA.[V]_	P05814															224286					
[L].PPQPLWSVPQPKVLPIPQQVVPYPQRAVPVQ.[A]_	P05814				80860	30265	752375		343347	409925					22473	939958		12798	24423		194985
[L].PPQPLWSVPQPKVLPIPQQVVPYPQRAVPVQA.[L]_	P05814											64526				621114					
[L].PQNILPLAQPAVVLPVPQPEIMEVPK.[A]_	P05814			43541		129686	181516	177984	1340446	303617	237597	76899	90466	78131	94966	37191					
[L].PQNILPLAQPAVVLPVPQPEIMEVPKAKDTVYTK.[G]_	P05814					311854	278720	306058	3081390	677831	285254	126315	47484	39765	38848	120862					
[L].PSKETIEQEKQAGES.[-]_	P62328								88320												
[L].PTKETIEQEKRSEIS.[-]_	P63313						291738	275860	310099	78583	25202	554034	425127	408350	566482	450334	26946	28339	27694		
[L].PTRVTAIHIDPATH.[R]_	P49327							35413				39350	26963		22645						
[L].PVPQPEIMEVPK.[A]_	P05814		26668			109755	290377	134891	1388277	289841	304489	24528	25995	21422	21997	27033					
[L].PVPQPEIMEVPKA.[K]_	P05814						111770	99340	123552		149407										
[L].PVPQPEIMEVPKAK.[D]_	P05814									64840											
[L].PVPQPEIMEVPKAKDT.[V]_	P05814																18143	18974	16188		29669
[L].PVPQPEIMEVPKAKDTVYT.[K]_1xOxidation [M8]	P05814						103580	102680	588520	85947	58375										
[L].PVPQPEIMEVPKAKDTVYTK.[G]_1xOxidation [M8]	P05814						35886		856177	38542	111193										
[L].PVPQPEIMEVPKAKDTVYTKGR.[V]_	P05814								686780												
[L].QAAHAQEQIR.[R]_	P47710		46184			136126	152111	132577	131750	201540	302147										
[L].QFSLGSKINVKVGGNSKGTLK.[V]_	P0C0L4											69701	90389	62281	58111	45695					
[L].QLQAAHAQEQI.[R]_	P47710							31975		45889	54556										
[L].QNPSESSEPIPLESREEY.[M]_	P47710									26191											
[L].QNPSESSEPIPLESREEYMNGMNR.[Q]_	P47710						771240	910313	560959	461052	269465	1258991	1223021	1233131	923915	400622	50262	38659	39491		
[L].QNPSESSEPIPLESREEYMNGMNR.[Q]_1xOxidation [M]	P47710						67179	76875	81365	20025											
[L].QPLMQQVPQPIP.[Q]_	P05814						191094	204250	112982	165919	305000	97023	175319	126688	136656	165074					
[L].QPLMQQVPQPIPQTLALPPQP.[L]_	P05814											1302409	1061883	1282659	1173879	650914					
[L].QPLMQQVPQPIPQTLALPPQPLWSVPQP.[K]_	P05814											1566439	1788114	1902652	1173770	792031					
[L].QRNEQEQPLGQWHLS.[K]_	O00391																	51523	12535		
[L].REKQTDEIKDTR.[N]_	P47710					117420	55906	71863	164021	199057	221101					42435					
[L].RPAVSSSDQQSLIRKL.[Q]_	Q8IZA0											26311	27959	33274				15978			
[L].RPVAAEVYGTER.[Q]_	P02788					35699	20369	23652		35719	65296										

Quantified modified peptide sequence	Protein ID	A_-80oC	A_-20oC_120h	A_4oC_6h	A_RT_6h	A_RT_24h	B_-80oC	B_-20oC_120h	B_4oC_6h	B_RT_6h	B_RT_24h	C_-80oC	C_-20oC_120h	C_4oC_6h	C_RT_6h	C_RT_24h	D_-80oC	D_-20oC_120h	D_4oC_6h	D_RT_6h	D_RT_24h
[L].RPVAAEVYGTERQPR.[T]_	P02788		29243			50832	131030	139090	243160	327942	308707										
[L].RYPERLQNP.[S]_	P47710						17996	22398		47783	37083										
[L].RYPERLQNPS.[E]_	P47710						127670	160305	59251	156744	73805										
[L].RYPERLQNPSE.[S]_	P47710						410987	366240	86632	518853	470685	80628	53628	48688	100130	120429					
[L].RYPERLQNPSESSEPIPLESREEYMNGMN.[R]_	P47710						554248	490812	457296	538551	782865										
[L].SDPGPHPEPGEGEPFFPKGQ.[-]_	P19634											37868	49901	32298	32033						
[L].SEAVDATRGAVQSGVDKTKSVVTGGVQ.[S]_	O60664											141837	147181	146412	86886	59029					
[L].SNIQGVPQNIQDQAKHMGVMA.[G]_	Q99541											126617	123744	146888	109787	48757					
[L].SQDRLDAPPPPAAPLPRWSGPIGVS.[W]_	Q99523																18736	27220	30159		
[L].SSSEESITEYKQ.[K]_	P05814						198664	247192	164715	139027	84316	267797	236219	235403	208080	117247					
[L].SSSEESITEYKQKV.[E]_	P05814						24899	100532		86915		432678	413500	508748	836641	661939		28524			130883
[L].SSSEESITEYKQKVEKV.[K]_	P05814																				56158
[L].SSSEESITEYKQKVEKVK.[H]_	P05814											73304	83198	75173	39450						
[L].STKLHSRAYQ.[Q]_	Q99541											63743	83178	32121	41039	20306					
[L].STKLHSRAYQQALS.[R]_	Q99541											168429	221168	189311	166348	90051					
[L].STPAPEARPVIGALGL.[-]_	Q96AD5											38320	57228		40753	21035					
[L].TDLENLH.[L]_	P05814											21017	26586	39688	38855	32875					
[L].TDLENLHL.[P]_	P05814						56155	54193		66164	46112	47257	48927	34983	86841	78179					
[L].TDLENLHLP.[L]_	P05814											55661	33553	35948	58289	53219					
[L].TDLENLHLPLPLLQ.[P]_	P05814						72322	38810		57848	49225	12257	18921	13301	38233	19564					
[L].TDLENLHLPLPLLQP.[L]_	P05814			45852		51780	94910	112194		104081	207646	87818	102251	95027	80260	75561					
[L].TDLENLHLPLPLLQPLM.[Q]_	P05814			47159		33982	204837	181742		162684	210393	471248	418805	542535	372801	389227					189581
[L].TDLENLHLPLPLLQPLMQ.[Q]_	P05814						148012	161719		84044	123243	389006	303426	393898	394206	435889				46553	82540
[L].TDLENLHLPLPLLQPLMQQ.[V]_	P05814											35762		43501	18605	51294					
[L].TDLENLHLPLPLLQPLMQQVPQPIPQT.[L]_1xOxidation [M17]	P05814						67773	62698	722236	43188		28229	31373	39684	26975	30750					
[L].TDLENLHLPLPLLQPLMQQVPQPIPQTLA.[L]_	P05814											130273	112302	102575	102964	72927					
[L].TDLENLHLPLPLLQPLMQQVPQPIPQTLAL.[P]_	P05814											187527	97603	212780	161381	154295					
[L].TPSQIGVILR.[D]_	P62277											35515	52511	44334	31166	17739					
[L].TQQTELQSLR.[R]_	O75888																42472	103489	57825	57234	
[L].VPTEIALAQHRANA.[K]_	P19835						29066	42424		25192	34625										
[L].VQDGIAKGRVA.[L]_	Q13410											62128	63247	81663	71621	25587	156982	230583	173476	87397	48336
[L].VQDGIAKGRVAL.[R]_	Q13410																14524	23910	15346		
[L].VQDGIAKGRVALRIR.[G]_	Q13410																	16746	15178		26821
[L].VSSGVENALTK.[S]_	Q99541											38927	20337	34540	25300						
[L].VSSGVENALTKSELL.[V]_	Q99541												41470	37827	22302	14647					
[L].WSVPQPK.[V]_	P05814						42830	53001	65769	119499	161372										
[L].WSVPQPKVLPIPQQVVPYPQRAVP.[V]_	P05814																85433	91557	81285		52464
[L].YEDSKSVRLE.[D]_	Q13410											144009	214106	21892	152715	101491					
[L].YNKYPDAVATWLNPDPSQK.[Q]_	P10451										259700	67045		73817	94563	118414					
[M].AENGVKTITS.[V]_	Q99541											25499	42417	28908	31066	18355					
[M].AENGVKTITSVAMTS.[A]_	Q99541											184494	211363	165231	131902	68058					
[M].ALLTQQTELQSLR.[R]_	O75888																16962	50816			
[M].AVVLNGGTIPTAPPSHTGRHL.[W]_	Q9NZH0												39266	33238							
[M].DETMKELK.[A]_	P02649						32212	26423		51300	13633										
[M].DPAEEDTNVYTEKHSDSL.[F]_	P34741						73252	133078	95855	75837	60284										
[M].DPSKPSSNVAGVVIIVILL.[I]_	P22897								141506												
[M].ESEELNGAYK.[A]_	P10451											120933	132233	105738	91861	22298					
[M].ETVAEKALQ.[E]_	P10909									26592	18615										
[M].ETVAEKALQEY.[R]_	P10909						54961	62547		54735	64105										
[M].ETVAEKALQEYR.[K]_	P10909						51412	54470		74473	54439	44926	40465	47026	20859						
[M].EVPKAKDTVYTKG.[R]_	P05814						71299	84145	96487	60178	20675										
[M].GEDSAPRDADTLHSK.[L]_	Q13410											70029	93817	68610	59022	32404					
[M].GEDSAPRDADTLHSKLIP.[T]_	Q13410											35972	65465	65597	36220	27470					
[M].GIGLVKGGVSAVAGGVTAVGSAVVN.[K]_	Q8WUH6											31933	32742	47831	34311	24590					
[M].GTNRGASQAGMTGYGMPRQIL.[-]_	P37802											198406	221425	103131	146679	33398	28634	35711	43074		
[M].KPVPDLVPGN.[F]_	P02671						712843	747185	323182	847823	187548	80558	79599	85570	235396	111416					
[M].KPVQKVLEDSDLK.[K]_	P11021						173003	122508		319295	125572										
[M].MQLVSSGVENALTK.[S]_	Q99541											48348	56844	60583	41776	27080					
[M].NENSHVQVPFQQ.[L]_	P47710							28406		36908	47013										
[M].NENSHVQVPFQQL.[N]_	P47710						138914	97579	46863	63156	90778		61057	89327	87644	93564					
[M].NENSHVQVPFQQLNQLA.[A]_	P47710											145669	220663	189488	131742	71083					
[M].NENSHVQVPFQQLNQLAAYPYA.[V]_	P47710						85228	96447		91232	93522										
[M].PGPTPSGTNVGSSGRSPSKA.[V]_	P60468											38225	52322	52883	34865	26624					
[M].PGPTPSGTNVGSSGRSPSKAVA.[A]_	P60468											371832	522406	426184	453196	377431	223945	234284	251813	197638	155103
[M].PGPTPSGTNVGSSGRSPSKAVAA.[R]_	P60468						105720	145844	217914	91457	57898	262542	271750	213804	308685	204996	102225	132305	142285	117896	126138
[M].PGVTVKDVNQQ.[E]_	P39019											19697			24771						
[M].PGVTVKDVNQQEFVRA.[L]_	P39019									24700	16553	42258	61666	52884	35245	40216					
[M].PVLKSPT.[I]_	P05814						167632	196075		157879	172313										
[M].PVLKSPTIP.[F]_	P05814						82484	85879	80556	88109	158742	102288	156495	144671	192492	221217					
[M].PVLKSPTIPFFDPQIPKL.[T]_	P05814						152147			84151		156280	110991	125535	250838	446115					
[M].PVLKSPTIPFFDPQIPKLTD.[L]_	P05814						174323	160123	132481	113415	153907	137458	128739	118420	225751	462954					
[M].PVLKSPTIPFFDPQIPKLTDL.[E]_	P05814														47947	56235					
[M].PVLKSPTIPFFDPQIPKLTDLEN.[L]_	P05814						93913		141872	64712											
[M].QLVSSGVENALTK.[S]_	Q99541											100100	78462	145361	74367	24695					

Quantified modified peptide sequence	Protein ID	A_-80oC	A_-20oC_120h	A_4oC_6h	A_RT_6h	A_RT_24h	B_-80oC	B_-20oC_120h	B_4oC_6h	B_RT_6h	B_RT_24h	C_-80oC	C_-20oC_120h	C_4oC_6h	C_RT_6h	C_RT_24h	D_-80oC	D_-20oC_120h	D_4oC_6h	D_RT_6h	D_RT_24h
[M].QQVPQPIPQTLALPPQPLWSVPQPK.[V]_	P05814						1141296	1069237	835166	1200617	2051974										
[M].QSKGGYGGGMPANVQMQLVDTKAG.[-]_	Q9NRR3											79198	83337	80364	51575	65948					
[M].SEYPTYHTHGRYVPPSS.[T]_	P15941											125098	175786	163530	99480	73907					
[M].SSAYLSTKDQYPYL.[K]_	Q99541											76212	122776	56108	48880	43512					
[M].TSALPIIQK.[L]_	Q99541											40274	37239	32008	31760	14358					
[M].TSALPIIQKLEPQIA.[V]_	Q99541											153245	140706	189237	147356	67569					
[N].APSDWDSRGK.[D]_	P10451											178144	190962	184667	175201	86739					
[N].APSDWDSRGKDS.[Y]_	P10451											195868	187862	213426	191599	112410					
[N].APSDWDSRGKDSYETSQL.[D]_	P10451											27351	44508	42900	54534	51325					
[N].DESNEHSDVIDSQELSK.[V]_	P10451						93827	136430	81523	74323	21661	216854	213830	214509	181492	60120					
[N].DESNEHSDVIDSQELSKVS.[R]_	P10451											612720	596011	658148	520317	301748					
[N].DESNEHSDVIDSQELSKVSREF.[H]_	P10451											142577	140169	116093	104071	101513					
[N].DIYGGEMHVRPML.[S]_	Q16625											27761		71686							
[N].DPEKVYGSGEKV.[A]_	Q9H3M7											174287	173821	188631	225939	82553					
[N].ENSHVQVPFQQL.[N]_	P47710											13595	29163	26242	32698	43007					
[N].EQEQPLGQWHLS.[K]_	O00391											27619	32437	27047	22201	17424		45553		26104	
[N].EYNQLQLQAAHAQEQ.[I]_	P47710				227405	133360		22610		81748	219890				43360	51259					
[N].GFKSHALQLNNRQ.[I]_	P0C0L4											29220		18875							
[N].GFKSHALQLNNRQI.[R]_	P0C0L4		33279	39927		30853						72669	95403	105132	120421	131737					
[N].GFKSHALQLNNRQIR.[G]_	P0C0L4											229294	315557	227297	289117	248715					
[N].GQLQQPTTGAASGGYIK.[R]_	O00161																	17412	16923		
[N].HPVRFGSDDEGRYLT.[Q]_	P12272									35572	67637										
[N].HPVRFGSDDEGRYLTQETN.[K]_	P12272									36190	85525										
[N].ILPLAQPAVVLPVPQPEIMEVPKA.[K]_	P05814						283454	457265	353888		38470										
[N].ILREKQTDEIKDT.[R]_	P47710							38417		73675	160982										
[N].KYPDAVATWLNPDPSQK.[Q]_	P10451											38257	39760	33467	33474	41996					
[N].LPGVDPNNEAIRN.[A]_	P55036						46082	34454				26535	20711	25332	30013						
[N].LTKDDLDEEEDTHL.[-]_	O00592						88834	91169	242308	31260	13639										
[N].NPYVPRTYYANPAVVRPHAQIP.[Q]_	P07498											22236	28147	31164	21647						
[N].PAVVRPHAQIPQRQ.[Y]_	P07498											24542	24638	26122		36001					
[N].PAVVRPHAQIPQRQYLPNSHPPTV.[V]_	P07498											115678	114104	90414	55911	70873					
[N].PAVVRPHAQIPQRQYLPNSHPPTVV.[R]_	P07498						273405	483831	627170	247635	178887	451902	441347	367360	239294	179471					
[N].PQPHPGWQGTL.[K]_	P19835						78264	71130	116571	75226	117277										
[N].PQPHPGWQGTLK.[A]_	P19835						319713	333354	159530	598002	475899	26382	35737	39296	83789	47653					
[N].PSESSEPIPLESREE.[Y]_	P47710											41889	32344	35672	34572	24321					
[N].PSESSEPIPLESREEYMNGMN.[R]_	P47710					61195	289951	295948	193195	354540	394400	654056	595781	667201	556497	390060					
[N].PSESSEPIPLESREEYMNGMNR.[Q]_	P47710					66204	242036	285407	254610	386983	354320	272309	238965	281156	316247	254578					
[N].PTAHENYEKNNVML.[Q]_	P47710			159739	133684	64886										34230	70950	90705	67828		128624
[N].PTHQIYPVT.[Q]_	P05814						223394	182289	95756	59481	33888	121566	156916	173357	208069	153260					
[N].PTHQIYPVTQ.[P]_	P05814						771051	734323	325913	415121	365331					34639	40016	50273	54417	87257	130581
[N].PTHQIYPVTQPLAP.[V]_	P05814																	39819			
[N].PTHQIYPVTQPLAPVH.[N]_	P05814						193246	149887	146787	127312	34417	33908		40275	84065	123124		12185			27125
[N].QELLLNPTH.[Q]_	P05814						137301	100457	79528	61829	51202	114967	115784	58527	93229	76545					
[N].QELLLNPTHQIYPV.[T]_	P05814															60221					
[N].QELLLNPTHQIYPVT.[Q]_	P05814																				458677
[N].QELLLNPTHQIYPVTQ.[P]_	P05814						1057458	752878	413252	602176	737369	184942	184876	203495	356610	569051	20889	23441	32320	67882	116017
[N].QELLLNPTHQIYPVTQP.[L]_	P05814											59475	61017	63913	57790	567925					
[N].QELLLNPTHQIYPVTQPL.[A]_	P05814											30915	27100	50774	73902	2084457		16972	22105	35057	49642
[N].QELLLNPTHQIYPVTQPLAP.[V]_	P05814											91461	79771	95075	97817	73216					
[N].QELLLNPTHQIYPVTQPLAPVH.[N]_	P05814											87095	103312	83030	134358	250785					50772
[N].QLQLQAAHAQEQIRR.[M]_	P47710						286987	227002	250731	742967	898844										
[N].SGYLEITKK.[M]_	P19835						31281	34080		41408											
[N].TVLGSRMM.[Q]_	Q99541											48035	64629								
[N].TVLGSRMMQL.[V]_	Q99541											24430	27680								
[P].AGAAPAPGLGAAAAAPGALVRDVHS.[L]_	Q8NES3																37792	61759	60656	30731	22826
[P].AIAINNPYVPRTYYANPAVVRP.[H]_	P07498											27389	46003	55288		170873					
[P].APGLGAAAAAPGALVRDV.[H]_	Q8NES3																44338	49844	59418	55895	31562
[P].AVLVHRDGREQEAEQMPEYR.[G]_	Q13410						255678	179362	332637	332927	97372	106278	98261	57442	50782			30602			
[P].AVLVHRDGREQEAEQMPEYRG.[R]_	Q13410							118186	108222	117651	78413										
[P].AVVLPVPQPEIMEVPKA.[K]_	P05814						149013	63406	96130	739071	419849	20450		26898	50223	55508					
[P].DATDEDITSH.[M]_	P10451											46400	51923	56740	59939	38318					
[P].DATDEDITSHM.[E]_	P10451						106716	133557	81232	108798	124698										
[P].DATDEDITSHMESEELNGAY.[K]_	P10451											45352	29537		18696	12567					
[P].DAVATWLNPDPSQK.[Q]_	P10451											491800	475141	485168	504427	281950					
[P].DAVATWLNPDPSQKQNL.[L]_	P10451											75133	79754	84104	83350	64752					
[P].DAVATWLNPDPSQKQNLLAPQ.[N]_	P10451											86882	75079	90101	89778						
[P].DEKVLDSGFREIEN.[K]_	P01833						27690	64222	60230	30565	79241										
[P].DGNGLEDGNVRH.[L]_	Q9ULZ1																12283	13773			10641
[P].DIQYPDATDEDITSHM.[E]_	P10451						62768	67463	82219	71495	59203	24292	44500	24631	15973	24952					
[P].DIQYPDATDEDITSHMESEELNGAYK.[A]_1xOxidation [M16]	P10451						122118	103766	173868	68111	42526										
[P].DTAHPHLF.[L]_	Q13410											29878	31253	49311	24706						
[P].DVSSALDKLKEFGNTLEDK.[A]_	P02654						47758				35445										
[P].EIMEVPKAKDTVYT.[K]_1xOxidation [M3]	P05814									29342	53772										
[P].EIMEVPKAKDTVYTK.[G]_1xOxidation [M3]	P05814					16841															

Quantified modified peptide sequence	Protein ID	A_-80oC	A_-20oC_120h	A_4oC_6h	A_RT_6h	A_RT_24h	B_-80oC	B_-20oC_120h	B_4oC_6h	B_RT_6h	B_RT_24h	C_-80oC	C_-20oC_120h	C_4oC_6h	C_RT_6h	C_RT_24h	D_-80oC	D_-20oC_120h	D_4oC_6h	D_RT_6h	D_RT_24h
[P].ENPSQPASPRVTKCKARSPVRLP.[H]_	O15018											69216	54799	38469	38615	63759					
[P].ERLQNPSE.[S]_	P47710						50912	44241		30889											
[P].ERLQNPSESSEPIPLE.[S]_	P47710											49103	45179								
[P].ERLQNPSESSEPIPLESREE.[Y]_	P47710											44211	33934	45914	29422	14238					
[P].ERLQNPSESSEPIPLESREEYMN.[G]_	P47710											64174	52731	50599	47721	30709					
[P].FAAPTKALENPQPHPGWQGTL.[K]_	P19835					40154	152138	150742	135941	150873	224579										
[P].FFDPQIPKL.[T]_	P05814						36220	37593			17154	30668			58695	100151					
[P].FFDPQIPKLTD.[L]_	P05814						52325	35405		19763		42896	49020	34631	46641	97295					
[P].FFDPQIPKLTDLENLHLPLP.[L]_	P05814		27885	35085		67445	102836	110750	107042	163799	283073	18351	18003	21786	23186	53515					
[P].FFDPQIPKLTDLENLHLPLPLLQPLM.[Q]_	P05814															40408					
[P].FFDPQIPKLTDLENLHLPLPLLQPLMQ.[Q]_	P05814											91509	58365	54378	62231	61227					
[P].FPPFSDISNPTAHENYEKNNVMLQW.[-]_1xOxidation [M22]	P47710			89026																	
[P].FVEPIPYGFLPQNILPLAQPAVVLPVPQPEIMEVPKAK.[D]_	P05814											15512	19225	21298	14471	81446					
[P].GLDGAQIPRDPSQQELPR.[L]_	P49327										26327					45436					
[P].GQEPPEHMAELQRNEQEQPLGQWHLS.[K]_	O00391						61358	35500	73086	62173	70102	45905	33348	35350	25740	33429					
[P].IIQKLEPQIA.[V]_	Q99541											23726									
[P].IPLESREE.[Y]_	P47710						97563	132674	100552	70648	36902	159033	211547	201771	226679	89269					
[P].IPLESREEYM.[N]_	P47710											35334	34872	32456	24231	11643					
[P].IPLESREEYMNGM.[N]_	P47710						54504	44195		46286	72111	72127	71872	82953	60043	46596					
[P].IPLESREEYMNGMN.[R]_1xOxidation [M13]	P47710						63427	74625	119909	55750	30049										
[P].IPLESREEYMNGMNR.[Q]_	P47710					28220	637921	773248	709758	835833	700758	817196	775726	785888	645086	443561					
[P].IPQQVVPYPQRAV.[P]_	P05814						114319	95245		54485	87864										
[P].IPQQVVPYPQRAVPVQALL.[L]_	P05814							292421	154094	161160	88167	955529	1143689	1121402	978195	665684					
[P].IPQQVVPYPQRAVPVQALLL.[N]_	P05814											78475	83758	75991	57900	74222					
[P].IPQQVVPYPQRAVPVQALLLN.[Q]_	P05814											60973	51771	64429	79038	45380					
[P].KLPLRYPE.[R]_	P47710						101299	102720	60810	70404	38285										
[P].KLPLRYPERLQ.[N]_	P47710						53429	48483		54992	28380										
[P].KLTDLENLHLP.[L]_	P05814						46271	37795	49493	23207	17178	109114	106934	51132	52992	25893					
[P].LAPVHNPISV.[-]_	P05814											135953	141504	136082	138717	225751	42914	75228	51759	70161	150439
[P].LAQPAVVLPVPQPEIMEVPKAKDTVYTKG.[R]_	P05814					51032															
[P].LESREEYMNGMN.[R]_1xOxidation [M11]	P47710						33712	36050	43747	20943	18056										
[P].LESREEYMNGMNR.[Q]_	P47710				173705	131440	1087410	1217126	1833179	2788938	935864	319876	346477	294253	453221	422764		28076			25438
[P].LESREEYMNGMNR.[Q]_1xOxidation [M]	P47710						27318	32039	72385	42960	5275										
[P].LESREEYMNGMNRQ.[R]_	P47710						80640	101228	95706	60471	27020										
[P].LIYPFVEPIPYGFLPQNILP.[L]_	P05814																18119	13043	35296	74301	27092
[P].LIYPFVEPIPYGFLPQNILPLAQPAVVLPVPQP.[E]_	P05814						79963	29685	43138	36750											
[P].LLQPLMQQVPQPIPQTLALPPQPLWSVPQPK.[V]_	P05814		303342	696557	354564	3512516	10951149	8838878	10468382	10255132	11105828			126403	327774	463242					
[P].LMQQVPQPIPQTLALPPQPLWSVPQPK.[V]_1xOxidation [M2]	P05814							46014	73896	57969	100288										
[P].LPVRGGNVGINS.[F]_	P49327											423794	511597	418775	238387	59181	343046	392175	319871	264803	89174
[P].LQPVTPLQLFEGRRN.[R]_	P0C0L4											170456	179632	160186	141105	124677					
[P].LRYPERL.[Q]_	P47710						74382	55839		74105	73928										
[P].LRYPERLQNPS.[E]_	P47710							33357		64771	42262										
[P].LRYPERLQNPSE.[S]_	P47710						63594	41601		33466	31004										
[P].LRYPERLQNPSESSEPIPLESREEYMNGMN.[R]_	P47710						221717	196766		253714	135370										
[P].LWSVPQPKVLPIPQQ.[V]_	P05814						79513		196266	43731	66300		55169			161751					
[P].LWSVPQPKVLPIPQQVVPYPQRAVPVQ.[A]_	P05814											19348	32130		59932	341011					
[P].LWSVPQPKVLPIPQQVVPYPQRAVPVQALLLNQE.[L]_	P05814					158883															
[P].MGEDSAPRDADTLHSK.[L]_	Q13410											33191		37699	32149						
[P].PPYRPDEFKPNH.[Y]_	Q16625											40764	39536	37379	28752	24326					
[P].PQPLWSVPQPK.[V]_	P05814						27609			38177	79176										
[P].PQPLWSVPQPKV.[L]_	P05814												135102			23039					
[P].PQSPWDRVKDLATVYVDVLK.[D]_	P02647											43521	26299		44545	44155					
[P].PSPPPPPPP.[P]_	P10323											34504	30157	50692	30230	18760					
[P].QIPKLTDLENLH.[L]_	P05814											52572	60301	59526	49384	39356					
[P].QIPKLTDLENLHLP.[L]_	P05814											72820	64842	55279	49992	69514					
[P].QIPKLTDLENLHLPLP.[L]_	P05814											73817	55214	59453	63802	46480					
[P].QIPKLTDLENLHLPLPLLQPLMQQVPQPIPQT.[L]_	P05814											38371	70163	76920	53506						
[P].QNILPLAQPAVVLPVPQPEIMEVPKAKDTVYT.[K]_	P05814			51403		209529	601193	739656	347742	825382	1323801	53966	47945	55323	94779	109117					
[P].QPIPQTLALPPQP.[L]_	P05814											343318	346677	368251	295384	164259					
[P].QPIPQTLALPPQPLWSVPQP.[K]_	P05814											170565	154399	180878	161231	135682					
[P].QPKVLPIPQQVVPYPQ.[R]_	P05814						75225	63883	79674	24293											
[P].QPLIYPFVEPIPYGFLP.[Q]_	P05814											229535	257515	257124	228357	87265					
[P].QPLIYPFVEPIPYGFLPQ.[N]_	P05814											152848	126954	177135	151798	79297					
[P].QPLIYPFVEPIPYGFLPQN.[I]_	P05814											27975		20209	23123	23587					
[P].QPLIYPFVEPIPYGFLPQNI.[L]_	P05814													52913		42159					
[P].QPLWSVPQPKVLPIPQQ.[V]_	P05814											12385		24586		54248					
[P].QPLWSVPQPKVLPIPQQVVPYPQR.[A]_	P05814											109499			95464	71491					
[P].QQVVPYPQRAVPVQA.[L]_	P05814												55628	63583							
[P].QTLALPPQPLWSVPQPKVLPIPQQVVPYPQR.[A]_	P05814			37086	47419	47550						23902		23014	24673	60979					
[P].RLFAEEKAVADTR.[D]_	P01833						19882	24852		14851	24176										
[P].SDWDSRGK.[D]_	P10451											30914	37644	36743	22900	19981					
[P].SESSEPIPLESREEYMNGMNR.[Q]_	P47710						144244	197009	199386	301011	348769	240946	226538	289543	293051	245450					
[P].SFQPQPLIYPFVEPIPYGFLP.[Q]_	P05814													26591	56960	69946					
[P].SNDIYGGEMHVRPML.[S]_	Q16625											41067	43052	42931	22018	33327					

Quantified modified peptide sequence	Protein ID	A_-80oC	A_-20oC_120h	A_4oC_6h	A_RT_6h	A_RT_24h	B_-80oC	B_-20oC_120h	B_4oC_6h	B_RT_6h	B_RT_24h	C_-80oC	C_-20oC_120h	C_4oC_6h	C_RT_6h	C_RT_24h	D_-80oC	D_-20oC_120h	D_4oC_6h	D_RT_6h	D_RT_24h
[P].TIPFFDPQIPK.[L]_	P05814						1364415	1270327	435403	1925876	2018348										
[P].TIPFFDPQIPKL.[T]_	P05814						147404	105513		36941	45967	46841		61288		134488					
[P].TIPFFDPQIPKLTD.[L]_	P05814						152640	132032		84495	92227			64738	175667	310493					
[P].TIPFFDPQIPKLTDLENL.[H]_	P05814															39632					
[P].TIPFFDPQIPKLTDLENLHLPLPL.[L]_	P05814											76455	41193	106071	38091	65630					
[P].TIPFFDPQIPKLTDLENLHLPLPLLQPL.[M]_	P05814											445758	375210	369742	287974	248824					
[P].TIPFFDPQIPKLTDLENLHLPLPLLQPLM.[Q]_1xOxidation [M29]	P05814											131740	145281	148948		58063					
[P].TIPFFDPQIPKLTDLENLHLPLPLLQPLMQ.[Q]_	P05814											1207990	958038	914338	784159	569161					
[P].TPSGTNVGSSGRSPSKAVA.[A]_	P60468											37308	56205	48540	54734	34124	26054	17111	14159		16111
[P].VAQDLNAPSDWDSR.[G]_	P10451						52692	71457		91903	74142										
[P].VAQDLNAPSDWDSRGK.[D]_	P10451						111810	97705	95960	66165	49274										
[P].VAQDLNAPSDWDSRGKDS.[Y]_	P10451											313035	302678	257191	189962	128159					
[P].VAQDLNAPSDWDSRGKDSYETS.[Q]_	P10451															22123					
[P].VKQADSGSSEEKQLYNKYPDAVATWLNPDPSQ.[K]_	P10451												55748	34630		27590					
[P].VLKSPTIPFFDPQIPKL.[T]_	P05814															66468					
[P].VLKSPTIPFFDPQIPKLTD.[L]_	P05814																26676	23754	36082		90433
[P].VLKSPTIPFFDPQIPKLTDL.[E]_	P05814														88077	1618190					
[P].VLKSPTIPFFDPQIPKLTDLEN.[L]_	P05814						172656	173886			102132										
[P].VLKSPTIPFFDPQIPKLTDLENLHLPLP.[L]_	P05814						50135	45796	155588	13822	80344	22496	93675	14979	24184	32194					
[P].VPDLVPGNFK.[S]_	P02671		101386	173537	240944	29972	118069	129037	115216	65498											
[P].VPQPEIMEVPKAKDT.[V]_1xOxidation [M7]	P05814						52547	48831	101283	36712	11858										
[P].VPQPEIMEVPKAKDTVYTK.[G]_	P05814		84320		190765	35856															
[P].VQALLLNQELLLNPTHQIYPVTQPLAPVH.[N]_	P05814											423901	393096	381330	443887	399369					
[P].VQALLLNQELLLNPTHQIYPVTQPLAPVHN.[P]_	P05814											72407	79645	57493	100183	129116					
[P].WVEQEGPEYWDRETQKY.[K]_	P30510																16770	15815	20612		16066
[P].YEKVSAGNGGSSL.[S]_	P15941																	29217	25433		
[P].YEKVSAGNGGSSLSY.[T]_	P15941							47343	274202		36679	80577	79389	47888	53961		45491	49668	29224		17833
[P].YGFLPQNILPLAQPAVVLPVPQPEIMEVPKA.[K]_	P05814						73278				39055	36057	46273	36342	20398	41413					
[P].YGFLPQNILPLAQPAVVLPVPQPEIMEVPKAK.[D]_	P05814						162845	140063	441589	33656	136244										
[P].YGFLPQNILPLAQPAVVLPVPQPEIMEVPKAKDTVYT.[K]_	P05814						269995	442960	341694	317807	586627										
[Q].AAHAQEQIRR.[M]_	P47710						385264	417857	444139	1144766	1379692	63721	90569	55397	106242	202136					
[Q].ADGSRASVDSGSSEEQGGSS.[R]_	P01833																26394	12205	14396		7640
[Q].ADGSRASVDSGSSEEQGGSSR.[A]_	P01833						74398	117466	130593	49581	8491	76307	94666	88109	57560	34505	130170	217393	161228	86973	78209
[Q].ADGSRASVDSGSSEEQGGSSRALVST.[L]_	P01833											217607	230240	217249	203296	100152	133972	148499	156512	121877	60169
[Q].ADGSRASVDSGSSEEQGGSSRALVSTLVPLG.[L]_	P01833																400100	364739	362854	346585	234253
[Q].ALLLNQELLLNPTH.[Q]_	P05814											124901	74185		69572	77560		17399			
[Q].ALLLNQELLLNPTHQIYPVT.[Q]_	P05814						131464	129221	132517	116754	132344	84254	82301	78926	535546	1596122		168086			938876
[Q].ALLLNQELLLNPTHQIYPVTQ.[P]_	P05814						90830	76323	99808	85564	111874	37504	30744	55472	174002	1006487				41761	231346
[Q].ALLLNQELLLNPTHQIYPVTQPLAPVH.[N]_	P05814											131040	136069	137478	199528	1622788	62618	71089	102843	443935	232088
[Q].DAYDGKDYIALNEDLR.[S]_	P13746; P30443																33267	27219	25316	34253	18642
[Q].DEPPQSPWDRVKDLATVYVDVL.[K]_	P02647											56392		55910		17432					
[Q].DEPPQSPWDRVKDLATVYVDVLK.[D]_	P02647		93162				133048	122693		95151	85471	434694	368164	399809	401951	293915					
[Q].DLNAPSDWDSR.[G]_	P10451						47954	74100	44996	154312	215629	31857	37157	43882	54180	27761					
[Q].DLNAPSDWDSRG.[K]_	P10451							30777	194999	22121	26831										
[Q].DLNAPSDWDSRGKD.[S]_	P10451						572217	460614	98096	462045	187376	73000	71326	74808	87949	70033					
[Q].DLNAPSDWDSRGKDSYETSQ.[L]_	P10451											43522	46116	46201	31800						
[Q].DPRLFAEE.[K]_	P01833						140252	124350		112222	61927										
[Q].DPRLFAEEKA.[V]_	P01833						27052			24788	28049										
[Q].DPRLFAEEKAVA.[D]_	P01833						30049	46751		69091	172344										
[Q].DPRLFAEEKAVADTRDQ.[A]_	P01833						328644	426986	354176	207298	125770	4272161	4174771	3792117	3332714	1984695	683353	763062	717827	450367	439503
[Q].EAEQMPEYR.[G]_	Q13410						62752	85247		40537	22192	41522	42082	35505	38608	13976	196670	273500	236625	178996	63337
[Q].EAEQMPEYRGR.[A]_	Q13410						109378	133595	120457	48229											
[Q].ELLLNPTH.[Q]_	P05814						140298	114882		40921	50362	47133	58774	55145	76169	87818					
[Q].ELLLNPTHQIYP.[V]_	P05814						258063	238469	125719	163764	253454										
[Q].ELLLNPTHQIYPVT.[Q]_	P05814						934473	844160	428886	1422360	2145273	144495	146257	242901	1651061	7798386					1436951
[Q].ELLLNPTHQIYPVTQP.[L]_	P05814											52906	58861	60135	92457	896616					
[Q].ELLLNPTHQIYPVTQPL.[A]_	P05814															585407					
[Q].ELLLNPTHQIYPVTQPLA.[P]_	P05814															61389					
[Q].ELLLNPTHQIYPVTQPLAP.[V]_	P05814											153054	153071	148369	200504	147909					
[Q].ELLLNPTHQIYPVTQPLAPVHN.[P]_	P05814											65067	49032	63504	43339	68723					
[Q].EMVSSAKDTVATQL.[S]_	O60664											37748	31586	32648	27514	17174					
[Q].EPPEHMAELQRNEQEQPLGQWHLS.[K]_	O00391						61898	63487				109809	117490	89983	81209	38713					
[Q].EQAEKEMY.[S]_1xOxidation [M7]	Q96JH7						256403	323578	143957	209661	75581	137756	136868	125759	105913	34934					
[Q].FSLGSKINVKVGGNSKGTLK.[V]_	P0C0L4											81054	113807	71314	73788	52609					
[Q].GEDEHQDKIYP.[S]_	P05814						1204346	1510079	3014728	1792774	1760484	139749	201040	118497	145566	179661	91094	137428	120764	65589	54235
[Q].GEDEHQDKIYPS.[F]_	P05814						89578	112614	108317	90349	62682	205987	219799	216073	216486	153293					
[Q].GEDEHQDKIYPSF.[Q]_	P05814						28785			22468	20218										
[Q].GEDEHQDKIYPSFQPQP.[L]_	P05814						46979	50716		39338	54880	29942	39683	29603	25292	25949					
[Q].GEDEHQDKIYPSFQPQPLI.[Y]_	P05814						95097	104367		103667	78055	32567		31596	53707	63760					
[Q].GGSSRALVSTLVPLG.[L]_	P01833											167591	209698	192037	140801	164450	163679	171401	151926	212099	123931
[Q].GPPPPPPGKPQ.[G]_	P02810																	75744			
[Q].GPPPQGGRPQGPP.[Q]_	P02810															10793					
[Q].GPPPQGGRPQGPPQGQSPQ.[-]_	P02810											71469	88277	78659	54416	32632		24216			
[Q].GPPQQGGHPPPP.[Q]_	P02810																	19017			

Quantified modified peptide sequence	Protein ID	A_-80oC	A_-20oC_120h	A_4oC_6h	A_RT_6h	A_RT_24h	B_-80oC	B_-20oC_120h	B_4oC_6h	B_RT_6h	B_RT_24h	C_-80oC	C_-20oC_120h	C_4oC_6h	C_RT_6h	C_RT_24h	D_-80oC	D_-20oC_120h	D_4oC_6h	D_RT_6h	D_RT_24h
[Q].GPPQQGGHPPPPQ.[G]_	P02810																	45684			
[Q].GPPQQGGHPPPPQG.[R]_	P02810											68570	108419	146084	116500	41358	27508	40205	31296	27534	18219
[Q].GVPQNIQDQAKHMGVM.[A]_	Q99541											29944	29295	32251		18891					
[Q].GVPQNIQDQAKHMGVMA.[G]_	Q99541											76976	77361	69935	60940	36260					
[Q].GVQVQVSTSNISSLEGARGLIA.[E]_	P49327										35334										
[Q].IPKLTDLE.[N]_	P05814												42162	35213	53631	97569					
[Q].IPKLTDLENL.[H]_	P05814						38961	29034	37161		504348	22491	26151	33300	50551	86559					
[Q].IPKLTDLENLH.[L]_	P05814						40709	32730		26074		337831	326806	364300	340334	275349					
[Q].IPKLTDLENLHLP.[L]_	P05814											74092	73934	60911	126150	55985					
[Q].IPKLTDLENLHLPLP.[L]_	P05814						226918				253623	348676	239610	322909	403776	545755					
[Q].IPKLTDLENLHLPLPLL.[Q]_	P05814											42071	62749	58758							
[Q].IPKLTDLENLHLPLPLLQP.[L]_	P05814						100462			74213	52176	233254	223700	227586	163109	106813					
[Q].IPKLTDLENLHLPLPLLQPLM.[Q]_	P05814							48244		45455		388266	359302	364948	351783	228146					
[Q].IPKLTDLENLHLPLPLLQPLMQ.[Q]_	P05814											287560	253847	263075	217902	164587					
[Q].IPKLTDLENLHLPLPLLQPLMQQVPQPIPQT.[L]_	P05814											82728	128520	83558	93663	74784					
[Q].IPQRQYLPNS.[H]_	P07498											78347	61749		37910	52951					
[Q].IYPVTQPLAPVHNPIS.[V]_	P05814											57269	72126	62252	43642	54409					
[Q].KDLELLLQAQQPSLQLSQTKS.[P]_	O15016											554516	586467	642744	964876	1400253					
[Q].KPSYYVRLG.[S]_	Q99541						199620	215121	695303	487606	137059	9172982	10288537	6961920	5860180	3772007					
[Q].KPSYYVRLGSLS.[T]_	Q99541											335314	404562	477608	212979	108036					
[Q].LDDQSAETHSHK.[Q]_	P10451											87187	114006	87908	100406	74359					
[Q].LDDQSAETHSHKQ.[S]_	P10451											39540	51123	41991	43223	32157					
[Q].LDDQSAETHSHKQS.[R]_	P10451											315136	301169	279626	213255	140577					
[Q].LDDQSAETHSHKQSRLY.[K]_	P10451		63299	42555		27169	89105	78135	163721	108690	40381	1811194	2124434	2035265	1641298	1086832					
[Q].LVGVSTPLQGGSNSAAAIGQSSGELR.[T]_	P15291																20866	21777			
[Q].LVSSGVENALTK.[S]_	Q99541											33145		49278							
[Q].MGTNRGASQAGMTGYGMPRQIL.[-]_	P37802											53397	43411		37047	35924	25605				
[Q].MPAVIRF.[-]_	P19835							75965			53778							14248			76525
[Q].NILPLAQPAVVLPVPQPEIMEVP.[K]_	P05814											195281	175772	193070	197524	87234					
[Q].NILPLAQPAVVLPVPQPEIMEVPK.[A]_	P05814						550877	540346	550549	368412	275167	959018	902845	1044705	1152676	927744					
[Q].NILPLAQPAVVLPVPQPEIMEVPKAKDTVYT.[K]_	P05814											147132	200327	169012	180885	135238					
[Q].NLLAPQNAVSSEETNDFK.[Q]_	P10451											34333	21525	28411		45714					
[Q].NPSESSEPIPLE.[S]_	P47710											223729	207341	216882	215547	122522					
[Q].NPSESSEPIPLESR.[E]_	P47710						164859	232038	80679	83051	79175	97355	101845	96496	82854	33652					
[Q].NPSESSEPIPLESREEYMNG.[M]_	P47710						60125	64773		37039		295556	270265	265625	246982	101713					
[Q].NPSESSEPIPLESREEYMNGM.[N]_	P47710						61787	53610	55107		46691	174815	182058	196779	143458	65971					
[Q].NPSESSEPIPLESREEYMNGMN.[R]_1xOxidation [M]	P47710						843020	987615	1358135	470608	164305	180608	313119	285528	182962	189811					
[Q].NPSESSEPIPLESREEYMNGMN.[R]_2xOxidation [M18; M21]	P47710						115309	169441	233945	57907	26706	77864	135488	112611	59648	53939					
[Q].NPSESSEPIPLESREEYMNGMNR.[Q]_1xOxidation [M]	P47710						468315	440657	784849	386168	46307	49830	105286	114002	95455	107294					
[Q].NPSESSEPIPLESREEYMNGMNR.[Q]_2xOxidation [M18; M21]	P47710												48400	60597							
[Q].PACHENDERPFYQK.[T]_	P07498					22511		19858		60896	71201										
[Q].PAVVLPVPQPEI.[M]_	P05814						136154	101931		70477	50938	33817	28984	31526	54255	34320					
[Q].PAVVLPVPQPEIMEVPK.[A]_	P05814											158720	163227	188488	180397	353400					
[Q].PAVVLPVPQPEIMEVPKA.[K]_	P05814						80489	100761		82906	98459					88455					
[Q].PAVVLPVPQPEIMEVPKAK.[D]_	P05814						28831	36832	345616	159932	137916										
[Q].PAVVLPVPQPEIMEVPKAKDTVYT.[K]_	P05814		44132	52628	298213	385098	977915	900366	3275747	950878	947398	437980	71090	479452	472686	323668					
[Q].PAVVLPVPQPEIMEVPKAKDTVYTKGR.[V]_	P05814																	26204			18143
[Q].PEIMEVPKAKDTVYTKGRVMP.[V]_	P05814												27957	13963	20232	29661		38051			9420
[Q].PKVLPIPQQVVPYPQR.[A]_	P05814											38053	36629	38344	23734	20040					
[Q].PLMQQVPQPIPQT.[L]_	P05814											45720	69995	73884	178168	521383					
[Q].PLMQQVPQPIPQTL.[A]_	P05814		29807		67859	77552	229471	194061	416998	185846	246579	86372	83201	102258	185485	301289	12857	11970	19622		100504
[Q].PPPPPYYPPEDKKTQ.[-]_	Q969T9						43469	40022	56792	24765		33193	34690	39770	38572	29466					
[Q].PSTQIVANAKGAVT.[G]_	Q99541											75727	54131		96361	48342					
[Q].QQGEDEHQDK.[I]_	P05814									36544											
[Q].QQGEDEHQDKIYP.[S]_	P05814						183956	281663	362148	295076	312875										
[Q].QQGEDEHQDKIYPSFQPQPLIYP.[F]_	P05814						119347	168185	111406	79135		327764	318839	319310	206971	83297					
[Q].QQGPPPPQGKPQ.[G]_	P02810																	45974			
[Q].QVPQPIPQTL.[A]_	P05814						184487	139073	366019	110622	131717										
[Q].QVPQPIPQTLALPPQPLWSVPQP.[K]_	P05814											1370630	1319475	1403344	1343893	1277452					
[Q].QVPQPIPQTLALPPQPLWSVPQPK.[V]_	P05814						213977	251226	162100	138267	241297										
[Q].QVVPYPQRAVPVQ.[A]_	P05814											56454	67513	70283	73663	634769		18181	30516		48689
[Q].QVVPYPQRAVPVQA.[L]_	P05814						82195	90943		34895	29973	108399	105328	127317	115348	239645	17794	19078	24874	45238	77865
[Q].QVVPYPQRAVPVQAL.[L]_	P05814															75785					
[Q].QVVPYPQRAVPVQALLLN.[Q]_	P05814											135200		142221		495225					
[Q].RAVPVQALLLN.[Q]_	P05814											75037	80254	52673	43386	34193					
[Q].RAVPVQALLLNQELLLNPTHQIYPVTQPLAPVHNPISV.[-]_	P05814											47671		37709	109970	200725					
[Q].RNEQEQPLGQWHLS.[K]_	O00391																19899	117747	31234		
[Q].RQYLPNSHPPTVV.[R]_	P07498											66954	75616	34379	44424	57529					
[Q].RQYLPNSHPPTVVR.[R]_	P07498						50416	70515		50960	35694	181707	230442	147856	126222	78182		34123	13987		
[Q].SAETHSHKQS.[R]_	P10451											95249	100056	68362	95337	39698					
[Q].SAETHSHKQSR.[L]_	P10451											18982	26017								
[Q].SAETHSHKQSRLY.[K]_	P10451											685026	825465	478223	612075	337723					
[Q].SGVALSPWVIQKNPLF.[W]_	P19835							30233			52951										
[Q].SGVDKTKSVVTGGVQ.[S]_	O60664											105650	117759	93103	84667	48684		23069			

Quantified modified peptide sequence	Protein ID	A_-80oC	A_-20oC_120h	A_4oC_6h	A_RT_6h	A_RT_24h	B_-80oC	B_-20oC_120h	B_4oC_6h	B_RT_6h	B_RT_24h	C_-80oC	C_-20oC_120h	C_4oC_6h	C_RT_6h	C_RT_24h	D_-80oC	D_-20oC_120h	D_4oC_6h	D_RT_6h	D_RT_24h
[Q].SGVDKTKSVVTGGVQSVMGSRLG.[Q]_	O60664											65861	76631	75131	51932	131244					
[Q].SPPRAALRPAVSSSDQQSLIR.[K]_	Q8IZA0											26588				33361					
[Q].TLALPPQPLWSVPQP.[K]_	P05814		21651	25901	45147	49813	40608	38671		86491	250410										
[Q].TLEGGVEKPHSLL.[S]_	P01133											15017		33476		11014					
[Q].VPQPIPQTLALPPQPLWSVPQP.[K]_	P05814											1235701	997000	1207104	1079009	833096					
[Q].VVPYPQRAVPVQ.[A]_	P05814						62780	53963	186955	46879	45722	112757	120864	146938	404733	738837					
[Q].VVPYPQRAVPVQA.[L]_	P05814						70265	70767		45396	49598	59843	84010	90244	128857	312037	26928	31628	22212	42578	172972
[Q].VVPYPQRAVPVQALLLN.[Q]_	P05814											21562	28142	17678		318100					
[Q].VVPYPQRAVPVQALLLNQE.[L]_	P05814											127655	191101	148996	118594	133718	187776	110702	191853	326768	291867
[Q].YLPLTEEELEKEAKKVEGFDLV.[Q]_	Q99541											219198	206091	175419	126408	112583					
[Q].YLPNSHPPTVVR.[R]_	P07498											45812	60866	102811	36186	34188					
[Q].YPDATDEDITSHM.[E]_	P10451						109916	137178	72665	63930	42640										
[Q].YPDATDEDITSHMESEELNGAY.[K]_	P10451											68480	57335	71897	29888	22529					
[Q].YVRTLPTNT.[L]_	P33121											27399	37867	31668	26571	6838	26494	30618	22912	22954	
[R].AAEDDEDDDVDTKK.[Q]_	P06454						368472														
[R].AAPGQEPPEHMAELQ.[R]_	O00391																27131	65902	33311	27830	
[R].AAPGQEPPEHMAELQRNEQEQPLGQWHLS.[K]_1xOxidation [M1	O00391																	9199		46341	47538
[R].ALQQNGAPGIAKVKAN.[-]_	Q9BW60						104022	77436													
[R].APWIEQEGPEYWDQETR.[N]_	P13746; P30443																43572	28954	30082		
[R].APWMEQEGSEYWDRETR.[S]_	P13747																118287	111043	84304	33309	14708
[R].APWMEQEGSEYWDRETRSA.[R]_	P13747																111383	118952	102387	50171	28943
[R].APWVEQEGPEYWDRETQNYK.[R]_	Q07000																77350	92192	73734	49854	17640
[R].ARSVDALDDLTPPSTAESGSRSPTSNGGR.[S]_	Q86X29						52038	44695		43025											
[R].ASVDSGSSEEQGGSSRA.[L]_	P01833																100109	55454	74853	39384	26459
[R].ASVDSGSSEEQGGSSRALVST.[L]_	P01833											95392	77816	46958	36535		190015	166714	198572	135834	33781
[R].ATLVQDGIAKG.[R]_	Q13410																42866	57157	46829		
[R].ATLVQDGIAKGRVA.[L]_	Q13410																50188	75953	45453		18958
[R].ATSEVSPNSKPSPNT.[K]_	P12272				195675	18490		31556	42824	91182	93029						21786	17463	15329	32415	28299
[R].AVPVQALLLNQE.[L]_	P05814											29848	26951	17170							
[R].AVPVQALLLNQELLLNPT.[H]_	P05814											40043	50259	50300	50291						
[R].AVPVQALLLNQELLLNPTH.[Q]_	P05814										167003				69016						
[R].AVPVQALLLNQELLLNPTHQ.[I]_	P05814						127332	73499		110650	196579	52697	44307	55719	59245	93775					
[R].AVPVQALLLNQELLLNPTHQIYPVTQPLAPV.[H]_	P05814						352810	295428	314764	270401	214642										
[R].AVPVQALLLNQELLLNPTHQIYPVTQPLAPVHN.[P]_	P05814											190976	169068		192703	89841					
[R].AVSEHQLLHDKGKSIQDL.[R]_	P12272						51646	40068	61431	121062	70943										
[R].AVSEHQLLHDKGKSIQDLR.[R]_	P12272						36961	24805		89968	57223							46634			
[R].DADDLQKRLAVYQAGAREGAE.[R]_	P02649						124821	120426	226243	261722	53968										
[R].DADTLHSKLIPT.[Q]_	Q13410						140109	162448	94539	92290	59328	658426	704793	782655	605266	368196					
[R].DADTLHSKLIPTQ.[P]_	Q13410											400871	458483	516437	339181	206157					
[R].DADTLHSKLIPTQPSQG.[A]_	Q13410						52067	52102		38318	26954	22924	32246	32954	25378	21568	22666	26490	19514		23827
[R].DDDKNNDGYIDYAEFAKSLQ.[-]_	Q8NI22						108247	128275	112923	87618	119430	150341	126176	129475	132737	31285					
[R].DGREQEAEQMPE.[Y]_	Q13410						48230	76931	67753	49158	16107	32941	31345	32432	33763		176441	183993	190851	99232	115417
[R].DGREQEAEQMPEYRG.[R]_1xOxidation [M10]	Q13410		22880	48880	72658	16786	74009	123109	174429	65120	20345		37383	19867		24334	19505	13153		78298	29752
[R].DGREQEAEQMPEYRGR.[A]_1xOxidation [M10]	Q13410																158961	169586	187400	684992	211956
[R].DGREQEAEQMPEYRGRAT.[L]_	Q13410						44202	34059	102712	24968	11365	71159	71329	65724	65011	64860	74612	41873	43909	81244	47802
[R].DMRQTVAVGVIKAVDK.[K]_	P68104						98734	82031	72837	57496	85883	108845	108485	115311	72766	69281					
[R].DQADGSRASVD.[S]_	P01833											27282	25180	25576	16940	11894					
[R].DRLDEVKEQVAE.[V]_	P02649						75724	125322	133358	96817	50315										
[R].DRLDEVKEQVAEV.[R]_	P02649						232940	252128	328559	186040	148533										
[R].DRLDEVKEQVAEVRA.[K]_	P02649						129030	121361	182432	110367	71087										
[R].DSILRSDEAVKPFGLK.[V]_	P49327						47424	61414	55317	88005	87129	28412		45348	34599	29303					
[R].DTSLFSDEFKVETSNK.[V]_	O14672						60550	44755	71506	173738	130718	26875		33534	42019	26746					
[R].DTYHPMSEYPTYH.[T]_	P15941						64829	71472													
[R].DVAVVAGGLGRQL.[L]_	Q15904													24492	44810						
[R].DVSLAKADAAPDEKVLDSGFR.[E]_	P01833											71157	67227	57598	47129		117177	134529	122150	72051	67111
[R].DYAGALIRPLT.[F]_	O60543											70876	88658	62544	65674	37320					
[R].EDGSYEEALVHLK.[V]_	Q13410						74026	66629	74084	91735	62985						23294	22776	24413	23781	16833
[R].EEAPSLRPAPPPISGGGYR.[A]_	P02675						95068	105533		79958	43029	31261	41563	38074	27830	25070	27116	37538	24790		16873
[R].EEYMNGMNR.[Q]_1xOxidation [M]	P47710						112275	93833	77985	106706	26925										
[R].EEYMNGMNRQ.[R]_	P47710						131812	125503		87097	73395					26756					
[R].EEYMNGMNRQR.[N]_	P47710						217693	127237		196183	16140										
[R].EEYMNGMNRQRNIL.[R]_	P47710						878312	861679	405264	1071453	872343										
[R].EGAERGLSAIRER.[L]_	P02649						48231	30769		143157	69185										
[R].EIENKAIQDPRLF.[A]_	P01833																123295	149231	126134	97848	56242
[R].EKQTDEIKDTRNESTQN.[C]_	P47710					29673		15406	31566	17767	17164										
[R].ELDESLQVAER.[L]_	P10909						28150	26601		23718	33598										
[R].ELDESLQVAERLT.[R]_	P10909											49052	45210	58182	51825	23377					
[R].ELDESLQVAERLTRK.[Y]_	P10909						212651	170197	205065	172303	172895	257456	222943	240815	169913	90543					
[R].ELDESLQVAERLTRKYNE.[L]_	P10909											88919	91601	90257	79130	60546					
[R].ELDESLQVAERLTRKYNELL.[K]_	P10909						392562	213294	236875	372655	522939	131418	51905	56348	179312	207491					
[R].ELDESLQVAERLTRKYNELLK.[S]_	P10909											29913	34722	32748	32809	64816					
[R].EPQDTYHYLPFSLPH.[R]_	P10909						60540	77956		43559	51415		23682	21333							
[R].EPQDTYHYLPFSLPHR.[R]_	P10909						31273	42726		36604	64627	30260	27485	30441	37896	23844					
[R].EQEAEQMPEYR.[G]_	Q13410						60958	80474		43413	12480	58086	62412	48215	42754	15232	314906	314185	218153	134502	37392

Quantified modified peptide sequence	Protein ID	A_-80oC	A_-20oC_120h	A_4oC_6h	A_RT_6h	A_RT_24h	B_-80oC	B_-20oC_120h	B_4oC_6h	B_RT_6h	B_RT_24h	C_-80oC	C_-20oC_120h	C_4oC_6h	C_RT_6h	C_RT_24h	D_-80oC	D_-20oC_120h	D_4oC_6h	D_RT_6h	D_RT_24h
[R].EQEAEQMPEYRGR.[A]_	Q13410																76412	94156	79181	36931	19827
[R].ERLGPLVEQGRVR.[A]_	P02649						65004	64014	53371	96977	57296										
[R].ETIESLSSSEESITE.[Y]_	P05814				44668	34280						46084	55354	54896	44169	35552	66281	48105	64611	32327	47754
[R].ETIESLSSSEESITEYKQKVEKVK.[H]_	P05814						54119		59450			137839	144606	126874	62244	74659					
[R].ETIESLSSSEESITEYKQKVEKVKHEDQQQG.[E]_	P05814						38472	21934		21333											
[R].EVSRLQGTGGPS.[Q]_	O75888						87637	98213	40903	47783	10373	103185	127318	136446	108380	29148	265230	338114	303454	337525	188954
[R].EVSRLQGTGGPSQNGEGYPW.[Q]_	O75888																21792	28314	22840		
[R].FPQATPRYDSKNGTLV.[-]_	Q96A22											117452	131728	132281	88348	49512					
[R].GAKTTFDVYTESWAQDPSQEN.[K]_	P19835				57249	228136	177872	149446	191741	445355	1011480										
[R].GLDKLEENLPIL.[Q]_	O60664													41549							
[R].GLEEELQFSLGSKIN.[V]_	P0C0L4										108118	117435	109946	125636	326105	378095					
[R].GLEEELQFSLGSKINVKVGGNS.[K]_	P0C0L4											55291	23833	35684	27095	56989					
[R].GLEEELQFSLGSKINVKVGGNSK.[G]_	P0C0L4											102153	117667	104617	101920	95509					
[R].GLEEELQFSLGSKINVKVGGNSKG.[T]_	P0C0L4											97977	114156	94201	61687	48212					
[R].GLEEELQFSLGSKINVKVGGNSKGT.[L]_	P0C0L4											106506	107333	89635	90715	78491					
[R].GLEEELQFSLGSKINVKVGGNSKGTL.[K]_	P0C0L4					198999						628510	689248	565892	496375	508148					
[R].GLEEELQFSLGSKINVKVGGNSKGTLK.[V]_	P0C0L4											122893	192782	104637	91889	109282					
[R].GPGTPAPTGLSAPLSFIPR.[H]_	Q6WN34		133044			84963	777789	758350	955754	645529	1238201										
[R].GPGTPAPTGLSAPLSFIPRHF.[R]_	Q6WN34					67338	193676	161680	156053	141616	195747										
[R].GPLVKYSSDYFQAPSDYR.[Y]_	Q08380					36043	34663	49792	49526	87769	283433										
[R].GRATLVQDGIA.[K]_	Q13410						62327			64469	31624						48306	59765	79573	36650	15559
[R].GRATLVQDGIAKG.[R]_	Q13410																118532	220095	175685		
[R].GRATLVQDGIAKGRVA.[L]_	Q13410											137537	172150	150132	119744	94003	104769	334182	149068	48079	158560
[R].GVEEEEEDGEMRE.[-]_1xOxidation [M11]	P62306						37774														
[R].GWDQEPAREQAGGGW.[R]_	Q86X29																20393	24024	29906	52828	56067
[R].GWDQEPAREQAGGGWR.[A]_	Q86X29																64197	94931	71298	67826	65268
[R].GYDQSAYDGKDYIALNEDLR.[S]_	P30510																45529	34223	37565	27955	15391
[R].HQGVMVGMGQKDSYVGDEAQSK.[R]_	P60709						148102	157938	153226	212884	266803	77465	72840	92444	86815	111932					
[R].HQGVMVGMGQKDSYVGDEAQSK.[R]_1xOxidation [M]	P60709							81550		92239	96444										
[R].HQGVMVGMGQKDSYVGDEAQSK.[R]_2xOxidation [M5; M8]	P60709											29745				13596					
[R].HRHPDEAAFFDTASTGK.[T]_	P02671						24549	20194		42067	38176										
[R].IEERLPIL.[N]_	Q99541											56721	46877	40365	30437	16295					
[R].KANDESNEHSDVIDSQELSK.[V]_	P10451							46890	81755	207198	182036	53958	91927	41266	120605	137218					
[R].KANDESNEHSDVIDSQELSKVS.[R]_	P10451											148099	142625	123794	112823	104024					
[R].KIHIDLPNE.[Q]_	P62333											27478			18713	29835					
[R].KMDPAEEDTNVYTEKHSDSLF.[K]_	P34741					26142	124817		198773	20357											
[R].KMDPSKPSSNVAG.[V]_	P22897																131141	170028	116738	54327	35047
[R].KPTLDKPSPETFVK.[S]_	Q53GQ0											59117	65937	58473	56294	30119					
[R].LDAPPPPAAPLPRWSGPIGVS.[W]_	Q99523																	102427			
[R].LDINTNTYTSQDLK.[S]_	Q9H173						44336	38721		61343	22459										
[R].LFAEEKAVADT.[R]_	P01833										48206										
[R].LFAEEKAVADTRD.[Q]_	P01833						32257	32196		28227											
[R].LFAEEKAVADTRDQ.[A]_	P01833																21232	17947	17876		
[R].LFAEEKAVADTRDQADGSRASVDSGSSEEQGGSSR.[A]_	P01833							30530		40078	19199										
[R].LLEDGEDFNLGDALDSSNSMQTIQK.[T]_	P05783						105302	118000		69551	186484										
[R].LNEYNQLQLQAAHAQEQI.[R]_	P47710						151331	119919	195872	324568	350425										
[R].LPEVEVPQHL.[-]_	Q02818						51178			123923	145691										
[R].LQNPSESSEPIPLESREEYMNG.[M]_	P47710											39358	51323	57305	56678	41077					
[R].LQNPSESSEPIPLESREEYMNGM.[N]_	P47710											44621	52834	54046	61557	34258					
[R].LQNPSESSEPIPLESREEYMNGMN.[R]_1xOxidation [M]	P47710						700149	820690	1172834	422279	417542										
[R].LQNPSESSEPIPLESREEYMNGMNRQRNIL.[R]_	P47710					77821															
[R].MNENSHVQVPFQQL.[N]_	P47710											55019	43369	32714	64387	69897					
[R].MNENSHVQVPFQQLN.[Q]_	P47710													15022	14921	103447					
[R].MNENSHVQVPFQQLNQ.[L]_	P47710								99407	248080	120726	81657	75292	81273	60526	35601					
[R].MNENSHVQVPFQQLNQL.[A]_	P47710														342328	59779					
[R].MPELVDFRQY.[V]_	P33121												23074	33337	23374	11596					
[R].NAASFKEVSDSLLTSSKGQLQK.[M]_	Q99541						75062	53018	97157	77035	128007	81190	49120	102587	33597	37885		21336			
[R].NEFSSKERLLEELKW.[K]_	Q13410							46790		42627											
[R].NEFSSKERLLEELKWK.[K]_	Q13410						37903			57636	72928										
[R].NEQEQPLGQWHLS.[K]_	O00391																38891	88983	44808	40193	11565
[R].NGFKSHALQL.[N]_	P0C0L4												65864	76138	71460						
[R].NGFKSHALQLNNR.[Q]_	P0C0L4		24101			38214						46947	78731	45791	25555						
[R].NGFKSHALQLNNRQIR.[G]_	P0C0L4											266544	407388	272369	333173	366944					
[R].NILREKQTDEIKD.[T]_	P47710						19703	30560		53053	34145				25658	44120					
[R].NNLKGKRLDINTNTYTSQDLK.[S]_	Q9H173										22256										
[R].NQSPVDQGATGASQGLLDRKEVLGGVIAGGLVG.[L]_	P18827											140725	186659	169465	97633	58177	141676	154171	123689	131076	83980
[R].NVNFQKAINEK.[L]_	P0C0L4					32627															
[R].NVNFQKAINEKLGQYASPTA.[K]_	P0C0L4											46945	60406	52550	50919	33699					
[R].NVNFQKAINEKLGQYASPTAK.[R]_	P0C0L4											32834	34022	23142	17444	13291					
[R].PAGDGTFQKWAAVVVPSGQEQR.[Y]_	Q95604																18124	24032	18173		
[R].PAIAINNPYVPR.[T]_	P07498						65471	87109	125296	49811	111272										
[R].PAIAINNPYVPRT.[Y]_	P07498											30499	35785	53390	33347						
[R].PAIAINNPYVPRTY.[Y]_	P07498											47458	66925	135160	48052	18781					
[R].PDIQYPDATDEDITSHMESEELNGAYK.[A]_	P10451						410084	290503	238604	78981	273012	1127338	1171795	1085213	943419	768601	32713				

Quantified modified peptide sequence	Protein ID	A_-80oC	A_-20oC_120h	A_4oC_6h	A_RT_6h	A_RT_24h	B_-80oC	B_-20oC_120h	B_4oC_6h	B_RT_6h	B_RT_24h	C_-80oC	C_-20oC_120h	C_4oC_6h	C_RT_6h	C_RT_24h	D_-80oC	D_-20oC_120h	D_4oC_6h	D_RT_6h	D_RT_24h
[R].PVAAEVYGTERQPR.[T]_	P02788									17246	32940										
[R].QGVQVQVSTSNISSLEGARGLIA.[E]_	P49327								617356		49364										
[R].QPYAVSELAGHQTSAESWGTGR.[A]_	P36578							63263			48781										
[R].QRNILREKQTDEIKDTR.[N]_	P47710					19593	24730	18038	197953	88811	35972	21878	59666		43319	35629					
[R].QWAGLVEKVQAAVGTSAAPVPSDNH.[-]_	P02649						37425	40094	85224	43632	22522										
[R].QYLPNSHPPTVV.[R]_	P07498						121680	107195	116128	95977	88254	45145	86878	70137	37625	36258					
[R].QYLPNSHPPTVVR.[R]_	P07498						82551	93692	59816	58141	60280	268834	347279	383723	152711	111624					
[R].RDFIDIESK.[F]_	P06858									36584											
[R].RFALEHEASDLVEIYLWK.[L]_	Q6WN34									41434	50744										
[R].RLERCIKELTYQAEEDKKNLSRMQTQMDK.[L]_	Q9Y2K3						86353	75239	120746		42931										
[R].RPAIAINNPYVPRT.[Y]_	P07498											117243	128743	144405	191013	185881					
[R].RPAIAINNPYVPRTYY.[A]_	P07498							24863			47472	50946	86393	68706	106220	150675					
[R].RPAIAINNPYVPRTYYANP.[A]_	P07498															101115					
[R].RPDIQYPD.[A]_	P10451						556598	756333	182161	332078	174028										
[R].RPDIQYPDA.[T]_	P10451						478913	414931	149305	207722	134275										
[R].RPDIQYPDATDE.[D]_	P10451						83480	80914	46841	65101	36524	28152	36144	29444	23917	17601					
[R].RPDIQYPDATDEDITSHM.[E]_	P10451						278031	331940	289575	216229	201594										
[R].RPDIQYPDATDEDITSHME.[S]_	P10451											43591	37402	78716	30709	30824					
[R].RPDIQYPDATDEDITSHMESEELNGA.[Y]_	P10451						129331	185661	265271	111115	107296	65048	52574	46233	28122	32214					
[R].RPDIQYPDATDEDITSHMESEELNGAYK.[A]_1xOxidation [M18]	P10451					55931	128691	157839	464432	116376	36777										
[R].RPNLHPSFIAIPPK.[K]_	P07498					27827	109210	109680	185121	122949	231610										
[R].RPSAAPASQQLQSLESKLTSVR.[F]_	Q9Y653											25221		23335	13644						
[R].RPSPAAPDGAPASGTSEPGRA.[R]_	O15232					17552															
[R].SAWLDSGVTGSGLEGDH.[L]_	P12272																149528	223608	156037	73473	14585
[R].SAWLDSGVTGSGLEGDHLS.[D]_	P12272																26011	48070	29366		
[R].SAWLDSGVTGSGLEGDHLSDTSTTSLELDSR.[R]_	P12272						92437	108474	186218	120201	76240	68402	59482	60776	60924		57413	42498	42523		34323
[R].SAWLDSGVTGSGLEGDHLSDTSTTSLELDSRRH.[-]_	P12272							52554	95697	52748											
[R].SDLYAVEMKKP.[-]_	Q9NQR4											33932	52984	25710	22841	14978					
[R].SEETKENEGFTVTAEGK.[G]_	P01024					20378		33079		53917	82595										
[R].SEETKENEGFTVTAEGKGQGTLSVVTMYHA.[K]_	P01024						64206	55690	85062	66045	89343										
[R].SGKDPNHFRPAGLPEKY.[-]_	P0DJI8		62708	73443		48604															
[R].SIGVVEEKVS.[Q]_	Q6UXA7																22316	36425	15587		
[R].SISDSDELASGFFVFPYPYPFRPLPPIPFP.[R]_	Q8NFU4																32980	19362	63786		74662
[R].SKSKKFRRPDIQYPDATD.[E]_	P10451						46322	24548		57419	27015										
[R].SLAGPAGAAPAPGLGAAAAAPGALVRD.[V]_	Q8NES3																36511	30363	35814	36019	25873
[R].SLAGPAGAAPAPGLGAAAAAPGALVRDV.[H]_	Q8NES3																19434	27365			25306
[R].SLYQSAGVAPESFEYIEAHGTGTKVGDPQELNGIT.[R]_	P49327						53806	58862	60535	46619	57628										
[R].SNDLARVPLK.[L]_	Q6UXA7																	18534	29136		35536
[R].SPGLMSEDSNLHY.[A]_	Q96A22											41022	69705	44569	43264	37944					
[R].SPRGWDQEPAREQAGGGWR.[A]_	Q86X29							65791										26230			
[R].SPYEKVSAGNGG.[S]_	P15941						29304	27171		11649	7628										
[R].SSEHENAYENVPEEEGKVRSTPM.[-]_	Q13113							58384	54793												
[R].SSKITHRIHWESASLLR.[S]_	P01024		168380	229475		111561	87923	101160		194322	153011										
[R].SWTAADMAAQITK.[R]_	P13746; P30443																15472	19856	22908		10237
[R].TAIRNGQVWEESLK.[R]_	P22079						48910	60972		98556	108081										
[R].TAIRNGQVWEESLKR.[L]_	P22079						26263	49029	56301	27191	12579										
[R].TALGLKENNQPEPLHLFLSSATPVHS.[K]_	Q9NV23						43548			47669											
[R].TEGDGVYTLNDKKQWIN.[K]_	P00738						21312	22330			138519										
[R].TEGDGVYTLNDKKQWINK.[A]_	P00738						83666	71579		132414	230821										
[R].TGSSKIFPGNWDNHSHK.[K]_	Q08431						45441	51770		39883	42574										
[R].TPEAVQKLLEQGLR.[H]_	P49327						98283	85370		52344	72477	52959		56763	57177	53239					
[R].TRTAIRNGQVWEESLK.[R]_	P22079						42350	61531	91322	132117	147154										
[R].TYYANPAVVRPH.[A]_	P07498						79827	95561	48169	12765								103056			
[R].TYYANPAVVRPHA.[Q]_	P07498											99006	100825	42054	65716	81505					
[R].TYYANPAVVRPHAQIPQRQY.[L]_	P07498											17670	28540	22225	31012	35220					
[R].TYYANPAVVRPHAQIPQRQYLPNS.[H]_	P07498						121521	109911	98649		106485	482036	621662	410688	407507	397381		29750		20095	15914
[R].TYYANPAVVRPHAQIPQRQYLPNSHPPTV.[V]_	P07498											123878	185510	153090	80366	107772					
[R].TYYANPAVVRPHAQIPQRQYLPNSHPPTVVR.[R]_	P07498														15135	39456					
[R].VETGVLKPGMVVTFAPVNVTTEVK.[S]_	P68104						50875					46502	74330		85392	61925					
[R].VMPVLK.[S]_	P05814						1820663	1663931	560720	4188071	1416066										
[R].VMPVLKSPT.[I]_	P05814						135183	130023		120639	91529										
[R].VMPVLKSPTIP.[F]_	P05814						372056	358908	129980	502226	704861	260297	406916	345444	463987	792555					
[R].VMPVLKSPTIPF.[F]_	P05814						209872	230836			231402										
[R].VMPVLKSPTIPFFDPQIP.[K]_1xOxidation [M2]	P05814						39007	184294	506063	39767	116141	57685		54220	51229	76691					
[R].VMPVLKSPTIPFFDPQIPKL.[T]_	P05814					84729				97976	196206										86692
[R].VMPVLKSPTIPFFDPQIPKLTD.[L]_	P05814		99440	191759	219928	322522	405402	422838	354150	509545	570492	98517	97289	141801	250804	1623908					265429
[R].VMPVLKSPTIPFFDPQIPKLTD.[L]_1xOxidation [M2]	P05814															63777					
[R].VMPVLKSPTIPFFDPQIPKLTDL.[E]_	P05814					163426							116125	101128	68572	220136					
[R].VMPVLKSPTIPFFDPQIPKLTDLENL.[H]_	P05814					133780	202989	204678		157652	156715				78683	190907					
[R].VMPVLKSPTIPFFDPQIPKLTDLENLHLPLP.[L]_1xOxidation [M2]	P05814						99622	89578	359073	104227	132947										
[R].VVNLPLVSSTYDLMSSAYLSTKDQYPYLK.[S]_	Q99541											95237	73653	94145	82810	80402					
[R].YEEIVKEVSTYIKK.[I]_	P68104													29479	19239			15923	16733		15943
[R].YPERLQ.[N]_	P47710						359554	356627	69512	336828	184910										
[R].YPERLQNP.[S]_	P47710						174692	193681	36256	147514	125207	68583	85828	52956	64733	42074					

Quantified modified peptide sequence	Protein ID	A_-80oC	A_-20oC_120h	A_4oC_6h	A_RT_6h	A_RT_24h	B_-80oC	B_-20oC_120h	B_4oC_6h	B_RT_6h	B_RT_24h	C_-80oC	C_-20oC_120h	C_4oC_6h	C_RT_6h	C_RT_24h	D_-80oC	D_-20oC_120h	D_4oC_6h	D_RT_6h	D_RT_24h
[R].YPERLQNPS.[E]_	P47710						623385	824032	232684	737195	612523										
[R].YPERLQNPSES.[S]_	P47710						47167	68758	65677	41064	30106										
[R].YPERLQNPSESS.[E]_	P47710						73682	93347		31822	22558	30684	45548	41772	25854						
[R].YPERLQNPSESSEPIPLE.[S]_	P47710						305534	292168	244988	220495	273431	604068	660170	665605	564733	353980	29007	27977	25913	22263	23261
[R].YPERLQNPSESSEPIPLESR.[E]_	P47710						85985	106610	96506			86731	66183	94713	71273						
[R].YPERLQNPSESSEPIPLESREE.[Y]_	P47710						60015	72118		68201	48488	172055	185944	176211	157262	63600					
[R].YPERLQNPSESSEPIPLESREEY.[M]_	P47710						50401	44579			31584	73996	62146	64299	50636						
[R].YPERLQNPSESSEPIPLESREEYMNG.[M]_	P47710											77995	55936	86489	68003	31225					
[R].YPERLQNPSESSEPIPLESREEYMNGM.[N]_	P47710											45902	29160	56756	29959						
[R].YPERLQNPSESSEPIPLESREEYMNGMN.[R]_1xOxidation [M]	P47710						522172	494686	973046	280426	180147	56558	127140	89761	41321	185361					
[R].YPERLQNPSESSEPIPLESREEYMNGMN.[R]_1xOxidation [M27]	P47710			76446																	
[R].YPERLQNPSESSEPIPLESREEYMNGMN.[R]_2xOxidation [M24; M	P47710						37894	77063	242097	20860	23792	35726	38231	48456	38277						
[R].YPERLQNPSESSEPIPLESREEYMNGMNR.[Q]_	P47710											194230	222394	183897	157603	177710					
[R].YVPPSSTDRSPYEKVSAGNGGSSLSYTNPAVAATSANL.[-]_	P15941						694503	665064	654875	328831	291159										
[RW].DGEDQTQDTELVETRPAGDGTFQK.[W]_	P13746; P30443; Q07000; P30510						86502														
[S].AETHSHKQSRLY.[K]_	P10451						83970	42859	114715	61833	83126	348073	432616	241265	284042	206891					
[S].AGTQDVPSPPSDYVERVDSPMAY.[S]_	Q16625											55305	36543	29895	35342						
[S].ALPIIQKLEPQIAVANT.[Y]_	Q99541											87711	91023	101735	69095	51859					
[S].ALPIIQKLEPQIAVANTY.[A]_	Q99541											1215885	1224948	1287342	1088712	742100					
[S].APLSFIPR.[H]_	Q6WN34						50659	42810	87221		51656										
[S].APRDADTLHSKLIPTQ.[P]_	Q13410											124351	93183	124500	76437	58849					
[S].APRDADTLHSKLIPTQPSQ.[G]_	Q13410		83537	70576																	
[S].AVPTHWEPYTTENSGYLEITKK.[M]_	P19835						43941	21949	72684	146943	46305										
[S].AYLSTKDQYPYLK.[S]_	Q99541											585480	645595	115576	374801	229974	71827	77965	54246	37296	20446
[S].DDSILRLAKADGIVSKNF.[-]_	P63220											51912	52450		50718	25758					
[S].DISNPTAHENYE.[K]_	P47710						41315	98052	77191	29126	25280										
[S].DISNPTAHENYEKNNVMLQW.[-]_1xOxidation [M17]	P47710						90750	87955	126043	73701										72107	69258
[S].DPITVTVPVEVSRK.[N]_	P10909											74592	58152	54665	34177	23061					
[S].DTSLTWNSVKG.[K]_	P02788						36954	47572		39867	73154										
[S].DVIDSQELSKVSREF.[H]_	P10451											53037	47402	49398	50818	42433					
[S].DWDSRGKDSYETSQ.[L]_	P10451											59567	70200	68089	79250	46809					
[S].DWDSRGKDSYETSQL.[D]_	P10451											50833	42161	79043	103687	108772					
[S].DWDSRGKDSYETSQLD.[D]_	P10451											86159	46899	79142	62033	30438					
[S].DWDSRGKDSYETSQLDDQSAETHS.[H]_	P10451											134964	138909	107538	115137	61091					
[S].DWDSRGKDSYETSQLDDQSAETHSH.[K]_	P10451											178097	200377	143793	147205	135908					
[S].DWDSRGKDSYETSQLDDQSAETHSHK.[Q]_	P10451													29013	43332	48085					
[S].DWDSRGKDSYETSQLDDQSAETHSHKQS.[R]_	P10451												60031		94159	63763					
[S].EAVDATRGAVQ.[S]_	O60664											28565	43561	28317	34356						
[S].EAVDATRGAVQS.[G]_	O60664											16657	23972	22925	21517						
[S].EAVDATRGAVQSGVDKTKSVVTG.[G]_	O60664											163173	184216	177202	133613	110508		40139	30044		
[S].EAVDATRGAVQSGVDKTKSVVTGGVQ.[S]_	O60664											80959	45110	91734	57188	57485		19004	16154		
[S].EEQGGSSRALV.[S]_	P01833											44463	55103	83859	73504	40868					
[S].EEQGGSSRALVSTLVPLG.[L]_	P01833											276496	296845	370951	564282	388260					
[S].EESITEYKQK.[V]_	P05814				103793	14676	59734	66557		69731	14631	81876	90545	102663	89370	63115					
[S].EESITEYKQKVE.[K]_	P05814						47338	43148		26507		71371	63293	80831	91839	56701					
[S].EFTITKGL.[R]_	P19835									43206	91572										
[S].EHQLLHDKGKSIQDL.[R]_	P12272															27641					
[S].ELEEQLTPVAEETR.[A]_	P02649						43742	42521		87727	117889										
[S].ELEEQLTPVAEETRA.[R]_	P02649						17802		39251	37134	74178										
[S].EPIPLESREE.[Y]_	P47710						247978	237630	151973	185013	166340	425493	453923	471541	486883	242425					
[S].EPIPLESREEYM.[N]_	P47710						336401	261197	97751	149743	164740										
[S].EPIPLESREEYMNG.[M]_	P47710						311556	400907	263841	262439	254439	1129488	1157836	1187982	929103	552351					
[S].EPIPLESREEYMNGM.[N]_	P47710						204407	176999	158245	190696	180707	342621	334972	318620	370369	306269					
[S].EPIPLESREEYMNGMN.[R]_	P47710						6241125	5972960	3223314	5376364	5607456										
[S].EPIPLESREEYMNGMN.[R]_1xOxidation [M]	P47710						825541	879905	887832	457573	320554	262321	396431	405206	258782	407676					
[S].EPIPLESREEYMNGMN.[R]_1xOxidation [M15]	P47710		290154	132378	96708	56226															
[S].EPIPLESREEYMNGMN.[R]_2xOxidation [M12; M15]	P47710						94484	136239	155072	50231	43797	80700	101339	98219	75207	90516					
[S].EPIPLESREEYMNGMNR.[Q]_1xOxidation [M]	P47710						297631	274103	264502	191161	91536	50392	126327	73084	49898	200949					
[S].EPIPLESREEYMNGMNRQ.[R]_	P47710						152476	170389	104161	184212	126723	103851	101937	127654	81806	93726					
[S].EQKDTLGNTQIKQ.[K]_	Q14512											36273	30907	20543	62671	36940					
[S].EQKDTLGNTQIKQK.[S]_	Q14512											27411	55495	52708	36292	10410					
[S].ESSEPIPLESREE.[Y]_	P47710											50056	61541	41519	61156	51427					
[S].ESSEPIPLESREEYMNG.[M]_	P47710												19819	21136	29869	20979					
[S].ESSEPIPLESREEYMNGMN.[R]_1xOxidation [M]	P47710						102436	150876		90024	65380										
[S].ESSEPIPLESREEYMNGMNR.[Q]_	P47710						1445452	1529827	969269	2011737	2033482	898267	933509	879201	1048329	1033968	163001	152992	125837	77655	63698
[S].ESSEPIPLESREEYMNGMNR.[Q]_1xOxidation [M15]	P47710						72047	65031	151756												
[S].EVSPNSKPSPNT.[K]_	P12272				108524	39962	21962	33459		40727	49173	13534		14183	14008	18392					
[S].EYPTYHTHGRYVPPS.[S]_	P15941											332342	578097	550551	295331	191590					
[S].EYPTYHTHGRYVPPSS.[T]_	P15941						365949	360652	1063214	264725	161375	871325	962513	853074	709282	565846	59567	165235	101107	32815	67426
[S].EYPTYHTHGRYVPPSSTD.[R]_	P15941											160068	282091	199589	180075	91510					
[S].FLSALEEYTKKLNTQ.[-]_	P02647							33652		55689	46531										
[S].FQPQPLIYPFVEPIPYGFLP.[Q]_	P05814											253027	271987	317496	193527	157271		37898			
[S].FQPQPLIYPFVEPIPYGFLPQ.[N]_	P05814											245291	258168	317100	390572	186132	28892	37345		51134	68009
[S].GAQEMVSSAKDTVATQLS.[E]_	O60664											23148	30450	27865							

Quantified modified peptide sequence	Protein ID	A_-80oC	A_-20oC_120h	A_4oC_6h	A_RT_6h	A_RT_24h	B_-80oC	B_-20oC_120h	B_4oC_6h	B_RT_6h	B_RT_24h	C_-80oC	C_-20oC_120h	C_4oC_6h	C_RT_6h	C_RT_24h	D_-80oC	D_-20oC_120h	D_4oC_6h	D_RT_6h	D_RT_24h
[S].GAQPILSKLEPQIA.[S]_	O60664											195412	209173	63809	135457	77713	129190	116502	109450	96811	29907
[S].GAQPILSKLEPQIASASE.[Y]_	O60664											126516	136507	132546	92053	52427	44104	48757	40728	28693	
[S].GAQPILSKLEPQIASASEY.[A]_	O60664											39711	43265	41411	31336	17628					
[S].GRITINGESPKHR.[S]_	Q8N7A1											285115	344205	329639	254703	140515	862872	687670	763657	924041	510584
[S].GSSEEQGGSSRALV.[S]_	P01833											26555	28300	32519	41279	39669	168515	225499	216300	227416	70710
[S].GSSEEQGGSSRALVST.[L]_	P01833						61001	74152	82629	37856	40195	35463	48861	54108	61902	41712					
[S].GSSEEQGGSSRALVSTLVPL.[G]_	P01833						148186	135624		156107	167220										
[S].GSSEEQGGSSRALVSTLVPLG.[L]_	P01833						479535	511087	390543	441008	467938	550188	598753	627478	577068	677033	350536	289055	266433	326273	193255
[S].GVALSPWVIQK.[N]_	P19835									17909	88496										
[S].GVALSPWVIQKNPLF.[W]_	P19835									24607	117879										
[S].GVDKTKSVVTG.[G]_	O60664											19144	23489	31455	13808	10729					
[S].GVDKTKSVVTGGVQ.[S]_	O60664											92868	109389	96334	74602						
[S].GVENALTKSELL.[V]_	Q99541											285776	288019	248507	170099	94753	27413	56053	57725		7594
[S].GVENALTKSELLVEQ.[Y]_	Q99541											193994	182879	173341	127847	88972	14463	12827	12857	22333	
[S].HEDMLVVDPK.[S]_	P10451											92392	79207	74326	61371	29394					
[S].HELDSASSEVN.[-]_	P10451						114185	171513	109859	180698	33978	175782	197988	235181	269245	116364					
[S].HMESEELNGAYK.[A]_	P10451						121352	86212		81125	15490	81740	73530	37101	95782	53554					
[S].HQNLKEFALTNPEK.[S]_	Q9H173									33301	26554										
[S].IPASSLPRLTPWIVAVAVI.[L]_	Q13410											108617	125995	123794	63832	44367					
[S].KEIPLSPMG.[E]_	Q13410											22528	28206	23330	44637	29382					
[S].KEIPLSPMGE.[D]_	Q13410											34724	23776		67135	40083					
[S].KEIPLSPMGEDS.[A]_	Q13410											63335	75611		40206	27009	25528	25133	13535		
[S].KEIPLSPMGEDSAPR.[D]_	Q13410				39904	20175						218939	174007		148701	68354	101601	117445	43384		20236
[S].KEIPLSPMGEDSAPRD.[A]_	Q13410														39689						
[S].KEIPLSPMGEDSAPRDADTLHSK.[L]_	Q13410											118437	138858	70883	104057						
[S].KEIPLSPMGEDSAPRDADTLHSKLIPTQ.[P]_	Q13410											109485	127060	110621	59606	55056					
[S].KLDYKPV.[-]_	P62333											29337	46251	25793	24768	14432					
[S].KLIPTQPSQ.[G]_	Q13410											54928	54180		32664						
[S].LGSKINVKVGGNSKGTLK.[V]_	P0C0L4												27121		11605	12263					
[S].LPRLTPWIV.[A]_	Q13410							87363		56144	29719										
[S].LSSSEESITEYKQK.[V]_	P05814						91425	101185		101153	69688	94822	88089	83332	94981	66645					
[S].LSSSEESITEYKQKVE.[K]_	P05814											45046	40694	39593	19857						
[S].LSSSEESITEYKQKVEK.[V]_	P05814					98251	455178	513156	452791	591518	268520	229389	247870	166432	333482	253521					
[S].MGIGLVKGGVSAVAGGVTAVGSAVVN.[K]_	Q8WUH6												23290	27585		43502					
[S].NEHSDVIDSQELSKVSREF.[H]_	P10451											20192	24819	24723	42468	22731					
[S].NIQGVPQNIQDQAKHMGVM.[A]_	Q99541											72517	73318	74959	40098	15051					
[S].NIQGVPQNIQDQAKHMGVMA.[G]_	Q99541											168428	172833	194101	142076	71816					
[S].NPTAHENYEKNNVMLQ.[W]_	P47710					31121															
[S].NPTAHENYEKNNVMLQW.[-]_1xOxidation [M14]	P47710						61013	63951		46910	12405										
[S].PAVLVHRDGREQEAEQMPEYR.[G]_	Q13410						36114	53451		100855	56245										
[S].PAVLVHRDGREQEAEQMPEYRG.[R]_	Q13410						14785		40183	32442	22335										
[S].PMGEDSAPRDADTLHSK.[L]_	Q13410											87260	104336	83380	91857	30895					
[S].PMGEDSAPRDADTLHSKLIPTQ.[P]_	Q13410											73831	66681	61390							
[S].PTIPFFDPQIPKLTD.[L]_	P05814				84496	54173	1700406	1226184	1082870	1514312	2481281	252938	272607	368337	863957	1730435				95946	271895
[S].PTIPFFDPQIPKLTDLENLHLPLP.[L]_	P05814						275180	312647	216262	335817	506345	359616	312295	400156	370432	421847					
[S].PTIPFFDPQIPKLTDLENLHLPLPL.[L]_	P05814											20388	29693	20497							
[S].PTIPFFDPQIPKLTDLENLHLPLPLL.[Q]_	P05814											130480	85733	125150	192134	210794					
[S].PTIPFFDPQIPKLTDLENLHLPLPLLQ.[P]_	P05814																				112447
[S].PTIPFFDPQIPKLTDLENLHLPLPLLQP.[L]_	P05814						52960	89690	113493	92746	107377	178358	109536	214862	154662	89606					
[S].PTIPFFDPQIPKLTDLENLHLPLPLLQPLM.[Q]_	P05814						90225	43195		94177	200638	326460	207922	298762	348148	413866					69025
[S].PTIPFFDPQIPKLTDLENLHLPLPLLQPLMQ.[Q]_	P05814							258680	208596	105380	36209	535348		284504	426116	174175					
[S].PTIPFFDPQIPKLTDLENLHLPLPLLQPLMQ.[Q]_1xOxidation [M30]	P05814																		13037		20902
[S].PTIPFFDPQIPKLTDLENLHLPLPLLQPLMQQV.[P]_	P05814						24339	28782	238122	72022	24039	56747	103049	20985	73739	43265					
[S].QLDDQSAETHSHKQS.[R]_	P10451											47301				25409					
[S].QLDDQSAETHSHKQSRLY.[K]_	P10451											155495	141713	91363	58763	116195					
[S].QNLGTNLPQLGQPSSTGPSNSEHPQPALDPR.[S]_	Q6UXA7																	25240	23033		
[S].QPVSSEMYRDPFGNPFA.[-]_	P98082						69750	35864													
[S].RASVDSGSSEEQGGSSRALVST.[L]_	P01833											137835	118127	59678	37449		132535	204090	130292	62136	
[S].RASVDSGSSEEQGGSSRALVSTLVPL.[G]_	P01833																114247	148076	120490	132357	103084
[S].RASVDSGSSEEQGGSSRALVSTLVPLG.[L]_	P01833						186391	192005	98703			2122711	2265786	1886875	1400687	359395	2586943	3406873	2625925	2783540	1588873
[S].REEYMNGMNR.[Q]_	P47710						302077	260204	261520	488501	139494	265964	243620	148445	228540	183338	26929	39137	24732		
[S].REEYMNGMNRQR.[N]_	P47710						38629	40685	49801	69134											
[S].REEYMNGMNRQRNIL.[R]_	P47710						113900	82447	144513	231196	136263										
[S].SAKDTVATQL.[S]_	O60664												30964								
[S].SAYLSTKDQYPYLK.[S]_	Q99541									80000	30776	2901235	3210565	864607	2024792	1303481	362722	425740	399723	238789	95530
[S].SEEQGGSSRALV.[S]_	P01833																28655	27141	29870	28860	11044
[S].SEEQGGSSRALVSTLVPLG.[L]_	P01833											128313	127195	174300	202008	79043	71036	81421	67046	66085	34862
[S].SEESITEYKQ.[K]_	P05814						46750	43687		44862											
[S].SEESITEYKQK.[V]_	P05814						57522		44593	45360	11022	103607	106889	108126	100557	92677					
[S].SEESITEYKQKV.[E]_	P05814						71130	80739		66627	32584	479003	464663	605564	545270	369006		25556	17100		46041
[S].SEESITEYKQKVE.[K]_	P05814						36562	78562		84633	18679			23271	29616	15390					
[S].SEPIPLESRE.[E]_	P47710						50252	53166		42601	24630										
[S].SEPIPLESREEY.[M]_	P47710						102298	79980		86316	44954										
[S].SEPIPLESREEYMNGMN.[R]_	P47710						309444	289540	217991	253035	411312	75952	54120	146605	90831	102159					16289

Quantified modified peptide sequence	Protein ID	A_-80oC	A_-20oC_120h	A_4oC_6h	A_RT_6h	A_RT_24h	B_-80oC	B_-20oC_120h	B_4oC_6h	B_RT_6h	B_RT_24h	C_-80oC	C_-20oC_120h	C_4oC_6h	C_RT_6h	C_RT_24h	D_-80oC	D_-20oC_120h	D_4oC_6h	D_RT_6h	D_RT_24h
[S].SEPIPLESREEYMNGMNR.[Q]_	P47710						291629	206166	486341	416111	188104	148624	148664	139522	145099	131965	18997	21049	17437	57377	14566
[S].SGVENALTK.[S]_	Q99541											323483	423685	119223	290391	151433	46578	21118			
[S].SGVENALTKSE.[L]_	Q99541											35026	37807	31556	37732						
[S].SGVENALTKSELLVEQYLPLTE.[E]_	Q99541											62035		50102	45069						
[S].SKVSGAQEMVSSAKDTVATQL.[S]_	O60664											66586	94121	62973		37270		20040	19323		
[S].SPPASPLQHLLPGKAVDLG.[P]_	Q96TA1											33077	22485	20238							
[S].SSEESITEYKQ.[K]_	P05814											31177	143718		30009	20601					
[S].SSEESITEYKQK.[V]_	P05814											62714	69619	71601	89447	62904					
[S].SSEESITEYKQKV.[E]_	P05814									32056		183379	149465	143603	180030	177946					
[S].SSEESITEYKQKVE.[K]_	P05814											24731	24816	20594	26443	23181					
[S].SSYSKQFTSSTSYN.[R]_	P02671																44986	43514	101111		
[S].STYDLMSSAYLSTKDQ.[Y]_	Q99541											35596	21963		26296						
[S].STYDLMSSAYLSTKDQYPYLK.[S]_1xOxidation [M6]	Q99541											94376	142062	130156	101945	55018					
[S].SYSKQFTSSTSYN.[R]_	P02671																31134	29895	64256		
[S].TDRSPYEKVS.[A]_	P15941											49044	67652	25616	43958	14519					
[S].TDRSPYEKVSAGNGGSSLSYTNPAVA.[A]_	P15941																33006	34617	32947		
[S].TKDQYPYLK.[S]_	Q99541		70878	30790		35183	27862	29065		58831	18687	595217	689011	476907	498956	205505	18962	43289	42824	22213	9553
[S].TPAPEARPVIG.[A]_	Q96AD5											34361	39965	37169	43192	22820					
[S].TPAPEARPVIGA.[L]_	Q96AD5											15289		29951							
[S].TPAPEARPVIGAL.[G]_	Q96AD5											34088	19233	12158	24877	15268					
[S].TSYNRGDSTFESKSY.[K]_	P02671			38121		23698															
[S].TTVKIVLK.[E]_	Q6WN34		37354		137723	31460	133972	114525		154124	153638										
[S].TVHLIEFARKN.[V]_	Q99541											40194	39107	68820	26157						
[S].VDSGSSEEQGGSSRA.[L]_	P01833																32849	25608	28737		13440
[S].VPQPKVL.[P]_	P05814											118845	137693	131727	178466	137083					
[S].VPQPKVLPIPQQVVPYPQRAV.[P]_	P05814						175592	115170	948462	99883	52329	50074	78533	59375	142094	191544					
[S].VPQPKVLPIPQQVVPYPQRAVPVQAL.[L]_	P05814															289767					
[S].YETSQLDDQSAETHS.[H]_	P10451											52324	42947	28615							
[S].YETSQLDDQSAETHSH.[K]_	P10451											428872	432969	313456	120538	24566					
[S].YETSQLDDQSAETHSHK.[Q]_	P10451											226372	185352	131706	76798	41517					
[S].YETSQLDDQSAETHSHKQ.[S]_	P10451											40320	38978								
[S].YETSQLDDQSAETHSHKQS.[R]_	P10451											260667	231299	208627	141326	59740					
[S].YETSQLDDQSAETHSHKQSR.[L]_	P10451											44050	43412								
[S].YETSQLDDQSAETHSHKQSRLY.[K]_	P10451											333235	449824	407079	323764	224794					
[S].YPPPPAGHSGPGPAGFPVPNQPVY.[N]_	O15162											43980	51616	45711	42243	23112					
[S].YPYYGTNLYQRRPAIAINNP.[Y]_	P07498											88851	120343	109627	49207	22714					
[S].YSKQFTSSTSYNRGDSTFESKSY.[K]_	P02671					27438															
[S].YTNPAVAATSANL.[-]_	P15941												58295			27792	84266	31453	29380	49709	13748
[T].AAAVSGAQPILSKLEPQIA.[S]_	O60664											89051	99968	96355	67175	38046		28170			
[T].APYVPMYYVPNSYPYYGTNLYQR.[R]_	P07498											173927	150927	163184	143018	61815					
[T].APYVPMYYVPNSYPYYGTNLYQRRP.[A]_	P07498											51144	27507	41981							
[T].DEDITSHME.[S]_	P10451						85257	63829		57105	29291										
[T].DEDITSHMESEELNGAY.[K]_	P10451						125598	149398	131679	224270	307534	26293	25125	26045	29701	22336					
[T].DLENLHL.[P]_	P05814						141998	100553		86102	98224	34822	32075	40757	62716	81765					
[T].DLENLHLP.[L]_	P05814						137060	159760	162793	219205	714122										
[T].DLENLHLPL.[P]_	P05814					48777	140108	157030		112964	210113										
[T].DLENLHLPLPL.[L]_	P05814						149608	182348		55441	82891										
[T].DLENLHLPLPLL.[Q]_	P05814				60930	429665	629901	564194	353456	677374	1783595	219536	203981	330606	376602	369341					
[T].DLENLHLPLPLLQP.[L]_	P05814		129562	113412		438059	368584	409972	276216	563876	1391620	173511	194930	193295	173307	164068					
[T].DLENLHLPLPLLQPL.[M]_	P05814					191264	728004	735353	349979	684784	806455	337598	283470	349463	468401	466801		18745			84450
[T].DLENLHLPLPLLQPLM.[Q]_1xOxidation [M16]	P05814		35799			54383	89155	93831		105753	234899	56013	77883	63188	111794	229623					29366
[T].DLENLHLPLPLLQPLMQ.[Q]_1xOxidation [M16]	P05814						144481			73503		162067	177070	228070	176187	180541					
[T].DLENLHLPLPLLQPLMQQ.[V]_	P05814						51486	33934	60253	50066	53485	18091		16915	17350	34924					
[T].DLENLHLPLPLLQPLMQQVPQPIPQT.[L]_	P05814						4040160	3464946	1317525	1423688	868714	6366328	5185541	6736990	4984433	2985218					
[T].DLENLHLPLPLLQPLMQQVPQPIPQT.[L]_1xOxidation [M16]	P05814												48108	63747	35119	21194					
[T].DLENLHLPLPLLQPLMQQVPQPIPQTL.[A]_	P05814											463680	446675	475117	390899	256531					
[T].DLENLHLPLPLLQPLMQQVPQPIPQTLA.[L]_	P05814											83791	62314	90460							
[T].DLENLHLPLPLLQPLMQQVPQPIPQTLAL.[P]_	P05814											122544	103125	148431	98120	50290					
[T].DVLFLVPTEIALAQHRANA.[K]_	P19835						50431	23219													
[T].EEDFYKLVSEFTITKGL.[R]_	P19835										72764										
[T].EEDFYKLVSEFTITKGLR.[G]_	P19835					114166	124088	120508		373122	644644										
[T].EEDFYKLVSEFTITKGLRGA.[K]_	P19835					28761	61985	56965	50431	150023	161818										
[T].EEELEKEAKKVEGFDLV.[Q]_	Q99541											337345	315880	304154	241189	153353					
[T].EGDGVYTLNDKKQWIN.[K]_	P00738							18232		24004	47701										
[T].EGDGVYTLNDKKQWINK.[A]_	P00738						105430	134077	95381	69640	34343										
[T].EGGFVEGVNKKL.[G]_	P19835						103139	75859		65197	32735										
[T].EGGFVEGVNKKLGLLG.[D]_	P19835						129434	133924		62064	68665										
[T].EGGFVEGVNKKLGLLGD.[S]_	P19835						61485	38325		23172	11318										
[T].EGGFVEGVNKKLGLLGDSVDIF.[K]_	P19835						1256405	1233586	1102129	845218	366927										
[T].EGGFVEGVNKKLGLLGDSVDIFK.[G]_	P19835						214110	270091	200819	282609	102406										
[T].ENSGYLEITKK.[M]_	P19835					28488		38705		50421	54228	34347	44168	22682	30593	24755					
[T].ESWAQDPSQENK.[K]_	P19835						30344	36027		26368	25591										
[T].FDVYTESWAQDPSQENK.[K]_	P19835						36522			115022	130617										
[T].GAKDAVTTTVT.[G]_	Q99541											62909	63633	16003	62946	41043					

Quantified modified peptide sequence	Protein ID	A_-80oC	A_-20oC_120h	A_4oC_6h	A_RT_6h	A_RT_24h	B_-80oC	B_-20oC_120h	B_4oC_6h	B_RT_6h	B_RT_24h	C_-80oC	C_-20oC_120h	C_4oC_6h	C_RT_6h	C_RT_24h	D_-80oC	D_-20oC_120h	D_4oC_6h	D_RT_6h	D_RT_24h
[T].GAKDAVTTTVTGAKDSVA.[S]_	Q99541											36257	60906	39887	33241	27528					
[T].GAKDAVTTTVTGAKDSVASTITGVMD.[K]_	Q99541											153365	142408	100197	100221	48977					
[T].GAKDSVASTITGVMD.[K]_	Q99541											201693	233226	81715	183590	92730	75777	98711	73064	33201	15431
[T].GAKDSVASTITGVMDKTKGAVTG.[S]_	Q99541											198602	213370	161861	136390	81898					
[T].GSGLEGDHLSDTSTTSLELDSRRH.[-]_	P12272						26736	32419		35268	23115										
[T].HQIYPVTQPLAPVH.[N]_	P05814												37808			24926					
[T].HQIYPVTQPLAPVHN.[P]_	P05814											96361	145485	138712	118647	61341					
[T].HRIHWESASLLR.[S]_	P01024		48061	80997		14282	25796	19322		20703	25933										
[T].IESLSSSEESITEYKQKV.[E]_	P05814															38271					
[T].IESLSSSEESITEYKQKVE.[K]_	P05814						56840	57570		46119	30173	39287	22351	33781	66454	87350					
[T].IPFFDPQIPKL.[T]_	P05814					156894	1109939	868656		557640	977700	329806	354572	366318	1008556	2378575					50061
[T].IPFFDPQIPKLTD.[L]_	P05814					95877	835659	692835		483116	810071	145259	133541	151459	386557	1150698					
[T].IPFFDPQIPKLTDL.[E]_	P05814						61155	46743	211859	89519	126274					209823					
[T].IPFFDPQIPKLTDLE.[N]_	P05814											71695		128832	77485	191493					
[T].IPFFDPQIPKLTDLEN.[L]_	P05814							179712	277670												
[T].IPFFDPQIPKLTDLENLH.[L]_	P05814											50425	72989								
[T].IPFFDPQIPKLTDLENLHL.[P]_	P05814						43213	35941					46683	58593	17348						
[T].IPFFDPQIPKLTDLENLHLPL.[P]_	P05814											191996	123926	231764	174145	88527					
[T].IPFFDPQIPKLTDLENLHLPLPL.[L]_	P05814													28063	23317	20332					
[T].IPFFDPQIPKLTDLENLHLPLPLL.[Q]_	P05814													32012							
[T].IPFFDPQIPKLTDLENLHLPLPLLQ.[P]_	P05814						141509	83167			58109	125216	129514	138970	111988	97817					
[T].IPFFDPQIPKLTDLENLHLPLPLLQP.[L]_	P05814						314674	293776	280241	202584	148495	357481	270617	357337	300568	205163					
[T].IPFFDPQIPKLTDLENLHLPLPLLQPL.[M]_	P05814													68217	59645	37886					
[T].IPFFDPQIPKLTDLENLHLPLPLLQPLM.[Q]_	P05814											240811	154965	175130	181008	140736					
[T].IPFFDPQIPKLTDLENLHLPLPLLQPLM.[Q]_1xOxidation [M28]	P05814											24362	23285	21394	19234	16646					
[T].IPFFDPQIPKLTDLENLHLPLPLLQPLMQ.[Q]_	P05814											104641	83561	89165	124475	119323					
[T].ITGVMDKTKGAVTG.[S]_	Q99541											33080	29101	35840	23589	19853					
[T].KALENPQPHPGWQGTLK.[A]_	P19835						31965	37056		50836	28001										
[T].KDQYPYLK.[S]_	Q99541											285826	316702	186394	230249	120424					
[T].LALPPQPLWSVPQP.[K]_	P05814									56374	90941										
[T].LALPPQPLWSVPQKV.[L]_	P05814											54969	69294	85451	122671	149805					
[T].LALPPQPLWSVPQKVLPIPQ.[Q]_	P05814														92724	181066					
[T].LALPPQPLWSVPQKVLPIPQQ.[V]_	P05814																82890	37967		151632	68935
[T].LALPPQPLWSVPQKVLPIPQQVVPYPQ.[R]_	P05814			20675		582124															
[T].LALPPQPLWSVPQKVLPIPQQVVPYPQRAV.[P]_	P05814																82546	18865	40949	150297	41709
[T].LALPPQPLWSVPQKVLPIPQQVVPYPQRAVPVQ.[A]_	P05814														214139	1591359	46704		48154		403253
[T].LDPDTAHPHLF.[L]_	Q13410													40951	14976						
[T].LPTKETIEQ.[E]_	P63313						39890	40888									41464	44401	46175		19688
[T].LPTKETIEQE.[K]_	P63313																	28897	33496		
[T].LPTKETIEQEKRSEIS.[-]_	P63313						95891	82661	89215	36421											
[T].LVQDGIAK.[G]_	Q13410																64741	68822	46780	57203	14239
[T].LVQDGIAKGRVA.[L]_	Q13410																35883	62060	29732		
[T].NEERKTLLSNLEEAK.[K]_	P10909						79579	86164		96566	15817										
[T].NLYQRRPAIAINNPYVPRT.[Y]_	P07498												32396		29891	61526					
[T].QPLAPVHNPISV.[-]_	P05814						39245673	39018754	37145317	32797775	34259593	25865358	28299988	25071740	34160609	51296523	11903838	10213606	10623810	19385005	22311665
[T].SALPIIQKLEPQIAVANTY.[A]_	Q99541											121076	104998	128158	94217	86874					
[T].SEVSPNSKPSPNT.[K]_	P12272				163811	42621	15226	18481		78527	92518										
[T].SPQPASEDTLTYADLDMVHLN.[R]_	P78324												51206	37699	26163						
[T].SPVDDFRQPRYSSGGNF.[E]_	Q16625											54493	57648		43165	39909					
[T].SQLDDQSAETHSHKQS.[R]_	P10451											17191	14973	15490	15507	13956					
[T].SQLDDQSAETHSHKQSRLY.[K]_	P10451											204303	281830	183167	318916	189624					
[T].TFDVYTESWAQDPSQENKKK.[T]_	P19835						139429	190658	266049	140070	179426										
[T].VPSMGIGLVKGGVSAVAGGVTAVG.[S]_	Q8WUH6											48915	28127	42209	24281						
[T].VPVGRVETGVLKPGMVVT.[F]_	P68104											43463	44515	36686	28369	29995					
[T].VVDFETDVLFLVPTEIALAQH.[R]_	P19835						182718	165629	146686	164322	288315										
[T].VVDFETDVLFLVPTEIALAQHR.[A]_	P19835		82219			67929	527184	435605	496217	389229	507692	78914	96739			66106					
[T].VVDFETDVLFLVPTEIALAQHRAN.[A]_	P19835										24731										
[T].YYANPAVVRPHAQIPQR.[Q]_	P07498											36383	41437	61143	43838	50504					
[V].ADTRDQADGSR.[A]_	P01833						500784	503887	1099737	473505	377629						218137	312563	234457	37365	104179
[V].ADTRDQADGSRASVD.[S]_	P01833						179128	187643	107958	125604	24349										
[V].APEEHPVLL.[T]_	P60709											55082	53542	51661	47212	36563					
[V].APEEHPVLLTEAPLNP.[K]_	P60709											180463	156283	174075	161646	115832					
[V].APQKMAGASPTKDDSKDSDFWK.[M]_	Q13438							31438		67800	50987										
[V].AQDLNAPSDWDSRGKDS.[Y]_	P10451											207771	211261	193349	148076	84607					
[V].DADADPRQYADTVKALRV.[R]_	P08571						61527	57931		54370											
[V].DALRTHLAPYSDEL.[R]_	P02647					29194															
[V].DALRTHLAPYSDELR.[Q]_	P02647					47452	16341			47952	47578										
[V].DDTQFVRFD.[SN]_	P13746; P30443; Q07000; P30510; P13747																39563	42618	33744	33380	23779
[V].DDTQFVRFDSDAASPRGEP.[R]_	Q07000; P30510																29871	22286	20346	21913	10990
[V].DDTQFVRFDSDAASPRGEPR.[A]_	Q95604							93322	95698												
[V].DDTQFVRFDSDAASQKMEPR.[A]_	P30443		64343	37314		35949															
[V].DFETDVLFLVPTEIALAQHRANA.[K]_	P19835		122739	90637		47155	928579	894918	536657	446251	445105	120059	17893	122263	88548	56990					
[V].DLDQLLDMSYEQLMQLYSARQ.[R]_	P62841													32849							
[V].DPQPSVVTRVVN.[L]_	Q99541											258528	297596	408772	259631	159920					

Quantified modified peptide sequence	Protein ID	A_-80oC	A_-20oC_120h	A_4oC_6h	A_RT_6h	A_RT_24h	B_-80oC	B_-20oC_120h	B_4oC_6h	B_RT_6h	B_RT_24h	C_-80oC	C_-20oC_120h	C_4oC_6h	C_RT_6h	C_RT_24h	D_-80oC	D_-20oC_120h	D_4oC_6h	D_RT_6h	D_RT_24h
[V].DPQPSVVTRVVNLPLV.[S]_	Q99541											32578		57766	30289						
[V].DSGSSEEQGGSSRALVSTLVPL.[G]_	P01833											1976863	1928045	2159867	1841464	963675	902414	786856	783500	775221	521154
[V].DSGSSEEQGGSSRALVSTLVPLGLVL.[A]_	P01833											108874	106323	103279	85209	43208					
[V].DSKNFDDYMKSLGVGFAT.[R]_	P05413										63683										
[V].DVTLDPDTAHPHLF.[L]_	Q13410						24196	36935	140333			422834	401633	631052	401463	210098					
[V].DVTLDPDTAHPHLFLY.[E]_	Q13410											42603	23835	75098	53820	21094					
[V].DVTLDPDTAHPHLFLYEDSK.[S]_	Q13410											47752		38717	39801						
[V].EGFDLVQKPS.[Y]_	Q99541						47892	52595		38027	53563										
[V].EISIPASSLPRL.[T]_	Q13410						102423	94434						24035	33455		71936	107486	41639		23809
[V].EISIPASSLPRLT.[P]_	Q13410						44877	28800	81596	19630	35281										
[V].EISIPASSLPRLTP.[W]_	Q13410						70359	77195	57117	36814		48261	52213	61218	43330	18119	54902	154792	62810	89578	27100
[V].EISIPASSLPRLTPW.[I]_	Q13410							96052													
[V].EISIPASSLPRLTPWIVAV.[A]_	Q13410						96984	54109	264013	64940											
[V].EKVKHEDQQQGEDEHQDK.[I]_	P05814					35746	46710	52872	72965	55775	12860										
[V].EKVKHEDQQQGEDEHQDKIYP.[S]_	P05814						29277	23477	59476	58646	47139										
[V].EKVQAAVGTSAAPVPSDNH.[-]_	P02649						32206	36921		27291											
[V].ENALTKSELLVEQ.[Y]_	Q99541											79386	98187	128004	77428	46650					
[V].EPIPYGFLPQNILPLAQPAVV.[L]_	P05814											51791	65278	54154	52551						
[V].EPLRAELQEGARQK.[L]_	P02647											27448	27012	27884	15679						
[V].ETEPEPELR.[Q]_	P02649						23139	16611		51270	17231										
[V].FPYPYPFRPLPPIP.[F]_	Q8NFU4																209691	201225	156854	215211	149015
[V].FPYPYPFRPLPPIPFP.[R]_	Q8NFU4																92544	81926	77601	64208	112415
[V].HRDGREQEAEQMPEY.[R]_	Q13410						86124	106884	111030	171337	156651										
[V].KHEDQQQGEDEHQDKIYP.[S]_	P05814					31112	20657	25509	34887	66344	53888										
[V].LDSGFREIENK.[A]_	P01833						56513	73593	80591	89553	45012	31443	79704			24341	32697	46359	47594	31279	20873
[V].LKSPTIPFFDPQIP.[K]_	P05814						94783	72569		83766	103936	49823	96314	29174	39276	116637					
[V].LPIPQQVVPYP.[Q]_	P05814						876837	920197	805648	1871684	2680136	434975	531022	523303	405308	180157					
[V].LPVPQPEIMEVPKA.[K]_1xOxidation [M9]	P05814						137549	160187	141029	103395											
[V].LPVPQPEIMEVPKAK.[D]_	P05814		21905		46627	23303						35705	33680	31662	30780	81149					
[V].LPVPQPEIMEVPKAKDTV.[Y]_1xOxidation [M9]	P05814		26158	41700																	
[V].LVHRDGREQEAEQMPEYR.[G]_	Q13410						62537	63531	82564	66171	28046										
[V].MPVLKS.[P]_	P05814						111310	77830	79456	84509	53643										
[V].MPVLKSPT.[I]_	P05814						2192652	2787047	523569	2390302	2641937	222462	215801	308620	343525	467378					
[V].MPVLKSPTIP.[F]_1xOxidation [M1]	P05814											102882	138021	133827	205178	339760					
[V].MPVLKSPTIPF.[F]_	P05814						1026149	1097992		700393	461003										
[V].MPVLKSPTIPFF.[D]_1xOxidation [M1]	P05814									30327											
[V].MPVLKSPTIPFFDPQI.[P]_	P05814						131960	183468	59430	167644	189519	62151			41612	155781					
[V].MPVLKSPTIPFFDPQIPKLTDL.[E]_	P05814											455235	564458	501829	420429	227419					
[V].MPVLKSPTIPFFDPQIPKLTDLENL.[H]_	P05814						153893	359634	671855	140697	100595	173020	111254	335738	139191	282292					
[V].MPVLKSPTIPFFDPQIPKLTDLENLHLPLP.[L]_	P05814											174624	149009	175891	157696	138039					
[V].PGLDGAQIPRDPSQQELPR.[L]_	P49327																	12668			14553
[V].PGSQTVVVKEITIHTK.[L]_	O00592											29135	31891	22711	18841						
[V].PKAKDTVYTK.[G]_	P05814				162769	213659	143546	155460	257099	346014	193708	41552	49416	34121	91835	168293					33715
[V].PKAKDTVYTKGR.[V]_	P05814											130652	178930	201905	166986	84950					
[V].PNSYPYYGTNLYQR.[R]_	P07498							27352			26599	32683	45753	37927	27974	34794					
[V].PNSYPYYGTNLYQRRPAIAINNPYVP.[R]_	P07498											235808	285679	468985	281969	314658					
[V].PPSSTDRSPYEKVSAGNGGSSL.[S]_	P15941						49456	52590		22158											
[V].PPSSTDRSPYEKVSAGNGGSSLS.[Y]_	P15941						38743	79350		38246											
[V].PQNIQDQAKHMGVMA.[G]_	Q99541											56215	49087	48046	42482	19734					
[V].PQPEIMEVPK.[A]_	P05814					61086	127234	147815	698292	140863	160723										
[V].PQPEIMEVPKAKDTVYTKGR.[V]_	P05814											37302	39353	33794	24431	32053					
[V].PQPKVLPIPQQ.[V]_	P05814											20041	20673		21974	58249					
[V].PQPKVLPIPQQVVPYPQR.[A]_	P05814			28250		301943	39039			82388	234921	434953	476220	464677	328767	256837	51362	45301	38905		18575
[V].PQPKVLPIPQQVVPYPQRAVPVQ.[A]_	P05814															69593					
[V].PQPKVLPIPQQVVPYPQRAVPVQAL.[L]_	P05814													46709		28156					
[V].PTEIALAQHRANA.[K]_	P19835						19443	39401		47569	161007										
[V].PTEIALAQHRANAK.[S]_	P19835						35066	27630	189232												
[V].PTHWEPYTTENSGYLEITK.[K]_	P19835							34719		34957	45460										
[V].PTHWEPYTTENSGYLEITKK.[M]_	P19835						126883	132914		150058	56089										
[V].PYPQRAVPVQ.[A]_	P05814												26169	18302	32963	88974					45170
[V].PYPQRAVPVQA.[L]_	P05814						51163	52639		42131	29247										
[V].QALLLNQELLLNPTHQIYPVTQPLAPVHNPISV.[-]_	P05814																146397	160553	171044	199379	450962
[V].QKPSYVVRLG.[S]_	Q99541											156263	239571	166378	160825	141963	47810	41089	15154		
[V].SEFTITK.[G]_	P19835									39144	13826										
[V].SEFTITKGLR.[G]_	P19835										47588										
[V].SEHQLLHDKGKSIQDL.[R]_	P12272							30681		93965	55612										
[V].SEHQLLHDKGKSIQDLR.[R]_	P12272						43539	53455	100885	151833	81629	50302	49590	28614	45776	34636		68767			
[V].SEQKDTLGNTQIKQK.[S]_	Q14512											96364	136820	109764	122532	43384					
[V].SPAVLVHRDG.[R]_	Q13410											28479	45727	31960				30332	27415		
[V].SPAVLVHRDGREQEAEQMPEYR.[G]_	Q13410						355328	333899	415481	339089	139100	123973	177881	121674	114529	48380		85159			
[V].SPAVLVHRDGREQEAEQMPEYRG.[R]_	Q13410						208538	151733	314799	177997	117213	25600	31253	44928	44338	28112					
[V].TEEDFYK.[L]_	P19835									39477	11789										
[V].TEEDFYKLVSEFTITKGL.[R]_	P19835									19985											
[V].TGSGLEGDHLSDTSTTSLELDSRRH.[-]_	P12272						28075	41654													

Quantified modified peptide sequence	Protein ID	A_-80oC	A_-20oC_120h	A_4oC_6h	A_RT_6h	A_RT_24h	B_-80oC	B_-20oC_120h	B_4oC_6h	B_RT_6h	B_RT_24h	C_-80oC	C_-20oC_120h	C_4oC_6h	C_RT_6h	C_RT_24h	D_-80oC	D_-20oC_120h	D_4oC_6h	D_RT_6h	D_RT_24h
[V].TLDPDTAHPHLF.[L]_	Q13410											94781	81741	160299	86478						
[V].TLDPDTAHPHLFLY.[E]_	Q13410											15075	15750	36599		25699					
[V].TTVASHTSDSDVPSGVTEVVVK.[L]_	P10909											48447	38597	51793	34069	23564					
[V].VDFETDVLFLVPTEIALAQHRANA.[K]_	P19835						227314	184982			96124										
[V].VLPVPQPEIMEVPK.[A]_	P05814						117457	112541	88459	137573	97522	71603	82782	97780	202361	227658					
[V].VLPVPQPEIMEVPKA.[K]_	P05814						40436	27510		47034	43151										
[V].VLPVPQPEIMEVPKAKDTVYT.[K]_	P05814						1625736	1788885	1368101	965167	763680	441717	486231	508258	432807	356704					
[V].VPGLDGAQIPRDPSQQELPR.[L]_	P49327						92303	122934	85589	70017	100319	145027	129814	115239	74048	89713	28736	35063	20118		
[V].VPYPQRAV.[P]_	P05814						68633	68193	398807	91989	46413	56711	72217	46525	96985	44583					
[V].VPYPQRAVPV.[Q]_	P05814						164149	174305	54054	60423	45528										
[V].VPYPQRAVPVQAL.[L]_	P05814						115491	92583	102806	69107	91713										
[V].VPYPQRAVPVQALL.[L]_	P05814											155094	211395	191730	302048	811567		46053	25492	55874	142342
[V].VPYPQRAVPVQALLL.[N]_	P05814				72992	140221	123779	122024		229661	271875	80680	81137	91552	349803	1088281				76850	313876
[V].VPYPQRAVPVQALLLN.[Q]_	P05814											83974	104405	98498	119220	713474					
[V].VPYPQRAVPVQALLLNQ.[E]_	P05814						19725	13629		26508	48017	29472	31037	34339	42213	92243					
[V].YTEGGFVEGVNKKLG.[L]_	P19835						43891	56797		31066											
[V].YTEGGFVEGVNKKLGLLGDSVDIF.[K]_	P19835						124830	112899		124901	200190										
[V].YTEGGFVEGVNKKLGLLGDSVDIFK.[G]_	P19835						359842	397021	334298	342460	122574										
[V].YTESWAQDPSQENK.[K]_	P19835							33430		24560	35979										
[V].YTESWAQDPSQENKK.[K]_	P19835						72016	81193	68905	54302	48874										
[V].YTKGRVMPVLK.[S]_1xOxidation [M7]	P05814						419344	411498	807530	623917	125452										
[V].YTLNDKKQWINK.[A]_	P00738										35079										
[W].DDMEKIWH.[H]_	P60709; P68133											46687	51015	47695	56486	61467					
[W].DQEPAREQAGGGWRA.[R]_	Q86X29																	37221	35268	40862	33747
[W].DSRGKDSYETSQLD.[D]_	P10451						249018	182418		84610	31070	33001	31866	59640	53225	65652					
[W].DSRGKDSYETSQLDD.[Q]_	P10451													20620	17161	21826					
[W].EFVRDLLLSPEE.[N]_	Q9UKW6		70240	133108	89534	46803												7954			36180
[W].EPYTTENSGYLEITK.[K]_	P19835						54248	52121	68980	45046	51827										
[W].LVGPFAPGITEKAPEEKK.[-]_	O60664											84417	82129	66915	48438	28400					
[W].SVPQPKVLPIPQQ.[V]_	P05814																233331	261124	234222	172531	134409
[W].SVPQPKVLPIPQQV.[V]_	P05814						146395	97095	56713	46636		45064	55101	39889	52927	44462					
[W].SVPQPKVLPIPQQVVPYPQRAV.[P]_	P05814						138228	90585	165384	61769	50622	36017	37736	60951	108670	185070		20409	32886		40912
[W].SVPQPKVLPIPQQVVPYPQRAVP.[V]_	P05814											253155	270217	260476	219280	179387					
[W].SVPQPKVLPIPQQVVPYPQRAVPVQA.[L]_	P05814											1060405	1221191	1163248	1005768	992595	650436	640430	638058	516072	488098
[W].SVPQPKVLPIPQQVVPYPQRAVPVQAL.[L]_	P05814											18691	30027	20344		202066					
[W].SVPQPKVLPIPQQVVPYPQRAVPVQALLL.[N]_	P05814											182113	207869	173985	166802	232307					
[W].SVPQPKVLPIPQQVVPYPQRAVPVQALLLN.[Q]_	P05814											84105	109511	34504	53117	108087					
[W].VGADHADDIQYVFGKPFATPTGYRPQD.[R]_	P19835						82545	69635		48322	73154										
[Y].ANPAVVRPHAQIPQ.[R]_	P07498												54580		20064						
[Y].ANPAVVRPHAQIPQRQ.[Y]_	P07498															43961					
[Y].ANPAVVRPHAQIPQRQYLP.[N]_	P07498		35282			34064						79221	62942	64277	86992	109073					
[Y].ANPAVVRPHAQIPQRQYLPN.[S]_	P07498															56372					
[Y].ANPAVVRPHAQIPQRQYLPNS.[H]_	P07498											149469	183141	138070	102997	109478					
[Y].ANPAVVRPHAQIPQRQYLPNSHPPTV.[V]_	P07498											105663	107098	89904	87658	108259					
[Y].DAAKRGPGGAWAAEVISNA.[R]_	P0DJI9		56291	38150		30728															
[Y].DEGSIIVKL.[G]_	P35606											46556	46527	60455	32978	11121	32973	40938	33733	30926	17042
[Y].DHINEGKLW.[K]_	Q9UDW1					26585					88326										
[Y].DIDVAKVNTLIRPDGEKK.[A]_	P62750											54082	43596	50433	31689						
[Y].DLMSSAYLSTKDQ.[Y]_	Q99541											71045	80880	30149	64824	25366					
[Y].DLMSSAYLSTKDQYPYLK.[S]_	Q99541											217371	195671	221264	160643	81895					
[Y].DQALQQAVVDDDANNAK.[A]_	P60033						64501	74345	71173	40070	25344										
[Y].DQSAYDGKDYIALNEDLR.[S]_	P30510																41984	37603	44150	19913	20784
[Y].DSFKLQTKEFQVLK.[S]_	P15907					35031	47660	37747			91584										
[Y].EDSKSVRLE.[D]_	Q13410											188386	191934	255003	154409	74742					
[Y].EEALVHLK.[V]_	Q13410						39944	55984		89117	80790										
[Y].EEIVKEVSTYIKK.[I]_	P68104						47712			85535	45744	121344	98208	102230	140206	95409	36409	38304	28355		23921
[Y].ETSQLDDQSAETHSH.[K]_	P10451											98023	104860	170145	190256	69079					
[Y].ETSQLDDQSAETHSHK.[Q]_	P10451											84808	97875	102982	112856	53743					
[Y].ETSQLDDQSAETHSHKQ.[S]_	P10451											24904	24555	27196	28493	25543					
[Y].ETSQLDDQSAETHSHKQS.[R]_	P10451											347352	11811		421853	209012					
[Y].ETSQLDDQSAETHSHKQSRL.[Y]_	P10451											28658	23393		22626	41099					
[Y].ETSQLDDQSAETHSHKQSRLY.[K]_	P10451						62621		53362	64383		1005762	1297096	1157161	1266936	1101878					
[Y].GFLPQNILPLAQPAVVLPVPQPE.[I]_	P05814						83115	125117	465027	94901	175555										
[Y].GFLPQNILPLAQPAVVLPVPQPEI.[M]_	P05814																28879		17102		54586
[Y].GFLPQNILPLAQPAVVLPVPQPEIMEVPK.[A]_1xOxidation [M25]	P05814						339029	373015	273307	231356	60925	57405	34578	33562	44505			24434		60461	20502
[Y].GFLPQNILPLAQPAVVLPVPQPEIMEVPKA.[K]_1xOxidation [M25]	P05814						33234	46184		98982											
[Y].GFLPQNILPLAQPAVVLPVPQPEIMEVPKAK.[D]_	P05814						1402966	1192522	1427449	913410	259496	291833	228193	250180	161087	146046					
[Y].GGGGSGGGYGGGSGSRGGSGGSY.[G]_	P35527															19412					
[Y].GTQTLEGGVEKPHSLL.[S]_	P01133												57476			19974					
[Y].LPLTEEELEKEAK.[K]_	Q99541						38727	62858		62248	71327	48716	59496	44893	56027	30467					
[Y].LPLTEEELEKEAKKVE.[G]_	Q99541											24967		22724	16670	14268					
[Y].LPLTEEELEKEAKKVEGFDLV.[Q]_	Q99541											127324	139118	100641	132675	77729					
[Y].LPNSHPPT.[V]_	P07498						52618	58118	31668	44302	14723										
[Y].LSTKDQYPYLK.[S]_	Q99541											1269741	1458696	717309	820535	520692	70911	102866	93127	45931	21453

Quantified modified peptide sequence	Protein ID	A_-80oC	A_-20oC_120h	A_4oC_6h	A_RT_6h	A_RT_24h	B_-80oC	B_-20oC_120h	B_4oC_6h	B_RT_6h	B_RT_24h	C_-80oC	C_-20oC_120h	C_4oC_6h	C_RT_6h	C_RT_24h	D_-80oC	D_-20oC_120h	D_4oC_6h	D_RT_6h	D_RT_24h
[Y].MNGMNRQRNILR.[E]_	P47710						62530	77925		130783	64656										
[Y].NKYPDAVATWLNPDPS.[Q]_	P10451						46594					181420	194810	185738	99786	48121		44024			
[Y].NKYPDAVATWLNPDPSQ.[K]_	P10451						62928	71774	84863		76451	306003	317060	354026	284415	205260					
[Y].NKYPDAVATWLNPDPSQK.[Q]_	P10451						296212	307291	159591	205723	545401	1796433	1769186	1904845	2052026	2097887		28293			24805
[Y].NKYPDAVATWLNPDPSQKQN.[L]_	P10451		56430	62864			99911	112882	61059	78071		657299	657474	674843	817252	657319	49713	62485	46000	57310	46788
[Y].NKYPDAVATWLNPDPSQKQNL.[L]_	P10451						78475	83059	290462	79155	89301	384257	399632	471329	421375	322611	124101	78489	126225	266620	109277
[Y].NKYPDAVATWLNPDPSQKQNLLAPQ.[N]_	P10451						213225	186945	116897	131755	157135	574059	541391	555647	572663	547942					
[Y].NQLQLQAAHAQEQ.[I]_	P47710		75017	55638	69139																
[Y].PDATDEDITSH.[M]_	P10451						130418	155041	114499	87729	61643	350494	343738	334596	318604	174154					
[Y].PDATDEDITSHMESEELNGAY.[K]_	P10451											54997	58607	44082	32959	34869					
[Y].PDAVATWLNPDPSQK.[Q]_	P10451											112790	116699	133518	126971	78080					
[Y].PERLQNPSE.[S]_	P47710						1183559	849284	426773	1319968	274279	86554	74048	72070	162081	107575					
[Y].PERLQNPSESSEPIPLES.[R]_	P47710						63068	54487		38020	34816										
[Y].PERLQNPSESSEPIPLESREEYMNGMN.[R]_	P47710											100576	152266	147174	82401	34380					
[Y].PTYHTHGRYVPPSS.[T]_	P15941											271922	430349	296974	153459	150611					
[Y].PVTQPLAPVHN.[P]_	P05814																	24984			
[Y].SLNGHEFDFIHDVNKK.[H]_	Q08431					42392					58831										
[Y].TEGGFVEGVNKKLG.[L]_	P19835					53779	153925	153756	76313	192063	192737	42108	48167	36044	114500	104978					
[Y].TEGGFVEGVNKKLGLLG.[D]_	P19835						33906	31021	59157	61899	82431										
[Y].TEGGFVEGVNKKLGLLGDSVDIF.[K]_	P19835						525519	474739	511750	401476	255359										
[Y].TEGGFVEGVNKKLGLLGDSVDIFK.[G]_	P19835						502925	533491	373817	572674	80834										
[Y].TESWAQDPSQENK.[K]_	P19835						29468	54374		32532	37899										
[Y].TESWAQDPSQENKK.[K]_	P19835						33007	49794	54884	84073	49368										
[Y].TKGRVMPVL.[K]_	P05814						99175	89932	75169	411544	217784										
[Y].TLNDKKQWINK.[A]_	P00738						45330	21801	154168	22328	34469										
[Y].TNPAVAATSANL.[-]_	P15941																41965	35870	38333	18195	
[Y].VPFPPFSDISNPTAHE.[N]_	P47710						75491	68497	54777	41053	30324		20222	19676	34591	26816					
[Y].VPFPPFSDISNPTAHENY.[E]_	P47710						54802														
[Y].VPMYYVPNSYPYYGTNLYQ.[R]_	P07498											131406	126938	115869	108218	30649					
[Y].VPMYYVPNSYPYYGTNLYQR.[R]_	P07498											154290	97483	72952	43451	28469					
[Y].VPNSYPYYGTNLYQR.[R]_	P07498							39156			27323										
[Y].VPNSYPYYGTNLYQRRPAIAINNP.[Y]_	P07498											73417		89412							
[Y].YANPAVVRPH.[A]_	P07498						84943	45143	52130	242886	72001										
[Y].YANPAVVRPHA.[Q]_	P07498											26033	29442			19414					
[Y].YANPAVVRPHAQIPQRQ.[Y]_	P07498							35804				58664	47047	55664	43235	73119					
[Y].YANPAVVRPHAQIPQRQYLPNS.[H]_	P07498											163683	174401	117378	130373	111149					
[Y].YANPAVVRPHAQIPQRQYLPNSHPPTVV.[R]_	P07498						365326	272064	331254	269780	460882										
[Y].YLAGRDLSRLPQLVGVSTPLQGGSNSAAAIGQSSGELR.[T]_	P15291						60424	77805	108252		39261										

Supplementary Table S2 Assignment of quantified peptides to proteins.
Functions and localization of proteins are shown.

Protein	Entry name	Gene names	Protein names	function	localization	all conditions	-80oC	6h RT	24h RT	4oC	-20oC
A0JLT2	MED19_HUMAN	MED19	Mediator of RNA polymerase II transcription subunit 19 (Lung cancer metastasis-related protein 1)	regulation	other (intracellular compartments)	A0JLT2	A0JLT2	A0JLT2	A0JLT2	A0JLT2	A0JLT2
A6ND01	JUNO_HUMAN	IZUMO1R	Sperm-egg fusion protein Juno (Folate receptor 4)	other	integral component of membrane	A6ND01	A6ND01	A6ND01	A6ND01	A6ND01	A6ND01
B1AK53	ESPN_HUMAN	ESPN	Espin (Autosomal recessive deafness type 36 protein)	other	other (intracellular compartments)	B1AK53	B1AK53	B1AK53	B1AK53	B1AK53	B1AK53
O00161	SNP23_HUMAN	SNAP23	Synaptosomal-associated protein 23 (SNAP-23)	transport	extracellular exosome	O00161	O00161	O00161	O00161	O00161	O00161
O00264	PGRC1_HUMAN	PGRMC1	Membrane-associated progesterone receptor component 1 (mPR)	other	integral component of membrane	O00264	O00264	O00264	O00264	O00264	O00264
O00391	QSOX1_HUMAN	QSOX1	Sulfhydryl oxidase 1 (hQSOX) (EC 1.8.3.2)	regulation	integral component of membrane	O00391	O00391	O00391	O00391	O00391	O00391
O00560	SDCB1_HUMAN	SDCBP	Syntenin-1 (Melanoma differentiation-associated protein 9) (MDA-9)	migration	extracellular exosome	O00560	O00560	O00560	O00560	O00560	O00560
O00592	PODXL_HUMAN	PODXL	Podocalyxin (GCTM-2 antigen)	migration	integral component of membrane	O00592	O00592	O00592	O00592	O00592	O00592
O14669	TMG2_HUMAN	PRRG2	Transmembrane gamma-carboxyglutamic acid protein 2 (Proline-rich gamma-carboxyglutamic acid protein 2)	other	integral component of membrane	O14669	O14669	O14669	O14669	O14669	O14669
O14672	ADA10_HUMAN	ADAM10	Disintegrin and metalloproteinase domain-containing protein 10 (ADAM 10) (EC 3.4.24.81)	response to cytokine	integral component of membrane	O14672	O14672	O14672	O14672	O14672	O14672
O15016	TRI66_HUMAN	TRIM66	Tripartite motif-containing protein 66	regulation	other (intracellular compartments)	O15016	O15016	O15016	O15016	O15016	O15016
O15018	PDZD2_HUMAN	PDZD2	PDZ domain-containing protein 2 (Activated in prostate cancer protein)	other	endoplasmic reticulum	O15018	O15018	O15018	O15018	O15018	O15018
O15162	PLS1_HUMAN	PLSCR1	Phospholipid scramblase 1 (PL scramblase 1)	acute phase response	extracellular matrix	O15162	O15162	O15162	O15162	O15162	O15162
O15232	MATN3_HUMAN	MATN3	Matrilin-3	metabolism	extracellular matrix	O15232	O15232	O15232	O15232	O15232	O15232
O15498	YKT6_HUMAN	YKT6	Synaptobrevin homolog YKT6 (EC 2.3.1.-)	transport	integral component of membrane	O15498	O15498	O15498	O15498	O15498	O15498
O43581	SYT7_HUMAN	SYT7	Synaptotagmin-7 (IPCA-7)	response (other)	integral component of membrane	O43581	O43581	O43581	O43581	O43581	O43581
O43768	ENSA_HUMAN	ENSA	Alpha-endosulfine (ARPP-19e)	response to nutrient	other (intracellular compartments)	O43768	O43768	O43768	#NV	#NV	O43768
O60266	ADCY3_HUMAN	ADCY3	Adenylate cyclase type 3 (EC 4.6.1.1) (ATP pyrophosphate-lyase 3)	response to nutrient	integral component of membrane	O60266	O60266	O60266	O60266	O60266	O60266
O60543	CIDEA_HUMAN	CIDEA	Cell death activator CIDE-A (Cell death-inducing DFFA-like effector A)	adaptive immune response	lipid droplet	O60543	O60543	O60543	O60543	O60543	O60543
O60664	PLIN3_HUMAN	PLIN3	Perilipin-3 (47 kDa mannose 6-phosphate receptor-binding protein)	transport	lipid droplet	O60664	O60664	O60664	O60664	O60664	O60664
O75888	TNF13_HUMAN	TNFSF13	Tumor necrosis factor ligand superfamily member 13 (A proliferation-inducing ligand)	adaptive immune response	extracellular exosome	O75888	O75888	O75888	O75888	O75888	O75888
O75954	TSN9_HUMAN	TSPAN9	Tetraspanin-9 (Tspan-9)	other	integral component of membrane	O75954	O75954	O75954	O75954	O75954	O75954
O94985	CSTN1_HUMAN	CLSTN1	Calsyntenin-1 (Alcadein-alpha)	regulation	integral component of membrane	O94985	O94985	O94985	O94985	O94985	O94985
O95436	NPT2B_HUMAN	SLC34A2	Sodium-dependent phosphate transport protein 2B	response to hormone	integral component of membrane	O95436	O95436	#NV	#NV	#NV	O95436
P00709	LALBA_HUMAN	LALBA	Alpha-lactalbumin (Lactose synthase B protein)	antimicrobial response	extracellular region/space	P00709	P00709	P00709	P00709	#NV	P00709
P00734	THRB_HUMAN	F2	Prothrombin (EC 3.4.21.5) (Coagulation factor II)	antimicrobial response	extracellular exosome	P00734	P00734	P00734	P00734	P00734	P00734
P00738	HPT_HUMAN	HP	Haptoglobin (Zonulin)	acute phase response	extracellular exosome	P00738	P00738	P00738	P00738	P00738	P00738
P00747	PLMN_HUMAN	PLG	Plasminogen (EC 3.4.21.7)	migration	extracellular matrix	P00747	P00747	P00747	P00747	P00747	P00747
P01023	A2MG_HUMAN	A2M	Alpha-2-macroglobulin (Alpha-2-M)	regulation	extracellular matrix	P01023	P01023	#NV	P01023	P01023	#NV
P01024	CO3_HUMAN	C3	Complement C3 (C3 and PZP-like alpha-2-macroglobulin domain-containing protein 1)	adaptive immune response	extracellular exosome	P01024	P01024	P01024	P01024	P01024	P01024
P01133	EGF_HUMAN	EGF	Pro-epidermal growth factor (EGF)	migration	integral component of membrane	P01133	P01133	#NV	P01133	P01133	P01133
P01833	PIGR_HUMAN	PIGR	Polymeric immunoglobulin receptor (PIgR)	other	integral component of membrane	P01833	P01833	P01833	P01833	P01833	P01833
P02647	APOA1_HUMAN	APOA1	Apolipoprotein A-I (Apo-AI)	adaptive immune response	lipoprotein particle	P02647	P02647	P02647	P02647	P02647	P02647
P02649	APOE_HUMAN	APOE	Apolipoprotein E (Apo-E)	adaptive immune response	lipoprotein particle	P02649	P02649	P02649	P02649	P02649	P02649
P02654	APOC1_HUMAN	APOC1	Apolipoprotein C-I (Apo-CI)	regulation	lipoprotein particle	P02654	P02654	#NV	P02654	#NV	#NV
P02656	APOC3_HUMAN	APOC3	Apolipoprotein C-III (Apo-CIII)	regulation	lipoprotein particle	P02656	P02656	P02656	P02656	P02656	P02656
P02671	FIBA_HUMAN	FGA	Fibrinogen alpha chain	adaptive immune response	extracellular matrix	P02671	P02671	P02671	P02671	P02671	P02671
P02675	FIBB_HUMAN	FGB	Fibrinogen beta chain	adaptive immune response	extracellular matrix	P02675	P02675	P02675	P02675	P02675	P02675
P02679	FIBG_HUMAN	FGG	Fibrinogen gamma chain	response to cytokine	extracellular matrix	P02679	P02679	P02679	P02679	P02679	P02679
P02788	TRFL_HUMAN	LTF	Lactotransferrin (Lactoferrin) (EC 3.4.21.-) (Growth-inhibiting protein 12)	antimicrobial response	extracellular exosome	P02788	P02788	P02788	P02788	P02788	P02788
P02810	PRPC_HUMAN	PRH1; PRH2	Salivary acidic proline-rich phosphoprotein 1/2 (Db-s) (PRP-1/PRP-2)	other	extracellular region/space	P02810	P02810	P02810	P02810	P02810	P02810
P04233	HG2A_HUMAN	CD74	HLA class II histocompatibility antigen gamma chain (HLA-DR antigens-associated invariant chain)	adaptive immune response	integral component of membrane	P04233	P04233	P04233	P04233	P04233	P04233
P04792	HSPB1_HUMAN	HSPB1	Heat shock protein beta-1 (HspB1) (28 kDa heat shock protein)	response to growth factor	extracellular exosome	P04792	P04792	P04792	P04792	P04792	P04792
P04843	RPN1_HUMAN	RPN1	Dolichyl-diphosphooligosaccharide--protein glycosyltransferase subunit 1 (Dolichyl-diphosphooligosaccharide--protein glycosyltransferase 67 kDa subunit)	other	integral component of membrane	P04843	P04843	P04843	P04843	#NV	P04843
P05067	A4_HUMAN	APP	Amyloid-beta precursor protein (APP)	antimicrobial response	integral component of membrane	P05067	P05067	P05067	P05067	P05067	P05067
P05413	FABPH_HUMAN	FABP3	Fatty acid-binding protein, heart (Fatty acid-binding protein 3)	response (other)	extracellular region/space	P05413	#NV	#NV	P05413	#NV	#NV
P05783	K1C18_HUMAN	KRT18	Keratin, type I cytoskeletal 18 (Cell proliferation-inducing gene 46 protein)	regulation	extracellular exosome	P05783	P05783	P05783	P05783	#NV	P05783
P05814	CASB_HUMAN	CSN2	Beta-casein	regulation	extracellular region/space	P05814	P05814	P05814	P05814	P05814	P05814
P06454	PTMA_HUMAN	PTMA	Prothymosin alpha	regulation	other (intracellular compartments)	P06454	P06454	#NV	#NV	#NV	#NV
P06858	LIPL_HUMAN	LPL	Lipoprotein lipase (LPL) (EC 3.1.1.34)	response to nutrient	lipoprotein particle	P06858	P06858	P06858	P06858	P06858	P06858
P07195	LDHB_HUMAN	LDHB	L-lactate dehydrogenase B chain (LDH-B) (EC 1.1.1.27)	metabolism	extracellular exosome	P07195	P07195	P07195	#NV	P07195	#NV
P07498	CASK_HUMAN	CSN3	Kappa-casein	transport	extracellular region/space	P07498	P07498	P07498	P07498	P07498	P07498
P07900	HS90A_HUMAN	HSP90AA1	Heat shock protein HSP 90-alpha (Heat shock 86 kDa)	response to stress	extracellular exosome	P07900	P07900	P07900	P07900	P07900	P07900
P07910	HNRPC_HUMAN	HNRNPC	Heterogeneous nuclear ribonucleoproteins C1/C2 (hnRNP C1/C2)	regulation	extracellular exosome	P07910	P07910	P07910	P07910	P07910	P07910
P08571	CD14_HUMAN	CD14	Monocyte differentiation antigen CD14 (Myeloid cell-specific leucine-rich glycoprotein)	adaptive immune response	integral component of membrane	P08571	P08571	P08571	#NV	#NV	P08571
P09603	CSF1_HUMAN	CSF1	Macrophage colony-stimulating factor 1 (CSF-1)	adaptive immune response	integral component of membrane	P09603	P09603	P09603	P09603	P09603	P09603
P0C0L4	CO4A_HUMAN	C4A	Complement C4-A (Acidic complement C4)	adaptive immune response	extracellular exosome	P0C0L4	P0C0L4	P0C0L4	P0C0L4	P0C0L4	P0C0L4

P0DJI8	SAA1_HUMAN	SAA1	Serum amyloid A-1 protein (SAA)	acute phase response	lipoprotein particle	P0DJI8	P0DJI8	P0DJI8	P0DJI8	P0DJI8	P0DJI8
P0DJI9	SAA2_HUMAN	SAA2	Serum amyloid A-2 protein (SAA2)	acute phase response	lipoprotein particle	P0DJI9	P0DJI9	P0DJI9	P0DJI9	P0DJI9	P0DJI9
P10323	ACRO_HUMAN	ACR	Acrosin (EC 3.4.21.10)	response to hormone	extracellular exosome	P10323	P10323	P10323	P10323	P10323	P10323
P10412	H14_HUMAN	H1-4	Histone H1.4 (Histone H1b)	regulation	other (intracellular compartments)	P10412	P10412	P10412	P10412	P10412	P10412
P10451	OSTP_HUMAN	SPP1	Osteopontin (Bone sialoprotein 1)	response to hormone	extracellular exosome	P10451	P10451	P10451	P10451	P10451	P10451
P10586	PTPRF_HUMAN	PTPRF	Receptor-type tyrosine-protein phosphatase F (EC 3.1.3.48)	migration	integral component of membrane	P10586	P10586	P10586	P10586	P10586	P10586
P10909	CLUS_HUMAN	CLU	Clusterin (Aging-associated gene 4 protein) (Apolipoprotein J)	antimicrobial response	extracellular matrix	P10909	P10909	P10909	P10909	P10909	P10909
P11021	BIP_HUMAN	HSPA5	Endoplasmic reticulum chaperone BiP (EC 3.6.4.10) (78 kDa glucose-regulated protein)	response to nutrient	integral component of membrane	P11021	P11021	P11021	P11021	P11021	P11021
P12272	PTHR_HUMAN	PTHLH	Parathyroid hormone-related protein (PTH-rP)	regulation	extracellular region/space	P12272	P12272	P12272	P12272	P12272	P12272
P13746	HLAA_HUMAN	HLA-A	HLA class I histocompatibility antigen, A alpha chain (Human leukocyte antigen A) (HLA-A)	adaptive immune response	endoplasmic reticulum	P13746	P13746	P13746	P13746	P13746	P13746
P13747	HLAE_HUMAN	HLA-E	HLA class I histocompatibility antigen, alpha chain E (MHC class I antigen E)	adaptive immune response	integral component of membrane	P13747	P13747	P13747	P13747	P13747	P13747
P15291	B4GT1_HUMAN	B4GALT1	Beta-1,4-galactosyltransferase 1 (Beta-1,4-GalTase 1)	acute phase response	integral component of membrane	P15291	P15291	P15291	P15291	P15291	P15291
P15515	HIS1_HUMAN	HTN1	Histatin-1 (Histidine-rich protein 1)	antimicrobial response	extracellular region/space	P15515	P15515	P15515	#NV	P15515	P15515
P15907	SIAT1_HUMAN	ST6GAL1	Beta-galactoside alpha-2,6-sialyltransferase 1 (Alpha 2,6-ST 1) (EC 2.4.99.1)	adaptive immune response	integral component of membrane	P15907	P15907	P15907	P15907	P15907	P15907
P15941	MUC1_HUMAN	MUC1	Mucin-1 (MUC-1) (Breast carcinoma-associated antigen DF3)	response to stress	integral component of membrane	P15941	P15941	P15941	P15941	P15941	P15941
P17987	TCPA_HUMAN	TCP1	T-complex protein 1 subunit alpha (TCP-1-alpha)	regulation	extracellular exosome	P17987	P17987	P17987	P17987	P17987	P17987
P18827	SDC1_HUMAN	SDC1	Syndecan-1 (SYND1)	response (other)	integral component of membrane	P18827	P18827	P18827	P18827	P18827	P18827
P19440	GGT1_HUMAN	GGT1	Glutathione hydrolase 1 proenzyme (EC 3.4.19.13) (Gamma-glutamyltransferase 1)	adaptive immune response	integral component of membrane	P19440	P19440	P19440	P19440	P19440	P19440
P19634	SL9A1_HUMAN	SLC9A1	Sodium/hydrogen exchanger 1 (APNH) (Na(+)/H(+) antiporter, amiloride-sensitive)	response to hypoxia	integral component of membrane	P19634	P19634	P19634	#NV	P19634	P19634
P19835	CEL_HUMAN	CEL	Bile salt-activated lipase (BAL) (EC 3.1.1.13)	metabolism	integral component of membrane	P19835	P19835	P19835	P19835	P19835	P19835
P20138	CD33_HUMAN	CD33	Myeloid cell surface antigen CD33 (Sialic acid-binding Ig-like lectin 3)	adaptive immune response	integral component of membrane	P20138	P20138	P20138	P20138	P20138	P20138
P20671	H2A1D_HUMAN	H2AC7	Histone H2A type 1-D (Histone H2A.3)	other	extracellular exosome	P20671	P20671	#NV	#NV	P20671	P20671
P22079	PERL_HUMAN	LPO	Lactoperoxidase (LPO) (EC 1.11.1.7) (Salivary peroxidase)	antimicrobial response	extracellular exosome	P22079	P22079	P22079	P22079	P22079	P22079
P22897	MRC1_HUMAN	MRC1	Macrophage mannose receptor 1 (MMR) (C-type lectin domain family 13 member D)	response to cytokine	integral component of membrane	P22897	P22897	P22897	P22897	P22897	P22897
P24821	TENA_HUMAN	TNC	Tenascin (TN) (Cytotactin) (GMEM)	response (other)	extracellular matrix	P24821	P24821	P24821	P24821	P24821	P24821
P24844	MYL9_HUMAN	MYL9	Myosin regulatory light polypeptide 9 (20 kDa myosin light chain)	regulation	other (intracellular compartments)	P24844	P24844	P24844	P24844	P24844	P24844
P28300	LYOX_HUMAN	LOX	Protein-lysine 6-oxidase (EC 1.4.3.13) (Lysyl oxidase)	response to hormone	extracellular matrix	P28300	P28300	P28300	P28300	P28300	P28300
P30443	HLAA_HUMAN	HLA-A	HLA class I histocompatibility antigen, A alpha chain	adaptive immune response	endoplasmic reticulum	P30443	#NV	#NV	P30443	P30443	P30443
P30510	HLAC_HUMAN	HLA-C	HLA class I histocompatibility antigen, C alpha chain (HLA-C)	adaptive immune response	endoplasmic reticulum	P30510	P30510	P30510	P30510	P30510	P30510
P31431	SDC4_HUMAN	SDC4	Syndecan-4 (SYND4)	migration	integral component of membrane	P31431	P31431	P31431	P31431	P31431	P31431
P33121	ACSL1_HUMAN	ACSL1	Long-chain-fatty-acid--CoA ligase 1 (EC 6.2.1.3)	response to nutrient	integral component of membrane	P33121	P33121	P33121	P33121	P33121	P33121
P34741	SDC2_HUMAN	SDC2	Syndecan-2 (SYND2) (Fibroglycan)	migration	extracellular matrix	P34741	P34741	P34741	P34741	P34741	P34741
P35527	K1C9_HUMAN	KRT9	Keratin, type I cytoskeletal 9 (Cytokeratin-9)	other	extracellular exosome	P35527	P35527	P35527	P35527	P35527	P35527
P35542	SAA4_HUMAN	SAA4	Serum amyloid A-4 protein (Constitutively expressed serum amyloid A protein)	acute phase response	lipoprotein particle	P35542	P35542	P35542	P35542	P35542	P35542
P35606	COPB2_HUMAN	COPB2	Coatomer subunit beta' (Beta'-coat protein)	transport	endoplasmic reticulum	P35606	P35606	P35606	P35606	P35606	P35606
P36578	RL4_HUMAN	RPL4	60S ribosomal protein L4 (60S ribosomal protein L1)	RNA catabolic process	extracellular exosome	P36578	#NV	P36578	P36578	#NV	P36578
P37802	TAGL2_HUMAN	TAGLN2	Transgelin-2 (Epididymis tissue protein Li 7e)	other	extracellular exosome	P37802	P37802	P37802	P37802	P37802	P37802
P39019	RS19_HUMAN	RPS19	40S ribosomal protein S19 (Small ribosomal subunit protein eS19)	antimicrobial response	extracellular exosome	P39019	P39019	P39019	P39019	P39019	P39019
P46778	RL21_HUMAN	RPL21	60S ribosomal protein L21 (Large ribosomal subunit protein eL21)	RNA catabolic process	endoplasmic reticulum	P46778	P46778	P46778	P46778	#NV	P46778
P47710	CASA1_HUMAN	CSN1S1	Alpha-S1-casein	response to hormone	extracellular region/space	P47710	P47710	P47710	P47710	P47710	P47710
P47914	RL29_HUMAN	RPL29	60S ribosomal protein L29 (Cell surface heparin-binding protein HIP)	RNA catabolic process	other (intracellular compartments)	P47914	#NV	#NV	P47914	#NV	#NV
P47989	XDH_HUMAN	XDH	Xanthine dehydrogenase/oxidase	regulation	extracellular region/space	P47989	P47989	P47989	P47989	P47989	P47989
P49327	FAS_HUMAN	FASN	Fatty acid synthase (EC 2.3.1.85)	response to cytokine	extracellular exosome	P49327	P49327	P49327	P49327	P49327	P49327
P50591	TNF10_HUMAN	TNFSF10	Tumor necrosis factor ligand superfamily member 10 (Apo-2 ligand)	adaptive immune response	integral component of membrane	P50591	P50591	P50591	P50591	P50591	P50591
P53999	TCP4_HUMAN	SUB1	Activated RNA polymerase II transcriptional coactivator p15 (Positive cofactor 4)	regulation	extracellular exosome	P53999	P53999	P53999	P53999	P53999	P53999
P55036	PSMD4_HUMAN	PSMD4	26S proteasome non-ATPase regulatory subunit 4 (26S proteasome regulatory subunit RPN10)	response to hypoxia	other (intracellular compartments)	P55036	P55036	P55036	P55036	P55036	P55036
P55327	TPD52_HUMAN	TPD52	Tumor protein D52 (Protein N8)	other	endoplasmic reticulum	P55327	P55327	P55327	P55327	P55327	P55327
P60033	CD81_HUMAN	CD81	CD81 antigen (26 kDa cell surface protein TAPA-1)	adaptive immune response	integral component of membrane	P60033	P60033	P60033	P60033	P60033	P60033
P60468	SC61B_HUMAN	SEC61B	Protein transport protein Sec61 subunit beta	transport	integral component of membrane	P60468	P60468	P60468	P60468	P60468	P60468
P60709	ACTB_HUMAN	ACTB	Actin, cytoplasmic 1 (Beta-actin)	response (other)	extracellular exosome	P60709	P60709	P60709	P60709	P60709	P60709
P60866	RS20_HUMAN	RPS20	40S ribosomal protein S20 (Small ribosomal subunit protein uS10)	RNA catabolic process	extracellular exosome	P60866	P60866	P60866	P60866	P60866	P60866
P61769	B2MG_HUMAN	B2M	Beta-2-microglobulin	antimicrobial response	extracellular exosome	P61769	P61769	#NV	P61769	P61769	P61769
P62269	RS18_HUMAN	RPS18	40S ribosomal protein S18 (Ke-3)	RNA catabolic process	extracellular exosome	P62269	P62269	P62269	#NV	P62269	P62269
P62277	RS13_HUMAN	RPS13	40S ribosomal protein S13 (Small ribosomal subunit protein uS15)	regulation	extracellular exosome	P62277	P62277	P62277	P62277	P62277	P62277
P62306	RUXF_HUMAN	SNRPF	Small nuclear ribonucleoprotein F (snRNP-F)	metabolism	other (intracellular compartments)	P62306	P62306	#NV	#NV	#NV	#NV
P62328	TYB4_HUMAN	TMSB4X	Thymosin beta-4 (T beta-4) (Fx)	response (other)	extracellular region/space	P62328	P62328	P62328	P62328	P62328	P62328
P62333	PRS10_HUMAN	PSMC6	26S proteasome regulatory subunit 10B (26S proteasome AAA-ATPase subunit RPT4)	response to hypoxia	extracellular exosome	P62333	P62333	P62333	P62333	P62333	P62333
P62750	RL23A_HUMAN	RPL23A	60S ribosomal protein L23a (Large ribosomal subunit protein uL23)	RNA catabolic process	extracellular exosome	P62750	P62750	P62750	#NV	P62750	P62750
P62841	RS15_HUMAN	RPS15	40S ribosomal protein S15 (RIG protein)	RNA catabolic process	other (intracellular compartments)	P62841	#NV	#NV	#NV	P62841	#NV
P62937	PPIA_HUMAN	PPIA	Peptidyl-prolyl cis-trans isomerase A (PPIase A) (EC 5.2.1.8) (Cyclophilin A)	migration	extracellular exosome	P62937	P62937	P62937	P62937	#NV	P62937
P63220	RS21_HUMAN	RPS21	40S ribosomal protein S21 (Small ribosomal subunit protein eS21)	RNA catabolic process	endoplasmic reticulum	P63220	P63220	P63220	P63220	#NV	P63220
P63313	TYB10_HUMAN	TMSB10	Thymosin beta-10	migration	other (intracellular compartments)	P63313	P63313	P63313	P63313	P63313	P63313
P68104	EF1A1_HUMAN	EEF1A1	Elongation factor 1-alpha 1 (EF-1-alpha-1)	response to growth factor	extracellular exosome	P68104	P68104	P68104	P68104	P68104	P68104

P68133	ACTS_HUMAN	ACTA1	Actin, alpha skeletal muscle (Alpha-actin-1)	response to nutrient	extracellular exosome	P68133	P68133	P68133	P68133	#NV	P68133
P78324	SHPS1_HUMAN	SIRPA	Tyrosine-protein phosphatase non-receptor type substrate 1 (SHP substrate 1)	response to cytokine	integral component of membrane	P78324	P78324	P78324	P78324	P78324	P78324
P80303	NUCB2_HUMAN	NUCB2	Nucleobindin-2 (DNA-binding protein NEFA)	regulation	extracellular exosome	P80303	P80303	P80303	P80303	P80303	P80303
P98082	DAB2_HUMAN	DAB2	Disabled homolog 2 (Adaptor molecule disabled-2)	migration	integral component of membrane	P98082	P98082	#NV	#NV	#NV	P98082
Q02818	NUCB1_HUMAN	NUCB1	Nucleobindin-1 (CALNUC)	response (other)	extracellular exosome	Q02818	Q02818	Q02818	Q02818	#NV	#NV
Q04656	ATP7A_HUMAN	ATP7A	Copper-transporting ATPase 1 (EC 7.2.2.8) (Copper pump 1)	antimicrobial response	integral component of membrane	Q04656	Q04656	Q04656	Q04656	Q04656	Q04656
Q07000	HLAC_HUMAN	HLA-C	HLA class I histocompatibility antigen, C alpha chain (HLA-C)	adaptive immune response	endoplasmic reticulum	Q07000	Q07000	Q07000	Q07000	Q07000	Q07000
Q08380	LG3BP_HUMAN	LGALS3BP	Galectin-3-binding protein (Basement membrane autoantigen p105)	response (other)	extracellular matrix	Q08380	Q08380	Q08380	Q08380	Q08380	Q08380
Q08431	MFGM_HUMAN	MFGE8	Lactadherin (Breast epithelial antigen BA46) (HMFG)	regulation	extracellular matrix	Q08431	Q08431	Q08431	Q08431	#NV	Q08431
Q09666	AHNK_HUMAN	AHNAK	Neuroblast differentiation-associated protein AHNAK (Desmoyokin)	regulation	extracellular exosome	Q09666	Q09666	Q09666	Q09666	Q09666	Q09666
Q10471	GALT2_HUMAN	GALNT2	Polypeptide N-acetylgalactosaminyltransferase 2 (EC 2.4.1.41)	other	integral component of membrane	Q10471	Q10471	Q10471	Q10471	Q10471	Q10471
Q12830	BPTF_HUMAN	BPTF	Nucleosome-remodeling factor subunit BPTF (Bromodomain and PHD finger-containing transcription factor)	regulation	extracellular exosome	Q12830	Q12830	Q12830	Q12830	Q12830	Q12830
Q12913	PTPRJ_HUMAN	PTPRJ	Receptor-type tyrosine-protein phosphatase eta (Protein-tyrosine phosphatase eta)	migration	integral component of membrane	Q12913	Q12913	Q12913	Q12913	Q12913	Q12913
Q13113	PDZ1I_HUMAN	PDZK1IP1	PDZK1-interacting protein 1 (17 kDa membrane-associated protein)	other	integral component of membrane	Q13113	Q13113	Q13113	#NV	Q13113	Q13113
Q13410	BT1A1_HUMAN	BTN1A1	Butyrophilin subfamily 1 member A1 (BT)	adaptive immune response	integral component of membrane	Q13410	Q13410	Q13410	Q13410	Q13410	Q13410
Q13438	OS9_HUMAN	OS9	Protein OS-9 (Amplified in osteosarcoma 9)	response to stress	endoplasmic reticulum	Q13438	Q13438	Q13438	Q13438	Q13438	Q13438
Q14512	FGFP1_HUMAN	FGFBP1	Fibroblast growth factor-binding protein 1 (FGF-BP)	migration	extracellular region/space	Q14512	Q14512	Q14512	Q14512	Q14512	Q14512
Q14766	LTBP1_HUMAN	LTBP1	Latent-transforming growth factor beta-binding protein 1 (LTBP-1)	regulation	extracellular matrix	Q14766	Q14766	Q14766	Q14766	Q14766	Q14766
Q14802	FXYD3_HUMAN	FXYD3	FXYD domain-containing ion transport regulator 3 (Chloride conductance inducer protein Mat-8)	regulation	integral component of membrane	Q14802	Q14802	Q14802	Q14802	Q14802	Q14802
Q14839	CHD4_HUMAN	CHD4	Chromodomain-helicase-DNA-binding protein 4 (CHD-4) (EC 3.6.4.12)	regulation	other (intracellular compartments)	Q14839	Q14839	#NV	#NV	Q14839	Q14839
Q15365	PCBP1_HUMAN	PCBP1	Poly(rC)-binding protein 1 (Alpha-CP1) (Heterogeneous nuclear ribonucleoprotein E1)	regulation	extracellular exosome	Q15365	Q15365	Q15365	Q15365	Q15365	Q15365
Q15904	VAS1_HUMAN	ATP6AP1	V-type proton ATPase subunit S1 (V-ATPase subunit S1)	response (other)	integral component of membrane	Q15904	Q15904	Q15904	Q15904	Q15904	Q15904
Q16181	SEPT7_HUMAN	SEPTIN7	Septin-7 (CDC10 protein homolog)	migration	extracellular exosome	Q16181	Q16181	Q16181	Q16181	Q16181	Q16181
Q16518	RPE65_HUMAN	RPE65	Retinoid isomerohydrolase (EC 3.1.1.64) (All-trans-retinyl-palmitate hydrolase)	response (other)	endoplasmic reticulum	Q16518	Q16518	Q16518	Q16518	Q16518	Q16518
Q16625	OCLN_HUMAN	OCLN	Occludin	response to cytokine	integral component of membrane	Q16625	Q16625	Q16625	Q16625	Q16625	Q16625
Q16851	UGPA_HUMAN	UGP2	UTP--glucose-1-phosphate uridylyltransferase (EC 2.7.7.9) (UDP-glucose pyrophosphorylase)	metabolism	extracellular exosome	Q16851	Q16851	Q16851	Q16851	Q16851	Q16851
Q2I0M4	LRC26_HUMAN	LRRC26	Leucine-rich repeat-containing protein 26 (BK channel auxiliary gamma subunit LRRC26)	regulation	integral component of membrane	Q2I0M4	Q2I0M4	Q2I0M4	Q2I0M4	Q2I0M4	Q2I0M4
Q53GQ0	DHB12_HUMAN	HSD17B12	Very-long-chain 3-oxoacyl-CoA reductase (EC 1.1.1.330) (17-beta-hydroxysteroid dehydrogenase 12)	regulation	extracellular matrix	Q53GQ0	Q53GQ0	Q53GQ0	Q53GQ0	Q53GQ0	Q53GQ0
Q5JWF2	GNAS1_HUMAN	GNAS	Guanine nucleotide-binding protein G(s) subunit alpha isoforms XLas	regulation	extracellular exosome	Q5JWF2	Q5JWF2	#NV	Q5JWF2	Q5JWF2	Q5JWF2
Q6UX71	PXDC2_HUMAN	PLXDC2	Plexin domain-containing protein 2 (Tumor endothelial marker 7-related protein)	other	integral component of membrane	Q6UX71	Q6UX71	Q6UX71	Q6UX71	Q6UX71	Q6UX71
Q6UXA7	CF015_HUMAN	C6orf15	Uncharacterized protein C6orf15 (Protein STG)	other	extracellular region/space	Q6UXA7	Q6UXA7	#NV	Q6UXA7	Q6UXA7	Q6UXA7
Q6WN34	CRDL2_HUMAN	CHRDL2	Chordin-like protein 2 (Breast tumor novel factor 1) (BNF-1) (Chordin-related protein 2)	other	extracellular region/space	Q6WN34	Q6WN34	Q6WN34	Q6WN34	Q6WN34	Q6WN34
Q6ZS81	WDFY4_HUMAN	WDFY4	WD repeat- and FYVE domain-containing protein 4	other	other (intracellular compartments)	Q6ZS81	Q6ZS81	Q6ZS81	Q6ZS81	Q6ZS81	Q6ZS81
Q7Z7D3	VTCN1_HUMAN	VTCN1	V-set domain-containing T-cell activation inhibitor 1 (B7 homolog 4)	adaptive immune response	integral component of membrane	Q7Z7D3	Q7Z7D3	Q7Z7D3	Q7Z7D3	Q7Z7D3	Q7Z7D3
Q86X10	RLGPB_HUMAN	RALGAPB	Ral GTPase-activating protein subunit beta (p170)	regulation	other (intracellular compartments)	Q86X10	Q86X10	Q86X10	Q86X10	Q86X10	Q86X10
Q86X29	LSR_HUMAN	LSR	Lipolysis-stimulated lipoprotein receptor	regulation	lipoprotein particle	Q86X29	Q86X29	Q86X29	Q86X29	Q86X29	Q86X29
Q86Y38	XYLT1_HUMAN	XYLT1	Xylosyltransferase 1 (EC 2.4.2.26) (Peptide O-xylosyltransferase 1)	metabolism	integral component of membrane	Q86Y38	Q86Y38	Q86Y38	Q86Y38	Q86Y38	Q86Y38
Q8IZA0	K319L_HUMAN	KIAA0319L	Dyslexia-associated protein KIAA0319-like protein (Adeno-associated virus receptor) (AAVR)	other	integral component of membrane	Q8IZA0	Q8IZA0	Q8IZA0	Q8IZA0	Q8IZA0	Q8IZA0
Q8IZC6	CORA1_HUMAN	COL27A1	Collagen alpha-1(XXVII) chain	other	extracellular matrix	Q8IZC6	Q8IZC6	Q8IZC6	Q8IZC6	Q8IZC6	Q8IZC6
Q8N114	SHSA5_HUMAN	SHISA5	Protein shisa-5 (Putative NF-kappa-B-activating protein 120) (Scotin)	response (other)	integral component of membrane	Q8N114	Q8N114	#NV	#NV	Q8N114	Q8N114
Q8N387	MUC15_HUMAN	MUC15	Mucin-15 (MUC-15)	other	integral component of membrane	Q8N387	Q8N387	Q8N387	Q8N387	Q8N387	Q8N387
Q8N474	SFRP1_HUMAN	SFRP1	Secreted frizzled-related protein 1 (FRP-1) (sFRP-1)	response to hypoxia	extracellular matrix	Q8N474	Q8N474	Q8N474	Q8N474	Q8N474	Q8N474
Q8N7A1	KLDC1_HUMAN	KLHDC1	Kelch domain-containing protein 1	other	other (intracellular compartments)	Q8N7A1	Q8N7A1	Q8N7A1	Q8N7A1	Q8N7A1	Q8N7A1
Q8N9U0	TAC2N_HUMAN	TC2N	Tandem C2 domains nuclear protein (Membrane targeting tandem C2 domain-containing protein 1)	other	other (intracellular compartments)	Q8N9U0	Q8N9U0	Q8N9U0	Q8N9U0	Q8N9U0	Q8N9U0
Q8NBJ4	GOLM1_HUMAN	GOLM1	Golgi membrane protein 1 (Golgi membrane protein GP73) (Golgi phosphoprotein 2)	regulation	extracellular region/space	Q8NBJ4	Q8NBJ4	Q8NBJ4	Q8NBJ4	Q8NBJ4	Q8NBJ4
Q8NES3	LFNG_HUMAN	LFNG	Beta-1,3-N-acetylglucosaminyltransferase lunatic fringe (EC 2.4.1.222)	regulation	integral component of membrane	Q8NES3	Q8NES3	Q8NES3	Q8NES3	Q8NES3	Q8NES3
Q8NFU4	FDSCP_HUMAN	FDCSP	Follicular dendritic cell secreted peptide (FDC secreted protein) (FDC-SP)	other	extracellular region/space	Q8NFU4	Q8NFU4	Q8NFU4	Q8NFU4	Q8NFU4	Q8NFU4
Q8NI22	MCFD2_HUMAN	MCFD2	Multiple coagulation factor deficiency protein 2 (Neural stem cell-derived neuronal survival protein)	transport	endoplasmic reticulum	Q8NI22	Q8NI22	Q8NI22	Q8NI22	Q8NI22	Q8NI22
Q8WUH6	TM263_HUMAN	TMEM263	Transmembrane protein 263	other	integral component of membrane	Q8WUH6	Q8WUH6	Q8WUH6	Q8WUH6	Q8WUH6	Q8WUH6
Q92673	SORL_HUMAN	SORL1	Sortilin-related receptor (Low-density lipoprotein receptor relative with 11 ligand-binding repeats)	migration	integral component of membrane	Q92673	Q92673	Q92673	Q92673	Q92673	Q92673
Q92896	GSLG1_HUMAN	GLG1	Golgi apparatus protein 1 (CFR-1) (Cysteine-rich fibroblast growth factor receptor)	migration	extracellular matrix	Q92896	Q92896	Q92896	Q92896	Q92896	Q92896
P10321	HLAC_HUMAN	HLA-C	HLA class I histocompatibility antigen, C alpha chain (HLA-C)	adaptive immune response	extracellular exosome	Q95604	Q95604	Q95604	Q95604	Q95604	Q95604
Q969T9	WBP2_HUMAN	WBP2	WW domain-binding protein 2 (WBP-2)	response to hormone	other (intracellular compartments)	Q969T9	Q969T9	Q969T9	Q969T9	Q969T9	Q969T9
Q96A22	CK052_HUMAN	C11orf52	Uncharacterized protein C11orf52	other	extracellular exosome	Q96A22	Q96A22	Q96A22	Q96A22	Q96A22	Q96A22
Q96A33	CCD47_HUMAN	CCDC47	Coiled-coil domain-containing protein 47 (Calumin)	response (other)	integral component of membrane	Q96A33	Q96A33	Q96A33	Q96A33	Q96A33	Q96A33
Q96AD5	PLPL2_HUMAN	PNPLA2	Patatin-like phospholipase domain-containing protein 2 (EC 3.1.1.3)	regulation	lipid droplet	Q96AD5	Q96AD5	Q96AD5	Q96AD5	Q96AD5	Q96AD5
Q96D31	CRCM1_HUMAN	ORAI1	Calcium release-activated calcium channel protein 1 (Protein orai-1)	adaptive immune response	integral component of membrane	Q96D31	Q96D31	Q96D31	Q96D31	Q96D31	Q96D31
Q96IU4	ABHEB_HUMAN	ABHD14B	Protein ABHD14B (EC 3.-.-.-) (Alpha/beta hydrolase domain-containing protein 14B)	regulation	extracellular exosome	Q96IU4	#NV	Q96IU4	Q96IU4	Q96IU4	Q96IU4
Q96IY4	CBPB2_HUMAN	CPB2	Carboxypeptidase B2 (EC 3.4.17.20) (Carboxypeptidase U)	response to nutrient	extracellular region/space	Q96IY4	Q96IY4	#NV	#NV	#NV	Q96IY4

Q96JH7	VCIP1_HUMAN	VCPIP1	Deubiquitinating protein VCIP135 (EC 3.4.19.12)	other	endoplasmic reticulum	Q96JH7	Q96JH7	Q96JH7	Q96JH7	Q96JH7	Q96JH7
Q96S86	HPLN3_HUMAN	HAPLN3	Hyaluronan and proteoglycan link protein 3	other	extracellular matrix	Q96S86	Q96S86	Q96S86	Q96S86	Q96S86	Q96S86
Q96S97	MYADM_HUMAN	MYADM	Myeloid-associated differentiation marker (Protein SB135)	migration	integral component of membrane	Q96S97	Q96S97	Q96S97	Q96S97	Q96S97	Q96S97
Q96SI9	STRBP_HUMAN	STRBP	Spermatid perinuclear RNA-binding protein	other	other (intracellular compartments)	Q96SI9	Q96SI9	Q96SI9	#NV	Q96SI9	Q96SI9
Q96T51	RUFY1_HUMAN	RUFY1	RUN and FYVE domain-containing protein 1 (FYVE-finger protein EIP1)	transport	other (intracellular compartments)	Q96T51	#NV	#NV	Q96T51	#NV	Q96T51
Q96TA1	NIBA2_HUMAN	NIBAN2	Protein Niban 2 (Meg-3) (Melanoma invasion by ERK)	regulation	extracellular exosome	Q96TA1	Q96TA1	#NV	#NV	Q96TA1	Q96TA1
Q99523	SORT_HUMAN	SORT1	Sortilin (100 kDa NT receptor) (Glycoprotein 95)	response (other)	integral component of membrane	Q99523	Q99523	Q99523	Q99523	Q99523	Q99523
Q99541	PLIN2_HUMAN	PLIN2	Perilipin-2 (Adipophilin)	response (other)	lipid droplet	Q99541	Q99541	Q99541	Q99541	Q99541	Q99541
Q9BW60	ELOV1_HUMAN	ELOVL1	Elongation of very long chain fatty acids protein 1 (EC 2.3.1.199)	metabolism	integral component of membrane	Q9BW60	Q9BW60	#NV	#NV	#NV	Q9BW60
Q9GZN4	BSSP4_HUMAN	PRSS22	Brain-specific serine protease 4 (BSSP-4) (EC 3.4.21.-) (Serine protease 22)	other	extracellular region/space	Q9GZN4	Q9GZN4	Q9GZN4	Q9GZN4	Q9GZN4	Q9GZN4
Q9GZU8	PIP30_HUMAN	PSME3IP1	PSME3-interacting protein (NEFA-interacting nuclear protein NIP30)	regulation	other (intracellular compartments)	Q9GZU8	Q9GZU8	Q9GZU8	Q9GZU8	Q9GZU8	Q9GZU8
Q9H173	SIL1_HUMAN	SIL1	Nucleotide exchange factor SIL1 (BiP-associated protein) (BAP)	transport	endoplasmic reticulum	Q9H173	Q9H173	Q9H173	Q9H173	Q9H173	Q9H173
Q9H3M7	TXNIP_HUMAN	TXNIP	Thioredoxin-interacting protein (Thioredoxin-binding protein 2)	response (other)	other (intracellular compartments)	Q9H3M7	Q9H3M7	Q9H3M7	Q9H3M7	Q9H3M7	Q9H3M7
Q9H3Z4	DNJC5_HUMAN	DNAJC5	DnaJ homolog subfamily C member 5 (Ceroid-lipofuscinosis neuronal protein 4)	regulation	integral component of membrane	Q9H3Z4	Q9H3Z4	Q9H3Z4	Q9H3Z4	Q9H3Z4	Q9H3Z4
Q9HB40	RISC_HUMAN	SCPEP1	Retinoid-inducible serine carboxypeptidase (EC 3.4.16.-) (Serine carboxypeptidase 1)	regulation	extracellular exosome	Q9HB40	Q9HB40	Q9HB40	Q9HB40	Q9HB40	Q9HB40
Q9NP72	RAB18_HUMAN	RAB18	Ras-related protein Rab-18	transport	integral component of membrane	Q9NP72	Q9NP72	Q9NP72	Q9NP72	Q9NP72	Q9NP72
Q9NQC3	RTN4_HUMAN	RTN4	Reticulon-4 (Foocen) (Neurite outgrowth inhibitor)	response to hypoxia	integral component of membrane	Q9NQC3	Q9NQC3	Q9NQC3	Q9NQC3	Q9NQC3	Q9NQC3
Q9NQR4	NIT2_HUMAN	NIT2	Omega-amidase NIT2 (EC 3.5.1.3) (Nitrilase homolog 2)	metabolism	extracellular exosome	Q9NQR4	Q9NQR4	Q9NQR4	Q9NQR4	Q9NQR4	Q9NQR4
Q9NRJ3	CCL28_HUMAN	CCL28	C-C motif chemokine 28 (Mucosae-associated epithelial chemokine) (MEC) (Protein CCK1)	antimicrobial response	extracellular region/space	Q9NRJ3	Q9NRJ3	Q9NRJ3	#NV	#NV	Q9NRJ3
Q9NRR3	C42S2_HUMAN	CDC42SE2	CDC42 small effector protein 2 (Small effector of CDC42 protein 2)	regulation	other (intracellular compartments)	Q9NRR3	Q9NRR3	Q9NRR3	Q9NRR3	Q9NRR3	Q9NRR3
Q9NS69	TOM22_HUMAN	TOMM22	Mitochondrial import receptor subunit TOM22 homolog (hTom22) (1C9-2)	regulation	integral component of membrane	Q9NS69	Q9NS69	Q9NS69	Q9NS69	Q9NS69	Q9NS69
Q9NV23	SAST_HUMAN	OLAH	S-acyl fatty acid synthase thioesterase, medium chain (EC 3.1.2.14)	other	other (intracellular compartments)	Q9NV23	Q9NV23	Q9NV23	#NV	#NV	#NV
Q9NZH0	GPC5B_HUMAN	GPRC5B	G-protein coupled receptor family C group 5 member B (A-69G12.1)	response (other)	integral component of membrane	Q9NZH0	Q9NZH0	Q9NZH0	Q9NZH0	Q9NZH0	Q9NZH0
Q9P2B7	CFA97_HUMAN	CFAP97	Cilia- and flagella-associated protein 97	other	other (intracellular compartments)	Q9P2B7	Q9P2B7	Q9P2B7	Q9P2B7	Q9P2B7	Q9P2B7
Q9UDW1	QCR9_HUMAN	UQCR10	Cytochrome b-c1 complex subunit 9 (Complex III subunit 9)	transport	other (intracellular compartments)	Q9UDW1	Q9UDW1	Q9UDW1	Q9UDW1	Q9UDW1	Q9UDW1
Q9UKW6	ELF5_HUMAN	ELF5	ETS-related transcription factor Elf-5 (E74-like factor 5) (Epithelium-restricted ESE-1-related Ets factor)	regulation	other (intracellular compartments)	Q9UKW6	#NV	Q9UKW6	Q9UKW6	Q9UKW6	Q9UKW6
Q9ULZ1	APEL_HUMAN	APLN	Apelin (APJ endogenous ligand) [Cleaved into: Apelin-36; Apelin-31; Apelin-28; Apelin-13]	adaptive immune response	extracellular region/space	Q9ULZ1	Q9ULZ1	#NV	Q9ULZ1	Q9ULZ1	Q9ULZ1
Q9UNQ0	ABCG2_HUMAN	ABCG2	Broad substrate specificity ATP-binding cassette transporter ABCG2 (EC 7.6.2.2) (ATP-binding cassette sub-family G member 2)	transport	integral component of membrane	Q9UNQ0	Q9UNQ0	Q9UNQ0	Q9UNQ0	Q9UNQ0	Q9UNQ0
Q9Y2K3	MYH15_HUMAN	MYH15	Myosin-15 (Myosin heavy chain 15)	other	other (intracellular compartments)	Q9Y2K3	Q9Y2K3	#NV	Q9Y2K3	Q9Y2K3	Q9Y2K3
Q9Y342	PLLP_HUMAN	PLLP	Plasmolipin (Plasma membrane proteolipid)	response to stress	integral component of membrane	Q9Y342	Q9Y342	Q9Y342	#NV	Q9Y342	Q9Y342
Q9Y653	AGRG1_HUMAN	ADGRG1	Adhesion G-protein coupled receptor G1 (G-protein coupled receptor 56)	migration	integral component of membrane	Q9Y653	Q9Y653	Q9Y653	#NV	Q9Y653	#NV
Q9Y679	AUP1_HUMAN	AUP1	Ancient ubiquitous protein 1	transport	extracellular exosome	Q9Y679	Q9Y679	Q9Y679	Q9Y679	Q9Y679	Q9Y679
